# Snakes and snakebites in the munduruku cosmology and medicine, central Brazilian Amazonia

**DOI:** 10.1371/journal.pntd.0014462

**Published:** 2026-06-22

**Authors:** Gisele Reis Dias, Gilza Reis Dias Lavareda, Talyson Aparício Gomes, Gilce Reis Dias da Silva, Geelle Sena de Melo, Mateus Chagas de Almeida, Maurício Lopes da Silva, Uziel Guaynê Oliveira, Fan Hui Wen, Jacqueline Sachett, Altair Seabra de Farias, Felipe Murta, Vinicius Azevedo Machado, Wuelton Monteiro

**Affiliations:** 1 School of Health Sciences, Universidade do Estado do Amazonas, Manaus, Brazil; 2 Department of Research, Fundação de Medicina Tropical Dr. Heitor Vieira Dourado, Manaus, Brazil; 3 Faculdade de Ciências Farmacêuticas, Universidade Federal do Amazonas, Manaus, Brazil; 4 Casa do Índio, Distrito Sanitário Especial Indígena de Manaus, Manaus, Brazil; 5 Secretaria Municipal de Educação, Prefeitura Municipal de Borba, Borba, Brazil; 6 Programa de Pós-Graduação em Ciências da Saúde, Universidade Federal do Amazonas, Manaus, Brazil; 7 Escola de Saúde Pública, Governo do Distrito Federal, Brasília, Brazil; 8 Núcleo de Escritores e Artistas Indígenas do Brasil, Rio de Janeiro, Brazil; 9 Centro Bioindustrial, Instituto Butantan, São Paulo, Brazil; 10 Duke Global Health Institute, Duke University, Durham, United States of America; UNESCO International Center for Biotechnology, NIGERIA

## Abstract

Snakebite envenoming is a major public health problem in the Amazon, disproportionately affecting Indigenous populations with high incidence and mortality rates. Efforts to decentralize antivenom treatment to remote areas require not only logistical adaptations, but also a deeper understanding of Indigenous medical systems to enable culturally appropriate care. This study aimed to construct an explanatory model of snakebites from the perspective of the Munduruku people, an Indigenous group in the Central Brazilian Amazon. We conducted a qualitative study based on in-depth interviews with nineteen traditional healers. Our methodological orientation follows the Amerindian perspectivism theory. Data was sorted into five relevant categories: 1) Participants’ identities; 2) Snakes and snakebites; 3) Course of sickness; and 4) Therapeutic resources in the Munduruku medicine. Munduruku healers interpret snakebites as events involving both natural and supernatural dimensions, integrating bodily, social, and spiritual factors. Snakes are perceived as intentional beings, and envenomation may result not only from physical encounters but also from sorcery or transgression of social norms; perceived severity is shaped by the type of snake, adherence to dietary and sexual restrictions, and spiritual causality. Therapeutic practices predominantly involve topical preparations, rituals, and symbolic interventions embedded within broader relational and cosmological frameworks. Despite these distinct explanatory models, most participants recognized the importance of biomedical care, particularly for severe cases, and did not oppose referral to hospital-based treatment, while Indigenous healing practices remain central throughout the therapeutic itinerary. Improving snakebite outcomes in the Amazon requires intercultural health strategies that integrate biomedical and Indigenous systems, with symmetrical partnerships with Indigenous healers being essential to ensure timely access to antivenom while respecting local knowledge and practices.

## Author summary

Snakebite envenoming is a serious health problem in the Amazon, especially among Indigenous peoples, who face high rates of illness and death. Expanding access to antivenom in remote areas is important, but it must also consider local ways of understanding and treating illness. The Munduruku are an Indigenous people of Tupi linguistic origin living in the central Brazilian Amazon, with a strong historical, territorial, and cultural presence in the Tapajós River basin. In this study, we explored how the Munduruku people in the Brazilian Amazon understand snakebites through interviews with 19 traditional healers. For the Munduruku, snakebites involve both physical and spiritual aspects. Snakes are seen as beings with intention, and envenoming may result not only from direct encounters but also from sorcery or breaking social rules. The severity of illness depends on the type of snake, respect for dietary and sexual restrictions, and spiritual causes. Treatments include plant-based remedies, rituals, and symbolic practices connected to their way of life. Healers also recognize the importance of hospital care, especially in severe cases. Improving snakebite care in the Amazon requires collaboration between Indigenous and biomedical systems, ensuring timely treatment while respecting local knowledge.

## Introduction

Classified as a neglected tropical disease by the World Health Organization, snakebite envenoming is estimated to cause 5.4 million cases annually, including 400,000 amputations or disabilities and 138,000 deaths per year, particularly in low- and middle-ncome countries and predominantly affecting rural populations [1]. Rural areas of Latin America, particularly the Brazilian Amazon, are among the regions at highest global risk for snakebites and their complications [2]. This scenario results from the overlap between the high diversity and abundance of medically important snakes and the limited access of snakebite patients to urban centers that provide antivenoms [3].

In the Brazilian Amazon, *Bothrops atrox* accounts for approximately 90% of snakebite envenomings [3]. Clinical presentations range from local pain and swelling to severe complications, including secondary infections, necrosis, and compartment syndrome [4–7]. Long-term sequelae include musculoskeletal and sensory deficits, as well as stroke-related disability [8–10]. Major causes of death include systemic bleeding, shock, sepsis, and acute respiratory failure [11,12]. The Amazon region shows the highest severity and mortality rates in Brazil [13], disproportionately affecting Indigenous populations, who have incidence rates 7.5–11 times higher and a 3.5-fold greater risk of death compared with the general population [14,15]. Reducing this burden is a core objective of Brazil’s Indigenous Health Care Subsystem (SASISUS), implemented under the National Policy for the Health Care of Indigenous Peoples (PNASPI), which mandates comprehensive and culturally differentiated care [16]. Within this framework, interventions must be culturally acceptable and integrated into local therapeutic itineraries [17]. National policies promote collaboration between biomedical teams and traditional healers, who play a central role in early care and decision-making [16].

In Brazil, health facilities that provide antivenom are unevenly distributed across the territory, creating significant access barriers, especially for Indigenous populations living in the Amazon [18]. In some Indigenous territories, lack of access to health facilities for snakebite patients exceeds 30% [19]. Understanding Indigenous cosmologies and medical systems is essential for implementing cross-cultural care strategies and reducing resistance to snakebite treatment [20,21]. A program to decentralize antivenom treatment to Indigenous communities in the Amazon has demonstrated potential to reduce morbidity and mortality from snakebites [22]. The maintenance and expansion of this program depend on adapting Indigenous community health centers to effectively provide antivenom treatment in loco [23,24], promoting culturally tailored training of health professionals [25,17], involving health workers of Indigenous ancestry [26], and engaging traditional caregivers to integrate Indigenous healthcare models with timely referral of snakebite patients to facilities equipped with antivenom [21].

### The Munduruku people

The Munduruku people (self-identified as Wuy jugu) have a longstanding warrior tradition and historically dominated the Tapajós Valley region, with the first documented contacts with Portuguese colonizers dating to the second half of the 18th century [27]. They belong to the Munduruku linguistic family of Tupi origin. Historically portrayed as a strongly warlike society, the Munduruku undertook extensive expeditions during which they collected mummified enemy heads as trophies, attributing to them symbolic and magical powers [28]. Today, the Munduruku inhabit territories across the states of Pará, Amazonas, and Mato Grosso, in the central and southern Brazilian Amazon, with an estimated population of approximately 18,000 people. Most communities are located within the Munduruku Indigenous Land, particularly along the Cururu River, a tributary of the Tapajós River. Although their territory was officially demarcated in 2001, the Munduruku continue to face ongoing threats to territorial integrity, including illegal gold mining, hydroelectric development, and infrastructure projects such as the proposed Tapajós waterway. A comprehensive overview of the historical and anthropological context of the Munduruku people is provided by the Socioenvironmental Institute [29].

Subsistence farming is practiced according to longstanding traditional knowledge, primarily on dry land, with intensive use of space and intercropping systems [30]. Bitter cassava is the main crop cultivated in the villages and is processed into flour, a staple food consumed in nearly every meal. Other commonly cultivated crops include sweet cassava, bananas, potatoes, yams, peppers, and a wide variety of fruits such as pineapple, watermelon, açaí, patauá, bacaba, pupunha, ingá, and Brazil nuts, as well as tobacco. Fishing, hunting, and gathering also play a central role in food acquisition, with fishing currently representing the primary source of animal protein [30]. The Munduruku produce cassava flour both for subsistence and for sale, and the commercialization of surplus production enables the purchase of essential goods such as salt, sugar, soap, clothing, footwear, and fuel [31].

Despite this subsistence system, poor health indicators are reported among Munduruku populations, including documented cases of mercury exposure and associated clinical outcomes [32,33], chronic non-communicable diseases [33–35], tuberculosis [36], hepatitis B, malaria, and respiratory infections [29]. Although snakebites represent a significant health concern among Indigenous peoples in the Amazon, knowledge and practices related to their management among the Munduruku remain largely unexplored. This study aims to elaborate an explanatory model of snakebites in the Munduruku cosmology and medicine, in the Central Brazilian Amazonia.

## Methods

### Ethics statement

The study was approved by the Research Ethics Committee of the Universidade do Estado do Amazonas (CAAE: 17408719.2.0000.5016). Data were collected through in-depth interviews conducted after participants had been informed about the study objectives and had provided written informed consent. All data were anonymized and securely stored under the responsibility of the research team, ensuring confidentiality.

### Study setting

**Fig 1:**
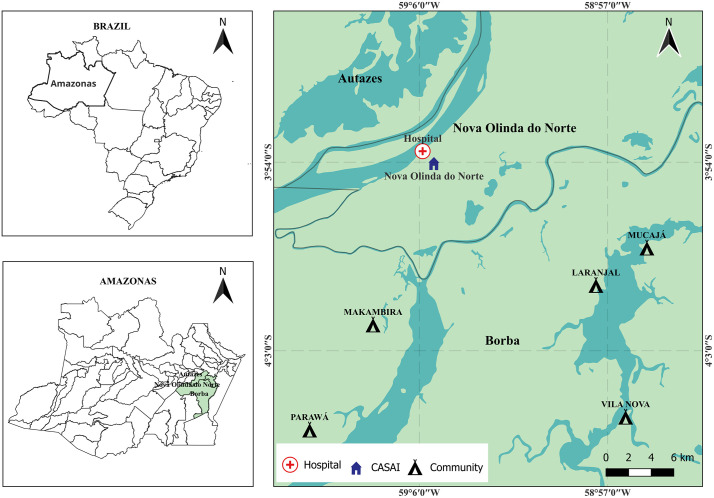
Location of the study area in the Manaus Special Indigenous Health District, municipalities of Nova Olinda do Norte and Borba, Amazonas state, central Brazilian Amazon. The map shows the distribution of Indigenous villages along the Canumã and Mari-Mari rivers. The Indigenous Health Support Center (Casa do Índio, CASAI) is part of the Indigenous Health Care Subsystem and provides accommodation, food, and nursing care for Indigenous patients and their companions referred for medium- and high-complexity care in reference municipalities. The map was produced by the authors using QGIS software and publicly available geographic data from the Brazilian Institute of Geography and Statistics (IBGE; *Instituto Brasileiro de Geografia e Estatística*), freely accessible under the Brazilian Access to Information Law (Law 12,527/2011) [37]. Instituto Brasileiro de Geografia e Estatística. Malha Municipal. 2025 [cited 3 Jan 2026]. Available: https://www.ibge.gov.br/geociencias/organizacao-do-territorio/malhas-territoriais/15774-malhas.html.

The study was conducted among Munduruku Indigenous populations living in the municipalities of Nova Olinda do Norte and Borba, in the state of Amazonas, in the central Brazilian Amazon. The study area comprises 34 villages located along the Canumã and Mari-Mari rivers, of which 33 are inhabited by Munduruku people and one by the Sateré-Mawé. Twenty-two villages are served by the Kwatá healthcare pole and twelve by the Laranjal healthcare pole (Fig 1). Both poles are administrative health units of the Manaus Special Indigenous Health District and are staffed by multidisciplinary teams, including physicians, nurses, dentists, nursing assistants, oral health technicians, Indigenous health agents, and boat operators.

At the beginning of the study (August 2023), these healthcare units did not provide antivenom treatment for snakebite envenoming and were limited to basic first aid, such as wound cleaning and analgesia. In August 2024, the Kwatá healthcare pole began providing antivenom treatment as part of a pilot decentralization program for Indigenous health services in the Brazilian Amazon [22]. Currently, mild and moderate cases of snakebite envenoming (SBE) are managed within the territory, while severe cases are referred to the hospital in Nova Olinda do Norte. The total population of the Kwatá and Laranjal Indigenous Lands is approximately 4,200 individuals.

### Study design

We conducted an explanatory qualitative study in a Munduruku Indigenous territory to understand the role of the folk healthcare sector, focusing on socially legitimized caregivers sought by community members in cases of snakebite. Data were collected between August 2023 and March 2025. The study was reported in accordance with the Consolidated Criteria for Reporting Qualitative Research (COREQ) guidelines (S1 File).

### Study participants

Munduruku Indigenous caregivers aged 18 years or older were invited to participate, following the typology described by Scopel et al. [38], which includes ‘bonesetters/contusion and rip takers’ (pegadores de desmentidura/rasgadura, in Portuguese), midwives, prayers and blessers, healers, ‘pajés’, ‘spiritists’, and ‘sacaca’ (Table 1). The nineteen participants were distributed across the villages of Vila Nova (n = 8), Mucajá (n = 5), and Laranjal (n = 2), in the Laranjal Indigenous Land, and Makambira (n = 3) and Parawá (n = 1), in the Kwatá Indigenous Land.

**Table 1:** Munduruku Indigenous caregivers’ typology and definitions.

Classification^1^	Profile
‘Bonesetters/Contusion and rip takers’ (*Pegadores de dismentidura e rasgadura*, in colloquial Portuguese)^2^	Specialists of both sexes, who work through physical manipulation of the patient’s body
Midwives (*Parteiras*, in Portuguese)	Women with traditional knowledge about pregnancy, childbirth and postpartum
Prayers and blessers (*Rezadores* and *benzedores*, in Portuguese)	Caregivers of both sexes who use faith and spiritual power as the tool of care
Healers (*Curadores*, in Portuguese), ‘*pajés*^3^*’* and ‘*spiritists*^4^*’* (*Espiritistas*, in Portuguese)^5^	Caregivers of both sexes, who use a variety of treatment techniques, which may differ within the same class, and be the same between different classes of caregivers
‘*Sacaca*’^4^	Spiritual specialists with connections to the invisible world and enchanted beings

^1^Based on the ethnographic studies from Scopel et al. [38].

^2^‘*Dismintidura*’ is a term that refers to the displacement of bones and joints, or muscle and ligament injuries. ‘*Rasgadura’* refers to muscle stretching.

^3^‘*Pajé’* is the term to denote a shaman among Brazilian indigenous people, and serves as a counselor, healer, sorcerer, and spiritual intermediary.

^4^‘*Spiritists*’ perform religious rituals, ceremonies and treatments that involve elements from Catholicism, Kardecism, religions of African origin (Umbanda and Candomblé), and Indigenous original practices.

^5^Healers, *pajés*, *spiritists* and the *sacaca* have the ‘*banqueiros*’ as their assistants.

None of the researchers had prior contact or established relationships with the participants.

Participants were identified through purposive sampling, with the support of local health and education professionals working in the Indigenous territories who were familiar with community-recognized caregivers. This approach enabled the inclusion of socially legitimized healing specialists across a range of therapeutic roles within the Munduruku healthcare system. Recruitment continued until thematic saturation was reached, defined as the point at which no substantially new themes emerged from the interviews.

### Methodological orientation

Studies on the social organization of healthcare identify three structural sectors: the professional sector (comprising organized healing professions within modern scientific medicine, such as physicians, nurses, and pharmacists); the popular sector (including family, social networks, and the community); and the folk sector (consisting of non-professional, non-bureaucratic specialists) [39]. In this study, we assume that, among Indigenous populations, the folk sector remains a central component of snakebite care [21]. We further consider shamanism to constitute a shared cultural foundation among Indigenous peoples of Central and South America [40,41]. Within this framework, healing represents a core function of shamans in culturally constructed clinical realities. In many societies, shamans are regarded as the primary agents capable of treating illnesses associated with sorcery, spirit attack, and soul loss [40].

Our methodological orientation also draws on ontological approaches within anthropology, with particular attention to Amerindian perspectivism, as described in ethnographic studies of Amazonian Indigenous peoples [42]. Within this framework, humans, animals, and other non-human beings are understood to share a common cultural or spiritual condition. Amerindian perspectivism emphasizes that different beings perceive reality according to the specificities of their bodies, while maintaining an underlying anthropomorphic spiritual essence. In this view, humanity is a reflexive condition of a species to itself, whereas animality corresponds to how a body is perceived from the perspective of another species: animals and other beings see themselves as human and experience their worlds accordingly [43].

### Data collection

Data were collected through in-depth interviews guided by a semi-structured script (Table 2). The script was developed by the research team, which has extensive experience in qualitative research and in the cultural and clinical aspects of snakebite envenoming in the Amazon, and was designed to align with the study’s objective of constructing an explanatory model of snakebites within Munduruku cosmology and medicine. Interviews were conducted in quiet and comfortable settings. At the beginning of each interview, the interviewer introduced themselves, provided a brief background, and explained the study objectives. Interviews lasted approximately 30 minutes, were audio-recorded, and complemented by field notes. All participants were fluent in Portuguese, and interviews were conducted in this language. Recordings were transcribed and de-identified prior to analysis. Care was taken to respect participants’ social roles within their communities, including their preferred forms of self-identification. The interviewer adopted a non-judgmental approach and avoided classifying the reported practices in terms of effectiveness.

**Table 2:** Interview script for Munduruku Indigenous caregivers.

Questions	Domain
	**Participant’s introduction**
1	Could you introduce yourself?
2	What type of caregiver do you consider yourself?
	**Snake cosmology and snakebite risk perception**
3	Tell me if there are any stories, legends, myths, tales about snakes among your people?
4	Have you ever had any experience with snakes in your village?
5	Do snakes have any particular meaning for you?
6	What types of snakes do you know?
	Do snakes have any utilization in your daily life?
7	Which snakes do you consider most dangerous?
	**Snakebite understanding and care**
8	Do you provide any care for people envenomated by snakes?
9	Could you tell me what care you provide?
10	Did you inherit this care from a family member or a specialist in your ethnic group?
11	When and for how long is this care performed?
12	Do you perform this care alone or in conjunction with medical care?
13	Did the care you provided result in any improvement in the snakebite?
14	Are the resources used in this practice easily available?
15	Have you ever used any part from the snake itself to perform this care?

Most interviews were conducted in the Indigenous villages. Three participants (two from Makambira and one from Parawá) were interviewed at the *Casa de Apoio à Saúde Indígena* (CASAI) in Nova Olinda do Norte, a facility that provides accommodation and support for Indigenous patients referred for medium- and high-complexity care. Field visits were conducted during the rainy season, when river navigation allowed access to the villages.

### Study team and reflexivity

The first author (GRD), a female researcher, conducted participant recruitment and interviews. She is a registered nurse of Indigenous ancestry from the same region as the study site, with formal training in qualitative research and a Master’s degree in Nursing.

The research team comprised eleven additional members with diverse academic backgrounds (five PhDs, three MScs, one MBA, and three BSc degrees), including registered nurses (GRD, TAG, MLS, JS, ASF), a pharmacist (GRDL), educators (GSM, VAM), a dentist (MCA), a social worker (GRDS), a physician (FHW), and an epidemiologist (WM). Given the cultural sensitivity of the topic, reflexivity was considered throughout the research process. The first author’s Indigenous ancestry and academic background positioned her close to the study context, which may have influenced interpretation. To address this, a rigorous reflexive approach ensured interpretations remained grounded in participants’ narratives. Analyst triangulation and team discussions were used to minimize bias and strengthen consistency. Ethnographic images were produced manually as paintings by one of the co-authors (UGO), an Indigenous artist from the study area.

### Data analysis

After data organization, interview transcripts were analyzed using a four-phase deductive content analysis approach [44]. In the first phase, two independent researchers (GRD and TAG) developed separate codebooks using ATLAS.ti software. Discrepancies between coders were systematically discussed and resolved through consensus meetings, with iterative refinement of the coding framework to ensure coherence, transparency, and analytical consistency across transcripts. The researchers then met in person to discuss discrepancies and agree on a unified coding framework. In the second phase, data were organized into four main categories: (1) participants’ identities; (2) snakes and snakebites, including causality and cultural meanings within Munduruku medicine; (3) course of illness, including symptom onset, perceived pathophysiology, and prognosis; and (4) therapeutic resources in Munduruku medicine [40]. In the third phase, patterns and relationships within and across categories were identified. Representative excerpts from participants’ narratives were selected to illustrate key findings. The fourth phase involved refining interpretations through iterative discussions among the research team to ensure analytical consistency and coherence. Data analysis was completed in April 2025.

## Results

### Participants’ identities

A total of nineteen specialists were included, comprising fourteen women and five men, with ages ranging from 28 to 93 years. Regarding religious affiliations, eleven participants identified as Catholic, three as Spiritist, two as Protestant, two reported participating simultaneously in both the Catholic Church and a Spiritist center, and one participant reported participation in both Catholic and Protestant churches.

Most participants were midwives (ten; 53%), and six of these also performed additional roles, such as *bonesetters/contusion and rip takers* (five), blessers (two), *pajé* (one), prayers (one), healer (one), and spiritist (one). The remaining nine participants performed roles as *pajé* (five), *bonesetters/contusion and rip takers* (three), spiritists (three), prayers (two), blessers (two), healers (two), and *sacaca* (one) (Table 3).

**Table 3:** Participants’ specialties and social role within the Munduruku medicine and cosmology.

Participant	Gender	Age (years)	Religious affiliation	Specialty	Social role
P1	Male	64	Catholic	*Pajé* and ‘*bonesetters/contusion and rip takers*’^1^	The specialist has four years of experience and reports treating snakebites using açaí palm and preparations derived from snake and pig tissues.
P2	Female	53	Catholic	Midwive	The specialist has 29 years of experience. She reports treating snakebites using snake-derived materials. She describes the use of snakeskin for medicinal purposes and fumigation with tobacco, and snake fat in practices associated with harm or malevolent intent.
P3	Female	93	Catholic	Midwive	The specialist had 76 years of experience but is no longer practicing. She reported treating snakebites using lemon (*Citrus × limon*), fennel (*Foeniculum vulgare*), pipe vine (*Aristolochia cymbifera*), and gunpowder.
P 4	Female	58	Catholic	Midwive	The specialist has 28 years of experience. She reports treating snakebites using Brazil nuts and preparations derived from snake parts applied to the bite site. She also uses snakeskin for fumigation and for the treatment of various illnesses.
P5	Female	70	Catholic and Spiritist	Midwive, *pajé* and healer	The specialist has 40 years of experience. She has worked as a midwife for four decades and has served as a *pajé* and healer since a young age. She reports treating snakebites using forest resources and the snake itself. She uses bushmaster fat for medicinal purposes and for treating spells.
P6	Female	44	Spiritist	*Pajé* and spiritist	The specialist has three years of experience. She has worked as a *pajé* and spiritist for three years, practicing the white, red, and black traditions. She maintains a healing space (*seara*) within her home, where she performs therapeutic practices. She describes the use of snake fat for treating spells.
P7	Male	28	Spiritist	*Pajé* and spiritist	The specialist has 16 years of experience. He has served as a *pajé* and spiritist since the age of 12. He reports working with Honorato, a river master and spiritual guide who is believed to transform into a snake. He describes treating snakebites using *Cuniculus paca* bile and employing snake parts in practices related to spells.
P8	Female	46	Spiritist	*Sacaca*	The specialist has 21 years of experience. She is the village’s primary *sacaca* and is regarded as a matriarch. Her practice follows a syncretic perspective, incorporating rites associated with Afro-Brazilian religions. She reports working with spiritual entities such as Tupinambá, Índio Guerreiro, Sete Flechas, Pena Verde, Pereira, Caboclo Flecheiro, Manecatú, and Rompe Mato. For the treatment of snakebites, she uses lemon juice, capeba juice (*Piper umbellatum*), cupuaçu juice (*Theobroma grandiflorum*), and wild pineapple (*Ananas comosus* var. *bracteatus*), in addition to using snakeskin in ritual practices.
P9	Male	86	Protestant	Prayer and blesser	The specialist has 40 years of experience. He is a *fishbone prayer* and *blesser*, recognized for removing fish bones lodged in the throat or alleviating related discomfort through healing practices. He reports treating snakebites using gunpowder and preparations made from snake liver and fat applied as a poultice to the wound. He also describes the use of snake oil in practices related to spells.
P10	Female	66	Catholic and Spiritist	Midwive and ‘*bonesetters/contusion and rip takers*’	The specialist has 30 years of experience. She reports treating snakebites using grated Brazil nuts and preparations derived from the snake’s head applied to the wound. She has also used snake parts to treat *desmentidura* and for fumigation with tobacco.
P11	Female	88	Catholic	Midwive and ‘*bonesetters/contusion and rip takers*’	The specialist has been practicing for 50 years and knows how to treat snakebites with garlic and a mixture of manioc flour and roasted lemon.
P12	Female	65	Catholic	Midwife, prayer, blesser, *spiritist* and ‘*bonesetters/contusion and rip takers*’	The specialist has 40 years of experience. She reports treating snakebites using *palheira* leaves and fat from tegu lizards (*Tupinambis* spp.), paca (*Cuniculus paca*), and capybara (*Hydrochoerus hydrochaeris*). She also performs prayers for snakebite patients in hospital settings. In addition, she uses snake parts to treat various ailments and snakeskin for fumigation and practices related to healing spells.
P13	Female	42	Catholic	Healer and ‘*bonesetters/contusion and rip takers*’	The specialist has 30 years of experience. She describes dietary restrictions and guidelines for the management of snakebites. She is knowledgeable about the use of snake fat and snake parts for fumigation.
P14	Female	74	Catholic	Midwive, blesser and ‘*bonesetters/contusion and rip takers*’	The specialist has 40 years of experience. She reports treating snakebites using *paricá* tea (*Schizolobium amazonicum*) and cupuaçu juice (*Theobroma grandiflorum*), combined with *Saint George’s sword* (*Dracaena trifasciata*) and applied as a poultice. She also uses *tracuá* vine (*Philodendron* spp.), dolphin fat, copaíba oil (*Copaifera langsdorffii*), and ritual blessings intended to remove snake venom. In addition, she employs snake parts and fumigation in her practice.
P15	Female	30	Catholic	*Pajé* and healer	The specialist has 16 years of experience. She reports treating snakebites using the juice and plant parts of *julica* (a locally known plant with unidentified botanical classification). She also recognizes the use of snake fat for treating stingray injuries and snakebites. In addition, she describes the use of snake fat and snake heads in practices intended to reverse spells.
P16	Female	58	Protestant	Midwive	The specialist has 20 years of experience and compares snakebite care to postpartum care, including a 40-day restriction on fatty foods. She describes her knowledge of using snake parts for wound healing, rheumatism, and practices related to spells.
P17	Male	42	Catholic	*Pajé*	The specialist has 14 years of experience. He reports treating snakebites using shavings of cupuaçu fruit peel (*Theobroma grandiflorum*) and fat from tegu lizards (*Tupinambis* spp.). He also applies snake parts directly to the bite site and recognizes the use of bushmaster skin in practices related to spells.
P18	Female	55	Catholic and Protestant	Midwive and ‘*bonesetters/contusion and rip takers*’	The specialist has 21 years of experience. She reports treating snakebites using Brazil nuts, paca bile (*Cuniculus paca*), bellyache bush juice (*Jatropha gossypiifolia*), cupuí juice (*Theobroma subincanum*), and fat from dolphins and *Boa constrictor*. She also uses snake parts combined with chili peppers (*Capsicum frutescens*; *pimenta malagueta*, in Portuguese) for fumigating the house and for practices related to healing spells.
P19	Male	58	Catholic	Spiritist, prayer, blesser and ‘*bonesetters/contusion and rip takers*’	The specialist has 20 years of experience. He reports treating snakebites using grated avocado pits, snake parts, and snake fat applied to the bite site.

^1^‘*Pegadores de desmentidura/rasgadura’*, in Portuguese.

Midwives represented a large proportion of participants, reflecting their central and multifunctional role in Munduruku healthcare.

### Thematic synthesis of findings

The Munduruku interpret snakebites within an explanatory model that integrates the body, environment, social relations, and spiritual dimensions. Snakebite is not understood merely as a natural event, but as an experience involving both natural and supernatural forces, requiring forms of care that combine material practices and ritual elements.

### Snakes and snakebites

Although Portuguese is the predominant language spoken by the Munduruku, terms from their native language are still used in specific contexts, such as in the naming of snakes. The words used by participants to refer to snakes were *poibute*, *poibu*, and *piabu*. Despite minor phonetic variations, these terms reflect the symbolic continuity of this concept within the Munduruku lexicon.

Snakes are recognized by Munduruku healers based on their physical characteristics and behavior. The species referred to as *surucucu pico-de-jaca* (or *pico de jaca*) is described as less aggressive toward humans but responsible for the most severe envenomations. Based on reported characteristics such as color and size, this Indigenous taxonomic category may correspond to *Lachesis muta* (bushmaster) or *Bothrops atrox* (Amazonian lancehead). However, other morphologically similar snakes, including species considered non-venomous, such as *Helicops angulatus* and *Xenodon* spp., may also be included in this category. The terms *jararaca*, *surucucu*, and *surucucurana* are generally associated with snakes perceived as more aggressive toward humans but causing less severe envenomations. In practice, the identification of the snake responsible for a bite is often established a posteriori, based on the clinical progression of the patient, particularly the perceived severity of the condition. Munduruku healers also report sightings of a snake referred to as the *parrot snake* (*cobra papagaio* or *papagaia*, in Portuguese), described as green, arboreal, and highly aggressive. This description may correspond to *Bothrops bilineatus*, a species widely distributed in the Amazon but not yet formally reported in Munduruku territory [45]. Coral snakes are considered less common in the region and are recognized by their bright coloration and discreet behavior. The *sucuriju*, also known as *sucuri*, is associated with aquatic environments and likely corresponds to *Eunectes murinus* (anaconda). Rather than biting humans, it is described as preying on animals through constriction, including dogs, chickens, and ducks. Healers also reported other animals classified as snakes, including some considered venomous; however, these accounts were imprecise and inconsistent.

Snakes inhabit a wide range of environments, including forests, pastures, deforested areas, plantations, trails, wetlands, and peridomestic settings, reflecting their constant coexistence with Indigenous communities. As a result, encounters between humans and snakes are embedded in daily activities such as work, subsistence practices, and community life. Indigenous healers describe snakes as beings endowed with intentionality and capable of interacting with the spiritual realm. They are characterized as cunning, fearless, agile, and dangerous, capable of responding to perceived threats from other living beings. These attributes evoke both fear and respect among humans. While snakes and humans may avoid direct contact, encounters can nonetheless result in attacks in either direction: from snake to human, causing envenomation, or from human to snake, resulting in injury or death. Some encounters are intentional, as Indigenous people actively seek to eliminate snakes in areas surrounding villages, including cultivated fields. This practice aims to reduce the risk of snakebites and to obtain animal tissues for medicinal, ornamental, or ritual purposes. However, snakebites are also frequently associated with unintentional encounters, often attributed to momentary inattention, reflecting the shared occupation of space by humans and snakes.

**Fig 2:**
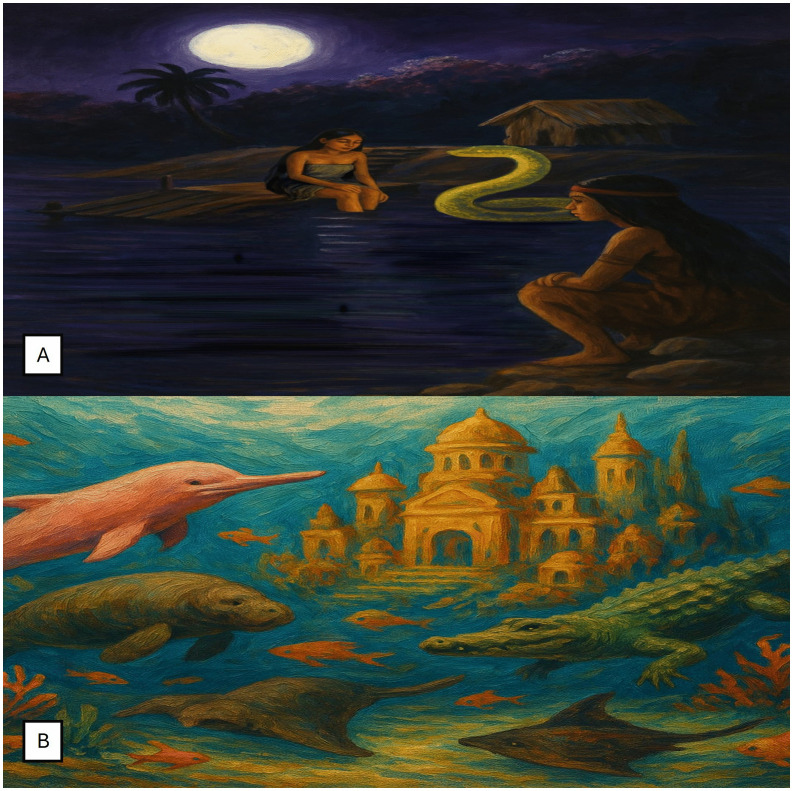
Ethnographic representation of Munduruku healers’ accounts of the enchantment of women by snakes inhabiting the *‘Underwater World’.* **A)** Narrative describing the disappearance of a young woman following episodes of illness and her subsequent immersion in a river, interpreted by the Munduruku as a spiritual transition to an enchanted underwater city: ‘*She went down to the riverbank and invited her younger sister to accompany her. She told her she was going to bathe and would return shortly to wash her. However, when she entered the water, they saw only a whirlpool; she was already gone, carried away by the snake*’ (Participant 6, *pajé* and *spiritist*). **B)** Representation of the *‘Underwater World’*, described as a city located at the bottom of rivers and lakes, inhabited by ancestral beings or spirits. These entities are understood to have agency over life on Earth and to influence daily human activities. Munduruku cosmology is characterized by processes of transformation, in which beings may shift between human, plant, animal, and spiritual forms, inhabiting and acting across different ontological planes, including the underwater domain.

According to the Munduruku, snakebites may also arise from causes that are not explained within scientific rationality, such as witchcraft. In this context, the snake is understood as an instrument used by a sorcerer to attack an enemy or to defend against an aggressor, who may be another human or a forest spirit. Another causal domain relates to snakebites interpreted as punishment for the transgression of customary norms. For example, menstruation is described as provoking the anger of snakes, as well as other forest beings. In Munduruku cosmology, prohibitions and taboos are closely associated with women’s bodies, particularly during menstruation and pregnancy. Women who do not observe practices such as menstrual seclusion are considered vulnerable not only to snakebites but also to other forms of misfortune. Participants 4, 7, and 18 reported the possibility of snakes impregnating women during this period, particularly if the snake comes into contact with their clothing. The reported signs and symptoms of such pregnancy include loss of appetite, marked weight loss, general malaise, and persistent pain lasting until childbirth, when the woman would give birth to a snake. This event is described as culminating in the enchantment of the mother, who would then be taken to live with the snake in the underwater world. Participant 6 added that, according to the knowledge of her community, menstruating women are subject to restrictions on accessing rivers at certain times, especially at midday and twilight. Failure to comply with these norms may result in the woman being enchanted by the snake and taken to the ‘*Underwater World*’ (Fig 2).

On the other hand, snake body parts, particularly fat and skin, are used to confer protection or fortune upon their possessor and are also employed as medicinal resources. Participant 2 reported that a fumigation ritual using snakeskin helps prevent and treat illnesses, including snakebites. Preparations made from snake parts are also believed to repel snakes and are applied to both adults and children who travel through forested areas. Participants 2 and 14 described the use of bracelets made from snakeskin to prevent *quebranto*, a condition attributed to malevolent influences such as witchcraft, envy, or the evil eye, which manifests in children as prostration, apathy, physical exhaustion, weakness, diarrhea, and vomiting. Participant 8 described the potential benefits attributed to snakes: ‘*We catch a snake, remove its head, and place it in a jar with alcohol so we can keep it in our house, so that we do not lack money or food. The snake is very treacherous; it betrays others to bring benefits to us*.’ Participant 6 reported a premonitory dream in which she perceived that she might be bitten by a snake at the behest of an enemy. She therefore sought the assistance of a healer in her village to reverse the spell. On another occasion, during a river journey, she described being suffocated by *Cabocla Janaína* - a snake-shaped spiritual entity - as a warning to interrupt the trip, as a storm was approaching.

### Course of sickness

**Fig 3:**
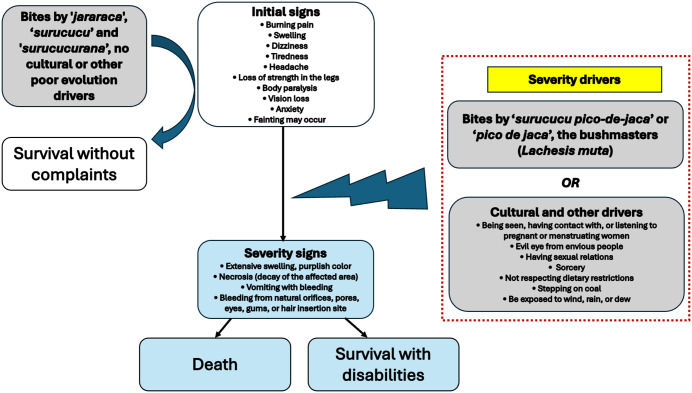
Possible clinical courses of snakebite according to Munduruku medicine. Severity is understood to be influenced by both the type of snake involved and cultural determinants, particularly adherence to social norms, as well as sexual and dietary restrictions.

The course of illness is understood to depend on both the causative agent and adherence to social norms, including sexual and dietary restrictions (Fig 3). The species referred to as *surucucu pico-de-jaca* (or *pico de jaca*) is associated with the most severe envenomations, potentially leading to physical disability, amputation, and death. In contrast, bites attributed to *jararaca*, *surucucu*, and *surucucurana* are generally described as causing milder clinical outcomes. Cultural determinants include, in particular, contact with pregnant or menstruating women, the *evil eye* from envious individuals, sexual activity, sorcery, and failure to observe dietary restrictions. These interpretations, transmitted across generations, shape behavioral norms from the moment of the bite through to recovery.

Dietary restrictions include the avoidance of foods classified as *remosos*, a category referring to items considered difficult to digest or high in fat. These include pork and game meats such as paca (*Cuniculus paca*), deer (*Mazama americana*), capuchin monkey (*Sapajus* spp.), and tapir (*Tapirus terrestris*). Fish with elongated bodies, smooth skin, or morphological features such as stingers or sharp teeth are also avoided, including piranha (subfamily *Serrasalmidae*), matrinxã (*Brycon amazonicus*), branquinha (*Potamorhina altamazonica*), jatuarana (*Brycon falcatus*), and surubim (*Pseudoplatystoma fasciatum*).

### Therapeutic resources in the Munduruku medicine

Table 3 presents participants’ characteristics and the roles through which caregivers identify themselves within their communities. Munduruku medicine encompasses a range of therapeutic resources for managing snakebites. However, healers report that, with the organization of patient transportation to hospitals within the Indigenous health system, their role has increasingly shifted toward providing initial, temporary care prior to biomedical treatment. Participant 16 noted that, currently, ‘*anyone bitten by a snake goes to the hospital. Before, no, we would take them home, keep them at home, and treat them with home remedies*.’ Similarly, Participant 17 stated, ‘*Sometimes home treatment is done first until they reach the city, the hospital. The first remedy is the home remedy, given when there is no other medicine.*’ Participants emphasized that, in the past, when transportation to hospitals was unavailable, Munduruku healers were the primary source of care. Most participants recognized the importance of hospital-based treatment, particularly for severe cases, and did not express resistance to referral to biomedical services.

Healers describe the effectiveness of their therapeutic practices based on successful cases, particularly from earlier periods when patients were treated exclusively within Munduruku medicine and experienced relief from symptoms or recovery without lasting disabilities. These perceptions of effectiveness are grounded in experiential knowledge, including empirical observation and, in some cases, informal animal experimentation, as reported by Participants 4 and 18, who described treating dogs bitten by snakes.

**Fig 4:**
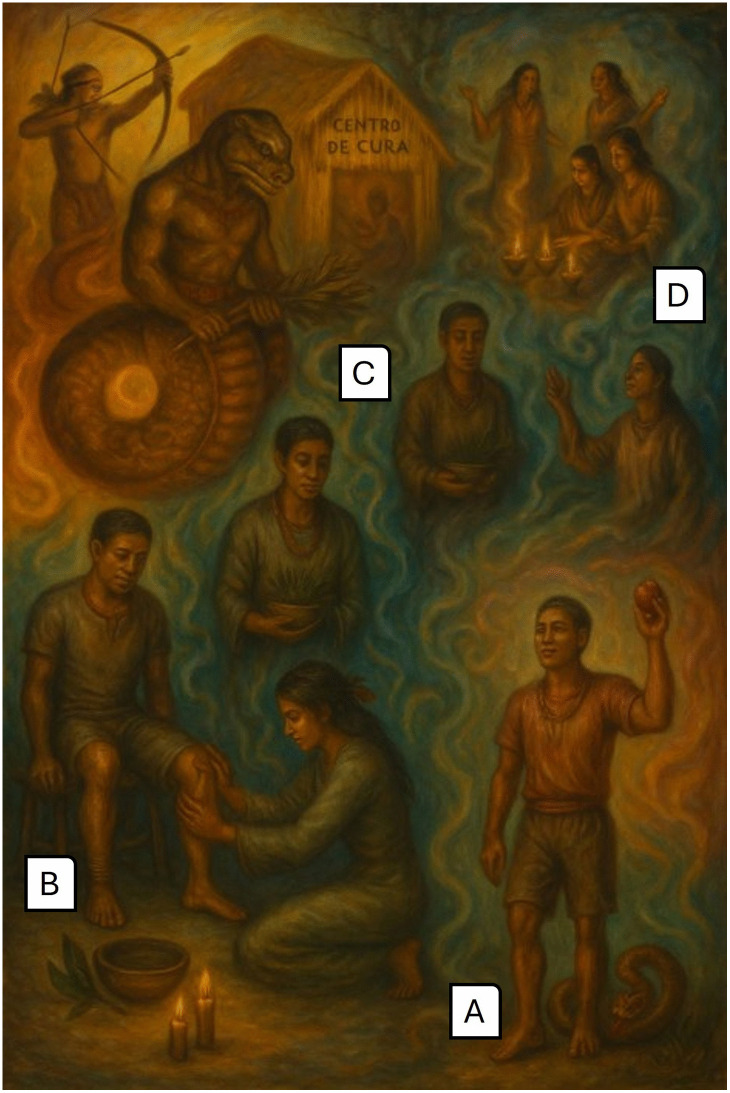
Ethnographic illustration representing healing rituals in Munduruku medicine for the treatment of snakebites. **A)** Snakebites are commonly reported to occur in areas of extractive activities or along the daily routes of Indigenous people within the village. **B)** Pain relief is described as a central therapeutic practice. Healers report employing this approach when individuals experience acute suffering, with the aim of alleviating symptoms. Concurrently, fumigation using elements of local fauna, such as hairs from capuchin monkeys and paca, is performed. **C)** Ritual practices include singing, dancing, fumigation, and collective enactment. Singing serves as a means of connecting with entities from the forest and aquatic domains, including enchanted beings such as spiritual snakes. **D)** Spiritual mediation involves the presence of forest and underwater entities, such as *Mané Catu, Índio Flecheiro (‘Indian Archer’), Pena Verde (‘Green Feather’),* and *Honorato*. These entities are invoked during ceremonies and are described as communicating through psychophony, using the *sacaca* and other healers as intermediaries, often in a language not fully understood by participants.

**Fig 5:**
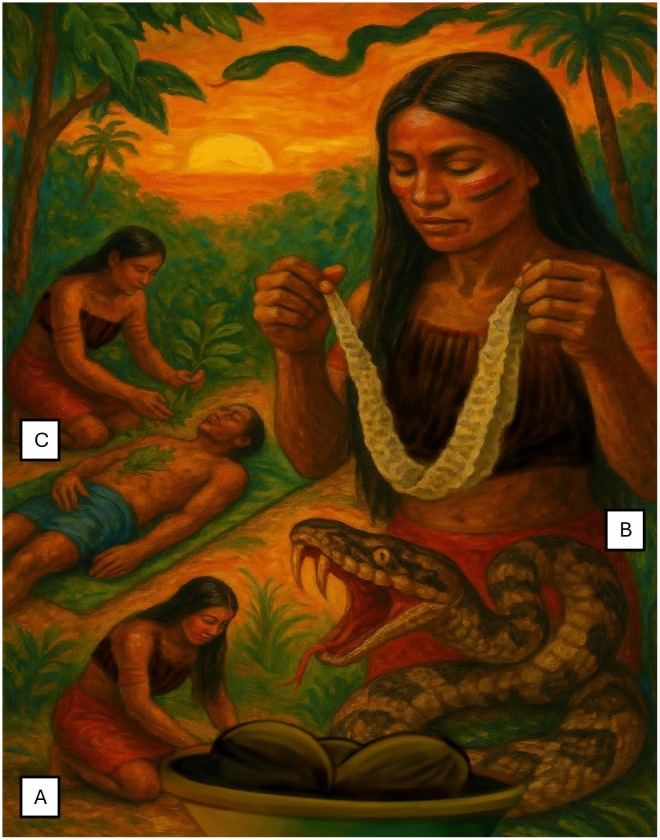
Ethnographic illustrations representing therapeutic resources in Munduruku medicine used to treat snakebites. **A)** The use of Brazil nut (*Bertholletia excelsa*) has been reported for both oral and topical applications in the treatment of snakebites. Topical application involves heating the nuts or extracting their sap and applying it directly to the affected area, which is then covered with a cloth. **B)** Another therapeutic resource involves the use of snake parts. After the snake is killed, its head is decapitated and buried as a symbolic act of containing harmful forces. The animal’s organs are cut into small pieces and mixed with flour and cassava to form a porridge, which is administered orally to both humans and non-human animals, such as dogs. **C)** Plants and snake meat are tied to the affected area to alleviate pain and to ‘neutralize’ the venom. These healing elements may also be applied to other parts of the patient’s body.

Each healer maintains a distinct therapeutic repertoire, shaped by family inheritance, learning from other healers, and personal experimentation. Reported practices include prayers and blessings, as well as the use of diverse preparations derived from plants and animals. Some of these preparations are applied topically, such as poultices made from fat, meat, and animal viscera, particularly from snakes. Participant 10 prescribes the topical application of snake brain, secured with a cloth over the fang marks. Participant 15 indicates the use of *Boa constrictor* fat for treating snakebites and for massage until full recovery. Participant 17, in turn, recommends applying the snake’s tail directly to the wound. Snakeskin is typically dried and used in fumigation rituals, in which it is burned and the resulting smoke is directed over the patient. Plant-based preparations are also widely used. Juices obtained by crushing leaves may be administered either topically or orally, while infusions made from leaves, bark, and roots are commonly taken orally. Participant 13 recommends the oral use of juice prepared from the ‘crushing of 12 leaves’ of bellyache bush (*Jatropha gossypiifolia*). Participant 14 prescribes the oral use of *paricá* tea (Brazilian firetree, *Schizolobium amazonicum*), along with a poultice made from cupuaçu juice (*Theobroma grandiflorum*) combined with *Saint George’s sword* leaves (*Dracaena trifasciata*). Figs 4 and 5 present ethnographic illustrations depicting healing rituals and other therapeutic practices reported by the participants.

**Fig 6:**
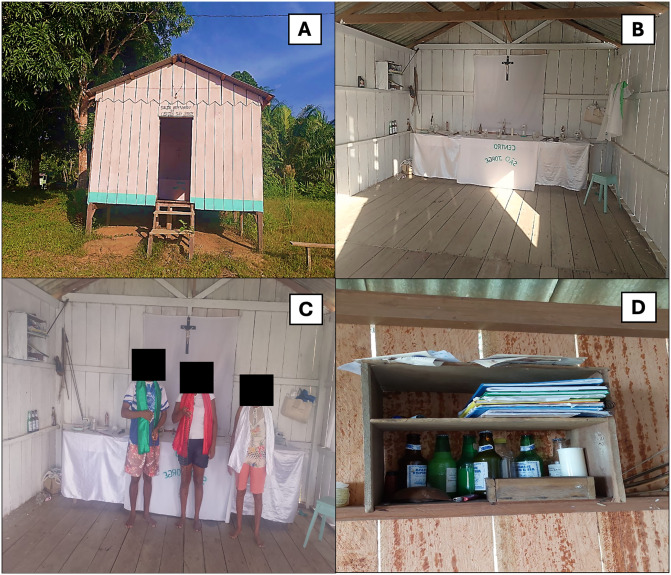
Characteristics of a Munduruku medicine *center*, located in Vila Nova village, Urariá River, municipality of Borba, Laranjal Indigenous Land, Amazonas state. **A)** Services are provided in a rectangular wooden structure with a single room, a raised floor, and a metal-tile roof. **B)** The interior space is organized to accommodate collective rituals and the circulation of participants. At the center stands a table, locally referred to as *bancada*, a key element in the spiritual and therapeutic practices carried out in the *center*. The table is covered with white fabric and arranged with containers of water, candles, tobacco, and other religious, spiritual, and Indigenous artifacts. **C)** Two *pajés* and one *sacaca* work in the *center*, wearing colorful garments, necklaces, and religious objects such as rosaries. **D)** Activities conducted in the *center* are systematically recorded in logbooks, which document spiritual services, and the prescriptions made by the *sacaca*.

Patient care may take place in different settings. Some healers maintain their own *centers* (Fig 6), which may be shared with other practitioners, while others provide care in their own homes. In some cases, healers also conduct home visits to perform rituals and treatments involving patients, their families, and the domestic environment.

Some participants reported that doctors and nurses do not value Indigenous medicine, often questioning its effectiveness and safety.

‘*The nurses say that you can’t use home remedies because it will interfere with their work*’ (Participant 1).

‘*Because sometimes even the doctors hardly trust us*’ (Participant 2).

Despite these perceptions, no explicit resistance among Munduruku healers was observed regarding patients seeking care at Indigenous health centers or hospitals outside the villages. On the contrary, there appears to be a shared understanding that certain cases should be referred to hospitals to receive antivenom treatment.

*‘If she (a spiritual guide) says that a patient is not for me, then I tell them to see a doctor, because it is not within my domain. So, the person must seek medical care’ (Participant 2).*

*‘I advised that he (a severe case) should first go to the city, to the doctors, so they could do their work. There, they have the injection against snake venom. When he returns to the village, once he is better, then I will take care of him’ (Participant 7).*

The return of the patient to the village after hospital treatment marks the resumption of Indigenous healthcare practices. This movement highlights the continuity of Munduruku medicine in guiding the healing process, within a complementary dynamic in which hospital care plays an important role, while recovery and the social validation of care are consolidated in the village.

‘*When he returned, I took care of him. For eight days I continued taking care of him*’ (Participant 15).

‘*They took him to Nova Olinda just to receive an injection, but on the same day he returned to the village, and it was here that he recovered, using home remedies that we taught him how to prepare*’ (Participant 18).

## Discussion

The cultural specificities of the Munduruku people shape a particular understanding of the health-disease process, integrating body, territory, social relations, and spiritual dimensions [31,38]. Our findings show that snakebite care is provided through networks of Indigenous healers who mobilize therapeutic resources grounded in shamanism, alongside practices derived from Christianity, Umbanda, Kardecist Spiritism, and the biomedical sector. This pluralistic system reflects ongoing processes of incorporation and reinterpretation rather than a fixed hybrid model, in agreement with previous ethnographic studies [46].

Since the 19th century, intensified contact with non-Indigenous populations and missionary activity has reshaped Munduruku social organization and belief systems [28,47]. The later introduction of Protestantism and Afro-Brazilian religions, particularly Umbanda, further contributed to this process [46,48]. However, rather than replacing Indigenous cosmology, these influences have been selectively incorporated. Umbanda, for instance, articulates ritual practices with Amazonian cosmology and maintains relationships with Indigenous spiritual entities [49]. Our findings indicate that different religious systems coexist without displacing the shamanic framework, which remains central to the interpretation and management of illness. This supports previous observations that Indigenous belief systems are continuously reinterpreted through contact with external influences [50–52].

Our results show that the coexistence of Christianity, Umbanda, and Spiritism does not imply antagonism with Munduruku shamanism. Rather, healers move between religious traditions while maintaining a shamanic cosmology. In this view, the cosmos is populated by potentially threatening beings, requiring constant action to ensure collective well-being [38]. Relationships between humans and other beings depend on practices such as healing and funeral rituals, dietary and sexual taboos, and the use of territorial resources, shaping social ties and bodily conditions [31,38]. Although external religions influence Indigenous belief systems, processes of resistance and reinterpretation allow their incorporation without erasing core elements [50]. Conversion is thus not stable hybridity but an ongoing, reversible transformation [51], a pattern that also appears in Indigenous engagement with biomedical and external religious practices, historically noted by missionaries [52].

Another key aspect is the overlap of social roles among Munduruku healers. Shamans and midwives may also act as spiritists or bonesetters. These roles are understood as innate ‘gifts’ that manifest throughout life [53], sometimes accumulating in a single individual. Beyond this intrinsic capacity, healers emphasize empirical validation based on observed recovery, from pain relief to rapid healing, reinforcing the legitimacy of their practices. Some accounts even describe animal experimentation, where treatments were recommended to humans after successful use in dogs bitten by snakes.

Positions of authority, such as shamanism, are often associated with men, reflecting both bodily assumptions and gendered knowledge systems [54]. However, our findings show that women play a central role in healing among the Munduruku. In contrast to research among the Tikuna [21], female healers predominated in this study. This highlights the need to incorporate women, especially midwives, into strategies for snakebite prevention and care. Given their extensive reach beyond pregnancy and childbirth [53,55], midwives are essential for effective treatment.

Although some Munduruku nomenclature for snakes persists, there are clear signs of linguistic loss. Terms related to color, size, and behavior, still known by elders, are being replaced by Portuguese among younger generations, threatening knowledge of species identification, venom characteristics, and symbolic meanings [56]. As reported for other Indigenous groups [21], Munduruku snake taxonomy is based on visual traits, habitat, behavior, and perceived severity of envenomation. Thus, the same species may be classified differently depending on context; for example, *Bothrops atrox* or *Lachesis muta* may both be identified as ‘surucucu’ when associated with severe symptoms.

This study adopts the premise that snakes, like other non-human beings, share a cultural and psychological background with humans [42,43]. In Amerindian perspectivism, all beings possess an anthropomorphic spiritual essence, differing in their bodily perspectives of reality. Myths describe an original shared human condition later transformed into diverse species [43]. Shamanism reveals that this condition persists, as humans, animals, and spirits retain an inner human essence [42,43].

Among the Munduruku, snakebites may have natural or intentional causes. In the first case, following Lévi-Strauss [57], they reflect a probabilistic model in which incidence depends on the abundance of humans and snakes and their shared environments. At another level, they follow a mechanical model based on individual relationships and a conscious process of otherness, shaped by the affective value of the snake-human interaction. This may range from situational triggers, such as a menstruating woman entering a snake’s habitat, to intentional aggression, including cases involving sorcery.

These mechanical explanations reflect a social world of intentional beings, including humans, snakes, and spiritual mediators. Between these models, intermediate interpretations exist, where snakebites may result from violations of social norms. Similar patterns are observed among other Indigenous groups: among the Tikuna, lying or disrespecting elders increases vulnerability to snakebites [21], while among the Achuar, retaliation by the Lords of the Animals may occur after excessive hunting [58].

Amazonian ethnographies describe snakes as both dangerous and essential for human survival, as mythical beings that generate or sustain fish stocks [59,60]. Consistently, Munduruku healers frequently use snakeskin and body parts in medicines, amulets, and ornaments, aiming to incorporate qualities attributed to snakes, such as power, protection, wisdom, and fortune.

Our findings also reveal a strong association between snakes and women in Munduruku cosmology, expressed in taboos related to menstruation and pregnancy, as well as myths of seduction and impregnation. In these narratives, snakes often assume human form to seduce women and take them to the Underwater World. The motif of the ‘snake’s wife’ symbolizes the union between human and non-human worlds and is often linked to the origin of sacred medicines or plants [61]. Lévi-Strauss [61] presents a Munduruku myth of the ‘*wife of the snake*’: The woman went to the forest every day under the pretext of picking rowan fruit, but in reality her intention was to find the snake. They had sexual relations and, when the time came to say goodbye, the snake dropped enough fruit to fill the woman’s basket. The woman became pregnant. One day the woman’s brother followed her, and discovering the situation, killed the snake. Later, the woman’s son with the snake would avenge his father.

In this study, poor prognosis in snakebite cases is associated with the type of snake, the perceived cause (natural or sorcery), and adherence to dietary, sexual, and behavioral restrictions. Amazonian Indigenous diets include numerous taboos, especially for the sick and their families. Among these, the avoidance of ‘*reimoso*’ foods, items believed to aggravate inflammation or blood conditions, is particularly important [62]. These include pork, shellfish, certain fish, birds such as ducks, and some game meats, which are avoided in situations involving wounds, infections, or recovery due to their perceived potential to worsen symptoms [63,64]. Consistent with findings among the Tikuna and Baniwa [21,65], snakebite treatment is always accompanied by restrictive diets. Patients must avoid fish resembling snakes or considered harmful (e.g., smooth, elongated, fatty, or spiny species), believed to weaken the body and exacerbate bleeding or illness [21,65]. Similar logic underlies the prohibition of contact with menstruating or pregnant women, as associated blood or bodily fluids are thought to disrupt bodily balance and worsen the patient’s condition [60,65].

In the Munduruku therapeutic system, topical treatments predominate, applied to the bite site to expel venom. These include poultices made from animal fat, skin, and tissues, especially from snakes, while plant-based preparations are less common. This suggests that the body surface is considered a key pathway for introducing active substances. In Indigenous conceptions, the body is formed through continuous exchanges with the environment and other beings, which underpins the perceived efficacy of topical, smoked, and aromatic medicines, as also observed among the Tikuna [21]. Among the Wari, for example, fragrant acidic honey is applied topically to expel venom through its aroma [66]. Although some medicines reported by participants have been investigated experimentally, their efficacy and safety in snakebite treatment remain unproven. Snakes possess endogenous toxin inhibitors, such as metalloproteinase and phospholipase inhibitors [67], and some plant metabolites have demonstrated inhibitory activity against venom components under laboratory conditions [68,69]. However, these findings are largely derived from experimental studies and should not be interpreted as evidence supporting the clinical use of traditional treatments for snakebites.

During this study, the Kwatá pole began providing antivenom treatment in the Indigenous territory [22]. Munduruku healers showed no resistance, commonly referring to it as the ‘*snakebite injection*.’ Antivenom was thus incorporated as a life-saving tool alongside Indigenous medical practices. This aligns with a broader preference for external therapies, as Amazonian Indigenous populations often regard injectable treatments as more effective than oral medications [66].

Our findings show that Munduruku healers already act as mediators between systems of care, identifying when referral is necessary while maintaining their role in healing practices. This highlights the potential of collaborative, rather than substitutive, intercultural strategies. Initiatives such as the decentralization of antivenom, culturally tailored training, and the inclusion of traditional healers can improve timely access to treatment while respecting Indigenous knowledge systems. However, challenges remain, including the predominance of biomedicine, limited institutional recognition of traditional specialists, and structural constraints such as inadequate sanitation and environmental protection. Strengthening more symmetrical partnerships between biomedical and Indigenous systems is therefore essential to improve outcomes in snakebite envenoming in the Amazon.

This study has limitations that should be acknowledged. Although the sample included diverse caregivers from multiple villages, it does not capture the full heterogeneity of the Munduruku population across territories. Regional and sociocultural differences may shape cosmological interpretations and therapeutic practices; thus, findings are not generalizable but analytically transferable, offering a contextually grounded model for intercultural health strategies. The semi-structured format allowed participants to expand beyond guiding questions, reducing framing effects. However, some questions may have suggested expected responses, particularly regarding treatment effectiveness. Despite the flexible and open-ended approach, response bias, especially social desirability bias, cannot be excluded, given participants’ roles as recognized healers within their communities.

## Conclusion

In this study, Munduruku medicine is not understood as a stable hybrid system, but as a dynamic process of incorporating and reworking practices from external religious traditions and biomedicine, guided by ongoing experimentation and the pursuit of therapeutic efficacy. Despite profound social transformations, this system remains anchored in a shamanic cosmology in which snakes share a cultural and psychological background with humans and may act intentionally, including in contexts of anger, envy, or sorcery. Within this framework, healers play a central role in diagnosing the etiology of snakebites and guiding treatment, including the management of spiritual dimensions of illness. Perceived severity is shaped not only by the biological characteristics of the snake, but also by moral, relational, and behavioral factors, such as adherence to dietary and social restrictions.

Importantly, participants reported no resistance to combining Indigenous and biomedical practices, particularly the use of antivenom, which is incorporated into local therapeutic itineraries. This highlights the capacity of Munduruku healers to act as mediators between knowledge systems, negotiating care across ontological frameworks. These findings underscore the potential for collaborative, intercultural health strategies that move beyond simple integration toward more symmetrical and dialogical partnerships. However, the effectiveness of such initiatives depends on their ability to engage with local epistemologies and practices, rather than subordinating them to biomedical logics. Strengthening Indigenous participation in health governance and ensuring culturally grounded implementation of interventions are therefore essential for improving outcomes in snakebite envenoming in the Amazon.

## Supporting information

S1 File Consolidated criteria for reporting qualitative studies (COREQ). (DOCX)

S2 File Anonymised database of interviews’ transcripts. (DOCX)
