# Supplementary material for: Snakes and snakebites in the munduruku cosmology and medicine, central Brazilian Amazonia
Source: PLoS Negl Trop Dis. 2026 Jun 22;20(6):e0014462. doi: 10.1371/journal.pntd.0014462 (PMC13309027; doi:10.1371/journal.pntd.0014462)
Supplement: S1 File — (DOCX) [file pntd.0014462.s001.docx]

**Consolidated criteria for reporting qualitative studies (COREQ): 32-item checklist**

**Snakes and Snakebites in the Munduruku Cosmology and Medicine, Central Brazilian Amazonia**

*Developed from:*

*Tong A, Sainsbury P, Craig J. Consolidated criteria for reporting qualitative research (COREQ): a 32-item checklist for interviews and focus groups. International Journal for Quality in Health Care. 2007. Volume 19, Number 6: pp. 349 – 357*

| **No. Item** | **Guide questions/description** | **Reported on Page** |
| --- | --- | --- |
| **Domain 1: Research team and reﬂexivity** |  |  |
| *Personal Characteristics* |  |  |
| 1. Inter viewer/facilitator | *Which author(s) conducted the interview or focus group?*  A researcher of G.R.D indigenous descent. | Page 11 |
| 2. Credentials | *What were the credentials of the researchers?*  Students, Masters, Doctors. | Page. 11 |
| 3. Occupation | *What was their occupation at the time of the study?*  Master's and doctoral students, and PhDs with expertise in qualitative health research. | Page 11 |
| 4. Gender | *Was the researcher male or female?*  Participation of both genders. | Page 11 |
| 5. Experience and training | *What experience or training did the researcher have?*  The researchers had previous scientific production, consolidated experience in conducting qualitative research in the Brazilian Amazon, and specific training for conducting interviews. | Page 11 |
| *Relationship with participants* |  |  |
| 6. Relationship established | *Was a relationship established before the start of studies?*  There was no previously established relationship between the participants and any of the authors before the beginning of the study. | Page 10 and 11. |
| 7. Participant knowledge of the interviewer | *What did the participants know about the researcher? (e.g., personal goals, reasons for doing the research).*  The researcher introduced herself to the participants at the beginning of the interview, with a brief personal history, and explained the objectives of the study. | Page 10 |
| 8. Interviewer characteristics | *What characteristics were reported about the interviewer/facilitator? For example, bias, assumptions, reasons, and interests in the topic of research.*  No bias related to the interviewer was identified. | N/A |
| **Domain 2: study design** |  |  |
| *Theoretical framework* |  |  |
| 9. Methodological orientation and Theory | *Which methodological orientation was declared to support the study? For example, grounded theory, discourse analysis, ethnography, phenomenology, content analysis.*  Deductive thematic analysis, based on the theory of Amerindian perspectivism. | Page 9 and 12 |
| *Participant selection* |  |  |
| 10. Sampling | *How were the participants selected? For example, with purpose, convenience, consecutive, snowball.*  The approach was intentional, the participants were identified with the help of health or education professionals who work in indigenous territories. | Page 10. |
| 11. Method of approach | *How were the participants approached? For example, in person, telephone, correspondence, e-mail.*  The contact with the participants took place in person after the study objectives were explained. | Page 10 and 11. |
| 12. Sample size | *How many participants participated in the study?*  Nineteen participants enrolled in the study | Page. 9. |
| 13. Non-participation | *How many people refused to participate or gave up? Reasons?*  There were no refusals or withdrawals of participants. | N/A |
| *Setting* |  |  |
| 14. Setting of data collection | *Where was the data collected? For example, at home, clinic, workplace.*  Most of the data collection was carried out in the indigenous villages. Two participants were interviewed at the *Indigenous Health Support House* (Indigenous Health House; CASAI) in Nova Olinda do Norte. | Page 11 |
| 15. Presence of non-participants | *Was anyone else present besides the participants and researchers?*  Yes, the health/education professionals who work in indigenous territories, who indicated the participants. | Page 10 |
| 16. Description of sample | *What are the important characteristics of the sample?*  Indigenous Munduruku caregivers over 18 years of age who were invited to participate in the study participated, and the detailed characteristics are mentioned in the manuscript. | Page 8 and 20 (table 1 and 3). |
| *Data collection* |  |  |
| 17. Interview guide | *Were questions, prompts, or guides provided by the authors? Was it pilot tested?*  Data collection was carried out through interviews guided by a semi-structured script by the group of researchers. | Page 11 and 12. |
| 18. Repeat interviews | *Were repeated interviews conducted? If so, how many?*  No. | N/D. |
| 19. Audio/visual recording | *Did the survey use audio or video recording to collect the data?*  The interviews were recorded using an audio recording device. The interviewer took field notes and they were later transcribed in full. | Page 10 |
| 20. Field notes | *Were field notes taken during and/or after the interview or focus group?*  The interviewer took field notes during and after the interviews. | Page 10 and 11 |
| 21. Duration | *How long were the interviews or focus group?*  The interviews lasted an average of 30 minutes. | Page 10 |
| 22. Data saturation | *Was data saturation discussed?*  Yes, this study probably did not reach full saturation due to the sample size. This is a sample of an indigenous population that is difficult to interview due to the need to preserve the identity of caregivers and elements of local culture. | Page 34 |
| 23. Transcripts returned | *Were the transcripts returned to the participants for comment and/or correction?*  No. | N/D. |
| **Domain 3: analysis and ﬁndings** |  |  |
| *Data analysis* |  |  |
| 24. Number of data coders | *How many data encoders encoded the data?*  Two coders. The initial coding was performed by researchers G.R.D and T.A.G. | Page 13 |
| 25. Description of the coding tree | *Did the authors provide a description of the coding tree?*  Yes. | Additional file 2 |
| 26. Derivation of themes | *Were the themes identified in advance or derived from the data?*  A face-to-face meeting was held to discuss disagreements and establish a common code book for the elaboration of the themes derived from the data. | Page 13 |
| 27. Software | *What software, if any, was used to manage the data?*  Atlas TI | Page 13 |
| 28. Participant checking | *Did the participants give feedback on the results?*  No. | N/A |
| *Reporting* |  |  |
| 29. Quotations presented | *Were the participants' quotes presented to illustrate the themes/findings? Has each quote been identified? e.g. participant number*  Citations constituted the study to explain the emerging themes, and each citation was identified with a participant number. | Page 13-26 |
| 30. Data and ﬁndings consistent | *Was there consistency between the data presented and the results?*  Yes. | Page. 13-26 |
| 31. Clarity of major themes | *Were the main themes clearly presented in the findings?*  The main themes were presented in the Results section, highlighted from large categorical groups. | Page 13-26 |
| 32. Clarity of minor themes | *Is there a description of various cases or discussion on minor topics?* | N/A |
