# Supplementary material for: Snakes and snakebites in the munduruku cosmology and medicine, central Brazilian Amazonia
Source: PLoS Negl Trop Dis. 2026 Jun 22;20(6):e0014462. doi: 10.1371/journal.pntd.0014462 (PMC13309027; doi:10.1371/journal.pntd.0014462)
Supplement: S2 File — (DOCX) [file pntd.0014462.s002.docx]

**ENTREVISTA 1**

**Caracterização do Entrevistado**

**Idade:** 64 anos

**Especialidade:** Pajé e pegador de desmentidura

**Informações Relevantes:**

**a)** Durante a entrevista estava presente o entrevistado, sua esposa e o guia da aldeia.

**b)** O pajé relatou que atua com essa especialidade há mais ou menos 4 anos, mas com pegação já realiza há muito tempo.

**c)**  O pajé relatou sua a experiência pessoal com mordida de cobras, o trauma que desenvolveu, os cuidados de resguardo, e sobre o tratamento realizado com recursos de plantas (açaí) e de animais (cobra e porco).

**d)** Ao final da entrevista ele relatou o uso da banha do porco “queixada” para massagem na região da mordida, informando que a banha do porco servia para acalmar a mordida e assustar as cobras (assim dizia o pai dele). Informações registradas no instrumento de pesquisa.

**Transcrição da entrevista**

[00:00:01]

G: Vou fazer a primeira pergunta ta, é..., me conte se existem histórias, lendas, mitos ou contos sobre cobras em seu povo? O senhor conhece algo de história aqui entre os Mundurukus sobre cobras?

EE1: assim, como, se diz assim

G: Lembra da história do boto que o boto tem um conto?

EE1: Ahamm....

G (Exemplificando): Tem que ele encanta as mulheres né, a cobra ela tem algo ou ela tem alguma história dentro da aldeia, em Nova Olinda quando eu era criança diziam em baixo da minha igreja da matriz existia uma cobra grande, né, eu cresci com esse pensamento, vocês tem alguma história aqui?

EE1: Existe, a cobra grande ela existe, só que as vezes nós diz que a cobra grande existe no rio, mas não é no rio, ela mora a metade no rio e a metade na terra, porque se ela morasse em cima do rio, ninguém poderia andar, passar onde ela tá. Ela é um animal que é muito grande.

G: E aqui na aldeia?

EE1: E aqui existe.

G: É e tem alguma história aqui sobre ela, assim que o senhor lembre?

EE1: Existe uma história né, não aqui né lá no outro rio, Paraná, e encantou uma moça chamada branca, foi a cobra grande que levou ela e hoje ela existe no fundo.

G: A moça?

EE1: Sim a moça. Não tem aquela saída do bem assim?

G: Sim, sei.

EE1: É lá que ela mora.

G: Eeee ela encantou como, essa moça tava fazendo o que?

EE1: Ela era uma moça [...] assim de ter um conhecimento, um dom que Deus deu para ela, e ela era muito assim de não andar sozinha por causa de que o curador falou para ela que um dia ia acontecer alguma coisa com ela e com a família dela, e justamente aconteceu, ela desceu para beira, e a cobra, o bicho levou ela. Ai.... o parente, a mãe o pai, se lastimarão muito de perder uma moça bonita, que ela era bonita e não volta mais até hoje. Então isso existe, como existe o boto, se o boto fazer qualquer coisa para você, você endoida, sente qualquer coisa, dor de cabeça, não consegue dormir sozinho, isso existe.

G: Na cobra também acontece isso?

EE1:Na cobra não acontece porque ela leva você, e fica como ela ta hoje né, até hoje ela tá, só que as vezes ela tende fazer mau para algumas pessoas, mais isso é muito dificilmente acontecer isso, mas as vezes acontece.

EE1: Às vezes quando você facilita demais, vocês mulheres, que ficam menstruadas, gostam de andar por cima do rio, então nesse momento que os animais do fundo, ficam irritado, que muitas vezes quando você tem alguém perturbando no seu terreno onde você mora, você fica irritado, o bicho do fundo, o animal do fundo é a mesma coisa, nunca ele gosta de sujar o terreiro dele que se diz o terreiro dele é a pessoa menstruada que não se resguarda ( ai acontece alguma coisa), o boto leva! Pega sua sombra, agora como? Porque ele vê você. Você não anda no rio e vê a sua sombra no fundo? A mesma coisa o bicho vê nós o ser humano.

G: Então no caso de cobras quando as mulheres estão menstruadas, eee, o ideal seria que elas não se aproximassem do rio?

EE1: É!

G: Ou ficassem em casa por exemplo?

EE1: Ficassem em casa se resguardando, ter aquele momento, o período dela, para depois descer, porque as vezes acontece, quando você se descuida acontece.

G: E quando acontece é mais assim voltado pro rio ou é se ela tiver por exemplo no roçado ou em algo também pode acontecer?

EE1:Não. Só no rio mesmo, o mais perigoso é no rio, o encantamento que aconteceu com a menina, foi no rio.

G: O senhor já teve alguma experiência com cobra aqui na aldeia?

EE1:Aqui no fundo?

G: No fundo ou fora! Já teve alguma experiência?

EE1: Pelo que me passou ainda não, não ....

G: Nem quando o senhor foi picado?

EE1: Agora quando eu fui picado de cobra é uma experiencia que você não vê, quando ela ta realmente para picar você, você não a enxerga. Que foi que aconteceu comigo tava numa roça, eu estava trabalhando igual nesse piso aqui. Ela por nada me picou bem aqui, e eu não sabia se era cobra. Eu proguntei a minha nora, dá uma espiada aqui, acho que foi o espinho que me estrepou, mas só que não tinha nada porque estava limpo onde eu estava trabalhando. Ela veio e disse parece uma mordida, ela e muito rápida. Ai fiquei assim, meu filho veio que estava lá comigo, papai parece uma mordida mesmo. A perna já foi ficando pesada, eu disse para ele um bora, um bora que o negócio não ta muito bom.

Ficando pesada a perna a modo que aqui o osso da canela estava estalando. Ai nos voltemos e tinha um pé de bananeira bem pertinho, olha para vê como a serpente é muito rápida. Ela me picou e foi se colocar embaixo da folha da bananeira. Aí ele pegou e levantou com o terçado ela estava lá enrolada.

G: Era grande?

EE1: Era mais ou menos uns 50 cm, meu filho disse papai foi cobra, então um bora, o polo base era bem aqui primeiro, quando cheguei aqui tem um igarapé do lado de lá do meu rancho, quando cheguei aqui, ai eu tinha uma sobrinha que estava gestante do primeiro filho, aí eu disse para ela né, minha nora, vai dizer para antônia que é nome dela, é uma agente de saúde. Diz para ela ir embora que o negócio não tá fácil. Ai ela subiu, ela trabalhava ai no polo base [...] e ai foi querer levantar para vê se conseguir andar, mas não consegui mais não, perna não ajudou mais.

G: E o senhor desmaiou daqui até lá, ficou desacordado?

EE1: Não, me lembro quando desceram aqui comigo ainda da lancha, quando chegou do furo não me lembro mais, fui me recordar já lá em Nova Olinda quando o finado machado que era um...

G: Sim, eu sei que era um técnico!

EE1: Era técnico que tava me aplicando o soro, aí que fui me recordar, o que foi Senhor Raimundo estava desmaiado mesmo, rapaz o negócio não e fácil não.

[00:00:09:05]

G: E as cobras elas possuem algum significado particular para o senhor?

EE1: Como assim?

G: assim a sua família ela tem um significado para o senhor né.

EE1: É.

G: Se o senhor fosse falar da sua família, numa palavra por exemplo, qual o significado da sua família para o senhor?

[Fez uma pausa para pensar]

EE1: Deixa-me pensar aqui [sorriu]. É porque para nós quanto família, a nossa família tem um significado muito grande entendeu, que é umas pessoas que esta do seu lado no dia a dia, como ela quando a cobra me picou ela não sabia o que fazia (esposa), porque o medo vem logo né, algumas coisas que acontecem. Eu tenho aqui um primo meu que é o Pedrinho, genro do compadre Jorge que ele puxando o lado da perna que foi mordido de cobra.

G:Aqui perto?

EE1:É!

G: Ainda puxa a perna?

EE1: Puxa até hoje, quase ele perde perna, e aí ficou, aparece a cicatriz.

G:A sua não aparece?

EE1: Não graças à Deus, mas eu sofri muito, sofre uns três anos, quando era lua nova, quando era lua nova, doía demais, eu sentia muita dor, [....]

G: Por conta de picada de cobra?

EE1: E eu graças a Deus não fiquei assim.

G: Que bom né, então sua família para o senhor é tudo?

EE1: É, pra mim minha família é tudo.

G: E as cobras o que são para o senhor?

EE1: [risos] agora as cobras não é [risos].

G: E se o senhor fosse falar do significado dela o que o senhor falaria para mim? Pensando nas cobras?

EE1: Nas cobras é um animal que você tem medo dela. Você não pode se encostar perto dela, até de você olhar ela você fica com medo, é um bicho, que acho que todo mundo tem medo dela, né. A onça, sempre digo pros meus filhos a onça não me faz medo, mas a cobra me faz, porque eu passei uns dois anos que eu não ia no mato.

G: Com medo da cobra!

EE1: Com medo, assim...

G: Deixa traumatizado né!

EE1: Com trauma!

G: Ainda era num lugar limpo né e rápido, sem esperar.

[00:00:12:05]

G: E o senhor acha que as cobras possuem alguma utilidade no seu dia a dia? Serve para alguma coisa a cobra no seu dia a dia? Tem serventia?

EE1: Pra mim não. Para mim não tem serventia!

G: Nem pensando em ....

EE1: Pra mim quando encontro uma cobra eu mato logo ela, eu não a deixo viva

G: É

EE1: Ainda mais quando é venenosa.

G: Nem pensando no couro dela, no óleo que ela tem?

EE1: Agora tem umas cobras que servem né, como a jiboia, a sucuri, que nós chamade sucuriju.

G: Hurum

EE1: Então a banha da jiboia ela serve para remédio e a banha da sucuriju também servi para remédio, por exemplo você faz uma cirurgia né.

G: Usa na cicatriz.

EE1: Usa na cicatriz!

É um cicatrizante, no caso!

EE1: Pra sarar, porque o sucuriju se fosse não matar ele ou cortar ele mau cortado ele vai embora, ele emenda, por isso que dizem que a banha dela faz isso. Qualquer cicatriz!

G: Ela mesmo se une.

EE1: É une ela só morre se cortar o espinhaço dela. Mas, sem ser isso se cortar mau cortado ela vai embora e sobrevive

[00:00:13:34]

EE1:

Aqui nós conhecemos a jiboia né, sucuriju, a surucucu que e pico de jaca, surucucurana também que é a mesma sururucucu mais é outro nome e a jararaca.

[00:00:13:52]

G: E qual o senhor acha mais perigosa?

EE1: É a pico de jacá

G: Pico de jacá e a surucucurana?

EE1: ......Ela faz o mesmo efeito porque, só que a pico de jaca, ela só pica você, se você esbarrar nela, ela não é uma cobra assim, gora a surucucurana ela é mais atrevida, se ela enxergar você perto ela pula mesmo, a jararaca também.

Eu já tinha escapado muito né, já tinha me assustado numa viagem com meu filho, pulei da proa da canoa, tinha uma bem, mas era a surucucurana. Eu dei um grito e fui no rumo da canoa de novo, a bicha fica muito valente.

G: Mas essa pegou o senhor inesperado.

EE1: Essa pegou sem esperar mesmo.

[00:00:14:48]

G: O senhor conhece ou sabe falar algum tipo, ou até o nome de cobra em Munduruku? Como chama cobra em Munduruku?

EE1: Na minha linguagem a cobra mesmo, o meu pai chamava de saicaiboi, na língua dele né, porque eu não sei se era isso mesmo por que eu nunca proguntei se era verdadeiro, mas, ele chamava assim.

G: Sacaiboi

EE1: Sacaiboi

[00:00:15:27]

G: (...) o senhor já realizou algum cuidado com alguém que foi picado por alguma cobra, em algum momento da sua vida?

EE1: Como assim?

G: Alguém da sua família, o senhor ou qualquer pessoa aqui da aldeia que foi picado, procurou o senhor, para o senhor cuidar?

EE1: Não!

G: Nem antes e nem após a picada? (Exemplificando)

EE1: Nem antes....

G: Não puxou nenhuma perna em nenhum momento? (Exemplificando)

EE1: De momento que puxaram a perna, foi só esses dois, o rapaz....

G: Que foi picado! Mas, o senhor chegou a cuidar dele

EE1:... Não a gente sempre diz qualquer coisa já leva para Nova Olinda, os enfermeiros dizem que não se pode usar remédio caseiro que vai atrapalhar o trabalho dele. Então nessa parte a gente respeita ne.

G: Hurum

[00:00:16:29]

G: Mas se o senhor tivesse que realizar o cuidado com alguém picado de cobra o que o senhor faria?

EE1: Olha a raiz do açaí batido e tirado o sumo, ele não fica bom, mas acalma a dor.

G: É um calmante!

EE1: É um calmante!

[00:00:16:48]

G: E como senhor faria do açaí, além de bater ele? Quanto tempo mais ou menos o senhor ia preparar queria que o senhor me falasse assim...

EE1: Não preparação dele é bem rapidinha. Você pega a raiz do açaí bate ele bem batidinho, se não quiser ferver bate ele, bota dentro duma água.

G: Se tivesse que ferver seria quanto tempo? O senhor botaria para fever?

EE1: Não, uns 20 minutos mais ou menos. Ele fica tipo um sangue né porque ele serve para tudo.

G: Amassa com as mãos, amasso com o pé?

EE1: Não, bate ele, e só faz

bater e deixa ele ferver, então bota na água, aí coa em um pano. Ele serve para tudo. E ele serve para duas coisas para picada de cobra e para anemia.

G: E a parte coada, o senhor ia colocar em cima?

EE1: Não você toma!

G: Toma né, ata entendi.

[00:00:17:39]

G: E esse cuidado ela faz efeito?

EE1: É tem que gente que diz quando faz isso né.

G: Mata a cobra e pega o couro dela mesmo?

EE1: Dizem que acalma mais.

G: Hurum, no caso...

EE1: Não, nunca fiz até porque quando fui picado não me lembrei que já tava pensando [risos]

G: Já tava chegando em Nova Olinda desmaiado.

EE1: É

G: Então (...) essas foram as perguntas, é, eu agradeço muito o Senhor!

**ENTREVISTA 2**

**Características do entrevistado**

**Idade:** 53 anos

**Especialidade:** Parteira

**Informações Relevantes:**

**a)** Na entrevista estavam presentes a entrevistada e a guia da aldeia.

**b)** A entrevista foi realizada na residência da parteira.

**c)**  A parteira informou que realiza partos há mais ou menos 29 anos na aldeia e em suas proximidades.

**Transcrição da entrevista**

[00:00:03]

EE2: E que eu também não sei de muita coisa não...

G: Tá, o que a senhora souber já é importante tá!

G: Eeee, a senhora sabe alguma história que envolva cobra, pode ser lenda, mitos contos?

EE2: Pra remédio....

G: Qualquer história assim, que tenha acontecido aqui.

EE2: Aqui já aconteceu várias gente mordida de cobra, mas realmente, diz o pessoal, tem uns que diz que engole o rabo da cobra, tem outros que diz que mata a cobra e coloca o bucho em cima né, realmente isso as vezes acontece aqui, não é todas pessoas que faz isso, mas tem muitas que faz né. E aí pra ferrada de bicho que eu já falei né do lacrau, que a gente molha a farinha e coloca em cima né, comigo já aconteceu isso.

[00:00:58]

G: A senhora já teve alguma experiência com ferrada de cobra?

EE2: Com cobra nunca me mordeu e nem quero! Eu tenho é medo!

G: E alguém da família?

EE2: Só mesmo meu filho que foi mordido de cobra.

G: Mas a senhora chegou a fazer algum tratamento?

EE2: Pra ele eu não fiz tratamento nenhum porque realmente ele foi embora logo, levaram ele para Nova Olinda né.

G: Ele que fez?

EE2:Ele que já que fez, no caso dele foi ele que fez! Realmente não foi eu.

G: Humrum

EE2: E ferrada de arraia como já falo tá no negócio de bicho, ele também foi ferrado de arraia, a senhora sabe qual foi o remédio dele? Foi a rapa do caju branco. A gente não dá nada pelo caju branco, a gente rapa ele, amorna e bota em cima da cisura, é bater e ver. É água em cima do fogo. E a gasolina também que diz o pessoal também que a gente usa para motor e isso também é muito bom ferrada de bicho.

G: No caso da cobra também?

EE2: No caso da cobra nós nunca coloquemo, só em ferrada de lacrau

G: Arraia?

EE2: Arraia, essas coisas assim

[00:01:58]

G: Entendi, e a senhora pode repetir o que a cobra significa para a senhora?

EE2: É um animal né, pra gente é um animal, que morde a gente né

G: E ela tem alguma utilidade no seu dia a dia?

EE2: Eu acho que tem né.

G: Qual utilidade ela serve pra senhora?

EE2: O couro da cobra diz o pessoal serve para remédio, para fazer uma defumação, a banha serve contra diz o pessoal os malefícios que falam né, que é a judiaria, tudo para isso serve, tipo de banha de cobra, de couro, tudo.

G: Em que situação a senhora usaria por exemplo o couro para defumar?

EE2: Para defumação, porque os curadores ensinam né, dá o remédio é bom, eles começam a ensinar. É couro de cobra, é espinho de cuandur que é um bicho que a gente tira aquele espinho dele para fazer a defumação, e aquilo é muito bom para remédio. É bom também pra botar, fazer a pulseirinha da criança para não pegar quebranto, tudo aquilo é bom!

G: Da cobra?

EE2: Não do porco espinho. A senhora já ouviu falar do porco espinho?

G: Sim já!

EE2: Pois é. O espinho dele é muito bom para isso.

G: No caso da cobra assim não tem um....

EE2: É só para defumação e a banha para remédio né, a gente, a gente não tira, os outros que tiram e a gente compra né.

G: E que tipo de remédio a senhora usaria a banha da cobra?

EE2: É [risos] só para tirar o maleficio que diz que é bom, falam que é bom, eu nunca usei esse tipo de banha, mas tem gente que diz que é bom. Nunca cai ainda na oportunidade de usar né.

[00:03:48]

G: A Senhora conhece algum tipo de cobra?

EE2: É surucucu que eu conheço né, surucucu aquela outra cobra pico de jacá que é a mesma surucucu, só que os pessoal uns chamam de um jeito, outro chamam de outro. Tem aquelas boiacica, a cobra mais perigosa que falam que é né. E tem...

[00:04:01]

G: Boiacica é a mais perigosa?

EE2: A boiacica é a mais perigosa ela não tem zoio aquela cobra lá, e tem aquela papagaio a cobra papagaio também, tem todo tipo de cobra, aquela falam que lamba na gente eu não sei que nunca me lambou.

G: Dessa todas que a senhora me falou qual a mais perigosa?

EE2: é a surucucu.

[00:04:27]

G: E a senhora sabe me falar o nome da cobra em Munduruku?

EE2: Não eu não sei falar, mas eu tenho filho que é profissional para falar!

G: É!

EE2: Só que ele não ta aqui

G: Onde ele ta?

EE2: Ele ta lá na reunião

G: Como é nome dele?

EE2: Ele é profissional para falar isso daí

G: É mesmo é? Vou procurar ele.

EE2: Ele estudou para isso [risos]

[00:04:51]

G: E a senhora, e se tivesse alguém se por exemplo viesse, se deparasse aqui na sua casa pedindo uma ajuda para senhora cuidar porque foi picado por uma cobra...

EE2: Eu ia ajudar né.

G: E o que a senhora faria?

EE2:

EU faria que tem uma planta por nome tajá, é um tajá.

G: Igual um coração eu sei.

EE2: Exatamente, aquilo é muito bom para ferrada de cobra de tudo quanto é bicho,

Aquilo é muito bom.

G: Como a senhora faria com tajá?

EE2:Ralava né, a gente rala e emplastava em cima.

G: Mas a senhora primeiro coloca na água ia ferver algo?

EE2: Não ia ferver nada, ralava e colocava em cima, amornar que a gente amorna.

G: Quanto tempo a senhor deixaria?

EE2: Acho que com uns 5 a 10 minutos por aí.

[00:00:05:48]

G: Eee se e a senhora tivesse que fazer esse cuidado, a senhora faria ele antes ou após?

EE2:Eu fazia antes logo que chegasse, que se deixar para depois a pessoa piora né.

G: E esse cuidado a senhora aprendeu com alguém da sua família ou alguém que lhe ensinou...

EE2: Não

G: Ou a senhora já testou em algum momento e deu certo?

EE2: Não. Euuu sempre diz o pessoal eu ando muito por aí vejo os pessoal fazerem né. E aí eu faço......

G: Então a senhora aprendeu com outras pessoas?

EE2: Porque a minha mãe que me criou ela tinha essa experiencia ai, que me criou porque eu tenho mesmo minha mãe legitima, só que eu não me criei com a minha mãe. que a minha mãe me deu com a idade de 3 anos e outra mulher que me criou. Não foi realmente minha mãe. Ai depois meu marido morreu e eu fiquei com meus filhos, uma meninazinha de 5 anos e os outros ficaram comigo e eu trabalhando dando murro para criar meus filhos. Se criaram tudinho, são tudo homem.

G: Tão bem graças a Deus

EE2: As meninas já tem tudo família, uma mora lá para canumã, diz que ela é pajé né. Que ela teve uma arreação.

G: Lá no Canumã?

EE2: Ela mora lá nas Fronteiras.

G: A ela mora lá nas Fronteiras é? Ela é pajé?

EE2: Ela é pajé falam que ela é, se ela fizer um trabalho que ela trabalha né, se ela fizer um trabalho você ta passando mal, se ela disser assim esse trabalho, esse paciente vai ficar bom me pertence, e ela vai cuidar a pessoa fica bom, e se ela disser esse paciente não é para mim. Então procure um médico, que não é comigo. E aí a pessoa tem que procurar um médico porque realmente não é com ela. E ela mora para lá, ela já veio aqui que ela tava de parto, teve um nenê, foi operada. Aí ela foi embora e ta para lá, mora pra lá. O marido dela é de lá, acho que vocês conhecem seu (...), ele trabalha como agente de saúde.

[00:00:08:11]

G: E se a senhora tivesse que fazer esse cuidado, a senhora faria associado ao cuidado médico ou sem o cuidado médico?

EE2: Sem o cuidado do médico.

G: É né, ta!

EE2: Porque as vezes também os médicos a gente nem se confia quase né.

[00:00:08:33]

G: E esse material que a senhora usaria, ele estaria acessível. É acessível assim para a senhora encontraria com facilidade?

EE2:Agora aqui na ocasião a gente não tem porque o tem que água, veio o verão muito forte né ai matou tudo, morreu tudo. A gente fiquemo sem planta.

G: Entendi.

EE2:Morre tudo, até nossa roça morreu metade da nossa roça, que o verão foi muito forte, acabou com tudo. Metade das plantas morreram.

G: Então por exemplo se acontecesse hoje quanto tempo a senhora teria para encontrar o material.

EE2: Hum eu acho que aqui ta difícil mana.

G: É?

EE2: Tempo ta difícil porque não é todas as pessoas que planta. E isso é um planta não é todas as pessoas que planta.

[00:00:08:33]

G: E senhora eee usaria algo da cobra ? Da própria cobra?

EE2: Só se for para fazer remédio né. Pra criar eu não vou usar, mas para remédio a gente usa.

G: Pois é se tipo chegasse alguém mordido pela cobra a senhora usaria algum remédio, da própria cobra, tipo uma parte dela? Algo dela.

EE2: Eu ouvi falar que o rabo.

G: E a senhora usaria algo da cobra, da própria cobra?

EE2: Só se for para fazer remédio né. Pra criar eu não vou usar, mas para remédio a gente usa.

G: Pois é se chegasse alguém mordido pela cobra, a senhora usaria algum remédio? Da própria cobra tipo uma parte dela, algo dela?

EE2: Eu ouvi falar que o rabo da cobra.

G: É?

EE2: Engole, toma.

G: Toma é? Mas a senhora?

EE2: Eu ouvi falar que engole o rabo da cobra.

G: Sem nada? Ou ela com água? Bate ele?

EE2: Nada ele inteiro, só o rabinho.

G: Só aquele restinho! Mas, a senhora já soube alguma história ou conheceu alguém que engoliu e deu certo?

EE2: Não eu vejo falar, parece que meu filho não sei se foi ele ou uma pessoa que engoliu o rabo da cobra, falam que bom né.

G: Hurum

EE2: Que o veneno não sobe.

G: No caso não subiu né?

EE2:Humhum, subiu não

**ENTREVISTA 3**

**Características do entrevistado**

**Idade:** 93 anos

**Especialidade:** Parteira

**Informações Relevantes:**

**a)** A entrevistada dispensou a companhia de um familiar no momento da entrevista.

**b)** A parteira informou que realiza partos desde os 17 anos de idade e que sua especialidade em 2020 devido a idade avançada.

**c)**  A aldeia onde a entrevistada reside é a mais próxima de Nova Olinda do Norte e é constituída por um número expressivos de seus familiares (Filhos e netos).

**d)** Em sua entrevista ela relatou o uso de limão e pólvora.

**Transcrição da entrevista**

[00:00:1]

G: Vou fazer pergunta pra senhora, é..., eu gostaria que a senhora me contasse se existem, é, histórias, lendas, mitos ou contos com cobras na sua etnia?

EE3: É porque a cobra, ele é uma coisa que eu tenho muito medo, muito medo. Se eu tivesse, [...] eu fui uma mulher que eu andava no mato, eu cortava seringa, eu tirava sorva, eu tirava copaíba, eu pintava o sete né, mas se eu tivesse medo da onça, que nem eu tenho da cobra, eu não andava no mato. Sabe por que? Porque a cobra ela se enrosca em qualquer cantinho né, e a onça a gente está enxergando ela longe né, e lá... Quando eu entrava no mato, eu já sabia, entrava com uma espingarda, porque todo tempo eu atirei né, atirava, matava caça, matei peixe de zagaia, eu não tinha precisão de homem na minha ilharga. Mas hoje, hoje já reparo quê... que eu fez, eu não faço mais, de jeito nenhum. Eu pegava o machado, eu derrubava roça, eu plantava roça, eu fazia tudo só eu, sem carecer de marido na minha ilharga, mas hoje eu não faço, nem tirar mais um pau, porque me apareceu uma dor nesse braço aqui que eu tenho, pelejando pra ver se fico boa, mas até agora, no momento nunca mais eu fiquei, não sei o que é. Não sei se é reumatismo, desmentidura, o que é. Mas... mas eu tenho fé de ficar boa ainda do meu braço, eu mandei fazer até uma roça pra mim, disque pra plantar banana (risos).

[00:01:26]

G: E nesse tempo a senhora teve algum momento, assim, alguma experiencia com cobras assim, que a senhora recorda assim, da história dos seus antepassados ou por exemplo, de alguma história que envolveu cobra, assim que marcou sua vida?

EE3: Não!

G: Não né.

EE3: Porque da minha família, só mesmo esse rapaz que foi mordido, que devido eu ter muita família, a família é grande, tenho filhos, tenho 8 filhos. Fui mãe de 15 filhos; 11 mulher e 04 homem, mas eu só criei 8, o resto morreu né. Mas, graças a Deus só foi esse rapaz, meu genro que foi mordido de cobra, que eu te contei naquela hora né.

G: Uhum

EE3: Que eu fiz remédio pra ele do cipó do boto né, e ele ficou bom. Agora eu não sei se todos ficam né. Agora eu já ensinei pra diversas gente, já tenho-me contado disque é bom, muito bom mesmo, porque boto a senhora sabe né, mora na agua, ninguém não sabe as experiencias que ele tem. Eu sei que nesse dia eu sei que foi bom porque eu fez pro rapaz e ele ficou bom, agora outra vez não porque também só me apareceu só essa vez aí, esse rapaz mordido, nesse tempo todinho. E sabe de a gente andar no mato, desde criança, meu pai me criou andando no mato, por aí, mas eu acho que é isso mesmo né, a gente tem que vencer tudo né.

[00:02:48]

G: Uhum... As cobras assim, quando a senhora vê elas, elas possuem assim algum tipo de significado particular pra senhora?

EE3: Ramm! Elas correm de mim.

G: É?

EE3: É! elas têm medo, elas não ficam me espiando não, porquê... de primeiro os velhos tinham um negócio nas orações pra cobra né, pra tudo né. Se você via uma cobra, se você entrava no mato você rezava, se você fosse pra água, fosse pra fogo... isso tudo eu sabia. Eu sabia oração pra água, eu sabia oração pra fogo, eu sabia pra cobra... eu sabia me defender no mato, se por acauso, a senhora chegasse assim, ah, eu vou matar aquela mulher ali! Eu vou isso, eu vou fazer aquilo! Nunca. No dia que ela chegar perto de mim, a gente vai brigar, isso eu sabia. Se eu soubesse quando eu chegasse na sua casa, eu rezava aqui tal, tal, tal... e... quando eu chegasse lá, a senhora tava me abraçando com as duas mãos. Hoje não me pergunte, que eu não sei.

G: E quando a senhora ver alguma cobra assim, em algum momento. A senhora já fez isso, de feito alguma oração?

EE3: Não, não senhora. Mas, elas não gostam de mim não.

G: É?

EE3: Eu chego por acaso, ê... uma cobra aqui, a bicha já vai estirando em rumo do mato, se der de eu matar ela, eu mato. No tempo que eu atirava, que eu era bom da minha vista eu pegava uma 20, oh! Pá na cabeça dela... hoje não, só se for de cacete, e se for bem pertinho, porque eu não enxergo mais, olha, Essa botija aí eu tô enxergando um negócio desse tamanho.

[00:04:20]

G: E se a senhora fosse pensar assim, o significado dela então, como que a senhora classificava, como a senhora... como poderia dizer que a cobra é um animal que ele é um animal do bem, que ele é um animal do mal, maléfico, como que senhora ver a cobra?

EE3: Eu acho que ele seja, pra mim, na minha ideia, que eles um bicho muito mal né, porque eu já tenho visto muitas histórias de cobra, muita mesmo.

G: Conte uma história pra gente.

EE3: Eu tinha um sobrinho, um filho de criação, que o meu marido criou ele desde pequeno né, aí ele ficou distante de nós, mais ou menos uns 15 dias né. Aí nós baixemo pra baixo, ele ficou pra cima, quando nós subimos soubemos só da história que a cobra tinha matado, ele foi andar no mato, pisou em cima de um pau, escurregou, caiu deu num buraco né, lá dentro do buraco a cobra mordeu ele, que mordeu na perna. ai quando ele pulou de dentro do buraco pra coisar, ela foi, pulou, pegou bem na cara dele assim. Aí disque ele ainda veio meio caxingando, caxingando até que ele chegou na casa, quando chegou na casa, tinha uma tia, aí disque ele deu pólvora com limão pra ele, fez uma porção de remédio caseiro né, que ela não sabia também né. Aí ela fez tanto da coisa, quando amanheceu o dia, ele já amanheceu morto.

Outro também, eu tinha uma cunhada, a minha sogra, mãe desse homem que eu vivia com ele ne, aí disque um dia eles iam subindo, aí ela tinha um macaco, o macaco era danado pra mexer com quem estava quieto né, aí disque ela disse: eu vou pescar. Ta bom eu vou cortar uns gomos, aí disque ela encostou na beira pra cortar os gomos, pra tirar aqueles bichos pra pescar né. Aí disque quando ela estava lá encostada, o macaco pula de lá de perto. Quando ela pulou de lá em terra, ela tinha uma meninazinha dela, que andava com ela, tinha uns cinco anos, tava lá sentada no meio da canoa, quando ela viu a cobra pulou de lá de terra, pei na bera conoa, mordeu bem a criança no braço. Ela também era uma mulher muito disposta, pegou essa cobra, retalhou tudo quanto... e a criança gritando, no porão da canoa. Quando foi sete horas da noite ela morreu. Isso é ela contou pra mim, ainda não foi do meu tempo, era uma historia que ela me contou né.

G: Hurum

EE3: E cobra eu tenho medo, tenho, eu tenho medo.

[00:06:48]

G: E a senhora ver a cobra como alguma utilidade no dia-a-dia. Ela tem alguma serventia, durante o dia-a-dia?

EE3: As vez ouvi dizer que tem gente que diz que tira banha delas pra fazer remédio. Eu sei que nunca tirei, nunca nem peguei, é a coisa que eu tenho mais medo. Agora a tal de sucuriju, não, isso aí eu tiro a banha, porque eu já tirei. Meu filho até o ano passado matou uma, lá onde nos morava. Parti ela assim, tirei bem uns cinco litros de banha.

G: E a senhora tirou pra quê?

EE3: Pra remédio. Aquilo é muito bom, pra pessoa que tá operado, beber né. Porque aquilo a gente retalha hoje aqui, pode largar, quando é amanhã, depois da manhã, cê vem espiar tá emendadinha, saradinha, você não sabe nem onde que foi cortado. Então a banha dela serve pra tudo né, pra beber, pra passar em cima, pra tudo a banha dela é boa.

[00:07:38]

G: É... e a senhora é... que tipo de cobra que a senhora conhece?

EE3: Eu só conheço bem a surucucu. A surucucu que chamam como é, pico de jaca, e a surucucurana, e a surucucu cor de fogo. Essas três eu tenho conhecimento bem com elas.

G: É né

EE3: Agora outro tipo de cobra, só naquela jararaca d’agua também tenho bem conhecimento com ela, jararaca d’agua e a sucuriju. A sucuriju, ele, era uma cobra que vevi em todo canto.

G: Hurum

EE3: Em todo canto ela vive..

[00:08:11]

G: E quais dessas cobras a senhora conhece, e ate as que a senhora não conhece a senhora considera a mais perigosa?

EE3: É a surucucu, dessa pico de jaca.

G: A pico de jaca né?

EE3: É, sim senhora. É a mais perigosa, porque ela... essas outras surucucuna, surucucu de fogo essas outras qualidades, pelo meno, ela só morde a pessoa, se pessoa mexer com ela, ou pisar, ou seja, lá o que for né. Mas, essa aí que eu tô falando, a surucucu, ela pula sem a pessoa mexer com ela já tá pulando. A basta ela vê o rosto, ali na sombra, ela já pulou. É a mais perigosa!

[00:08:47]

G: Na linguagem Munduruku, ainda que a senhora não fale mais, mas talvez a senhora tenha assim, alguma lembrança que a senhora recorde. A senhora é... lembra se tinha algum nome munduruku que chamavam algum tipo de cobra?

EE3: Não senhora!

G: Não, né.

EE3: Acho que não.

G: Se por exemplo eu desse uma folha dessa de papel, é, eu pedisse pra senhora desenhar uma cobra. A senhora conseguiria desenhar?

EE3: Não

G: Não?

EE3: Não sei nem pegar lapiseira.

G: É... Não? Ta bom.

EE3: De primeiro ainda fazia aqueles negócios de bordados, pelo menos eu desenhei esse ramo aqui, eu ainda desenhava. Mas hoje eu não sei mais por onde que vai.

G: Entendi. Ta bom.

EE3: Minha vista não dar mais de jeito nenhum.

[00:09:34]

G: No seu dia-a-dia a senhora realiza algum cuidado em pessoas picadas por cobras?

EE3: Ah... agora no momento?

G: Uhum...

EE3: Ainda ia fazer um remédio se aparecesse né

G: O que senhora faria?

EE3: O que eu ia fazesse por acauso fosse, por causo... a gente tem uma experiencia né.

G: Uhum...

EE3: O limão com a pólvora ele é muito bom, muito bom mesmo. A gente pega o limão, espreme, quando acabar a gente pega um pouquinho de pólvora, que nem isso existe mais também, é pólvora. Da que eu fazia remédio, que é daquele pólvora preta né. Dessas pólvoras de hoje em dia é só já aquele a gente bota na água, e nem dissolve aquilo.

G:Uhum

EE3: Então, a gente pegava o limão, pegava a pólvora exprimia dentro e dava pra pessoa beber. Se a pessoa baldiasse, aquele baldia, baldia, balsia, todo o veneno que tem, agora se ficasse preso dentro, a gente tem que procurar outro remédio para dar né.

A gente sabe outros remédios caseiros assim, de pau, de folha, a gente vai fazendo né, tu vai lá e da certo, e as vezes não da certo, aí ninguém. Nunca me achei nesses assuntos, só como eu falei, só nesses assuntos que eu falei. A do meu genro, esse aí eu tratei dele, e ficou bom né. Agora outros... já tenho visto muito falar né, mas nunca chegou no meu alcance não.

[00:10:55]

G: A senhora poderia assim, é... me esclarecer, além desses cuidados é... quanto tempo a senhora teria pra preparar se aparecesse alguém com cobra. Por exemplo, a senhora me falou que usaria limão e a pólvora, né. Quanto tempo mais ou menos dura pra senhora preparar esse tratamento?

EE3: Assim, por acaso a gente tendo no momento a gente faz logo. Agora se não tiver, aí é difícil. Que nem um, em lugar é meio difícil de fazer. Primeiro que a pólvora não existe mais, né. O limão até que a gente ainda acha, pra... ao menos pra comer mais...

G: Teria algo que a senhora poderia substituir ao invés da pólvora?

EE3: Ah... tem muito remédio que faz com a pólvora. Olha por acauso cê tem um animal, cria um cachorro, esse cachorro ele é ruim, não tem coragem... Você pega uma pitada de pólvora põem na comida e da pra ele comer, dá que uns dias ele tá um cachorro esperto pra todo canto, que eu sei a pólvora explode né, tudo isso a gente intende né, do tempo que eu era nova, que eu tinha certas ideias de andar. Hoje em dia até pra privada é ruim, imagina pra andar né.

[00:12:08]

G: Se a senhora fosse pensar em tempo, se tivesse disponível por exemplo a pólvora e o limão, quanto tempo mais ou menos a senhora conseguiria preparar esse remédio?

EE3: A gente perpara só na hora que é preciso, né.

G: (exemplificando) Mas leva quanto tempo, por exemplo assim horas, minutos?

EE3: Não, que aquilo a gente põe na vasilha né, aí mexe-mexe, bate bem batido, aí dá pra pessoa beber.

G: (indagando) uns 30 minutos mais ou menos?

EE3: É mais ou menos

G: É bem rápido né

EE3: O que pode ser né. Aí a gente vai esperar o resultado, se a pessoa provoca ou não né. Porque quando veneno tá perigoso, no que a pessoa bebe, não demora ele tá provocando sangue purinho.

G: Que é o baldiamento no caso?

EE3: É o por que faz a inalação do inseto na pessoa, é o sangue né, é o sangue que fica preso, né, mata a pessoa na hora

G: Seria como se fosse uma limpeza né?

EE3: É... é uma limpeza.

G: No sangue né

EE3: É sim senhora!

[00:13:01]

G: E... a senhora..., esse cuidado que a senhora poderia realizar com alguém que foi picado pela cobra, ele, a senhora herdou de alguém?

EE3: Não, nunca herdei.

G: Não

EE3: Só esse mesmo que eu falei... do meu genro.

G: Do seu genro no caso

EE3: Foi, era meu genro, ele conta... ele não tá aqui porque ele tá trabalhando no Apuí, lá... muito longe daqui.

G: Por exemplo... dos seus pais, dos seus avós...

EE3: Nunca também tratei isso. Eu também não teve. Meu pai morreu eu teve pequena, minha também, meu avo não teve de jeito nenhum (risos) me criei assim andando. Tenho visto me contarem muita história assim, tanta história que ninguém não pode nem contar né.

G: uhum... e se por exemplo uma pessoa ela é picada pela cobra, em qual momento a senhora faria o cuidado e por quanto tempo? Por exemplo... a (...) foi picado por uma cobra, ela chegou aqui, a senhora faz de imediato o cuidado, a senhora espera algumas horas e a senhora faria assim (exemplificando) durante uma semana... durante um mês... durante dois, três dias... qual o primeiro momento que a senhora faria. Logo imediato, se disseram assim, oh. Fulano de tal foi picado por uma cobra, a senhora já fazia de imediato?

EE3: Aí o que eu poderia fazer, era assim, fazer um remedinho e direto logo pra onde tivesse recurso né. Ia pra Foz, de Foz para Nova Olinda, se não dá jeito vai pra Manaus né.

[00:14:37]

G: E quanto tempo a senhora ficaria tentando pra ver se o tratamento daria certo ou não deu certo?

EE3: As vezes a gente presta atenção em um dia, dois ou três né. Se não da certo, aí tem que cuidar no outro caminho né, porque ninguém não vai ficar com uma pessoa, sem saber o que fazer né.

G: E geralmente quando faz esse cuidado né, porque eu lembro que a senhora contou uma história, é... de um tratamento que a senhora fez né, que só foi retratamento, a senhora não encaminhou assim, porque não tinha ainda medico né?

EE3: Não, não, não senhora tratado em casa mesmo

[00:15:11]

G: Então geralmente a senhora faz esse cuidado sozinho, só o cuidado da seu ou geralmente a senhora faz o seu cuidado mais o cuidado medico?

EE3: Ele... quando eu tratei dele, só quem mexia com ele, era a mulher dele mesmo, ela que fazia comida dele, eu mandava ela fazer. Não deixar ele andar porque quando pisa no carvão que é perigoso né

G: No carvão

EE3: No carvão disque é perigoso né, e aí eu não deixava ele tá andando assim... porque nós criava galinha né, ao menos até 8 dias, depois já pode fazer estrepolia né, porque essa cobra é bicho perigoso, rum... eu tenho tanto medo, tenho medo mesmo, por isso que eu digo se eu tivesse medo da cobra, como eu tenho da onça que nem tenho da cobra eu não saia nem de dentro de casa, principalmente hoje, porque hoje eu não enxergo, as vezes eu vou andando por aí, com cipózinho fino, se tiver um pau, lá longe... espalhando, porque eu não sei se é ou não né.

G: Sempre observando né.

EE3: É mana, eu tenho muito medo.

[00:16:15]

G: Desses cuidados que a senhora fez pra quem estava picado por cobra, é... teve algum momento alguma melhora?

EE3: Não eu nunca cuidei. Desse rapaz que eu disse que cuidei dele né, graças a Deus ele desde a hora que dei pra ele, ele sossegou, deitou na cama, porque disque na cama é perigoso né, e deixou na cama, e ele ficou... depois algumas horas ele baldiou, começou sair sangue, saiu aquele sangue aí fiquei com medo. Que lá não tinha pra onde ir, lá era perigoso, porque quantas horas, quantos dias ele não ia gastar porque não tinha médico. Aí fiquei, fiquei... ai disse vamos dá um chá de erva doce pra ele, e eu fui, fez o chá de erva doce, eu dei também, ai pronto tancou. Mas, ele já tinha provocado muito, já tinha provocado sangue, puro, puro... saia bem assim, pros olhos dele, sangue né, aí pelos dentes dele.

G: Esse chá de erva doce a senhora só deu vez?

EE3: Só dei uma vez de erva doce, e do negócio do cipó do boto só dei uma vez também, pronto. Graças a Deus, foi o que tratou dele.

[00:17:21]

G: Pra fazer esses cuidados é fácil adquirir os materiais ou nem sempre?

EE3: É meio difícil, o cipó do boto é meio difícil a gente arrumar olha, que desde esse tempo aí acabou, eu arrumei, que também não serve só pra isso, serve pra outras coisas né. Eu tinha uma cunhada, aí disque o menino dela andava doente, doente... de uma tal de aí eu dei também pra ela fazer o remédio, aí acabou. Sempre eu peço, gente eu queria um cipó de boto. Rumm... nem a banha, que a banha também é bom pra remédio, a banha do boto.

G: Então que possível a senhora pede pra ter em casa, no caso

EE3: É... porque a gente precisa né. Um lugar que nem esse aqui, a gente precisa ter uma banha, um remédio. Tem gente que você ver aqui no interior, não tem uma pílula ao menos pra tomar um remédio. As vezes dói meu estomago, vou tomar essa pílula não tem. Pois eu sou prevenida nesse ponto, eu gosto de ir na cidade, eu tenho um dinheiro, tudo eu gosto de comprar meu remédio pra ter alí.

Na hora... aí eu tô com dor no dente, vai lá na tabua pega uma pílula daquela, toma né. Pega uma pílula bota no dente, no ouvido, seja lá o que for, eu sou prevenida nesse ponto. Todo tempo eu foi, quando eu morava no Sasmi, no mato assim, eu tinha uma caixa assim, todo que era tipo de remédio. Eu não sabia ler né, nunca soube.

Mas, quando eu comprava eu mandava o cara ler pra mim, como era que tomava, como era que tratava, como é que não era. Ai a pessoa leu, ficava na minha cabeça, no dia que a pessoa precisa tratava né. Não carecia de fulano como é que trata assim como o cara tinha me falado tratando dele.

[00:18:58]

G: Quanto tempo mais ou menos, é... porque a senhora só tem um caso de pessoa que foi picado né, mas assim de outras vivencias quanto tempo a senhora acha que a pessoa que ela é picado por uma cobra ela busca, por exemplo o serviço médico?

EE3: Não sei. Já faz muito anos, já faz até tempo que eu nunca mais vi ele, tá fazendo uns dois anos.

G: Mas se acontecesse aqui hoje por exemplo, quanto tempo mais ou menos vocês fariam primeiro um cuidado tradicional ou encaminhariam para Nova Olinda.

EE3: Ia encaminhar, porque agora aqui no momento não tem nada. Ia encaminhar ele para Nova Olinda porque, a foz do canumã ai é tempo perdido. A gente vai alí e não tem nenhum remédio.

[00:19:40]

G: Entre estes recursos que a senhora vivenciou... a senhora utilizou algum recurso da cobra, tipo o veneno, a pele dela, ou amarrou por exemplo onde foi picado a região?

EE3: É... não, eu vejo falar que disque tem gente que quando ela morde na perna, a pessoa amarra na perna, eu já ouvir falar né, que disque o veneno não sobe, mas eu nunca vi acontecido, nunca vi.

G: Também nunca fez? Entendi.

EE3: Nunca fiz também, nunca vi, nunca fez... já ouvi falar né, que as vezes a gente conversa como esta a gente né... fulana aconteceu isso... assim a gente tá escutando né,

G: Tá observando

EE3: É... mas nunca fiz graças a Deus. Só esse aí mesmo. Esse daí eu conto porque aconteceu foi comigo.

G: E porque procurava a senhora mais pros partos né.

EE3: Era, agora pra parto era daqui pra li, me procuravam né.

G: Então (...) muito obrigado, tá, porque a senhora aceitou participar ta bom, e a gente é muito grato pela sua participação. Fique tranquila que a gente não vai divulgar seu nome tá, e nada que é seu, ele vai ser informado. Apenas a sua vivência.

EE3: Ta bom.

G: Muito obrigada tá... (risos)

EE3: Obrigado também.

**ENTREVISTA 4**

**Caracterização do Entrevistado**

**Idade:** 59 anos

**Especialidade:** Parteira

**Informações Relevantes:**

**a)** Na entrevista estavam presentes a entrevistada e a guia da aldeia.

**b)** A entrevista foi realizada próximo a residência da parteira, onde seus familiares estavam realizando o preparo da mandioca para a produção de farinha. Foi possível observar a participação das crianças e adolescentes neste processo.

**c)**  A parteira informou que realiza partos há mais ou menos 28 anos na aldeia e em suas proximidades.

**d)** Ao final da entrevista a interlocutora foi até sua residência para mostrar os couros de cobra que havia colocado para secar e para utilizar em defumações e tratamentos.

**Transcrição da entrevista**

[00:00:03]

G: É (...) eu gostaria que a senhora me contasse se existem histórias, lendas, mitos ou contos com cobras aqui na sua aldeia ou com o seu povo?

EE4: A cura da cobra é?

G: Não, que a senhora me contasse se tem histórias, lendas, mitos ou contos, se alguém contou alguma história, se a senhora sabe alguma história desde criança que seu e sua mãe contavam?

EE4: Sei, uma história dessa da minha sogra né, ela me contou, que ela adoeceu, ela adoeceu, aí a barriga dela ficava, ficou grande, ela pensou que estava gravida mesmo né, mas não era. Era gravidez de cobra, porque, ela falou para mim que ela teve duas cobras, duas cobrinhas. Ai essas duas cobras aí, ela disse que não mandou matar não, ela soltou, mandou soltar n’água, aí ela quase morre.

G: Sua sogra que contava?

EE4: Minha sogra, ela contou pra mim, porque ela ainda é viva minha sogra, aí ela me contava né.

G: Ela mora aqui?

EE4: Não, lá no paraná, ela é de la, e aí ela disse que quase ela morre com esse tipo de bicho né, assim dentro dela.

G: Ela contava qual era o tipo de cobra?

EE4: Não, isso daí ela não me contou não.

G: Nem como pode ter acontecido por exemplo a gravidez da cobra?

EE4: Ela também não me falou isso não, ela disse que, ela menstruava e deixou a roupa dela, num sabe como que foi, se o bicho passou lá ne, aí ela engravidou. Mas, só que essa gravidez dela, ela foi adoecendo, foi adoecendo, adoecendo, ficou bem magrinha mesmo, era pra morrer mermo, ela disse que ela foi pro curador ne, aí o curador falou pra ela que ela tava gravida de bicho. Aí ele ensinou um remédio pra ela, ele fez o remédio e ela tomou, aí começou a agoniar ela..., aí teve. Teve primeiro uma, aí depois ela teve outra. Aí ela disse que não mandou matar não, mandou soltar. Quase ela morre, Até hoje ela conta essas histórias dela, aí ela ainda tá viva até hoje ela. Contava, contava não, me conta até hoje.

G: Quando aconteceu ela era solteira ou já tinha marido?

EE4: não, já tinha marido já.

[00:03:24]

G: E..., além dessa história a senhora sabe alguma outra?

EE4: Olha..., de cobra? De cobra que eu sei que ela me falou, só essa daí né, agora de outras, de outros bichos assim....

G: Tem bastante!!!

EE4: Tem bastante.

[00:03:45]

G: É, e a senhora já teve alguma experiência com cobra aqui na aldeia? A senhora teve contato com alguma cobra, já viveu algum momento com cobra?

EE4: Não, não, não.

G: Nunca encontrou nenhuma cobra?

EE4: A si eu já encontrei?

G: se já teve uma experiencia com uma cobra?

EE4: assim, de eu topar ela? E já, vixe mana, até anteontem, ela mordeu meu cachorro, mordeu dois cachorro meu, tão tudo aí bem inchado, ai eu o menino, nós tava na roça tirando mandioca, a garotinha foram pra lá.

Vovó o cachorro tá no buraco do pau, aí eu disse: bem paca. Foi pra lá, deixa que eu vou lá, foi pra lá, mana ela meteu a cabeça, pulou no cachorro, o cachorro saiu, mordeu no nariz, ai o outro já tinha mordido na mão, saindo aquele sangue, ai meu Deus, é cobra! Aí eu chamei meu filho, ele veio, nós procuramos, procuramos, até que nós achamos ela, tava lá, ai matemos, dai eu disse tora a cabeça e enterra. Me dá essa cobra e vamos embora que eu vou fazer já um remédio pra ela. Peguei aquela cobra, torei ela no meio, cortei bem miudinho assim e misturei com farinha, fiz aquele pirão e dei pra eles. Tá desinchando, muito bom esse remédio.

G: No cachorro?

EE4: É no cachorro.

[00:05:11]

G: o quê que a senhora usou?

EE4: A carne dela mermo.

G: Da própria cobra?

EE4: Da própria cobra, aí eu cortei aquele pedaço, parti, amarrei, botei em cima, aquele sangue, desceu, desceu tudinho pra mão, e desceu do nariz, desceu tudo pra boca do cachorro, ficou roxo, roxo, roxo a boca dele, mas eles não morreram não.

G: A senhora já fez isso com alguém, com uma pessoa ?

EE4: Não.

[00:05:35]

G: Nunca cuidou assim quando teve um acidente com cobra?

EE4: Não, a gente só sabe né, porque quando acontece isso, so acontece longe da gente.

G: Só sabe que aconteceu a picada, a senhora não sabe o que fazem quando, por exemplo, alguém é mordido por ela?

EE4: Olha, uma vez a gente tratou um garotinho assim, eu com meu esposo né, a gente. A cobra mordeu ele, e como meu pai era assim, entendia um pouco dessas coisas, ele disse: minha filha, tu pega a castanha , ai, tu pila a castanha, com toda a casca, faz só lavar e ai tu pila, tira aquele sumo, bem grosso e tu dá pra pessoa tomar. Ai tu pega aquele bagaço, amorna e bota em cima, ai não acontece nada não. Aí o garotinho gritava, gritava, gritava, ai eu disse pra ele: vai lá, que eu não vou não, que eu tenho medo de olhar essas coisas assim. Ai ele foi pra lá e ensinou, fizeram. Pois num é que o garotinho ficou bem, ate hoje, ele é até pai de família. Não aconteceu nada não, nem ficou aleijado, porque a cobra deixa aleijado.

[00:06:49]

G: Também não foi para o hospital?

EE4: Não, em casa mesmo, meu pai só curava assim, mordido de cobra só em casa mesmo. Não iam não, quando ele curava ficavam bom mesmo.

G: Seu pai ainda é vivo?

EE4: Não,nem meu pai e nem minha mãe.

G: E seu esposo, ele também cura?

EE4: Não.

G: Ele só lhe ajudou nesse caso?

EE4: Ele só me ajudou nesse caso ai.

G: Entendi. Eles nunca foram mordidos?

EE4: Não, graças a Deus. Só uma vez, uma filha minha, a caçula, que mordeu no dedo dela, mas aqui mesmo, ela estava andando por ali

G: No dedo do pé?

EE4: É, no dedo do pé, na perna, saiu mordendo pela perna dela.

G: Onde ela conseguiu morder.

EE4: saiu arranhando todinha a perna dela, mas ela ainda foi pra Nova Olinda ainda, Deus o livre, mana eu me desesperava aqui em casa, huuuumm. Deus me livre, chorava que só, e levaram ela pra Nova Olinda.

[00:07:49]

G: E, e, as cobras assim, a cobra em particular ela tem um significado pra senhora?

Por exemplo assim, a, a, o seu esposo tem um significado pra senhora né? Qual significado?

É o amor!

EE4: É o amor!

G: E a cobra, qual o significado?

EE4: A mana, o significado da cobra que eu tenho é, é a raiva que eu tenho dela, se eu ver eu mato, mato mermo, como diz a historia, num tenho medo dela não. Se eu achar com o que matar, bato mermo, e eu mato ela, os meninos acham graça de mim. Se a senhora ver uma cobra, a senhora mata. Eu mato mermo, um dia desses tinha uma no caminho da nossa roça, eu vinha de lá do mato, ai, ainda bem que vinha toda embotada né, que eu ando por ai, mas eu ando de bota, de calça comprida, quando eu vi, pulou pertinho de mim, quando eu olhei assim, não era uma? E ala ficou prontinha pra me morder já, pera lá, corri la no pau, cortei, bati nela e matei. Ai, eu fui e tirei o couro. Eu tiro tudinho o couro da cobra que mato, eu tiro tudinho. Eu tenho ate ai da que mordeu o cachorro..

G: A senhora tem ai eles? A senhora me deixar bater uma foto tá? (RISOS) (9:02)

EE4:(RISOS)

G: (RISOS)

EE4: Menina tem medo? Tenho é nada, porque ela mordi a gente e tem que matar ela (hãã), comigo eu fico é com raiva quando vejo cobra.

[00:09:16]

G: E, e a senhora acha que a cobra tem alguma utilidade, alguma serventia pra senhora? Ela serve pra alguma coisa do seu dia a dia a cobra?

EE4: Pra mim ela serve.

G: Para que ela serve?

EE4: olha, quando a gente, de vez em quando acontece comigo, uma dor de cabeça, alguma coisa, eu tiro um pedaço do couro dela e faço defumação na minha cabeça.

G: A senhora defuma a cabeça com o couro da cobra?

EE4: Com o couro, com o osso dela mermo.

G: E qual benefício a senhora acredita que a defumação trás?

EE4: Não sei se é porque a gente já tem aquela coisa né, de acreditar nas coisas que a gente faz né, e acaba ficando bom.

[00:10:09]

G: Mas tem algo assim, específico que a senhora pega, o couro da cobra? Por exemplo..., alguém tá com diarreia, ai eu vou lá, pego o couro dá cobra, alguma coisa especifica? Quando a pessoa tá se sentindo mal, mal-estar? Ai a senhora vai lá e defuma.

EE4: Mas eu faço só pra mim....

G: Só pra senhora? Pra ninguém mais? A ta, mas é quando a senhora se sente mal?

EE4:É quando eu me sinto mal.

G: Ai a senhora pega e faz a defumação no corpo. É como se fosse uma limpeza?

EE4: É, a pessoa quando vai defumar a cabeça não abri o olho, por que a cobra uma hora faz o bem, outra hora ela pode fazer o mau pra vista.

G: Huum, entendi!

EE4: A senhora entendeu?

G: A senhora

EE4: Por isso que eu tenho medo de fazer pra qualquer pessoa assim, né?

G: E dá algum erro, por exemplo ...

EE4: Por isso que eu só faço pra mim mermo, não tem jeito não...

[00:11:04]

G: Éééé, quais os tipos de cobra que a senhora conhece?

EE4: eu conheço a surucucu, tem a surucucu de fogo, surucucurana, pico de jaca, e tem a jiboia, tem uma cobrinha que é fininha, igual um galinho de pau, ela é bem compridinha ela é, bem magrinha, éééé´....

G: cobra cipó.

EE4: É cobra cipó mermo, que ela é igual um cipómermo.

G: É a surucucurana a pico de fogo é?

EE4: De jaca.

G: De jaca e a senhora falou uma de fogo, qual foi?

EE4: Surucucu de fogo, é uma vermelhinha que tem.

G: huuuumm.

[00:11:51]

G: E dessas cobras que a senhora me falou qual delas que a senhora considera mais perigosa?

EE4: Eu acho assim, que é a surucucurana e a surucucu pico de jaca, por que a pico de jaca ela deixa a pessoa aleijada, né? É como eu tô falando, se a pessoa fazer logo esse remédio, assim como eu fez pros meus cachorrinhos, num fica não, num fica aleijada não, mas se ela não se cuidar direitinho fica aleijado, porque eu já vi muitas pessoas aleijada, assim aleijada. E eu acredito que a pessoa num fica não, olha por que tem um senhor ali no laguinho, que ele disse que a cobra mordeu ele e ele fez a mesma coisa que eu fiz pro cachorro, ele não ficou não, ele ta bonzinho.

[00:12:36]

G: E, e foi alguém que ensinou pra senhora ou a senhora é?

EE4: Foi ele que me ensinou, ai eu teve, fez o teste.

G: Huuum... Teve a experiência ne?

EE4: Uhum. E agora, eu teve, por que tudo que me ensina, diz a historia, eu faço o teste pra mim acreditar se é o que ele falou mesmo, e eu acreditei que é.

[00:13:00]

G: E a senhora fala pouco Munduruku, sabe o nome de alguma dessas cobras em munduruku?

EE4: Não, não, náo sei não.

G: Não.

G: E, a senhora é, se tivesse de fazer algum cuidado com alguma pessoa que foi picada por cobra, qual cuidado a senhora faria? Se fosse um cuidado domiciliar?

EE4: Assim, pra cuidar da pessoa?

G: Por exemplo, alguém aqui fosse picado, agora de imediato, a senhora faria algum cuidado ?

EE4: Eu ia fazer a mesma coisa que eu fiz com meu cachorro.

G: Então me fale como a senhora faria, assim por exemplo, me fale passo a passo de como a senhora faria? Pegaria o couro da cobra, colocaria no fogo, queria que a senhora me contasse detalhes, como a senhora faria?

EE4: É, eu ia cortar aquele rabo da cobra, por acaso fosse pra senhora, que DEUS zulivre, ia tirar um pedaço da cobra, do rabinho dela e ia dá pra senhora engolir. Ai, um pedaço, a cabeça eu ia cortar e botar em cima da cisura, ai deixar ali, porque ali ele puxa tudo o veneno, o veneno. Foi o que aconteceu com meus bichinhos, desceu todinho aquele veneno, ficou roxo, roxo, mas não morreram.

[00:14:26]

G: E quanto tempo a senhora acha que levaria para fazer, entre preparar e aplicar na pessoa?

EE4: ....., mana, agora esse dai, acho que não dura muito tempo não, por que, olha eu fez anteontem, e hoje meus cachorros tão tudo bem. Eu tiro por eles, já tão todo desinchado, já tão, tavão até latindo hoje.

G: O tempo que a senhora levou preparando, por exemplo: no fogo, é cortando, leva muito tempo, uma hora, trinta minutos?

EE4: Eu acho que uns 30 minutos, no que chega, faz logo. Cortou, faz logo e coloca logo em cima, por que tá ali, tá tudo com veneno ainda, ne! Se por acaso modi no mato, se matou a cobra, pega logo um pedacinho e engoli logo o pedacinho lá.

G: Nesse caso, esse cuidado que a senhora faz, a senhora herdou de um amigo, de outro lugar?

EE4: Foi

G: que tinha feito com outra pessoa?

EE4: Foi com ele mesmo, com ele mesmo, ele falou tudo pra mim. Aí fiquei pensando, eu vou fazer, o dia que Deus zulivre acontecer uma coisa dessa, eu vou fazer. Ai como aconteceu com meus bichinhos ne, ai eu disse, a eu vou fazer, vou fazer mermo. Aí eu peguei lá na casa do forno, cortei aquele toro da cobra, miudei, aquilo bem miudinho, assim batidinho, picadinho mermo, misturei com um pouco de sal e farinha, e botei pra eles comerem. Um pedaço assim ,cortei e botei na mão dele e no fucinho do outro. Mana, escorria aquele sangue, aquela água, aquele sangue assim, num fechou não, pronto. E agora eles tão bem, num tem nenhum deles aqui

[00:16:13]

G: É, quanto é, quanto tempo mais ou menos a senhora, a senhora já tem o couro da cobra, e se não tivesse ele, o que a senhora faria?

EE4:Bom, ai eu já ia com a castanha com ele...

G: Como a senhora faria com a castanha?

EE4: Não, a castanha a gente pega, a gente pila com toda casca, faz aquele sumo bem grosso, um leite e dá ´pra pessoa tomar, e aquele bagaço, a gente amarra em cima, com um pano, alguma coisa assim, e deixa lá, ai so cuida com aquilo mesmo.

G: A senhora já usou alguma parte do couro também pra amarrar ou algo assim?

EE4: Não.

G: Mas, usaria?

EE4: Usaria a carne com tudo, no que mata né, bota em cima e deixa lá, que aquele sangue vai sair, ai pronto, engoliu, o veneno já vai coisando por dentro de tudo.

[00:17:24]

G: Se a senhora fizesse essa pratica, a senhora faria só a pratica é, que a senhora conhecia ou ia associar a prática por exemplo lá do polo?

EE4: Pra mim cuidar né? Se fosse so pra mim cuidar?

G: Só a senhora cuidaria, se fosse de imediato ou a senhora passaria e avisaria a equipe de saúde?

[00:18:04]

EE4: Eles não deixam, não deixam mais como era antes, antes a gente só cuidava assim. Mordeu e já querem levar pro polo, a gente não pode nem fazer.

G: Mas assim ainda tem pessoas que conseguem fazer ?

EE4: Tem.

G: Em algum momento, ou antes ou depois, mas a senhora faria?

EE4: Eu faria.

G: Associado, as duas coisas, tanto aqui quanto lá?

EE4: É

G: Mais ou menos a pessoa que faria aqui com a senhora, se ela fizesse, ela teria um tempo de acesso pra Nova Olinda, mais ou menos de duas horas, três horas, de lá pra cá é mais ou menos isso né? Por que ainda vão acionar a equipe, levaria quanto tempo?

EE4: Acho que não demora, porque mordida de cobra, eles dão andamento, rapidinho logo..

G: É de imediato? Só o tempo de no máximo duas a três horas...

EE4:Imediato mermo, é mais ou menos.

[00:19:08]

G: E esse material de cobra a senhora encontra aqui por perto?

EE4: Humrum.

G: Eu queria ver esses que a senhora, posso ver?

EE4: Eu vou pegar.

**ENTREVISTA 5**

**Caracterização do Entrevistado**

**Idade:** 70 anos

**Especialidade:** Pajé

**Informações Relevantes:**

**a)** Na entrevista estavam presentes a entrevistada e a guia da aldeia.

**b)** Na entrevista informou atuar como parteira há mais de 40 anos, e como curadora e pajé desde muito jovem não sabendo informar a data. Em seu relato ela informou também ter recebido o dom da pajelança proveniente de Deus.

**e)** Entre os recursos utilizados da própria serpente, ela informou já ter utilizado a banha da surucucu para remédio contra judiaria e em acidentes ofídicos.

**d)** Seus relatos também mencionaram algumas restrições e condições de resguardo durante o cuidado com pessoas mordidas por serpentes.

**Transcrição da entrevista**

G: (...) eu vou começar com a primeira pergunta, tá? me conte se existem histórias, lendas, mitos, contos com cobras em seu povo?

EE5: Sim.

G: Sim. A senhora pode me contar?

EE5: Pode. Como dessas cobras que a senhora tava falando ainda agora, das grandes, existe.

G: existe?

EE5: existe.

G: E a senhora conhece alguma história?

EE5: Eu não conheço bem essas história não, mas por causo que num me dediquei assim prestar atenção nas ... nas história que cuntavam primeiro mas eu ouvi muito falar que tem mermo essas cobras, grandes têm.

G: O que que as pessoas falam, assim que a senhora já ouviu?

EE5: Eles falam assim que a cobra grande ela bóia, ela ... só que ela é perigosa, aí o pessoal fica com medo e num vai nem lá perto, aí eles ficam de longe só olhando elas quando bóia, até ela sentar pro fundo de novo aí que eles pode viajar porque durante ela tá boiada eles não viajam não.

G: E, se caso assim alguém viajar quando ela tiver boiada o que acontece?

EE5: Aí eu acho que aí eles ficam com medo que pode vim ingolir eles né? Porque ela tá boiada ali.

G: A senhora conhece alguma outra história relacionada a cobra?

EE5: De terra? História assim de cobra assim de terra até que não tem não mas muitas vezes acontece que a cobra morde né as pessoa, as pessoa vem para casa fazem remédio para não inflamar para não doer muito né, daí mandam para cidade... essa história, história não, isso daí é uma verdade mesmo por que aqui a gente já tem tratado né assim de pessoa, aí cumo a senhora tava falando por causo que ... a cobra morde .. na, a pessoa e não pode ter muita gente ali muito olhando porque tem muitos que eles tenham olho doído né e a pessoa grita muito com a dor, então a gente separa o paciente e aí faz o remédio, defuma cum o coro da cobra mermo, que morde, aí passa a banha da mesma cobra, em cima, para não doer muito enquanto a gente, até chegar no médico para tomar injeção para ficar bom.

G: A senhora já fez esse tipo de cuidado?

EE5: Esse daí a gente já fez já aqui.

G: É, e a senhora já fez muitas vezes?

EE5: A gente já tem feito sempre aqui ...

G: (...) a senhora é, as cobras elas possuem algum significado especial pra senhora?

EE5: .humm.. muitos é, mas muitos não é não, porque elas meio valente, é venenosa né, tem umas que ela é, é mais, é mansa, a gente pode até agarrar mas muitas não. Perigosas essas que morde a gente mas a como a jiboia chamada até a gente cria ela, ela se amansa.

G: A senhora já teve alguma experiencia com cobra aqui na aldeia?

EE5: Aqui só o meu genro que sempre ele gostava de criar, de agarrar essas cobras, eu não que eu tenho é medo, não agarrava não, mas ele sempre agarrava e colocava la na casa dele e agora não sei se ele ainda tem, sempre ele tinha.

G: Mas, ele agarrava e fazia o quê com a cobra?

EE5: Ele prendia a bichinha. Prendia assim na caixa, alimentava ela.

00.05.09 G: Mas, ela tinha alguma serventia?

EE5: Serventia, esse tipo de cobra elas tenho mas só que eles num sabe né para quê. Ele só amansa a bichinha só para viver em casa mesmo mas ele não sabia que significativo ela tinha

G: Mas, a senhora sabia?

EE5: … A gente sabe porque a banha dela é remédio né para muitas coisas para própria mordida de outra cobra ela é boa.

G: Quais tipos assim de remédio a senhora usaria, as partes da cobra?

EE5: ... A banha dela, a banha, o couro, que se outra cobra morder a gente pega tira o couro da jiboia né e faz uma defumação, a banha memo.

G: a senhora acha que as cobras, elas possuem uma utilidade no seu dia-a-dia?

EE5: Eu acho que sim porque elas, as bichinhas as vez elas venham eu tenho até pena de matar os bichos (risos).

G: Então, elas tem serventia?

EE5: Tem.

G: É. Se matam uma cobra ela vai servir para quê para a senhora?

EE5: Ela, se matar a cobra, ela já vai para assim, ela já vai dar falta a modo assim para outra. Porque eu sou muito coisa assim por bichos, sabe eu não gosto de matar os bichos não.

00.07.00 G: Aqui na etnia munduruku a cobra, ela tem algum significado?

EE5: Ah, olha na verdade, aqui mermo, o pessoal quase eles não tem coisas com esses tipos de cobra, com esses tipos de remédio que a cobra serve para tudo né, e eles ..

G: Humrum

EE5: E eles, acho que não sabem não, tem muitos que sabe que é bom, é pá remédio e que a banha da cobra serve para tudo, para doença, ela é contra doença no meu conhecimento agora, no conhecimento de outros eu não sei, pelo que eu estudei um pouco.

G: É, é a senhora como especialista né, como pajé a senhora usaria ou usa a cobra, ou alguma parte dela em algum ritual?

EE5: A, a gente usa a ... e a banha assim para remédio mesmo assim.

G: Que tipo de remédio por exemplo?

EE5: Até para curar uma enfermidade a banha dele serve, para golpe, é para mulher quando ela é operada, isso é bom.

G: Na parte espiritual assim, existe algum ritual feito com a cobra?

EE5: Tem, com certeza. Só que por assim, por aqui, aqui ainda não chegou causa ... de ... as pessoas assim para gente curar com esses tipos de remédios mas tem.

G: Ela serve para judiaria ou algo do tipo?

EE5: A mamãe dizia assim: a surucucu serve para remédio contra judiaria, tem muito significado da cobra (risos).

G: É. A senhora pode falar deles para mim?

EE5: Ela serve sim ... tem gente que judia dos outros e a gente pode fazer remédio para pessoa que é judiada né, aí pode curar, tratar com o mermo as coisas da cobra, com a banha, mas se for, vamo dizer a cobra que morde né a gente, pode fazer, o remédio.

G: é, quais os tipos de cobra que a senhora conhece?

EE5: Que a gente pode fazer o remédio dessas daí é só dessas surucucu.

G: Mais além, a surucucu a senhora conhece alguma assim no dia-a-dia?

EE5: Todo dia não...

00.06.12 G: no dia-a-dia quais os tipos de cobra a senhora conhece?

EEF5: Tem diversos tipos de cobra.

G: Cite o nome delas para mim?

EE5: Eu acho que é, tem jiboia, tem aquelas outras cobra papo amarelo, sei lá como ela não é, eu não sei o nome, tem jararaca, tem cotimboia.

G: A senhora já viu todas elas?

EE5: I já.

G: Já?

EE5: A gente vê entre elas

G: E dessas cobras que a senhora me falou qual delas a senhora considera mais perigosa?

EE5: Só é a surucucu, só ela é a mais perigosa. Mas as outra não são não.

G: A senhora sabe se aqui as cobras possuem algum nome em linguagem munduruku?

EE5: Sim.

G: A senhora sabe falar como chamam a cobra?

EE5: Poibúte.

G: Poibú?

EE5: Poibúte.

G: Poibúte.

EE5: É (risos).

G: E a surucucu ela tem algum nome especifico?

EE5: É mermo assim.

G: Mesmo poibúte.

EE5: É as cobras né (risos).

G: sim. A senhora realiza algum cuidado em pessoas envenenadas por serpentes?

EE5: Algumas vezes.

G: Quantas vezes, a senhora sabe mais ou menos que a senhora já cuidou de pessoas que foram mordidas?

EE5: Assim mais ou menos umas duas vezes só. Porque aqui é muito difícil dá, graças a Deus que aqui é muito difícil... minha filha tratar de gente doente aqui de mordida.

G: Ela já tratou a sua filha? A senhoria me relatar assim, dessas duas pessoas que a senhora cuidou, quais foram os cuidados que a senhora fez?

EE5: (Risos) agora que, deixa eu me lembrar por que já faz muitos tempos.

G: Pode lembrar, fique a vontade. Aí a senhora me diga o que a senhora fez, quais foram os cuidados e o tempo que levou para senhora preparar esse cuidado...

EE5:Hunrum... sim, mas num foi demorado assim muito não, porque a gente cuidou com a merma banha dela, da cobra.

G: Com a própria banha.

EE5: Aham, o coro a gente queima e faz a misturada com a banha e faz, tipo uma pumadinha.

G: A senhora chegou a defumar essa pessoa, ou a rezar nela?

EE5: Não, não, não.

G: E, quanto tempo a senhora fez esse cuidado nessa pessoa? Quantas vezes?

EE5: Umas duas vezes.

G:Umas duas vezes? Tinha um dia ou algum horário específico para fazer?

EE5: Tinha.

G: Quais eram os dias assim?

EE5: É porque, assim, as vezes era segunda e terça.

G: Segunda e terça feira.

EE5: Hunrum. Que a gente faz.

G: Esse cuidado que a senhora fez a senhora herdou de alguém da sua família ou de algum especialista aqui da etnia que alguém lhe ensinou?

EEF5: Olha, agora que, fazer como o mora, agora que a história vai ser... porque ninguém me ensino, ninguém me ensinou e eu aprendi fazer todo esses tipos de coisa, da memória, de (...) é Deus que deu esse, essas coisas, eu acho porque a gente saber fazer um remédio assim né.

G: Mas, tinha alguém da sua família antes da senhora que cuidava das pessoas?

EE5: Não, eu nunca tinha vista ninguém cuidar de ninguém por aqui assim, desse tipo.

G: Entendi. É, a senhora poderia me relatar, se esse cuidado, em qual momento ele é feito, por exemplo: ele é feito logo que acontece a mordida, ou é após a mordida?

EE5: Vishi, essas coisas aí é, assim é, tem por exemplo que a pessoa fica ferido assim a gente tem que cuidar logo porque a gente não pode passar dois, três dias para cuidar.

G: Então é imediato?

E5: É.

G: É ... mas a senhora quando faz esse cuidado, a senhora faz esse cuidado sozinha? com alguém? ou a senhora faz esse cuidado associado ao cuidado médico?

EE5: Não. As vezes só eu, porque o médico aqui é difícil vim né. (conversas no fundo)

G: Então o cuidado ele é sozinho,né?

EE5: Haram.

G: Dos cuidados que a senhora fez né, houve alguma melhora?

EE5: Sim.

G: E quanto tempo?

EE5: Dois dias mais ou menos ele já tava tudo bem.

G: Mas, ele também ainda foi para atendimento médico?

EE5: Não.

G: Não.

EE5: Não, não. Não foi preciso ele ir.

G: Não foi preciso né.

EE5: Não foi não.

G: Os recursos que a senhora utilizou nessa prática, eles são encontrados com facilidade?

EE5: Sim.

G: Sim? Eles são fáceis de encontrar?

EE5: As vez é fácil.

G: Mas, quando a senhora precisou fazer, a senhora é, tinha em casa?

EE5: O quê, os remédios? Tinha.

G: Quanto tempo mais ou menos a senhora realizou esse cuidado?

EE5: Uns três dias.

G: É?

EE5: Hunrum.

G: E, entre esses recursos que a senhora presenciou o que a senhora usou da própria serpente, a senhora usou algo da própria serpente? Pra essas pessoas que a senhora cuidou?

EE5: Não, eu usei é... remédio do mato.

G: Por exemplo o quê?

EE5: É uns mato que é igual mermo uma cobra, a gente pega ele e bate, tira o sumo e dá pra pessoa cuidar só com aquele lá, e cuidar com a própria banha também né, com coro e tudo.

G: E quanto tempo a senhora deu para pessoa esses matos?

EE5: Umas três vezes.

G:Umas três vezes? Quantas vez no dia a senhora dava?

EE5: duas

G: Duas vezes? E teve melhora em quanto tempo?

EE5: Uns três dias mais ou menos ele tava quase bom já.

G: E a senhora usou assim só esse mato específico, ou tinha alguma outra planta que senhora usou?

EE5: É porque a gente usa com outros tipos, né, de mato, de remédio assim. Tanto da farmácia quanto da gente né que a gente faz.

G: Mas, a senhora lembra o nome dos outros remédios que a senhora usou?

EE5: Humm... eu não me lembro mais é do nome do mato (risos). Do mato que é o negócio.

G: E se acontecesse hoje aqui assim por exemplo né, a senhora tivesse é na roça e tivesse que usar um recurso que a senhora tem aqui, como que a senhora cuidaria dessa pessoa?

EE5: Eu acho que cuidaria com o que tivesse aqui perto né no mato, e tem as vezes tem muito mato né?

G: Mas, qual mato a senhora usaria?

EE5: Bom, esses daí tem, tem uns mato que é igual mermo uma cobra sabe, ele da assim, ele é igualzinho, a gente pega ele, tosse ele e tira só o sumo e distempera e dá com outro remédio para pessoa tomar.

G: E, mandaria para Nova Olinda também para atendimento médico ou ficaria só no cuidado natural?

EE5: Só se a pessoa quisesse ir né, mermo. Porque aqui a gente já tratemos mermo assim, sem ir para Nova Olinda.

G: Tratou somente aqui né?

EE5: É.

G: Sem precisar ir, mesmo quando são casos de mordidas da cobra mais perigosa que a senhora considera?

EE5: Só que a cobra mais perigosa que a gente considera aqui é, agora quando é dessas daí é preciso ir para Nova Olinda mermo né, porque muitas vezes para um o remédio que a gente faz aqui, para uns serve para outros já não serve, porque a cobra não é só duma qualidade né.

G: Então, desses que a senhora fez, não foi dessas mais perigosas, no caso, mas a senhora sabe qual foi o tipo de cobra?

EE5: Eu não sei nem como dá o nome daquela cobra.

G: E foi alguém da sua família que a senhora cuidou?

EE5: Não, foi de outra família. Essas famílias não moram mais nem por aqui mais, já fora embora tudo.

G: Já tem um tempo né?

EE5: é, já tem um tempo já.

G: A senhora tem algo mais assim que a senhora queira me relatar, que a senhora queira acrescentar na sua entrevista? Algo que a senhora considera importante relacionado a cobra que a senhora não tenha me dito ou que eu não tenha perguntado?

EE5: Eu acho que, sobre esse daí que eu cuidei, foi só esses mermo.

G: Dessas pessoas que a senhora cuidou tinha alguma restrição, algo que a pessoa não podia fazer durante o tratamento?

EE5: Não.

G: Algum resguardo?

EE5:Tinha não. Por causo que naqueles tempo as pessoas sabiam como era, agora não.

G: Naquela época as pessoas sabiam como se resguardar no caso. E, como se resguardaria uma pessoa mordida de cobra? Qual seria sua orientação?

EE5: (Risos)

G: O que ela não pode fazer, por exemplo?

EE5: Ela não pode fazer, é porque eles não pode tarem no meio assim das pessoas, é porque tem muita gente que falo assim que o olho é venenoso, até a voz da pessoa quando fala, então, os cuidados era isso, por caso que a pessoa vai falar, a pessoa grita com dor, é capaz de matar a pessoa. É por isso que tem esses negócios de, tem que resguardar, a pessoa não vai ficar lá perto, por que cobra é assim, quando ela morder se tiver uma pessoa que for, que tem o olho venenoso é capaz de até matar a pessoa, então os cuidados da gente é aí, fazer remédio e resguardar a pessoa. Não deixar ficar andando muito assim durante o tratamento, durante ele tá, ele ainda tá doente.

G: Ta bom então (...) muito obrigada pela sua participação.

EE5: Precisando eu tô por aqui. (risos)

G: muito obrigada.

**ENTREVSTA 6**

**Caracterização do Entrevistado**

**Idade:** 44 anos

**Especialidade:** Pajé

**Informações Relevantes:**

**a)** A entrevistada se considera pajé por consagração e coroação e atua com a linha branca, vermelha e preta.

**b)** Informou que para atuar como pajé também foi batizada nas águas da aldeia e anteriormente participava do Centro São Jorge.

**e)** Em sua entrevista ela relatou receber orientações de seus mestres (guias) e que a banha da cobra serve para remédios e para judiaria (feitiço).

**Transcrição da Entrevista**

[00:00:01]

G:Me conte se existem histórias, lendas, mitos, contos com cobra no seu povo

EE6: É.… Começar da branca logo, né…. É… no caso, assim que eu sei, ela era uma moça, bonita também, como a minha mãe fala. E, a mamãe falou que ela sentia muita dor de cabeça… aí quando foi um dia a mãe dela pediu que o curador viesse pra cuidar dela, e ele veio. Aí o curandor ensinou um banho, ela fez o banho, tomou o banho, banhou-se, aí melhorou-se da cabeça. Ai minha mãe falou, que continuou de novo, ela não comia e não fazia nada, só queria está dormindo, hora de meio dia ela dormia bastante. Quando chegava seis horas ela não queria mais saber de nada. Aí minha mãe falou que ela sentia muita dor de cabeça. Aí quando foi um dia passado, aí, ela tava… ela tava muito doente. Aí ela ficou boa, do nada assim ficou boa. Aí Ela não tinha mais bem dizer, muito assim…ela não tinha mais sangue nos coisas dela né. Aí quando foi um dia ela disse que queria tomar banho na beira. Aí ela desceu pra beira… convidou a irmãzinha dela pra descer com ela, pra acompanhar ela até a beira do rio. Aí ela foi acompanhar ela na beira do rio, aí quando ela chegou lá, ela tirou os dentes dela, colocou dentro de um copo, e deixou em cima da ponte Aí ela falou pra irmãzinha dela agora me esperar aí que vou dar um mergulho, já eu volto, eu te pego pra ti dá banho. Aí quando ela caiu nágua, só viram um rebojo… ela já tinha ido. Aí passado, aí sumiu. Aí foi aquele alarme danado, a minha filha sumiu… aí tá… tava a a mãe dela lá… parece que ela levou uns quatro dias, ou cinco dias a mãe dela pra dar uma volta, pra mostrar onde que ela tava, no caso ela tava encantada.

G: Mas a mãe dela soube de que forma?

EE6: Veio num… subiu parece que num… ela, deixo ver, pra mim não falar errado… parece que ela levou, foi a tia dela, não foi a mãe dela. Levou a tia dela… não foi a irmã dela né, sim, foi a irmã dela, que levou pra passear né, veio buscar ela do nada, tipo assim, levou ela, só o espírito, deixou o corpo. Aí é la passou uns quatro dias andando com ela.

G: Ela veio em sonho ou ela veio assim… ela incorporou? Como foi?

EE6: Primeiro ela veio em né, depois ela levou ela, desmaiou ela, levou ela, só o espírito dela no caso. Aí ficou lá, deitado. Aí mandaram chamar o pajé…. O pajé veio e falou que ela que tinha levado ela, pra mostrar onde ela tava… quando ela voltou ao normal ela falou mesmo. Trouxeram ela de volta, ela não queria vir mais… só que ela não podia comer comida nenhuma.

G: A irmã dela não queria vir mais?

EE6: Uhum… porque ela achou bonito, só que ela tava amamentando, tinha um bebezinho que mamava, aí não pode ficar.

G: Ela achou bonito assim, era… como era lá, era?

EE6: Era uma cidade, no fundo é uma cidade.

G: Uma cidade?

EE6: Isso... Quando chega lá, tem jardim pra tudo quanto é lado. Você olha pra cima, você tá vendo tipo um espelho, entendeu. Eu sei porque eu já foi lá.

G: A senhora já foi lá?

EE6: Já…

G: É?

EE6: Quando meus espíritos… quando meus mestres vem me buscar, vou pra lá. Dela tô vendo tudinho que tá acontecendo aqui. Mas eu não tô sabendo quê que meu corpo tá fazendo com outra pessoa dentro. Entendeu? Já depois que vão me explicar tudo. Comigo isso que acontece.

G: A senhora já visitou, então o fundo?

EE6: Já! Já visitei… o incorporamento na hora que eles chegam comigo, eu vou. Foi isso daí que sei dela. No momento já não posso uma coisa que eu não sei mais, entendeu?

G: E quando a senhora vai assim, pro fundo, pra visitar a cidade do fundo, a senhora é… tem alguma permissão?

EE6: Tenho!

G: Pra ir?

EE4: Tenho…

G: É?

EE4: A permissão de um caboco. Ele só vai permitir se eu for, no caso falarem com ele, se não falarem com ele, aí não vou poder ir.

G: É? Mas qual seria esse caboco?

EE6: Seria o chefe lá da porteira lá, da onde eles estão protegendo lá… Tipo assim, tem os seguranças sabe, como tem aqui, a gente tem, eles tem também la. Aí no caso, se… eu chego lá… ele vai chegar lá, poxa eu quero levar ela, pra fazer… que ela quer fazer um trabalho, preciso… eu tô precisando do corpo dela agora… aí ele vai… o meu caboco já tá lá, já fala com ele, ele vem, conversa comigo, aí eu já vou… eu já vou ficar preparada esperando ele ali.

[00:05:01]

G: E a senhora… pra senhora, é, ser peje né, ou fazer as curas que a senhora faz… a senhora teve algum preparo?

EE6: Tenho

G: É? Como foi?

EE6: O preparo de… pra remédios?

G: É! Porque assim, a senhora falou pra mim, que a senhora é… na pajelança a senhora tem algumas linhas né?

EE6: Sim!

G: É… a senhora teve um preparo pra… pra ser consagrada pajé? Como foi? Teve algum batismo?

EE6: Teve, é… o batismo

G: E o que mais? Como foi? Me conte…

EE6: O batismo foi… Ele… a gente vai né, aí eles vão batizar a gente na água, entendeu? Aí eles vão fazer o coroamento, que falam… aí a gente vai chegar em terra, aí estão tudo lá. Aí a gente vai ficar na roda ali, pra chamar…

G: Os mestres ficam ao redor?

EE6: É… não… os pajés que vão fazer o trabalho, no caso como nós tamos aqui… eu vou ficar bem no meio, eu já tô coroada ali. Aí o quê que eles vão fazer… vai fazer aquele ritual lá, pra chamar o caboco que vai ser o cabeça de linha, que é o meu né. Que no meu caso, o meu cabeça de linha eu sou o índio guerreiro, ele é o meu cabeça de linha. Foi ele que… que ficou que… que ficou comigo trabalhando né. É ele que comando comigo.

G: E nesse batismo, quantos pajés estavam presentes?

EE6: Tinha um bucado, né amor? Eu acho que tinha uns dezessete…

G: Uns dezessete pajés?

EE6: Por aí… ou mais.

G: E no… mas foi aqui mesmo?

EE6: Foi ali no, ali na onde tem o… vocês não foram naquele centro ali?

G: Espirita?

EE6: Uhum… pois é, se batizemo… o meu batizado foi lá.

G: E quando a senhora se batizou, é como um batismo que a gente faz assim, tradicional? Tem padrinho ou algo do tipo?

EE6: Tem! Padrinho de terra como de fundo. São dois de terra e dois da água.

G: Quem são seus padrinhos da água?

EE6: O meu é Honorato e a caboca, é aquela assim, é a Mariana. Cabo a Mariana.

G: Caboca Mariana?

EE6: Uhum…

G: É… eles eram é… humanos ou eles é… sempre foram, é… do fundo?

EE6: Sempre foram do fundo. Agora quem é, do coisa, que é seu Honorato né, ele foi também como a gente, de terra.

G: Ele foi de terra?

EE6: Foi de terra…

G: O que aconteceu com ele?

EE6: O que aconteceu com ele… por causa que eles nasceram duas cobras, já foi nascido assim. Aí eles foram jogados… foram deixados no rio. A mãe dele teve duas cobrinhas. Eles eram irmãos. Só que no caso da irmã dele, eu esqueci o nome, mas um tempo desse a minha filha tinha uma coisa dele, eu lembrei o nome dela, porque falava lá, o nome dela, e era esse daí. Porque na época que eu conheci, minha vó falava o nome dela. Como é o nome da irmã dele? (Resposta ao fundo de seu acompanhante) isso. É caninana o nome da irmã dele.

G: Caninana?

EE4: Uhum… porque eu peguei, que disseram, foi esse nome aqui que minha vó falou. Aí quando foi um dia desse, eu tive resposta de sim, que era o nome dela. Só que ele matou ela, porque era muito ruim.

G: Ela era ruim?

EE6: Ela ataca as pessoas e ele não, ele sempre protegia né, esse daí.

G: E essa briga que ele teve foi no fundo no caso?

EE6: Foi no fundo no caso… foi no fundo, aí ele matou ela, e ficou só ele. Aí no caso, ele tá aí.

G: Ele é seu padrinho?

EE6: Ele é meu padrinho!

G: E no caso ele é também um guia seu?

EE6: Não somente… somente meu padrinho só que ele trabalha também nessas linhas aí né. No caso ele é o… ele é também o chefe de cabeça do Marcelo lá do Caialé. Ela trabalha lá com esse espírito aí. Mais sempre ele baixa aí.

G: E quando é… como pajé, a senhora tem assim, alguma restrição pra fazer alguma cura, por exemplo, tem algum momento que a senhora não pode fazer? Ou não dá no rio ou algo do tipo?

EE6: Tem alguns que eles proíbem não andar no rio. Tem alguns que fica… tem uns que fica na terra mesmo.

G: Quando a senhora não pode andar no rio, que momento a senhora não pode andar?

EE6: Quando eu tô de bandeira vermelha, que é a menstruação da gente.

G: O que acontece?

EE6: As vezes eles ficam, ficam brabo. Dá mais de a pessoa tiver fazendo coisa que, no caso, abusando entendeu? Aí eles vão ficar brabo, vão fazer coisa… vai me fazer pular, vão fazer aquelas coisas que não devem fazer.

G: A senhora já teve assim… alguma experiência assim de assim a senhora tá com a bandeira vermelha e ter que ir pro rio?

EE6: E já. Mas não abusando no caso.

G: É? Só de ter ido?

EE6: É…

G: Mas já aconteceu alguma coisa?

EE6: Não. Porque eles sabem que eu não tô abusando. Tô fazendo uma coisa que eu tenho que ir lá né, só que… só eu posso resolver.

G: Eles nunca também chegaram a descer quando a senhora foi pro rio, ou teve a presença deles em algum momento?

EE4: Só lá… de lá pra cá, naquele dia , foi sexta-feira.

G: Como foi?

EE6: Ela veio do nada. Ela foi enrolando, aí eu pesando que era cabelo, acaba que ela que tava enrolando aqui, só apareceu a cabeça dela… eu conheci ela por causo que ela colocou logo a cabeça dela por aqui, deu de entender

G: Mas quem era que tava enrolando?

EE6: Era a Janaína.

G: Janaína? Ela é o que?

EE6: Ela é uma caboca também. Só que ela vira gente e vira cobra na mesma hora.

G: Ah, ela tem o formato de cobra?

EE6: Isso, tem formato de cobra.

G: Ai a senhora sentiu ela enrolando foi… no pescoço?

EE6: Uhum… foi.

G: E o que a senhora fez?

EE6: Aí ela disse pra que, ela… eu falei pra ela, precisava vir assim?, ela disse… eu precisei vir, por causo que vem se formando um tempo, eu quero rápido você chegue do outro lado, porque eu tô no momento pra te ajudar. Porque a canoa vinha baixo né… E no caso ela tava lá.

G: Entendi… ela veio como um aviso…

EE6: Isso como aviso. E o temporal vinha perto mesmo, vinha roxo pra baixo. Cheguemos aqui trovão…

[00: 11:02]

G: E a senhora já teve alguma experiência com cobra aqui na sua aldeia?

EE6: Não!

G: Assim… a senhora já fez assim algum trabalho com cobra, alguém já foi mordido com cobra?

EE6: Não…

[00:11:12]

G: É… as cobras elas possuem alguma significado pra senhora?

EE6: As cobras tem um significado , que nem todas são da parte de Deus né?

G: Algumas não são da parte de Deus?

EE6: Algumas não são da parte de Deus…

G: É? E o que a senhora sente assim, quando ver uma cobra?

EE6: Tem umas, porque eu já passei por um momento que eu fui… apareceu pra mim umas cobras, apareceu pra mim um cachorro que era preto e ela vinha pra me fazer o mal. Isso as vezes não gosto nem de falar, nem de lembrar né. Porque ele tava já comigo quando aconteceu isso, as vezes eu gritava quando ela vinha, porque era muito feio no momento. É por isso, que eu tô falando. Nem todas as cobras são da parte de Deus.

G: E taí, era como se fosse um feitiço?

EE6: isso. Assim, eu sentia que era um mandado, que vinha né, como foi… a mulher cuidou de mim, graças a Deus. Até hoje nunca mais eu vi ela.

[00:12:12]

G: A senhora acha que as cobras possuem alguma utilidade no seu dia-a-dia?

EE6: Acho que não.

G: Elas serve pra alguma coisa?

EE6: Serve pra fazer remedio. A banha dela.

[00:12:26]

G: Que tipo de remédio?

EE6: Pra tirar feitiçaria, pra… pra fazer judiaria quando as pessoas pede o coro dela, mas no caso o coro dela… no momento no caso dela eu preciso pra tirar à judiaria, não a feitiçaria. Ela serve pra essas duas coisas.

G: Pra enfeitiçar e pra tirar?

EE6: É…

G: No caso, a senhora trabalha mais pra tirar?

EE6: Pra tirar! No caso pra fazer judiaria, eu jamais vou fazer isso.

G: A senhora já fez defumação com alguma parte de cobra?

EE6: Não!

G: Não?

EE6: Da não. No caso, somente pra tirar a judiaria né, porque os meus… os meus mestres eles não gostam dessas coisas. Porque eles falaram que não foi isso, que eles me ensinaram, pra ta judiando das pessoas. Porque todo mundo quer viver. Nem no caso que a pessoa me faça o mal, que vai me prejudicar… não é pra mim fazer isso. Ele falou que isso vai me prejudicar. Vai prejudicar tão ele, quanto eu, porque isso daí qualquer um descobre. Ele não quer isso. Porque tem pessoa que me faz mal né, me faz raiva, mas eu não faço isso. Nunca fez, pra ninguém. Somente ajudei.

[00:13:38]

G: Quis ao tipos de cobra que a senhora conhece?

EE6: Surucucu. Que ele é… tira a banha, a jiboia, também…- banha dela é muito boa pra mordida de cobra. A senhora pode passar na sua perna, e vai andar no mato que a cobra nem lhe olha.

G: Antes de eu ir caçar, por exemplo, passo na minha perna?

EE6: É… pode passar nesses negócios aqui…. Pode passar na suas pernas.

G: E aí ela não…

EE6: É… ela não vai nem lhe olhar… pode tá ate lá a cobra, mas ela vai é correr de medo. Isso daí eu também tenho experiência com ela.

G: A senhora já fez isso?

EE6: Eu nunca fez… mas eu acho que a cobra… acho que ela não gosta de mim mesmo não. Por causa que as vezes eu nem tenho assim né… tenho até a banha dela, da jiboia. Até pros cachorrinhos é bom pra eles caçarem, pra cobra não morder, é bom. E a… a banha do surucucu já é pra fazer esses tipos de remédios…

G: Entendi…

EE6: Entendeu? E quando se caso morder… lá no mato que a gente tiver junto com ele, o que vamos fazer? Vamos fazer… vamos tirar… vamos partir mediatamente a cobra, cobra surucucu, tirar aquele coisa dela, aquele couro dela, emplastrar onde está mordido. Que ele puxa todo o veneno. E as vezes não vai ser nem preciso levar lá… isso daí também…

G: Não precisa levar em Nova Olinda?

EE6: Uhum… Não precisa levar em Nova Olinda. Chega aqui em casa, vai fazer outros tipos de remédios. Já vamos cuidar dele em casa.

G: Que tipo de remédio a senhora faria?

EE6: Eu faria o sumo do, sumo do… do coisa, do… do ananás e também se eu tiver a que chamam… será que é feio eu falar? Mas é assim né… não é… como é o nome daquele tajá? Tajá buceta que a gente chama. Mas é um tajá, não é besteira tá… (risos)

As vezes eu falo assim… mas eu… pra uns é besteira, mas pra mim não é besteira, é um tajá. O nome dele é tajá buceta, ele é tipo um negocinho mesmo, e aquele lá a gente rala ele, bem raladinho, e emplastra em cima. Vai puxar todo o veneno…

G: Mas a senhora já chegou a fazer em alguém?

EE6: E já… foi no meu cachorro.

G: É?

EE6: Eu tiro pelo meu cachorro, não foi amor? Eu fez nele… ajunta todinho mesmo. Por ali já tirei…

G: E o cachorro não morreu?

EE6: Não morreu…

G: Teve melhora?

EE6: Teve melhora… Ele só morreu porque o bicho matou ele. O catitu… a queixada parece que mordeu o pescoço dele

G: Já depois…

EE6: Já depois. Mas ele foi curado. Uhum

[00:16:03]

G: Qual dessas cobras a senhora considera a mais perigosa, das que a senhora falou?

EE6: A surucucu, que é a mesma jararaca né. Pico de jaca elas são bem venenosas.

[00:16:15]

G: E a senhora sabe falar é… cobra na língua Munduruku?

EE6: Não! Isso daí que ele queria aprender, mas não aprendi não.

[00:16:22]

G: É… a senhora herdou esse cuidado que senhora me falou, de alguém ou a senhora é… quem lhe ensinou?

EE6: Foi um mestre que me ensinou.

G: Um mestre?

EE6: Uhum… o nome dele é Dom Luiz Salve

G: Dom Luiz?

EE6: Salve…

G: Salve…

EE6: Ele é um espírito também.

G: Ele é um espírito?

EE6: Foi ele que me ensinou…

G: Ele é… desceu?

EE6: Hum?

G: Ele desceu na senhora? A senhora recebeu ele? E ele lhe ensinou?

EE6: Uhum… e me ensinou.

G: A senhora registra esses cuidados em alguma lugar assim… num caderno, alguma coisa assim.

EE6: Não. Está tudo aqui na minha memória:

G: Tudo na memória… tá… Na sua família tinha alguém que era especialista? Tem alguém que cuida das pessoas também na sua família?

EE6: Tem!

G: Tem?

EE6: Não… no caso só eu mesmo. O meu irmão como estou falando, ele é outro cuidador de outras né. Só que no momento ele também se afastou, não quis mais. Que agora ele tá na igreja lá…

G: E antes vocês cuidavam alguém, tipos os avós de vocês, os tataravô de vocês… a senhora sabe se alguém era especialista, era algum curador, alguém pajé?

EE6: Acho que sim. A parte já da minha vó parece, do meu avô… papai falava né… tinha eles.

[00:17:35]

G: Quanto tempo mais ou menos a senhora realizaria esse cuidado com a pessoa… que a senhora falou que a pessoa não precisou ir pra Nova Olinda né? Quanto tempo a senhora realizou esse cuidado? Assim… demora pra fazer esse tratamento?

EE4: Vai ter que demorar um pouco né . Que a gente vamos ter que ter um cuidado, de passar um remédio, pra ele não tá andando, que as vezes incha né… vai ter que ter um cuidado ali pra ele não pegar outra infecção lá…

G: E tem algum cuidado específico assim… alguma restrição que a pessoa não possa fazer? Quando ela é mordida, quando a senhora tá tratando? A senhora diz assim oh, não pode fazer isso, não pode fazer aquilo… tem alguma coisa assim?

EE6: Tem!

G: O que?

EE6: Comida remosa…

G: Quais tipos de comida?

EE6: Piranha, a matrinxã, ai.. aquele outro é… essas coisas que faz mal. Queixada também, faz mal. Tem que comer uma comida que não faz mal. No caso… o tucunaré não faz mal, o cará também ele não faz mal, esses tipos de peixe mesmo, bem que não é venenoso.

G: E tem algum cuidado assim… de sair pra algum lugar, o… algum cuidado em relação a mordida onde foi mordido?

EE6: Uhum… vai ter que ter cuidado pra não sentar mosca né, pra não fazer, coisar… tem que passar um remedio que… no caso a copaíba, pra não sentar lá, que não gostam da copaíba.

G: Não gostam?

EE6: Não.

G: A senhora realizaria esse cuidado sozinha ou com alguém?

EE6: Eu poderia convidar alguém pra me ajudar.

G: É?

EE6: Uhum…

[00:19:09]

G: Mas é… auxilia, associa com cuidado médico ou só com seu cuidado?

EE6: Só com meu cuidado.

G: Só com seu cuidado?

EE6: Uhum…

[00:19:19]

G: Dos cuidados que a senhora já fez, a senhora disse que teve uma melhora né?

EE6: É…

[00:19:26]

G: E… Quanto tempo assim, no valor de dias mais ou menos, a senhora usaria esses recursos pra tratar alguém?

EE6: Acho que um mês.

G: Um mês?

EE6: É…

[00:19:38]

G: É… desses recursos que a senhora usou pra cobra né, ou que usaria… a senhora utilizou algo da própria cobra? É… por exemplo, é… a pele ou é… o veneno… ou a banha?

EE6: É… a gente ia passar depôs de coisar tudinho… Eu ia ter que fazer um remédio, pra passar pra não aparecer a marca né… que no caso eu tenho o remédio pra tirar cicatriz, pra não ficar feio, pra limpar… fazer a puxação…

G: Mas seria com a própria parte da cobra?

EE6: Não

G:Seria com o quê?

EE6: Seria com outra parte, do outro remédio já..

G: É? Mas da cobra alguma parte dela, a senhora usaria?

EE6: Não!

G: Pra puxar… ou pra defumar?

EE6: Não. Pra defumar não.

G: Não?

EE6: Não…

[00:20:28]

G: A senhora tem algum é… experiência com cuidado de pessoa mordida de cobra, que a senhora queira relatar?

EE6: Não!

G: Não?

G: Muito obrigada.

**ENTREVISTA 7**

**Caracterização do Entrevistado**

**Idade:** 28 anos

**Especialidade:** Pajé e espírita desde 12 anos de idade.

**Informações Relevantes:**

**a)**  O entrevistado se considera pajé e atua no Centro São Jorge em conjunto com outro pajé, 01 sacaca, 01 banqueiro e 01 ajudante.

**b)** Antes de iniciar a entrevista a sacaca geral e o pajé entrevistado apresentaram a pesquisadora o centro explicando sua funcionalidade, objetivo e registro de quantas pessoas já curaram.

**e)** No decorrer de seus relatos, o entrevistado mencionou ter tido uma experiência espiritual com um guia reconhecido como Honorato (mestre espiritual dos rios) que se transforma em cobra.

**c)** O entrevistado pontuou conhecer os benefícios da banha da jiboia e o uso do feú de paca para mordidas de cobras. E as finalidades da cobra também para feitiço.

**Transcrição da entrevista**

[00:00:01]

G: É… eu queria que o senhor me contasse se existem histórias, lendas, mitos ou contos com cobras em seu povo

EE7: Cobra é? Eu só tenho aquela do Honorato que tava te falando agora há pouco, que a cobra grande né, que a mãe… a mulher se emprenha da cobra, em que veio dois né, acabou que que veio dois que… da história que eu sei né, vem dois… veio o Honorato e aquela Maria Caninana que era a irmã dele né. Aí só que como a irmã dele era mal, ela gostava de alagar as canoas, alagar os barcos ribeirinhos, atacar os ribeirinhos… aí o… tinha o irmão dela, que era esse Honorato, ele era um homem do bem né, que ele não gostava de fazer isso. Inclusive até vinha com sua mãe… o que eu sei da história né… de noite ele virava gente, ele subia em terra, vinha visitar sua mãe, quando… tudo isso tinha que fazer antes de amanhecer né… quando amanhecia o dia, ele tinha que descer na água, que já virava cobra. Aí foi disse que eu sei dessa história, do seu Honorato aí. E surgiu né… da mulher, que estava menstruado, parece que esqueceu, o negócio do… das calcinhas na beira né…aí parece que lá passou um negócio da cobra, do bicho né. Eu sei que emprenhou, veio essas duas cobras, que era seu Honorato e a Maria Caninana que era irmã dele.

G: Então, seu Honorato era um cobra que se virava homem?

EE7: É… ele virava homem… conta a história né… dizia a mãe deles, que falava né, dessa história que eu soube aí, falava que a mãe dele, antes de for… falava pro outro. Foram falando, foram falando, foram falando até chegar no meu ouvido, dessa história agora que eu sei

G: E do nascimento dele então, nasceu ele e uma irmã dele?

EE7: É… é uma irmã dele.

G: Só que a irmã ela era…

EE7: Ela era má. Ela não gostava assim, de ver os ribeirinhos né… como ele… só que ele não né, como ele era gente, Só que só ele se transformava em gente, assim, quando era de noite assim, ele subia numa praia, lá ficava uma capa dele de cobra. Ele ia ver a mãe dele né. E aí, a irmã dele não, a irmã dele já coisou a maldade pra ela… ela alagava canoa, alagava barco… coisava os ribeirinhos aí né… ai como tinha o seu pai também , o pai dela né, uma cobra grande também. Ele era o pai dos dois né, aí ela queria acordar ele né… que as cobras grande que a gente ver hoje em dia né… acredito que essas cobras grandes que a gente ver né, é bem difícil… por aqui já viram umas aí boiadas, né… aqui no rio, cobra grande, boiadas aí… assim era o pai delas, só boiava de vez em quando, aí ela queria se chegar pro lado do pai dela né, pra recordar ele, pra ver se ajuda ela fazer maldade também né. Subir nas cidades aí. Aí foi na hora que o irmão dela também… como ela era do bem né… ele teve coragem, disse que ele não ia deixar ela fazer isso né. Aí eles fizeram um encontro… sei que ele matou essa… a irmã dele, que não era pra fazer maldade. Assim, que falava a história aí… nesse tempo… também eu sei que até virou gente, esse Honorato aí. Que ele ia nos corpo.. Ia de noite nas pessoas né, só que… queria virar gente né, só que pra isso tinha que fazer pra ele um ritual né, um ritual específico pra ele virar gente mesmo, que era pra ele deixar o corpo dele de cobra, que era pra ele ser só gente mesmo.

G: Mas ele é… incorporava em outras pessoas?

EE7: É… não, ele subia em terra, dizendo o… o vovô também até me falava dessa história, que ele subia como gente mesmo. Ia nas festas, subia e, a capa dele de cobra ficava na beira aí, subia pra terra como gente mesmo. Ele não é… não virava, não se transformava em outro não, não vinha no corpo do outro não, era ele … Aí o vovô até conta também que lá na ilha de Marajó, não sei… pra lá que ele falou pra um saldado, que teve coragem… que se o saldado tivesse coragem pra ir até ele, pra fazer esse pequeno ritual aí. Pra ele ficar virado só homem, ela ia dar tudo de riqueza que ele tinha lá no fundo pra ele. Aí o policial foi na ilha lá, assim eu sei dessas histórias… aí lá na ilha lá, ele fez o ritual pra ele né. Aí o vovô até falava que o ritual era pra atirar quando ele viesse boiando, atirar ele com a espingarda, pra acertar na cabeça dele, que era pro sangue escorrer dele… que era pra ele virar homem, virar gente. Foi que o saldado fez… Aí parece que ele ainda veio, de novo pra… aqui nessa terra né, pra… segundo os caras falam, ele ainda apareceu por aqui . Aí passou de novo esse tempos, esses tempos da velhice dele, que ele já estava velho né, aí parece que ele se encetou de novo… foi pra lá… aí teve esse filho de Honoratinho que até lá no Canumã, muita gente já sabe que nós trabalha com espírita, nos conhece ele né, o Honoratinho. Ele é espírita mesmo do fundo, ele.

G: Então, o Honoratinho, ele é filho do seu Honorato?

EE7: É… ele filho do seu Honorato grande mesmo.

G: Ele ainda é vivo?

EE7: É… ainda é vivo, agora o seu Honorato não é mais vivo não.

G: O Honoratinho sim?

EE7: Só esse Honoratinho que já é filho dele

G: E Ele trabalha com a parte espiritual é?

EE7: É… ele é espírita mesmo do fundo.

G: Ele é espírita do fundo?

EE7: Aham, Aham… ele tem o coisa cobra grande mesmo do mar, mas ele é espírito. Aparece quando…

G: Mas no corpo de uma pessoa?

EE7: É… no corpo de uma pessoa!

G: E ele mora aonde?

EE7: Ele mora lá no Canumã lá, no rio do Canumã lá.

G: Uma outra aldeia?

EE7: É… uma outra aldeia, lá no ? Tem um filho… também que trabalha, junto com nós, o Marcelo lá. Que… cuidou de nós aqui, também ele trabalha com esse espírito dessa tal cobra aí.

G: Lá no caso tem o centro espírita?

EE7: É… tem o centro espírita, igual esse daqui, ele baixa lá, ele que é chefe também lá…

G: Aqui nunca baixou?

EE7: Aqui não… ele já veio aqui já… baixou aqui também…

G: O Honorato?

EE7: É… o Honorato, quando ele veio sentar um trabalho pra nós… ele veio administrar… ele veio aqui…

G: Mas ele baixou como cobra?

EE7: Ele baixou como cobra!

G: Ele veio com trabalho específico?

EE7: Ele veio com trabalho específico. Que era ajeitar, só que ele vem em espírito em forma de homem mesmo.

G: Ele que batizou vocês?

EE7: É… ele que batizou nós. Ele veio em forma de homem, na parede dele

G: Então, quando aconteceu aqui o Batismo, do centro espírita, foi o filho dele que veio, e… incorporou o pai?

EE7: É!

G: Que é seu Honorato?

EE7: É…

G: Ele batizou vocês?

EE7: É… ele batizou

G: Isso faz quanto tempo?

EE7: Ta fazendo uns dez anos atrás…

G: Dez anos atrás?

EE7: É

[00:07:076]

G: É… O senhor já teve alguma experiência com cobras aqui na aldeia?

E: Aqui… não. Com cobra nunca teve experiência.

G: Assim… Ter encontrado, de alguém que ter sido mordido

EE7: É… Nunca cuidei… Eu nunca cuidei de pessoas assim, que tenha mordido de cobra. Nunca cuidei não.

[00:07:24]

G: As cobras tem algum significado pro senhor?

EE7: Pra mim as cobras, pra mim o significado, que eu coiso das cobras pra mim né, são coisas, da parte do inimigo né. Que… é o que pra mim significa né. Porque elas que coisa… basta… nós tá tá pecando. Dois em dois, por causa dela né ???

G: Então, ela tem o significado negativo?

EE7: É… negativo pra mim.

G: Ela tem o significado, é… de maldade por e exemplo?

EE7: É, de maldade.

[00:08:05]

G: É… As cobras elas possuem alguma utilidade no seu dia-a-dia, elas servem pra algo?

EE7: Pra.. pra… até a das banhas né, da cobra né. A banha serve pra remédio que eu uso né, por conta.. Assim da inflamação, contra até esse negócio de judiaria mesmo, eu só né, a banha dela…

G: O senhor já tratou alguém com a banha da cobra?

EE7: Já, já tratei pessoas com banha de cobra. Quando pega um baque, quando tá inchado assim, inflamação assim, quando tem no inchaço, é muito bom também banha de cobra. Depende da cobra né. Eu tô falando… Tem da sicuriju, tem da surucucu também, da jiboia também. Que muita gente usa a banha pra.. Eu vi... diz a mulher, eu… vi um velhinho lá no laranjal, onde a gente tava, que usou a banha da jiboia pra, pra vista né, como colírio, ele usou a banha dela.

G: E aqui o senhor, já tratou alguém com algo da cobra?

EE7: Já tratei pessoas aqui… quando aparece com pé inchado… como a banha da sucuri né, do sucuriju que eu falei já, pra passar em rasgadura, pra passar assim… em baqui né, quando a gente pega um baqui, pra passar em cima, pra desinflamar, desinchar… isso daí que eu já trabalhei.

G: O senhor já defumou alguém com cobra?

EE7: Não. Pra defumação, nunca usei cobra não.

[00:09:37]

G: É… Quais tipos de cobras o senhor conhece?

EE7: Eu conheço a sucuriju, eu conheço a jiboia, eu conheço a surucucu, eu conheço aquela tal de acoral, eu conheço aquela cobra papagaio, eu conheço umas cobras roxas também, que eu não sei como que é o nome delas direito, umas verdes também que tem, só que eu não sei o nome delas, mas essas daí que eu conheço.

[00:10:02]

G: Qual delas o senhor considera a mais perigosa?

EE7: É, a surucucu pico de jaca.

G: O senhor já viu ela?

EE7: Já!

G: Já? Já teve experiência de encontrar?

EE7: Já, já tive experiência de encontrar sim, mas só lá no mato né, que eu encontrei elas. Inclusive eu já até matei.

G: Já matou?

EE7: Já matei

G: Como foi? Quando foi também?

EE7: Foi lá quando tava tirando copaíba com papai, lá na mata, ali dentro. Ele ia na frente, ia roçando, ainda bem que era limpo lá assim… ia roçando na frente, quando vi… nós ia espiando pra cima, ia espiando pra cima, pra guia das árvores, ver se a gente enxergava copaíba. Aí foi na hora que ele falou… para aí, que tem uma surucucu. Eu falei cadê, tá ali, ela tava embolada lá né. Aí também ele não gosta de surucucu, que q gente muita gente que cobra também já matou né, já aleijou né. Aí ele foi lá… bora matar ela, nos matemos ela, matemos ela… deixamos ela lá né, não tiramos nada não, ficou lá mesmo.

[00:11:00]

G: E… o senhor sabe é, se as cobras possuem nome na linguagem Munduruku?

EE7: Possui!

G: Sabe chamar cobra em Munduruku?

EE7: Cobra eu não sei não. Não sei falar o nome não, mas tinha uma tia ali do outro lado, que era nossa tia, que ela falava todinho os nomes da cobra na linguagem né. Não gravei nenhum na minha cabeça, mas tem.

G: É? E na língua diária como o senhor chama elas?

EE7: Como assim na língua diária?

G: Tem um nome específico?

EE7: Não, acho que não.

G: Só os nomes que o senhor já me falou?

EE7: É. Só os nomes que eu já falei.

[00:11:40]

G: O senhor é, realiza algum cuidado em pessoas envenenadas por serpentes?

EE7: É… muitas… sabe assim de ver, muitas vezes já coisei, porque muitas vezes tem essas cobras surucucu, que eu tô falando… A… porque tem pessoas né, que quando está mordidas com cobras, tem certas pessoas que não pode olhar o ferimento da pessoa né, porque dói, tem não sei o que… dá um agitamento na pessoa lá né. Então… mas isso daí.. O Raimundinho pegou uma mordida né, pra melhorar, não deixaram nós ver né, devido esse negócio aí, isso que…

G: Uhum… Tem um cuidado específico que a pessoa mordida por cobra, tem que ter?

EE7: É , um cuidado específico. Porque não é todas pessoas que pode ver. A grávida não pode ver a pessoa mordida de cobra. Tem certas pessoas também não pode ver disque, o veneno coisa na pessoa, dói, uma coisa assim, grita, fica logo… e algumas pessoa pode ver assim…

G: Tem um tempo assim, pras pessoas não visitarem?

EE7: Aham! É Tem um tempo…

G: O senhor sabe quanto tempo?

EE7: Quase… praticamente um mês assim…. A pessoa não pode tá vendo.

[00:12:57]

G: E o senhor pode me relatar quais cuidados o senhor realiza, como o senhor realiza, esses cuidados? O senhor me disse que trabalha com cura né?

EE7: É!

GG7: Como é esse cuidado que o senhor realiza?

EE7: Que cuidado que eu realizo?

G: É… o senhor pode falar dessa cuidado aqui. Como é que o senhor realiza?

EE7: Hãn… daqui do centro? Ah sim. É como eu tô dizendo, de mordido de cobra, posso fala assim? Se chegar alguém por mordido de cobra?

G: Pode!

EE7: Se chegar alguém por mordido de cobra né, eu já sei, que eu não posso… vai ser só eu com o pessoal da saúde lá, e eu que vou fazer meu trabalho aqui né, não vou querer pessoa né. E também, eu também, como tá rolando agora na minha coisa, que tá clareado né, em caso de emergência né, a gente tem que pensar e analisar né… Por exemplo, tem a folha a folha do ananás. A folha do ananás também é muito bom pra mordida de cobra, que o moço lá, também falou pra mim.. Que é um velho lá né, que falou pra mim, que é bom também né. A folha do ananás, machuca bem… pa-pa.. misgalha bem misgalhadinho, tira o sumo dele né, e pode passar em cima de onde a cobra mordeu né. Passar, puxar, pra não inchar. Passar , passar e puxar. E Ferver também pra tomar. Aqui também tem uma folha de capeba, que ele falou também que é bom pra inchaço né… Pra mordida de cobra, pode amornar um bucadinho, e pode passar só o sumo, em cima da pele, disque a bateria também não deixa inchar..

G: Amorna, coloca em cima, e fica quanto tempo?

EE7: É… Fica lá assim… durante umas meia hora assim. Aí né, pega outra de novo, quando aquela lá tiver muxa, pega outra de novo, assim… muxa de novo pra…

G: As pessoas que são mordidas assim, em algum momento, elas procuram é… o atendimento aqui do centro?

EE7: Aqui nunca teve esse atendimento…Como eu tava dizendo… Nunca teve esse atendimento assim de pessoas né, de mordidas de cobra, aqui não. Porque muitas vezes, a pessoa quando pega mordida de cobra né, já vão direto lá pro polo, não passa por aqui na nossa ceara.

G: Mas aqui o senhor já defumou alguém com couro de Cobra?

EE7: Aqui no centro da não, ainda não defumei ninguém com…

G: Já tratou alguém assim, é… judiado por exemplo?

EE7: Já… judiado já.

G: Mas alguém judou com algum algo da cobra?

EE7: É… com algo da cobra. Tem uns que judiam com o dentre da cobra né.

G: Como faz?

EE7: Rapaz, esse daí não posso te explicar direito né, como que o parceiro faz né, pra mandar aquele… com o dente da cobra. Tem uns que enfeitiça com dente, tem uns que enfeitiçam com o veneno da cobra, que dar umas certas feridas né, no couro da pessoa né, outos feitiços também, aí dá uma dor de estômago, dá um certo… coisa no parceiro né, de doença assim. O parceiro fica triste, fica com febre, mas não sei como que faz né, pra…

G: E como vocês aqui no centro espírita sabem é.. que era algo da cobra assim, que a pessoa usou?

EE7: É que nós temos aqui, nossa copia de evidência, e aqui também, como temos os espíritos, eles indicam todinho porque já, eles… tem muitos que aqui, caboco, que não trabalham só aqui, como na cidade grande né. Tem um conhecimento, aí eles indicam pra gente, o material

G: Entendi… quando alguém chega aqui, vocês fazem é… a vidência?

EE7: A vidência né…

G: Na vidência, o guia de vocês ele…

EE7: Eles falam… o moço tá enfeitiçado com isso, isso. Ensinam ao mesmo tempo o remédio, pra passar pra…

G: Mas nesse caso aqui, já chegou assim, com o senhor, alguém…

EE7: E já…

G: Foi é… enfeitiçado com algo da cobra

EE7: E já!

G: É? E conseguiu é tratar?

EE7: Conseguimos tratar, conseguimos tratar com os remédios medicinais mesmo daqui, da região. Até mesmo a banha a da jiboia, que eu usei né, a banha da jiboia. Esse negócio… palha de ananás, com esse capeba, isso tudinho já fiz aqui,

[00:17:01]

G: E quando o senhor faz esse cuidado assim, pra alguém que foi enfeitiçado por exemplo. Usa vela, algo no ritual?

EE7: Usa, usa normalmente a vela, aqui na nossa ceara precisa disso daí.

G: Ai a ceara no caso tem uma cor específica pra essa vela?

EE7: Tem. Tem que ser vela branca.

G: Só vela branca?

EE7: Só vela branca. Vela da luz, que tem a caixa bem…

G: E além da vela branca, vocês também registram esses atendimentos?

EE7: É… registra o atendimento aí.

G: Toda pessoa que passa aqui é registrada?

ES7: É… toda pessoa que passa aqui, é registrada. Só como eu tava falando pra senhora, naquela hora que eu tava lhe informando. Muita vezes a gente tá aqui né… Vez chega uma vizinha aí que tá com quebranto, que tá com alguma desmentidura na costa, tem gente… Quando é trabalho mesmo…

G: E quando é trabalho, é… chamam os outros é… curadores?

EE7: Chamam…

G: Ou geralmente chamam só um?

EE7: Não. Chamam é… aqui nos faz trabalho nós três. Por exemplo, aqui nos chega uma pessoa que tá com espírito mal, chega aí pulando e bei-bei… aí aquele lá que eu tava lhe falando, meu pai né, é ele que fica encarregado. Por exemplo não to aqui, tô pra li, a titia tá pra li, aí chega, rapaz vou lá com os cara. Ai vai lá pra minha roça me chama, ou vai na tia Luziete ou então chama nós três. Ai chega aqui né. Nós aqui né, nós entra aqui dentro, acende nossas velas aí né, tem nossas espadas, santos aqui são todos batizados.

G: Aqui são as espadas?

EE7: É, nossas espadas. Nós só tem três. Assim quando eu sai daqui, todo tinha seus filhos aqui, tudo era santo, cada espada dessa era de uma gente.

G: Ah… cada espada tinha um dono?

EE7: É, cada espada tinha um dono. Ai como nós fizemos trabalho, era tudo jovem né, no começo, parece que eles tavam todo animado, depois pro meio do fim, deixaram tudo pra nós três, nós era dezessete.

G: Ahh… era dezessete pessoas que trabalhavam aqui, e só ficaram três?

EE7: É, só ficamos nós três. Eu, ela e o filho dela.

G: E as espadas que tem outras cores?

EE7: Essas daqui?

G: É…

EE7: Essas daqui?

G: É!

EE7: Essas daqui é pra espírito… assim, espírito mal né, que a gente tira com espada vermelha. Aprende com ele, na espada vermelha. Essas aqui azul, essa verdes, era dos guia mesmo, que eles vinham aqui e pediam, que pediam a cor da espada deles, que queriam trabalhar né. Essa daqui…

G: Então, toda vez que vocês atendem alguém, vocês estão com a espada?

EE7: É… com nossas espadas branca. Esses daqui é um material muito preciso né, pra amarrar espírito, pra expulsar espírito, pra judiaria né, é esses mateias que nós usa, espada da branca.

G: Todos três então, sempre estão…

EE7: É… todos os três tamo…

G: E o banqueiro também ele usa alguma espada ou não?

EE7: Não, o banqueiro não usa. Inclusive né, nós temos nosso colar também né, pro nosso trabalho, que eles precisam muito pra defesa, pra quando eles fazem trabalho, nada de mal aconteça com eles. Eles mesmo preparam né, com a benzição deles, com a defumação deles, com a reza dele. Inclusive o banqueiro tem um. Chega aqui, ele fica aqui no meio d gente, só que como os guias entra aqui, eles ficam tudo acompanhando por aqui que nada de ruim venha… se entrar em espírito mal aqui daqui eles já vão tirando, vão colocando pra fora. E o nosso fica aqui. Aqui só tem de ficar o espírito do bem mesmo.

[00:20:18]

G: Entendi… É esse cuidado né, que o senhor desenvolve essa habilidade, o senhor herdou de algum familiar ou de alguém que era especialista daqui da sua etnia?

EE7: Eu acredito que sim. Porque aqui na nossa, no nosso rio aqui, tinha muito. Tinha ali no… que era também nosso parente, um tal de finado, que ele era pajé mesmo, podia cuidar também né de espírito, afastar espírito, costurar rasgadura, pegar dismentidura, tirar judiaria. Ele trabalha… que era nosso parente… acredito que alguns deles, a gente herdou esse dom…

G: Mas nunca ninguém chegou com o senhor pra lhe ensinar?

EE7: Não, nunca ninguém chegou. Ninguém me ensinou não, eu mesmo aprendi.

G: Mas vocês já se prepararam pra…

EE7: Preparemo…

G: Fazer o centro, alguém veio aqui…?

EE7: Aham, veio. O pessoal lá do centro São Jorge, quem tem lá no Caioe, centro Caioe, que tem esse centro São Jorge, que também é membro de lá né. Fomos lá com eles lá… Lá eles preparam… Lá que eles descobriram que a gente podia né… trabalhar sobre, assim… no espiritismo. Aí eles vieram lá, passaram uns negócios de… até uns banhos, remédios pra nós né, noa fizemos. Aí quando eles vieram aqui, eles batizaram nós aqui.

G: Então, aqui faz parte de lá?

EE7: É, faz parte de lá! Só um membro. E botaram esse daqui , Centro São Jorge do Vila Nova, e ficou o São Jorge lá do Kanumã. Porque era muito longe né, pra gente trabalhar aqui. Não tinha nenhum local pra nós. Porque ele lá fizeram um local pra eles. Aí vieram aqui. Abriram esse espaço, batizaram esse local aqui todinho, em espírito né. Esse Honoratinho que eu tô falando pra senhora. Ele que batizou isso daqui todinho, com o sacaquinha que esse lá também, que batizaram tudinho… esse local todinho, pra gente trabalhar. Então, eles fecharam tudo aqui, deixaram os caboco, os guias aí, e deixaram nós fazendo nossa parte, é por isso que nós temos esse pedaço de terra.

[00:22:11]

G: Esse cuidado, o senhor, se o tivesse que realizar, em que momento que o senhor ia realizar, pensando em alguém que foi mordido por cobra?

EE7: Por exemplo se chegasse aqui comigo?

G: Uhum…

EE7: Eu… Como que eu ia realizar, assim cuidar dele?

G: Sim. Se era logo após a mordia ou se ia ser depois… que ele voltasse de Nova Olinda?

EE7: Eu acredito que… se viesse me… um parente meu daqui né, que somos todos parentes… viesse me procurar, assim, pra… pra mim cuidar dele né, por mordida de cobra…. É como eu tô falando que eu não sei né, essa informação de cobra, eu ia indicar que fosse primeiro lá na cidade, com os médicos, com os doutor lá, pra fazerem o trabalho deles. E como lá, eles já tem também a injeção né, que é contra o veneno da cobra. Ai se viesse… Quando ele viesse de lá, já tivesse tudo normal que eu ia já cuidar, por exemplo, pra não inchar né, no inchaço passar um remédio pra ele não ficar manquejando… Isso eu já ia fazer depois que ele viesse de lá.

[00:23:15]

G: O senhor ia associar né. Um cuidado seu com o médico né?

EE7: É!

[00:23:20]

G: É… dos cuidados que senhor ainda não realizou, mas do que o senhor contou né… o senhor falou que teve um, que foi um cuidado que o seu avô fez?

EE7: Foi!

G: Como foi?

EE7: Essa daí foi do parceiro né… daquele tempo eles trabalham né, só aí, nos centro. Nem tinha assim, esse negócios de motor, era no remo… Aí foi a cobra surucucu né, que me mordeu o parceiro dele lá. Estavam lá no centro, aí a cobra mordeu o parceiro dele lá, aí o parceiro dele né… ela carregaram pra sair da estrada lá pro tapiri, que eles tavam no garapé, botaram ele no tapiri. Aí tinha esse outro parceiro dele, que era pajé também né… Aí ele falou, eu vou já com o compadre… era compadre dele lá. Aí ele foi até esse finado Morera, que eu tava falando. Ele que era o sacaca aqui na região mais respeitado né. Todo mundo acreditava e confiava nele. E foi lá que ele ensinou pra ele, a cadê aquela?… inclusive né, tinha até matado uma paca né, naquela bendita noite. Eles mataram a paca a noite. No outro dia, de manhã, já ficou sabendo que a conta mordeu ele. Foi judiaria… foram pra lá né, aí o moço pegou o féu da paca, dele pra ele, e o parceiro dele lá caído, gritando com dor, mordido da cobra. Ele pegou o féu da paca né, rasgou aquele coisa da paca né, botou lá em cima da mordida cobra né. Tira esse pedaço de banha aí, pra tirar, pra dar pro homem… ai disque foram lá na paca, ainda bem que ela tava um pouco gorda né. Tiraram um pouco de banha lá, botaram numa panela lá, sei que tiraram o negócio da banha da paca, botaram pra ele no café, pra ele tomar. Aí ele tomou. O moço ia lá com o féu da paca, aí passava também essa folha do ananás, com essa folha capeba. Só que a dele lá, foi a folha desse ananarana, desse beirada mesmo, que sempre dar. Sem ser esse ananás que dá muito. Aí o homem fez o remédio pra ele. Ele viu lá… batiam lá daquele ananarana, colocava em cima da cobra né… Foi, Foi… aí o homem ficava lá com ele né. Foi esse remédio que ele usou pra dois, três dias… e melhorou.

[00:25:37]

G: É… o senhor é, usa algum… esses recursos que o senhor usa, eles são fácies de ser encontrados aqui?

EE7: São!

G: São?

EE7: São!

[00:25:48]

G: Quanto tem mais ou menos leva pra encontrar esses recurso?

EE7: Esses recursos aí… que inclusive aqui na nossa, na nossa… uns cinco minutos, eu acho que a gente já tem. Porque aqui nessa região, que tem né, esse remédio aí.

G: A folha de ananás, por exemplo.

EE7: A folha do ananás tem aí.

G: O féu da paca…

EE7: A folha da capeba… agora a o féu da paca a gente não tem, não matemos mais paca esses dias. Porque ela também, tem tipo um líquido né, se agente tiver paca, pode guardar. Mas a gente não matemos mais paca esses tempos.

[00:26:22]

G: E se o senhor tivesse que usar algo da própria cobra… o senhor já precisou usaram algo dela numa mordida de cobra? Vou dar um exemplo pro senhor… tipo é… o couro, é a carne da cobra, o… a banha? Usaria alguma coisa?

EE7: Usaria… a banha né. A banha também… teve um rapaz que já foi mordido de cobra, nós tava conversando com ele… ah rapaz, quando eu peguei mordida de cobra, rum… foi pro hospital, lá eles me aplicaram uma injeção, aí nada… quando ele tava inchando, sentia o corpo dele inchando assim… sentia que o fôlego dele tava fechando, aí esse tal de (...) que eu pra ti, que ele também é pajé daí de cima. Ah tu tem que comprar duas latinhas daquele leite moça que é pra ti tomar. Aí ele comprou, duas latas daquele lá né. O pai dele mais que depressa agoniado, comprou duas latas de leite moça pra ele tomar né. Ele falou que ele tomou né, tomou aquele leite moça. Ele se sentiu né, Amodo que foi amodo desinchando. Ele tomou de novo mais outras… sei que ele falou que aquele que vinha fechando ele, acalmou né. Ele sentiu que não tava mais inchando. Aí foi acalmando. O moço que falou pra ele, ah rapaz, pra não ficar aleijado assim, tu pega a banha da jiboia, tu tira um pouco da baga da jiboia pra passar em cima, toma pra ti não… pro veneno da cobra não entrar mesmo no seu sangue. Aí ele falou que quando ele voltou lá pra casa dele, aí o pai dele andou né, atrás da banha da jiboia, da cobra, da banha da jiboia. Ele passava em cima né, e tomava e graças a Deus diz ele, não andou com a perna dele mancando não, ele ficou normal.

G: Mas ele também foi pra atendimento médico

EE7: Ele foi…

G: Ele foi né

EE7: Ele tomou injeção. Só que ele falou que não tava dando jeito não. O que deu jeito mesmo nele foi o leite moça, que ele falou que comprou, duas latas pra ele tomar. Combater o inchaço dele e a banha da jiboia que ele tomou.

G: D própria cobra, mas de uma jiboia?

EE7: É, da própria cobra, mas da jiboia.

[00:28:40]

G: O senhor tem é… alguma coisa mais que o senhor queira acrescentar, relacionado a cobra? Assim pra etnia Munduruku a cobra ela tem uma representatividade?

EE7: Até nesse ponto, não sei se ela tem representatividade aqui na comunidade né. Assim, pro povo Munduruku né. Eu também ainda sou jovem. Não sei se as pessoas mais idosas né, tenham esse conhecimento, esse nome específico da cobra aqui no rio né, Munduruku. Eu não tenho, dizer assim, um nome específico que a cobra significa. Entendeu? Eu não tenho esse conhecimento não. Talvez uma pessoa mais idosa tenha…

G: Entendi! Então, muito obrigada tá (...).

EE7: Tá…

G: Obrigada pela sua participação.

**ENTREVISTA 8**

**Caracterização do Entrevistado**

**Idade:** 46 anos

**Especialidade:** Sacaca/ Pajé (Há mais ou menos 21 anos)

**Informações Relevantes:**

**a)** A entrevistada informou ser sacaca geral por batismo e coroação nas águas.

**b)** A entrevista foi realizada em um local que recebe várias pessoas de Nova Olinda e Borba para tratamentos e curas espirituais e de pajelança.

**c)**  Embora a entrevistada não tenha vivenciado nenhuma experiência com mordida de cobras, ela mencionou conhecer remédios para este caso como o suco do limão, o sumo da capeba, o sumo do cupuaçu e o uso do ananã do mato.

**d)** Em seus relatos a Sacaca mencionou restrições e medidas de resguardo em acidentes ofídicos como o contato com olhos venenos de mulheres gravida, bem como de mulheres menstruadas durante o processo de tratamento e cura.

**e)** Na entrevista também foram citadas restrições alimentares reconhecidas como “comidas remosas” como: Matrinchã, piranha, porco, queixada, anta, macaco prego.

**f)** Em relação aos cuidados com recursos da própria serpente, a Sacaca, mencionou o uso do couro para defumação e os rituais de canto.

**Transcrição da entrevista**

[00:00:03]

G: Me conte se existe histórias, lendas, mitos, contos com cobras em seu povo?

EE8: Sim, na minha família acho que tem uma, uma prima minha que ela foi encantada, hoje ela é uma encantada, que se chama cobra, que se chama branca. Então, a branca mora la no rio, no rio Urariá, ali na boca do bem assim ne, então ela foi um parente que a gente perdamos por causa que ela era sacaca e nenhuma família, é considerava que ela fosse uma sacaca também ne, então ela se tornou cobra por causa né. Então a gente, nos quando é sacaca de nascença, nós desde quando a gente se, de 08 anos pra lá a gente já sabe e conhece, as vezes pelo olhar das pessoas, qual e as pessoas que vai fazer o bem e qual vai fazer o mal pra nós. Então, eu tenho esse dom, que eu tenho revelação, quando eu olho pra uma pessoa, com qual pessoa que vem pra fazer uma pergunta de bem e qual pessoa que vem me perguntar mal ne, então eu tenho uma resposta sempre pra eu tá explicando pra essa pessoa. Então, esse meu dom que eu tenho é, eu acho que pra mim é muito bom, eu já vejo, conheço o pensamento da pessoa, eu olho pra pessoa e já vejo o pensamento dela, então eu tenho uma revelação comigo, eu acho que foi Deus mesmo que me deu isso, por que tem pessoas que fala que nós semos assim, pajé, sacaca, mas tem pessoas que não acredita em nós e diz que nós semo, que é o inimigo, mas eu acho que não é não, por que semos de Deus, porque se Deus deu uma revelação pra mim vê o pensamento dessas pessoa é porque ele quer que eu cure aquela pessoa, que que eu cuide dessa pessoa ali para que ela se sente bem. Então é isso que eu sou, sou sacaca, eu trabalho aqui, nesse Centro São Jorge há 21 anos já né, e eu tenho muitas experiencia de remédios caseiros, pra todo tipo de doença que for preciso eu sei, eu tenho minha receita feita nas atas, feita lá em casa, que eu tenho minha ata só de receita também, eu tenho pra todo tipo de doença como, diabete, como né, esse colesterol alto, infecção urinária, pá fígado, inflamação de fígado, pá inflamação de útero, tudo eu tenho o meu remédio que meus guia me ensina, eu vo anotando pra que, quando eu preciso desse remédio eu já tenho notado ali, e tá todo anotadinho no meu caderno, eu tenho um pouco de dificuldade de escrever, mas de lê quase não tenho, mas o meu marido sempre escreve mais bem do que eu, ele tá fazendo todinho pra mim, mas ai quando tá escrevido, eu vo lá lendo, já vo lá pegando os meus remédios que precisa.

G: Então, quando a senhora recebe uma informação do seu guia é seu esposo que escreve?

EE8: É meu esposo que escreve...

G: A senhora fala e ele escreve?

EE8: Eu falo e ele escreve

G: E ele é seu banqueiro a todos esses anos de atuação?

EE8: É, 21 anos ele é o meu banqueiro, todos esses anos.

G: E essa história que a senhora me contou da branca do Urariá, a senhora lembra a idade dela e como foi que aconteceu, como lhe contavam?

EE8: A minha prima ela tinha tree, ela tinha 15 anos né, então ela trouxe um dom pra curar, mas só que ninguém, a mãe e o pai num queriam que ela seguisse essa vida de pajé né, porque que o pai dela e a mae não queria que ela seguisse a vida de pajé, por case que, tem uns que acreditsm e tem uns que não acreditam, ai as pessoas zombam da cara da gente, a fulano não sabe de nada, a esse fulano não sabe nada, por isso né, que meu tio e minha tia não queriam que minha prima fosse pajé. E segundo, num dia de sexta feira ela ficou muito agoniada, ela disse que ia dormir e não sabia se ia acordar. Aí a mãe dela saiu pra ir pra roça, quando ela saiu pra ir pra roça, ai ela disse que ia tomar banho. Então nessa hora, meio dia, ela foi tomar banho, quando ela viu uma onda bem grande, ela desceu pra tomar banho, foi na hora que ela se encantou e virou a cobra maior que tem ai no rio Urariá é a branca. Ai, até hoje ela tá lá, sempre ela vem aqui, baixa aqui com a gente aqui tumbém, a gente conversa com ela e ela disse que tem muita vontade de sair de novo de lá, mas ela não sabe como. A gente também, não tentemo tirar ela por que já é muitos anos que ela tá ali, acho que, tem pra mais de 26 anos, 27 anos.

[00:05:02]

G: A senhora já teve alguma experiencia com cobra aqui na sua aldeia?

EE8: Ah sim de mordida de cobra, ai, a gente dá não teve experiencia com mordida de cobra aqui na nossa aldeia, por que é muito raro ter uma pessoa que seja mordida de cobra né, mas a gente conhece remédios caseiros, que possa também..., fazer, mas a gente ainda não pratiquemo em cima da cisura da mordida de cobra né, mas a gente tem remédio que possa, que faz bem também pro nosso parente aqui dentro do rio, da nossa aldeia. Principalmente, como o suco do limão, como, a, a, o sumo da capeba, o sumo do cupuaçu, da casca dele, a gente raspa o cupuaçuzeiro, da ávore, tira aquele sumo pra fazer, pra da pro nosso parente beber, aquele sumo da casca do cupuaçu, pra que o veneno da cobra não se espalhe pelo corpo dele, pra que ele fique num, num, fique tratado da mordida da cobra. Nós temo também do ananã do mato, que a gente chama de ananã do mato, é, é, a gente tira a palha dele e bota pra muchá no fogo, ai tira aquele caldo de dentro da palha dele, pra fazer também, dá pro nosso parente tomar pra mordida da cobra, também pra ele não ficar inchado,quando a cobra morde as pessoa incha a perna, incha lá onde ta mordido, isso tudo serve pra mordida da cobra....

[00:06:35]

G: É, é, a senhora, é, as cobras , elas possuem algum significado particular?

EE8: Particular, como assim, deee?

G: As cobras tem assim, alguma importância, alguma serventia, algum valor pra senhora?

EE8: ..., Sim, a cobra, a jiboia tem um valor pra nós né, se nós tem, nós pega uma jiboia, a rente tira a cabeça dela, a gente bota dentro do álcool, pra que a gente possa bota dentro da nossa casa, pra que não falte o dinheiro pra nós, não falte outras, nada de rancho, essas coisas dentro da nossa casa. Então, a jiboia é uma cobra que ela é muito trai, traiçoeira, traia, trai as pessoas né, então pra trazer os benefícios pra nós. Então a cobra ela tem essa serventia pra nós né,

G: E as demais cobras?

EE8: A surucucu é, é uma cobra que ela é um pouco atraiçoeira, mas é uma cobra que ela também é importante na nossa aldeia, como nós indígena, por cause que ela, tanto, a, a, a, surucucu por cause que ela é uma cobra que, a gente, tem serventia pra nós, porque a pele da cobra e a banha dela serve para, pra fazer negocio, quando tem aqueles, que falam de fitiço né. Então, a gente tira a banha da surucucu pra gente passar em cima, quando a gente tá enfeitiçado, a pele dela a gente faz defumação em pessoas que tão com derrame, então essas pesso..., a pele da surucucu serve pra isso. Então, ela tem serventia pra nós, a cobra assim é importante pra nós, por que nós semo indígena né, a gente indígena, antigamente, no tempo do meus avô, da minha avó, a gente num ia pro hospital por que a minha avô cuidava dos meu avõ, meus tio,só com banha de cobra, de cobra né, como a banha da jiboia. A banha da jiboia serve pra inchaço, a banha da jiboia serve pra gente botar no, como já foi falado. Mas ai, tudo isso serve, a banha da surucucu serve também pra passar em reumatismo, que tem reumatismo tem, pode passar a banha do surucucu, serve pra reumatismo também a banha do surucucu, não é so pra quem ta enfeitiçado não. E também serve, a pele do surucucu pra defumar a sua casa pra que nada do mal possa tá olhando ali dentro da sua casa, por que ar vezes a gente não sabe quem vai lá com o coração bom e não sabe quem vai lá com coração ruim, porque a gente conhece a cara e não conheci o coração das pessoas. Então a pele do surucucu é pra fazer defumação em casa, pra não deixar que nada do mal possa acontecer dentro da sua casa, pra tirar essemal, olho gordo, inveja que as pessoas tem as vezes da gente, que a vez a gente não sabe, mas. Principalmente pessoas que não conhece, que não tem um dom ansim né, ai se a banha, a pele da surucucu, serve pra isso.

[00:09:44]

G: Quais são os tipos de cobra que a senhora conhece?

EE8: Eu conheço a jiboia, a surucucu, a surucucurana, a surucucu pico de jaca, cobra coral, aquela surucu que, aquela cobra verde que fala, que meu pai falava cobra cipó né, que a gente conheci também, que dá na água, é, é a sucuriju, conhecida também, esses tipos de cobra a gente conhece também?

[00:10:24]

G: Qual delas a senhora acha mais perigosa?

EE8: A surucucu. A surucucu pico de jaca.

[00:10:32]

G: E as cobras, elas possuem é, na linguagem Munduruku algum nome especifico?

EE8: Possui sim.

G: Como é que vocês chamam?

EE8: Payba oie ,

G: Poibã?

EE8: Payba oie.

G: Oie?

G: Poibu oie.

EE8: É, é a cobra mais perigosa que tem.

G: Hurum..., que é a surucucu no caso?

EE8: Que é a surucucu pico de jaca.

G: Pico de jaca, ela que é poibu oie?

EE8: Payba oie.

G: Oie.

EE8: Oie (risos).

[00:11:05]

G: E as, as cobras no geral tem um nome?

EE8: Tem, Payba sú.

G: Poibã?

EE8: Payba sú...(riso), meu marido diz, eu não sei escrever Munduruku não.

G: A senhora sabe escrever Munduruku?

EE8: Não, a minha irmã escreve.

G: Ela escreve né?

EE8: Escreve.

[00: 11:27]

G: A senhora realiza algum cuidado com pessoas envenenadas por serpentes?

EE8: ...., que seja....

G: Se já realizou cuidado com alguém que já foi mordido?

EE8: Não, não.

G: Mas realizaria?

EE8: Sim.

[00:11:50]

G: Se a senhora fosse realizar, quais cuidados a senhora faria?

EE8: É..., ia ter o cuidado com ele, principalmente não tá no meio de muitas pessoas, que as veze tem pessoas que tem o olho venenoso que fala né, e também, principalmente, mulheres gravida né, e também mulher menstruadas que não poderia olhar nosso, nosso paciente mordido de cobra. Então esses cuidados eu ia ter também, pra que nenhuma dessas pessoa poderia tá olhando pra ele, pra que ele não pudesse passar mal.

[00: 12:21]

G: E o que a senhora faria pra cuidar dele?

EE8: Pra mim cuidar dele né, tinha que ter um ambiente bem estruturado pra mim tá coidando dele. Principalmente com remédio, nosso remédio caseiro né, uma comida, por que também não pode comer comida remosa, como matrinchã, piranha, é porco, queixada, anta, macaco prego, esses aí não poderia comer. Tatu, esses são as comidas remosa que ele não poderia ta comendo quando tivesse mordido de cobra.

[00:12:54]

G: A senhora faria algum benzimento, reza?

EE8: Sim, benzia, se tivesse com muita dor, a gente ta ali pra benzer pra calmar aquela dor. A gente benzia ele pra calmar a dor.

G: Algum ritual, alguma defumação?

EE8: Ritual, defumação também. Pêlo de macaco prego, pelo de paca, todo isso serve pra tirar a dor que ele tá sentindo, então é esse ai é nossos cuidado pro nosso paciente que, no dia que, ainda não tenha cuidade, mas se aparecer um dia a gente tem que fazer esse ritual ai. Cantar o ritual da mordida da cobra também.

G: Como é ritual da mordida da cobra?

EE8: Dançar e cantar, e ele fica no meio da roda.

G: Ele ficaria deitado?

EE8: Ele ficaria deitado ai no meio da roda enquanto nós ia cântaro ritual da mordida da cobra e dançar.

G: Mas a senhora sabe cantar esse ritual?

EE8: (Risos)...

EE8: Num sei, só se for um dacinho, pro causo que num peguei bem pra mim aprender, que nem..

G: Mas só um pedacinho que a senhora sabe...

EE8: Cantei o ritual lá, lá da mata donde a cobra moraaa, mermão não choraaa, meu irmão não choraaa. E essa cobra venenosa, vamo espera que ela morre, num chora, meu irmão não chore.... ( Cantando)

EE8: Dos outro lá,(risos..), sei bem não...

G: Enquanto ele tivesse deitado no cuidado, vocês estariam...?

EE8: Estaria fazendo isso.

G: E usaria mais alguma coisa, assim vela?

EE8: Vela, arco, que usaria ritual de tudo quanto é coisa... Pessoas que tão cum...

G: Teria algum guia específico?

EE8: Tem, tem...

G: Que guia por exemplo se aparecesse alguém mordido de cobra?

EE8: ..,é que são daqui, são os índios né, tanto eles moram na mata mesmo, e eles são guias que vem da mata e falam todo uma língua também. A gente , os meus menino que ficam aqui, meu banqueiro, eles não cumpreende quase o que eles falam na língua né. Então, por isso que eles cantam na língua, muitas vezes eles num tentam copiar pra nós aprender por causo que eles falam na língua deles. Alguns pedaços, que outros guia vem que eles já copiam ´pra gente aprender, cada vez que ele venham é um pedaço que eles ensinam.

G: Eles assim, tem algum guia especifico?

EE8: tem.

G: Ééé...

EE8: Tem,

G: E como seria o nome desses índios?

EE8: É Mané Catú.

G: Manéia?

EE8: Mané Catú;

G: Mané Catú..

EE8: Mané Catú e o coira também o, o, deixa eu se lembrar (riso), seja no ,.. tem outros guia também, se eu chamar, a Geelle vai correr da porta , vem o Pena Verde também, tem o seu.....e o deixa eu lembrar, se eu chamar e baixar guia vai já falar feio com a senhora(risos).

G: Pode, pode, é fique a vontade...

EE8: Tem também o outro que índio né, que é o Pereira, ele fala português que baixa aqui com nós. Também tem o Tupinambá que ele é índio também, veterano indígena. E tem o Sete Flecha né...

G: Índio Guerreiro.

EE8:Tem o Índio Guerreiro também, baixa aqui também.

EE8: Tem o Índio Guerreiro....

G: Índio da Cachoeira?

EE8: Não, Índio da Cachoeira não.

G: Baixa aqui?

EE8: Não.

G: Mas ele não é dessa linha?

EE8: É, mas a gente não chama ele.

G: Só os que estão aqui.

EE8: Só os que tão mesmo. Eu sei só o que vem da mata.

G: Da mata?

EE8: Da mata.

G: E da água?

EE8: Da água, ainda tem sóóó de, da água é o José de Ribamar......

.....(Pausa)

G: hurum....

EE8: Zé Pretinho...., Sacacá, Sacaquinho, Mariana...

G:Índia também?

EE8: Não, que vem da água, e também tem óóó, Agripide ....., (pausa),Maria Joana, Mariazinha,...., e tem mais (risos)

G: Tem mais.

EE8: Tem...

G: Mas desses aqui alguns são indígenas?

EE8: Não. Indigena só esses daqui....

G:Só aqui os da mata, da agua nenhum?

EE8: Só os da mata, da água não

G: Mas todos eles participam?

EE8: Todo eles participam

G: Entendi.

G: Quando a senhora chama um guia, é, a senhora chama só um o eles vem vários?

EE8: Não, quando aqui tá fechado a mesa, ai quando a gente abre a mesa, a gente usa as três, dois, três cigarros.

G: A mesa é a batida?

EE8: É.

G: Então ainda agora a senhora bateu?

EE8: Bati a mesa pra lembrar, ai é cruzado dois cigarro aqui pra abrir a mesa. Dois cigarros aqui, dois ali e dois lá. Aí a gente vai fazer depois da mesa, cantar a doutrina da mesa que é pra fazer a abertura, que tanto quanto a gente vai fazer a abertura em terra, quanto a gente vai fechar no mar. E também fechar as de terra, pra que também não possa vim outros guia que não for preparado para vim pra essa mesa. Então a gente, por isso que eu gosto de vim aqui de conversar, por que qualquer coisa que me esqueço eu bato aqui que já lembro. É assim, então a gente.

G: Que foi o que a senhora fez né?

EE8: Foi o que eu fiz, por causo que era pra lembrar o nome do, dos que senhora queriam, que, tem mais, que vem de terra também que é indígena, ele vem também, e são vários, não são só esses que....

Na minha linha baixa 68 guias, só na minha linha, na dele mais 68, na outra mais 68, são varias guias que vem pra fazer esse trabalho. Aqui trabalha, Rompimato de Umbanda, Flecheiro, que é esse nome que tenho ai. O chefe da minha linha é Rompimato, Rompimato que é o chefe dessa linha, o Flecheiro é a segunda pessoa de Rompimato que ele, que eles que são, que comanda o meu terreiro e a mesa, que são responsavi da mesa, como eu sou sacaca aqui, eles são responsável pelo território....

[00:20:05]

G: E a senhora herdou esse dom, esse cuidado de alguém, de outro especialista da família?

EE8: É acho que do meu avô ele era um pajé, ele morreu , e meu avô é enterrado no final rio Mari -Mari, que aqui, lá nas cabeceiras que, ele é interrado lá. Acho que esse dom já veio da minha família, da família do meu pai, da família da minha mãe, que minha mãe, minha avó por parte de mãe ela tinha esse dom, meu avô tinha esse dom. Então, esse dom a gente vê que, vai passando de filho pra filho, de neto pra neto e vai indo né...

G: Seu filho tem esse dom?

EE8: Meu filho tem esse dom....

G: Hurum...

[00:20:40]

G:Esse cuidado se a senhora fosse fazer pra alguém mordido de cobra, a senhora faria em que momento e por quanto tempo a senhora faria?

EE8: Eu ia fazer não por muito tempo, uma semana, né a gente poder fazer esse cuidados ai pra que não, o nosso parente não passasse nada.

[00:21:146]

G:E a senhora faria ele isolado ou em conjunto com o cuidado médico?

EE8: Não, fazia ele conforme a gente né conversasse com o médico, porque aqui na minha comunidade a gente tem uma parceria , tanto com o medico quanto com o nosso pajé. Tanto como médico cuida, como tanto nós. Então, essa parceria é nossa, que nós tem noção dele. Tanto que nós respeita eles, tanto quanto eles respeita nós. Então, nós temo uma parceria aqui, Então. eu poderia fazer tanto o nosso remédio, tanto o remédio dele. E se ele chegasse que nem o dele...

[00:22:01]

G: Os recursos que a senhora utilizaria nessa prática, eles são de fácil acesso, encontra com facilidade?

EE8: Encontra.

[00:22:13]

G: Quanto tempo a senhora levaria pra encontrar?

EE8: Cinco minutos.

G: Vocês tem aqui no caso?

EE8: Temo aqui..., dava uns cinco minuto por que eu tinha que fazer, é pilar pra tirar só o sumo pra passar na área. Mas, acho que pra conseguir tudinho, eu acho que 3 minutos, porque aqui, cada uma vai ajudando, pegando uma folha dali, outro vai pegando outra daqui e outro dali, outro vai trazendo, outro vai pilando, tirando o sumo. Então, eu boto 5 minutos por que pra dar mais um pouco de tempo.

G: Seria uma ajuda em conjunto.

EE8:Ajuda em conjunto.

[00: 22:53]

G: Se a senhora tivesse de usar algo da cobra né, ou se a senhora tivesse ouvido falar que alguém usou, o que da cobra usaria no cuidado com alguém que foi mordido?

Usaria algo dela, por exemplo: o couro, a banha ou a carne da cobra, ou o dente, o que a senhora, usaria algo dela?

EE8:Se tive, não....

G: Pra curar a própria pessoa..

EE8: A própria pessoa é, por zemplo se tivesse, chegasse aqui, tivesse a cobra, a gente ia usar primeiramente o couro da cobra, pra fazer a defumação. Usava o couro dela, butara num, não só do couro da cobra, mas como pelo da paca, pelo do macaco prego, pelo do coati pra que com esse dentro duma bacia, dum caco pra fazer defumação da própria cobra.

G: Tem alguma parte assim, alguma regra pra defumação?

EE8: T em, não poderia defumar assim, no meio de muita gente, só poderia tá o meu paciente e outro que poderia está nos ajudando. E também o ponto principal desse dai, só quem cuida da mordida de cobra, aquele vai cuidar, até ele ficar bom, até o final, outro não pode diser: eu vou passar. Não. É, aquele que cuida vai até o final.

[00:24:18]

G: E pra defumar, tem alguma parte especifica do corpo? Por exemplo, a senhora defuma só onde foi mordido ou a senhora defuma o corpo todo, ou tem alguma parte assim, da cabeça pra baixo ou da cabeça pra cima?

EE8: Defumaria o corpo todo, porque, se ele ta mordido da cobra, já tá um pouquinho demorado, o veneno já esta transpassando no corpo da pessoa, então tem que fazer o corpo inteiro. Eu não vou fazer só onde tá mordido, por que o corpo inteiro já tá com o veneno dela, então eu tenho que fazer no corpo inteiro.

[00:24:47]

G: E quanto tempo duraria a defumação?

EE8: Até terminar o caco.

EE8: Especifico não, terminar tudo que tano caquinho, acabar a fumaça, todinho e ele receber a fumaça. Não tem tempo não, não sei dizer, 5 minutos não, tem que terminar.

G: E quando tá defumando, faz é, algum benzimento?

EE8: Faz, a gente benze, conforme a gente ta defumando a gente ta benzendo pra sair metade do veneno da cobra e tem assim um ritual que canta quando tá assim com a mordida da cobra, que a gente tá fazendo a defumação, pra fazer a benzição, a gente canta.

(Canto)

Defuma, defuma, defuma você meu irmão

Defuma, defuma, defuma pra sair esse...

Se for veneno de cobra, a gente vai falando e ai vai espanando pra que saia dele né, então esse é o ritual.

[00:26:02]

G: Nesse momento ele tá de olhos abertos, fechados, tem algo?

EE8: Não, se ele tiver olhando é ate melhor ainda, por que a gente ta vendo que ele ta se sentindo bem ne, se tiver com os olho fechado, a gente não ta olhando pra que saber se ele tá se sentindo bem né. Então a gente aconselha que teja olhando pra gente, tambem cantando o próprio ritual.

G: Enquanto a senhora canta a pessoa canta também?

EE8: Canta também.

[00:26:29]

G: E, a senhora tem é mais algo relacionado com um cuidado pra pessoa que foi mordida de cobra que a senhora não tenha dito, após as perguntas ou algo que a senhora queira no seu relato?

EE8:Não, acho que não. Já falei, mas do que devia, falei que sabia falei (riso), de outras coisa tem não.

G: Obrigada (...)

**ENTREVISTA 9**

**Caracterização do Entrevistado**

**Idade:** 86 anos

**Especialidade:** Benzedor e rezador de espinha

**Informações Relevantes:**

**a)** O entrevistado devido a idade demonstrou ter dificuldades de compreender as perguntas que estruturavam o roteiro de entrevista.

**b)** Na experiencia pessoal do próprio entrevistado ele também utilizou pólvora durante a ocorrência da mordida de cobra.

**c)** Como recurso da própria cobra o entrevistado utilizou o fígado e aplicou como emplasto sob a lesão.

d) Outra menção sobre os recursos provenientes da cobra, o especialista citou o uso da banha da cobra utilizados em feitiços (judiaria).

**Transcrição da entrevista**

[00:00:01]

G: (...), me conte se existem histórias, lendas, mitos ou contos com cobra em seu povo.

EE9: Rapaz por aqui, só antigamente o… que… aquele que chamavam Honorato, cobra grande, era cobra ele, nasceu cobra ele. Jogaram no rio, ele cresceu, virou cobra, no rio ele virou cobra. Mas ele já nasceu cobra, ele e a irmã dele. Eram duas, um casal. E aí, contaram que eles tiveram uma briga com eles la, ela era brava né, queria comer os outros… ele pegou, lutou com ela, lutou…até que venceu a irmã dele, matou ela e só ficou ele.

G: Mas é… eles são de nascimento de alguma pessoa? De alguma mulher daqui da aldeia?

EE9: É… disseram que era uma de fora aí, nasceu pra lá né. Nasceu por aí, madeira por aí… pra lá que eles nasceram. E ainda até hoje, ainda tem… tem aí no… aqui nesse bem assim ai…. Tem um encantado aí.

G: Uma cobra?

EE9: É…

G: É… tem aqui. Ela é filha do seu, do seu… morava aqui no Fontenele. Como então, era o nome dele meu Deus? Ela foi, tava lavando roupa, aí só viu aquele rebojo, quando correram pra lá, tava só aquele negócio no meio, a bicha, ela levou com corpo e alma, ela é… e ela ficou encantada aí. Até hoje ela vive aí, tá naquele poço. Era branca o nome dela.

G: Branca?

EE9: Hamham.

G: Ela era aqui da aldeia?

EE9: Ela era daí, daqui mesmo, lá do Fontenele (...)

G: E as pessoas viam ela?

EE9: Agora? De vezes em quando é ela aparece, bota gente pra correr, mas ela não faz nada não, só pra espantar mesmo.

G: É né…

EE9: Já viram ela uma diversas vezes, só que ela já falou com um camarada pra desencantar ela, o camarada não teve coragem de ir. Mas desde que ela disse, tão fácil. Quando ela viesse meia noite, era pra ele tá com ovo na mão dele. Ela vinha tufando a água, tufando a água… quando ela boiasse, ela ia abrir a boca, ele ia jogar o ovo dentro da boca dela. Mas não teve coragem não.

G: Não teve coragem?

EE9: Não…Ela ia pegar nele por aqui, jogou…

G: E ela encantada, ela vive num mundo então, embaixo da água?

EE9: Embaixo da água. Ela vive no fundo lá.

G: E tem uma cidade então, embaixo?

EE9: Tem uma cidade com certeza, é…

[00:03:36]

G: E o senhor já teve alguma experiência com cobra, aqui na aldeia?

EE9: Cobra… qualquer uma cobra? Rapaz por aqui, a gente só tem experiência de, assim de… por exemplo morder ou a pessoa fazer o remédio… as vezes dá certo.

G: O senhor já foi mordido alguma vez?

EE9: Já senhora.

G: Me conte como foi.

EE9: Eu… aqui foi… nunca tinha sido mordido por uma bicha, por uma serpente. Logo chegamos pra cá, foi eu e a Isabela… vamos tirar uma cabeceira Isabela, aqui nesse garapé aqui. Aí vinha passando assim, tinha um pau dessa altura, e uma moita, aí passei mão, meti essa perna, suspendi essa aqui, só fez... só sentir aquele ardume, um lagarto me ferrou. Espia se, não é minha vista, amodo é uma lagarta. Por lagarta me ferrou, andei como daqui pra li, encherei a Isabela desse tamaninho já, depois espiei bem mesmo, isso foi uma cobra que me mordeu… ai eu olhei, ai limpei a vista, aí fui pra lá, aí cheguei ela tava la no galho do pau. Ela era uma desse tamanho assim. Desgraçada tu vai me matar, mas eu vou te matar, aí eu matei ela, tirei a parte da barriga dela, tirei o fígado e emplastrei em cima, bem aqui mesmo.

G: Do fígado da própria cobra?

EE9: É da própria cobra, eu botei. Pra não, pra não…, mas eu não cheguei andando não mana, cheguei engatinhando, igual criança. Embarquei na canoa, rum… a bicha arde, não dói não, arde. Ardeu que quase ela me matava, essa desgraçada. Por isso, que eu não gosto dessas cobras não. E aí me levaram pra Nova Olinda, passei oito dias lá no hospital.

G: E o senhor ficou com alguma sequela?

EE9: Hãn?

G: O senhor ficou com alguma sequela?

EE9: Essa daqui não pode pegar nada, olha como fica. Aqui entrou um estrepe, o estrepe quebrou o pedaço, ficou por lá, o bicho ficou parece que tá encasacado aí o, ela mordeu bem aqui em cima.

G: Exatamente aí?

EE9: Aham…

G: E o senhor é… quando foi mordido, o senhor usou algum remédio caseiro?

EE9: Usei só… daquele tempo meu filho ainda tavam pequeno, esse que tá pra ir. Ele também, ele também trabalha bem de remédio caseiro. E nesse tempo ainda tava pequeno ainda, tomei só pólvora.

G: Pólvora?

EE9: Aham… coloque na água e tomei.

G: E precedeu se teve alguma melhora?

EE9: Aliviou mais um pouco, deu pra mim chegar pra Nova Olinda. E aí foi só isso aí…

[00:06:55]

G: As cobras elas possuem algum significado pro senhor?

EE9: Cobra… significado? Eu não tô entendendo. Como é?

G: Ela tem alguma importância, alguma serventia no seu dia a dia, as cobras?

EE9: Pra mim, ela não tem não. Porque ela é braba com a gente, ela é perigosa.

G: É na etnia munduruku ela tem algum símbolo assim, ela é simbolizada de alguma forma, a cobra?

EE9: Como é que a senhora quer dizer?

G: Se ela tem alguma importância na história da etnia, tem algum valor?

EE9: Rapaz pra muitos tem valor, mas pra mim, não tem. Pra outras tem, mas pra mim não.

[00:07:59]

G: E no dia a dia elas servem pra alguma coisa?

EE9: Ela… aqui a banha é bom contra judiaria, ela… tira a banha, o pessoal toma. A pessoa que tá judiado, toma…, bota pra fora. Não sei quem foi, que uma vez aqui me deram uma colherada, mais é enjoado meu amigo, rum… eu tomei. Eu acho que foi isso que me deu essas doenças, essas doenças por exemplo. Eu tenho um negócio aqui no braço, aqui na minha pele, desde aqui, mas desse lado…. Não pode apertar assim, e nem esfregar assim com força, que fica roxo, aqui, isso aqui. Aqui um dia desse, eu mandei puxar o meu braço ali, o rapaz pegou aqui, parceiro não faz isso, vai roxear meu braço, ele soltou, olha como ficou, já tava sentando de novo.

[00:09:17]

G: Quais is tipos de cobras o senhor conhece?

EE9: É… eu conheço aqui na minha vista, é… eu conheço o surucucu, a pico de jaca, surucucu de fogo, ela é tudo vermelha ela é. Uma vez foram caçar pra li com os cachorros, mordeu cinco cachorros nosso. E cobra, quase a grossura daquele pau ali ó, aquele esteio ali, desse cumprimento aí, mordeu todos os cinco. Morreu um, mas não morreu nenhum, o remédio tava aqui, deixei o remédio pros cachorros, não morreu nenhum. Deixou um negócio, ele sabe onde os remédios aí, os remédio isso sabe. E aí a gente…

[00:10:25]

G: Dessas cobras, qual é a mais perigosa, qual o senhor considera?

EE9: A pico de jaca.

G: A pico de jaca?

EE9: É…

[00:10:35]

G: Na linguagem munduruku, elas têm algum nome específico?

EE9: Acho que tem, tem, mas eu não sei pronunciar.

[00:10:49]

G: E o senhor realiza ou já realizou alguma cuidado com alguém que já foi mordido por serpentes?

EE9: Eu? Só minha mulher que foi mordida lá pra cima… essa me deu um pouco de trabalho, mas ficou boa.

G: Mas o senhor deu alguma coisa pra ela tomar?

EE9: Dei… apliquei nela aquela injeção antiofídica.

G: Antiofídica né?

EE9: HumHum… isso eu apliquei nela.

G: O senhor mesmo aplicou?

EE9: Eu mesmo. Nesse tempo não existia, pra cá não existia polo base, essas coisas… mas a gente sabia aplicar.

G: Sabia aplicar né?

EE9: Hum Hum.

[00:11:34]

G: O senhor poderia me relatar, quais cuidados o senhor realiza, como o senhor realizou, o preparo… o senhor falou que quando foi mordido, tomou pólvora né. Como foi que o senhor preparou?

EE9: Botei dentro da água, eu tirei o cartucho, tirei a pólvora, botei dentro, bati bem batidinho com um pauzinho, dentro da cuia, aí tomei.

G: Além da pólvora o senhor também tomou mais alguma coisa?

EE9: Não.

G: Não?

EE9: Não tomei não. Já foi lá no hospital, tomar injeção.

[00:12:19]

G: E esse cuidado que o senhor fez com a pólvora, o senhor herdou de alguém ou o senhor aprendeu com algum especialista?

EE9: Eu via sempre quando tavam, quando tão conversando assim, eu tô escutando né. Aí o homem tava dizendo, rapaz não tem, pra não batalhar muito com a cobra. Disse ele, escangalha um cartucho seu, tire a pólvora, bote num pouco d’água, e toma que mata a força do veneno. Aí foi assim que eu aprendi.

[00:13:00]

G: E o senhor poderia me relatar, quanto tempo o senhor levou pra preparar, essa pólvora na água?

EE9: Coisinha de minutos… uns cinco minutos.

G: E o senhor fez logo após a mordida?

EE9: Foi, logo após a mordida logo.

G: E quando voltou de Nova Olinda, ainda chegou fazer mais alguma coisa?

EE9: Não.

G: Não rezou, não benzeu?

EE9: Não.

G: Não.

EE9: Não senhora.

G: Não puxava, massagem?

ER9: Agora puxação, a mulher sempre puxava.

G: Ela puxava com que?

EE9: Com banha, banha mesmo de azeite doce, ela puxava.

[00:13:46]

G: Da cobra ela usou alguma coisa, pra fazer essa massagem?

EE9: Não.

G: Não? Nem a banha?

EE9: Nada, nada…

[00:13:54]

G: Quando o senhor realizou esse cuidado, o senhor realizou sozinhos ou realizou junto com o cuidado médico?

EE9: Quando…

G: Quando aconteceu com o senhor e quando cuidou da sua esposa…

EE9: Agora o meu, só mesmo eu com a mulher e a enfermeira lá.

G: Ela tava presente, a enfermeira?

EE9: Tava. Eu mesmo que ajustava pra levar ela, pra cá, pra li.

G: No caso dela, foi só o senhor que…

EE9: Sim.

G: E o que o senhor fez?

EE9: Fiz… disseram pra mim comprar uma pílulazinha assim, uma cumpridinha, tetraciclina, comprar pra dar, pra desinflamar né.

G: Uhum.

EE9: Dava pra ela, pronto, primeira vez melhorou.

G: Além da tetraciclina o senhor usou alguma coisa daqui da aldeia, fez algum chá?

EE9: Não.

G: Não? Não deu nada pra ela tomar?

EE9: Não. Nada pra tomar não.

[00:15:14]

G: Quando o senhor fez, os recursos que o senhor fez, era fácil de encontrar?

EE9: O recurso?

G: É… a pólvora era fácil?

EE9: É, era fácil.

G: Era?

EE9: Era.

G: E quanto tempo o senhor usou essa pólvora, quantas vezes o senhor tomou?

EE9: Eu tomei uma vez

G: Uma única vez?

EE09: Só

[00:15:37]

G: Entres esses recursos, é o senhor já… usou ou já presenciou algo que foi usado da própria cobra pra realização da pessoa que foi mordida por cobra?

EE9: Não.

G: Não? Já soube de alguém que usou por exemplo, algo?

EE9: Não. Já…

G: Tipo o que?

EE9: Eles é… por exemplo, a cobra morde… ensinaram diferente. Diferente não, não caso usar uma faquinha, furar na cesura dela, aí espera até sair o sangue, o cara não sente nem dor de cabeça. Até o rapaz falou, porque tu não fizeste logo. Eu disse, rapaz eu não me lembrei, senão tinha feito mesmo, adormece tudo o pé da gente.

G: Faz um cortezinho?

EE9: Cortezinho e espreme pra sair o sangue, sair o sangue preto.

G: E dessa vez o senhor falou também, que senhor usou o fígado dela foi?

EE9: Foi, agora isso daí eu usei.

G: Como foi?

EE9: Parti ela né, tirei o fígado dela, misgalhei, botei em cima e amarrei com…

G: E foi da mesma coisa cobra que lhe mordeu?

EE9: Da mesma cobra que me mordeu.

G: É? Quanto tempo o senhor levou pra preparar esse…

EE9: Levei mais ou menos uns cinco minutos, aí fiquei ruim pra andar, andei daqui pra aquela rama ali… de lá não consegui mais ficar em pé, fui engatinhando até chegar na canoa, na canoa que me lembrei da pólvora pra tomar.

G: E o senhor é… quando colocou esse fígado, o senhor é… colocou ele natural mesmo?

EE9: Natural. Só fiz mesmo tirar ele, e quebrar ele assim, e botar em cima.

G: E ficou por quanto tempo?

EE9: Ah, uns… até chegar aqui em casa.

G: Até chegar em casa?

EE9: Foi uns vinte minutos.

G: Com o fígado na cesura?

EE9: Com o fígado na cesur.

G: Quando foi pra Nova Olinda, já não tava mais.

EE9: Já não tava mais.

[00:18:33]

G: Entendi… o senhor assim, algum cuidado que o senhor ouviu de familiar seu ou de algum outro especialista que use pra…quando a pessoa é mordida de cobra, que o senhor conheça?

EE9: Rapaz… por aqui não sei não. Se não esse curumim que ele…

G: Ele entende…

EE9: Ele Entende. Ele faz o remédio, quero ver se não mau ficar bom mesmo, só se quiser.

G: É nem vai pra Nova Olinda, só aqui?

EE9: Não.

G: Tá bom (...). Muito obrigada.

**Entrevista 10**

**Caracterização do Entrevistado**

**Idade:** 63 anos

**Especialidade:** Pegadora de desmentidura e Parteira

**Informações Relevantes:**

**a)**  A entrevistada relatou que já utilizou recursos da própria serpente para pegar desmentitura e em mordidas de cobras utilizar a parte da cabeça para passar no local afetado e o couro para defumação.

**b)** Em ocorrências de acidente ofídico a entrevistada relatou ter conhecimento do uso da castanha ralada.

**c)** Ela informou também que é proibido mulheres menstruadas, grávidas e com o olhar maldoso/venenoso visitarem pessoas mordidas que se encontram em recuperação.

**Transcrição da entrevista**

[00:00:01]

G: Me conte se existem histórias, lendas, contos, é… ou mitos com cobras em seu povo?

EE10: A cobra quando morde pessoa, ela é venenosa né. Um tipo de cobra, a surucucu é mais venenosa que tem no mundo. É aquela que quando morde a pessoa, se não se cuidar ela mata. Mata, porque ela é venenosa, a surucucu. Agora a jiboia, a jiboia não é venenosa. Ela trai, trai a pessoa, por exemplo, o caçador vai aqui né, no mato… se ele dar aquele volta, ela a cobra… ele vai embora, e volta de novo, e vai procurar a cobra tá no buraco… Essa é a jiboia que chamam. A jiboia traideira ela, trai a pessoa, trai caça.

G: É… aqui na aldeia, a senhora já ouviu alguma história assim, de lenda ou de mito ou de conto?

EE10: [silêncio....]

G: Se alguém foi é… por exemplo, alguma história de alguma cobra grande, de alguma cobra assim, que anda aqui pela aldeia, ou algum encantamento?

EE10: Da cobra?

G: É!

EE10: É, a cobra grande, ela vevi no rio né, a cobra grande. Ela trai também a gente, motor, e voadeira, gente mesmo… ela tá no fundo. Aquilo ela vai trair a gente de noite né… a gente ver, nós… do primeiro meu marido nós ia, nós ia morrendo aí oh, bem aí logo. Quando nós vimos a cobra focou no nosso rumo, ela ficou perto mesmo de nós, pra puxar nós. Felizmente ela não puxou nós, porque o homem apagou a luz, da canoa e nós fomos… Mas tem cobra grande no rio, existe isso aí.

G: A senhora já viu?

EE10:É já.

G:Já?

EE10: Já vi… Meu marido, esse homem aí, também lá… ia puxando ele, era cobra grande. Agora essas cobras, interra né, tem umas cobrinhas aí, que não faz mal a ninguém. Ela, só que ela gosta de chupar ovo de galinha, ela come lagarta que tem nas frutas, nos mato, isso daí tudo ela come. Mas tem umas que são venenosas, que são… a jiboia não morde, essa tal de chama… papa ovo ela não morde, mas ela lamba a gente, lamba… e as outras mais pequenas assim, não faz mal a ninguém, mas ela morde, morde mais não é muito assim como a surucucu… a surucucu que chamam, surucucu pico de jaca, essa daí que são mais venenosa que tem no mundo, que chamam tem essa daí.

G: Tem assim, algum cuidado com a mulher, em algum momento quando ela tá na fase assim, de menstruação aqui?

EE10: Tem!

G: Tem? E como é?

EE10: É… a mulher quando ela está menstruada, ela não passa onde a cobra tá, ela vai se apanhar, apanha mesmo…

G: A cobra ataca ela?

EE10: Uhum… ataca

G: Por quê?

EE10: Porque a mulher quando está nos tempo dela, da menstruação né, ela tem raiva da mulher, por ser… ela passa aqui pra beira a cobra, e a mulher vem, ela vai buscar onde ela tiver. E outra, a mulher mijar no lugar dela, vai buscar onde ela está, e lamba

G: Mijar?

EE10: Mija no lugar onde a cobra passa. Por exemplo dona (...) mora bem lá né, se ela vai pra trás, fazer… mijar né, aí naquela hora a cobra passou, ela vai buscar ela na rede. Mete a cabeça na terra e oh, porrada nela… é… nós já vimos, e tem muita gente que conta. Meu pai era homem, minha mãe contava, minha tia contava

[00:04:05:]

G: A senhora já teve experiência com alguma cobra aqui na aldeia?

EE10: Não!

[00:04:11]

G: E as cobras possuem alguma utilidade pra senhora?

EE10: Também não.

G: Mas tem serventia pra algo?

EE10: Tem umas né, essas cobras d’água que chamam, a sucuriju. A sucuriju tem a banha, a banha do sucuriju é pra desmentidura, é pra isso…

G: A senhora já pegou desmentidura com ela?

EE10: Já!

G: E já?

EE10: Faz aquela pomada com a banha da sucuriju, aí pode puxar.

G: É uma pomada é?

EE10: Não… a gente faz a pomada com a banha dela

G: E como é que faz essa pomada?

EE10: Pega a banha do sucuriju… surucucu. Sucuriju é um, é surucucu é outro. Ele é… sucuriju, ele também não morde. Ele traga mais sobre galinha, cachorro, gato essas coisas assim de terra. De lá vai pra água. Então, a banha dele serve pra muita coisas. Pra cair dente é bom, pra dor nos ossos é bom com pomada, e pra pegar desmentidura essas coisas mais. Faz aquela pomada, e pode misturar. Tenho certeza, porque nós já conseguimos essas banhas né. De jiboia, também ela é uma banha muito… pra fazer remédio. Pomada, aqueles outros de óleos, andiroba, copaíba. Esses outros em diante… pode fazer aquela pomada, e passar no corpo da pessoa, que na hora fica bom.

[00:05:54]

G: Ela tem algum significado pra senhora, a cobra?

EE10: Se tem? Tem…

G: Tem?

EE10: Tem…

G: Qual o significado?

EE10: Dela por causa, se a gente for mordida com a cobra, o remédio da gente, é injeção, é castanha ralada. A senhora sabe o que é castanha, né. A castanhas ralada, a injeção, e sorva daquele… de cupu. A casca de cupu, pode fazer e passar em cima. Leite…

G: O quê que melhora?

EE10: Melhora a dor.

G: A dor?

EE10: A dor. Ele fica todinho… ele fica inchado né. Puxa, puxa…

G: Desincha também?

EE10: Desincha…

G: É?

EE10: A castanha…

G: A senhora já puxou também com… uma pessoa mordida?

EE10: O meu marido aí… esse daí… foi mordido, duas, três vez cobra.

G: E a senhora fazia esse cuidado?

EE10: Eu fez pra ele. Aí foi em Nova Olinda, tomou injeção né. Aí quando foi uma festa lá, bebeu cachaça, não morreu, não morreu não. Meu irmão, aí de trás, mordeu foi o surucucu, ficou com a perna inchada. Nós acabamos com a banha da jiboia passando nele, ficou bom…

G: A própria banha da cobra a senhora cuidou quem foi mordido?

EE10: Quem foi mordido…

[00:07:19]

G: Quais os tipos de cobras a senhora conhece?

EE10: A banha dela é… o surucucu, da jiboia e do sucuriju.

G: É?

EE10: Três qualidades de cobras.

G: Mas a senhora conhece alguma outra cobra além dessas?

EE10: Não

G: Só essas mesmo?

EE10: Só essas mesmo. Essas aí. E a cobra que é do rio. É muito feia essa cobra aí, eu nunca vi, mas a gente tem a lenda né. A gente ver, que fala, eu já vi o foco dela, eu também já vi.

[00:07:52]

G: A senhora é… sabe falar é, o nome de cobra em Munduruku?

EE10: Não.

G: Não?

EE10: Aqui só quem sabe falar é o meu irmão, que é o professor da Marina.

G: Ele sabe?

EE10: Anham… Nome de cobra

[00:08:09]

G: É? E a senhora poderia me relatar quais cuidados a senhora é, realiza por pessoas envenenadas por serpentes?

EE10: É… a mulher grávida não poder olhar, a mulher menstruada não pode olhar, essa… estes dois tipos, mulher grávida e mulher menstruada.

G: Por que que não pode olhar?

EE10: Por causa que é venenosa, essas são venenosas. A cobra quando morde, não é toda pessoa que pode visitar o doente, com mordido de cobra. Então, se for por exemplo dona Geale, ela não é venenosa, vem chegando com mordida de cobra, ela vai fazer o que? Cuspir em cima, pode cuspir em cima, e deixar que nunca faz mal. Isso daí …as lendas…

[00:09:00]

G: Esses cuidados a senhora aprendeu com alguém da sua família ou com algum especialista?

EE10: Aprendi com meu pai e minha mãe.

G: Eles que lhe ensinaram?

EE10: Foi.

G: Eles cuidavam das pessoas que eram mordidas é?

EE10: Minha mãe e meu pai. Meu pai falava gíria, minha mãe ela não falava, ela era branca. Meu pai era Munduruku mesmo.

[00:09:24]

G: Esse cuidado que a senhora fez pro seu esposo e com as pessoas, a senhora fez como e quanto tempo? Quanto tempo a senhora cuidava dessa pessoa?

EE10: Até quinze dias.

G: Até quinze dias? Direto?

EE10: Direto…

G: Sempre passando a banha?

EE10: Passando a banha e fazendo puxação né, esquentando com água morna, e sem comer as coisas remosas também. E deixado ele assim, numa reserva onde não passava gente assim, menstruado, onde passava gente venenoso de olhar. Até trinta dias, eu cuidava dele lá, mas trinta dias ele não saía do quarto, mesmo que mulher ter neném, ela é igualmente uma mulher quando dar à luz numa criança, se resguarda mesmo.

G: E quais são os alimentos que a senhora considera venenoso? Remorso?

EE10: Pra comer?

G: É, quando tá mordido, que não pode, como a senhora falou

EE10: Olha aí é veado, macaco prego, piranha, matrinxã, esses peixes assim não presta. A comida dele já é frango, peixinho pacu, charuto, o tucunaré, Acará, isso daí não faz mal pra mordida desses bichos.

[00:10:51]

G: A senhora realizou esse cuidado sozinha ou a senhora fez junto com o cuidado médico?

EE10: Só eu. Quando ele veio de lá né, de lá de Nova Olinda, eu cuidei do meu marido, desse rapaz, desse velho. Ai do meu irmão, foi minha cunhada que cuidou dele. Mas foi assim, guardado na casa dele.

G: E a senhora percebeu se teve melhora?

EE10: Teve.

[00:11:18]

G: Teve? É… o recurso que a senhora utilizou a senhora encontrava com facilidade?

EE10: Essas banhas?

G: Uhum…

EE10: Eu tinha…

G: senhora tinha em casa?

EE10: Tinha.

G: Mas se não tivesse, tinha como conseguir com alguém?

EE10: Comprava. Por onde tivesse a gente corria atrás né. Ai achava pra comprar.

[00:11:38]

G: E quanto tempo mais ou menos a senhora acha que levaria pra caso a senhora não tivesse em casa.

EE10: Rum… acho que podia até morrer, não era fácil pra achar. Sempre aqui desse lado, os irmãos desse lado, sempre ela matam, a menina guardava, porque ela era pajé né, pajé do outro lado.

G: A senhora tem uma pajé aqui atrás é?

EE10: Tem uma aqui…

G: Quem é a pajé aqui?

EE10: É uma sobrinha minha, o nome dela é Francisca, chama só Chica pra ela. Ela é pajé… e lá do outro tem bem uns quatro…

G: É?

EE10: Eu ia lá comprar. Comprava banha, até de jaca eu comprava banha. Eles matavam, eu comprava, pouco mas eu comprava.

[00:12:23]

G: E desses recursos da cobra, a senhora usou alguma outra coisa? Ai vou dar um exemplo pra senhora, a senhora usou é… o couro pra defumar, ou a senhora usou o dente da cobra ou a cabeça da cobra, ou o rabo da cobra, ou carne da cobra?

EE10: Desse que mordeu ele?

G: É… se a senhora…

EE10: Não

G: Não usou?

EE10: Não

G: Mas chegaria a usar ou sabe algum cuidado?

EE10: Não, só foi mesmo da banha mesmo. Da conta mesmo não cheguei a usar.

G: É? Mas a senhora conhece algum cuidado, assim que é feito com a parte da cobra?

EE10: É… o couro dela quando morde, usa… as pessoas falam né, que quando morde, tira o miolo da cabeça pra passar no cisura e o couro pra defumação.

G: Mas coloca, coloca em cima com alguma coisa ou só o miolo?

EE10: Só o miolo… pega ele, onde tá mordida né. Por exemplo, mordeu na perna, pega o miolo, bota lá com um paninho, amarra e deixa lá. Aí depois que passar umas horas, tira aquele lá, coloca de novo. Umas três vezes.

G: E a defumação é feita como?

EE10: A defumação é botar um cavãozinho no caqui, aí bota a pele assim, e bota qualquer coisa pra fumaçar né. Aí deixa levar hora, até acabar aquele couro.

G: A perna próximo…

EE10: Quando é a perna né. Quando é braço ou outras coisas tem que defumar tudo….

G: Todo corpo né?

EE10: Todo corpo.

G: E quanto tempo defuma?

EE10: Umas três viagens, três vezes no dia. Três num dia, três no outro… uns seis dias assim.

G: E quanto tempo demora uma defumação?

EE10: Demora, por exemplo, é… se eu defumasse sete horas do dia, aí ia defumar já sete horas do outro dia. 24h.

G: Ai quando a senhora faz a defumação, tem mais alguém com a senhora, ou é sozinha?

EE10: Não, é sozinha.

G: É? Mas a senhora faz alguma reza, algum benzimento ou só a defumação?

EE10: Eu oro.

G: A senhora ora é? Orava pra pessoa…

EE10: Orava pra Deus ajudar a pessoa. Ajudar a curar a pessoa.

G: A senhora tem algum cuidado que a senhora não falou durante a entrevista, que a senhora queira acrescentar? Alguma experiência que a senhora teve com cobra?

EE10: Experiência?

G: De cuidado assim, ter visto alguém cuidar, de uma outra forma que não foi como a senhora…

EE10: Outra né?

G: É…

EE10: Já, a mesma coisa que eu fazia, o outra também faz.

G: Eles faziam o mesmo cuidado?

EE10: Faziam… aqui minha irmã, esse meu irmão, que chamam torrado, aí conhecem, foi mordido de cobra. De quando ela veio, aí ela mandou eu… perguntou pra mim o que eu fazia, quando mordeu o Salim (cachorro), aí eu falei… Era… eu defumava, não deixava ele pisar… ainda tem outra, que não presta pisar no carvão, na bosta de galinha, sem sandália. Era toda… em terra, era em na água, era casa não deixava a sandália. Aí eu, dei pra ela, e ela mesmo fez. Porque por exemplo entre uma, dois, três defumação, não pode o outro cheirar a cobra, só a gente que tá tratando dele, do paciente. Isso daí, que eu me lembro né.

G: Tá bom (...), obrigada tá, pela sua participação.

**Entrevista 10**

**Caracterização do Entrevistado**

**Idade:** 63 anos

**Especialidade:** Pegadora de desmentidura e Parteira

**Informações Relevantes:**

**a)**  A entrevistada relatou que já utilizou recursos da própria serpente para pegar desmentidura e em mordidas de cobras utilizar a parte da cabeça para passar no local afetado e o couro para defumação.

**b)** Em ocorrências de acidente ofídico a entrevistada relatou ter conhecimento do uso da castanha ralada.

**c)** Ela informou também que é proibido mulheres menstruadas, grávidas e com o olhar maldoso/venenoso visitarem pessoas mordidas que se encontram em recuperação.

**Transcrição da entrevista**

[00:00:01]

G: Me conte se existem histórias, lendas, contos, é… ou mitos com cobras em seu povo?

EE10: A cobra quando morde pessoa, ela é venenosa né. Um tipo de cobra, a surucucu é mais venenosa que tem no mundo. É aquela que quando morde a pessoa, se não se cuidar ela mata. Mata, porque ela é venenosa, a surucucu. Agora a jiboia, a jiboia não é venenosa. Ela trai, trai a pessoa, por exemplo, o caçador vai aqui né, no mato… se ele dar aquele volta, ela a cobra… ele vai embora, e volta de novo, e vai procurar a cobra tá no buraco… Essa é a jiboia que chamam. A jiboia traideira ela, trai a pessoa, trai caça.

G: É… aqui na aldeia, a senhora já ouviu alguma história assim, de lenda ou de mito ou de conto?

EE10: [silêncio....]

G: Se alguém foi é… por exemplo, alguma história de alguma cobra grande, de alguma cobra assim, que anda aqui pela aldeia, ou algum encantamento?

EE10: Da cobra?

G: É!

EE10: É, a cobra grande, ela vevi no rio né, a cobra grande. Ela trai também a gente, motor, e voadeira, gente mesmo… ela tá no fundo. Aquilo ela vai trair a gente de noite né… a gente ver, nós… do primeiro meu marido nós ia, nós ia morrendo aí oh, bem aí logo. Quando nós vimos a cobra focou no nosso rumo, ela ficou perto mesmo de nós, pra puxar nós. Felizmente ela não puxou nós, porque o homem apagou a luz, da canoa e nós fomos… Mas tem cobra grande no rio, existe isso aí.

G: A senhora já viu?

EE10:É já.

G:Já?

EE10: Já vi… Meu marido, esse homem aí, também lá… ia puxando ele, era cobra grande. Agora essas cobras, interra né, tem umas cobrinhas aí, que não faz mal a ninguém. Ela, só que ela gosta de chupar ovo de galinha, ela come lagarta que tem nas frutas, nos mato, isso daí tudo ela come. Mas tem umas que são venenosas, que são… a jiboia não morde, essa tal de chama… papa ovo ela não morde, mas ela lamba a gente, lamba… e as outras mais pequenas assim, não faz mal a ninguém, mas ela morde, morde mais não é muito assim como a surucucu… a surucucu que chamam, surucucu pico de jaca, essa daí que são mais venenosa que tem no mundo, que chamam tem essa daí.

G: Tem assim, algum cuidado com a mulher, em algum momento quando ela tá na fase assim, de menstruação aqui?

EE10: Tem!

G: Tem? E como é?

EE10: É… a mulher quando ela está menstruada, ela não passa onde a cobra tá, ela vai se apanhar, apanha mesmo…

G: A cobra ataca ela?

EE10: Uhum… ataca

G: Por quê?

EE10: Porque a mulher quando está nos tempo dela, da menstruação né, ela tem raiva da mulher, por ser… ela passa aqui pra beira a cobra, e a mulher vem, ela vai buscar onde ela tiver. E outra, a mulher mijar no lugar dela, vai buscar onde ela está, e lamba

G: Mijar?

EE10: Mija no lugar onde a cobra passa. Por exemplo dona (...) mora bem lá né, se ela vai pra trás, fazer… mijar né, aí naquela hora a cobra passou, ela vai buscar ela na rede. Mete a cabeça na terra e oh, porrada nela… é… nós já vimos, e tem muita gente que conta. Meu pai era homem, minha mãe contava, minha tia contava

[00:04:05:]

G: A senhora já teve experiência com alguma cobra aqui na aldeia?

EE10: Não!

[00:04:11]

G: E as cobras possuem alguma utilidade pra senhora?

EE10: Também não.

G: Mas tem serventia pra algo?

EE10: Tem umas né, essas cobras d’água que chamam, a sucuriju. A sucuriju tem a banha, a banha do sucuriju é pra desmentidura, é pra isso…

G: A senhora já pegou desmentidura com ela?

EE10: Já!

G: E já?

EE10: Faz aquela pomada com a banha da sucuriju, aí pode puxar.

G: É uma pomada é?

EE10: Não… a gente faz a pomada com a banha dela

G: E como é que faz essa pomada?

EE10: Pega a banha do sucuriju… surucucu. Sucuriju é um, é surucucu é outro. Ele é… sucuriju, ele também não morde. Ele traga mais sobre galinha, cachorro, gato essas coisas assim de terra. De lá vai pra água. Então, a banha dele serve pra muita coisas. Pra cair dente é bom, pra dor nos ossos é bom com pomada, e pra pegar desmentidura essas coisas mais. Faz aquela pomada, e pode misturar. Tenho certeza, porque nós já conseguimos essas banhas né. De jiboia, também ela é uma banha muito… pra fazer remédio. Pomada, aqueles outros de óleos, andiroba, copaíba. Esses outros em diante… pode fazer aquela pomada, e passar no corpo da pessoa, que na hora fica bom.

[00:05:54]

G: Ela tem algum significado pra senhora, a cobra?

EE10: Se tem? Tem…

G: Tem?

EE10: Tem…

G: Qual o significado?

EE10: Dela por causa, se a gente for mordida com a cobra, o remédio da gente, é injeção, é castanha ralada. A senhora sabe o que é castanha, né. A castanhas ralada, a injeção, e sorva daquele… de cupu. A casca de cupu, pode fazer e passar em cima. Leite…

G: O quê que melhora?

EE10: Melhora a dor.

G: A dor?

EE10: A dor. Ele fica todinho… ele fica inchado né. Puxa, puxa…

G: Desincha também?

EE10: Desincha…

G: É?

EE10: A castanha…

G: A senhora já puxou também com… uma pessoa mordida?

EE10: O meu marido aí… esse daí… foi mordido, duas, três vez cobra.

G: E a senhora fazia esse cuidado?

EE10: Eu fez pra ele. Aí foi em Nova Olinda, tomou injeção né. Aí quando foi uma festa lá, bebeu cachaça, não morreu, não morreu não. Meu irmão, aí de trás, mordeu foi o surucucu, ficou com a perna inchada. Nós acabamos com a banha da jiboia passando nele, ficou bom…

G: A própria banha da cobra a senhora cuidou quem foi mordido?

EE10: Quem foi mordido…

[00:07:19]

G: Quais os tipos de cobras a senhora conhece?

EE10: A banha dela é… o surucucu, da jiboia e do sucuriju.

G: É?

EE10: Três qualidades de cobras.

G: Mas a senhora conhece alguma outra cobra além dessas?

EE10: Não

G: Só essas mesmo?

EE10: Só essas mesmo. Essas aí. E a cobra que é do rio. É muito feia essa cobra aí, eu nunca vi, mas a gente tem a lenda né. A gente ver, que fala, eu já vi o foco dela, eu também já vi.

[00:07:52]

G: A senhora é… sabe falar é, o nome de cobra em Munduruku?

EE10: Não.

G: Não?

EE10: Aqui só quem sabe falar é o meu irmão, que é o professor da Marina.

G: Ele sabe?

EE10: Anham… Nome de cobra

[00:08:09]

G: É? E a senhora poderia me relatar quais cuidados a senhora é, realiza por pessoas envenenadas por serpentes?

EE10: É… a mulher grávida não poder olhar, a mulher menstruada não pode olhar, essa… estes dois tipos, mulher grávida e mulher menstruada.

G: Por que que não pode olhar?

EE10: Por causa que é venenosa, essas são venenosas. A cobra quando morde, não é toda pessoa que pode visitar o doente, com mordido de cobra. Então, se for por exemplo dona Geale, ela não é venenosa, vem chegando com mordida de cobra, ela vai fazer o que? Cuspir em cima, pode cuspir em cima, e deixar que nunca faz mal. Isso daí …as lendas…

[00:09:00]

G: Esses cuidados a senhora aprendeu com alguém da sua família ou com algum especialista?

EE10: Aprendi com meu pai e minha mãe.

G: Eles que lhe ensinaram?

EE10: Foi.

G: Eles cuidavam das pessoas que eram mordidas é?

EE10: Minha mãe e meu pai. Meu pai falava gíria, minha mãe ela não falava, ela era branca. Meu pai era Munduruku mesmo.

[00:09:24]

G: Esse cuidado que a senhora fez pro seu esposo e com as pessoas, a senhora fez como e quanto tempo? Quanto tempo a senhora cuidava dessa pessoa?

EE10: Até quinze dias.

G: Até quinze dias? Direto?

EE10: Direto…

G: Sempre passando a banha?

EE10: Passando a banha e fazendo puxação né, esquentando com água morna, e sem comer as coisas remosas também. E deixado ele assim, numa reserva onde não passava gente assim, menstruado, onde passava gente venenoso de olhar. Até trinta dias, eu cuidava dele lá, mas trinta dias ele não saía do quarto, mesmo que mulher ter neném, ela é igualmente uma mulher quando dar à luz numa criança, se resguarda mesmo.

G: E quais são os alimentos que a senhora considera venenoso? Remorso?

EE10: Pra comer?

G: É, quando tá mordido, que não pode, como a senhora falou

EE10: Olha aí é veado, macaco prego, piranha, matrinxã, esses peixes assim não presta. A comida dele já é frango, peixinho pacu, charuto, o tucunaré, Acará, isso daí não faz mal para a mordida desses bichos.

[00:10:51]

G: A senhora realizou esse cuidado sozinha ou a senhora fez junto com o cuidado médico?

EE10: Só eu. Quando ele veio de lá né, de lá de Nova Olinda, eu cuidei do meu marido, desse rapaz, desse velho. Ai do meu irmão, foi minha cunhada que cuidou dele. Mas foi assim, guardado na casa dele.

G: E a senhora percebeu se teve melhora?

EE10: Teve.

[00:11:18]

G: Teve? É… o recurso que a senhora utilizou a senhora encontrava com facilidade?

EE10: Essas banhas?

G: Uhum…

EE10: Eu tinha…

G: senhora tinha em casa?

EE10: Tinha.

G: Mas se não tivesse, tinha como conseguir com alguém?

EE10: Comprava. Por onde tivesse a gente corria atrás né. Ai achava pra comprar.

[00:11:38]

G: E quanto tempo mais ou menos a senhora acha que levaria pra caso a senhora não tivesse em casa.

EE10: Rum… acho que podia até morrer, não era fácil pra achar. Sempre aqui desse lado, os irmãos desse lado, sempre ela matam, a menina guardava, porque ela era pajé né, pajé do outro lado.

G: A senhora tem uma pajé aqui atrás é?

EE10: Tem uma aqui…

G: Quem é a pajé aqui?

EE10: É uma sobrinha minha, o nome dela é Francisca, chama só Chica pra ela. Ela é pajé… e lá do outro tem bem uns quatro…

G: É?

EE10: Eu ia lá comprar. Comprava banha, até de jaca eu comprava banha. Eles matavam, eu comprava, pouco mas eu comprava.

[00:12:23]

G: E desses recursos da cobra, a senhora usou alguma outra coisa? Ai vou dar um exemplo para senhora, a senhora usou é… o couro para defumar, ou a senhora usou o dente da cobra ou a cabeça da cobra, ou o rabo da cobra, ou carne da cobra?

EE10: Desse que mordeu ele?

G: É… se a senhora…

EE10: Não

G: Não usou?

EE10: Não

G: Mas chegaria a usar ou sabe algum cuidado?

EE10: Não, só foi mesmo da banha mesmo. Da conta mesmo não cheguei a usar.

G: É? Mas a senhora conhece algum cuidado, assim que é feito com a parte da cobra?

EE10: É… o couro dela quando morde, usa… as pessoas falam né, que quando morde, tira o miolo da cabeça pra passar no cisura e o couro pra defumação.

G: Mas coloca, coloca em cima com alguma coisa ou só o miolo?

EE10: Só o miolo… pega ele, onde tá mordida né. Por exemplo, mordeu na perna, pega o miolo, bota lá com um paninho, amarra e deixa lá. Aí depois que passar umas horas, tira aquele lá, coloca de novo. Umas três vezes.

G: E a defumação é feita como?

EE10: A defumação é botar um cavãozinho no caqui, aí bota a pele assim, e bota qualquer coisa pra fumaçar né. Aí deixa levar hora, até acabar aquele couro.

G: A perna próximo…

EE10: Quando é a perna né. Quando é braço ou outras coisas tem que defumar tudo….

G: Todo corpo né?

EE10: Todo corpo.

G: E quanto tempo defuma?

EE10: Umas três viagens, três vezes no dia. Três num dia, três no outro… uns seis dias assim.

G: E quanto tempo demora uma defumação?

EE10: Demora, por exemplo, é… se eu defumasse sete horas do dia, aí ia defumar já sete horas do outro dia. 24h.

G: Ai quando a senhora faz a defumação, tem mais alguém com a senhora, ou é sozinha?

EE10: Não, é sozinha.

G: É? Mas a senhora faz alguma reza, algum benzimento ou só a defumação?

EE10: Eu oro.

G: A senhora ora é? Orava pra pessoa…

EE10: Orava pra Deus ajudar a pessoa. Ajudar a curar a pessoa.

G: A senhora tem algum cuidado que a senhora não falou durante a entrevista, que a senhora queira acrescentar? Alguma experiência que a senhora teve com cobra?

EE10: Experiência?

G: De cuidado assim, ter visto alguém cuidar, de uma outra forma que não foi como a senhora…

EE10: Outra né?

G: É…

EE10: Já, a mesma coisa que eu fazia, o outra também faz.

G: Eles faziam o mesmo cuidado?

EE10: Faziam… aqui minha irmã, esse meu irmão, que chamam torrado, aí conhecem, foi mordido de cobra. De quando ela veio, aí ela mandou eu… perguntou pra mim o que eu fazia, quando mordeu o Salim (cachorro), aí eu falei… Era… eu defumava, não deixava ele pisar… ainda tem outra, que não presta pisar no carvão, na bosta de galinha, sem sandália. Era toda… em terra, era em na água, era casa não deixava a sandália. Aí eu, dei pra ela, e ela mesmo fez. Porque por exemplo entre uma, dois, três defumação, não pode o outro cheirar a cobra, só a gente que tá tratando dele, do paciente. Isso daí, que eu me lembro né.

G: Tá bom (...), obrigada tá, pela sua participação.

**Entrevista 11**

**Caracterização do Entrevistado**

**Idade:** 84 anos

**Especialidade:** Parteira e pegadora de desmentiruda há mais de 50 anos.

**Informações Relevantes:**

**a)** A Entrevista foi realizada em área externa da residência da especialista e próxima as margens do rio o que dificultou a gravação devido o tráfego dos veículos fluviais (rabeta).

**b)** A especialista relatou que quando seu pai foi mordido de cobra ele utilizou alho nas setes vezes como tratamento.

**c)** A especialista também informou conhecer tratamento realizado com papinha de farinha, misturada o limão assado.

**d)** Durante a entrevista a depoente enfatizou o predomínio do modelo biomédico na aldeia e a ausência das práticas de cura por especialistas no território.

**Transcrição da entrevista**

[00:00:01]

G: Me conte se existe histórias, lendas, mitos, contos com cobras em seu povo?

EE11: Sete, sete, mordida.

G: É? sete mordida de cobra

EE11: Ele não morreu de nenhuma e nem sentiu

G: Mas, só de uma vez ou de várias vezes?

EE11: De várias vezes, o remédio dele era só alho.

G: O que ele colocava?

EE11: Alho

G: Alho? Hum, somente? Mas ele fervia, como ele fazia esse alho?

EE11: Não, não, só batia e colocava em cima

G: Das sete vezes!

EE11: Todas as sete, por causa que ele já sabia qual remédio era bom.

G: E a senhora sabe alguma história assim, daqui do Mucajá, que seja relacionada a cobras?

EE11:Quando não, a gente, quando ela morde a gente, é só sarjar, espremer aquele sangue e faz mal não.

G: Não

EE11: Tem vezes que a gente não tem nem coragem de fazer

[00:01:18]

G: A senhora já teve alguma experiencia com cobra, algum contato com cobra?

EE11: O meu pai a cobra mordeu, mas ele ficou bom, mais era assim, era tanto daqui, do coisa, como tinha aquele específico pessoa, eles faziam o específico pessoa.

G: É aquele específico 33 é que chamam?

EE11: Não, é, não, pessoa é um líquido assim.

G: Um vidrinho assim? sim!

EE11:Hã, hã, específico pessoa fala muito.

G: Pessoa ne

EE11: E aí a gente fazia, aquela papinha de farinha, mistura e coloca em cima, e assa o limão, bota em cima, ai o meu pai, inchou, mas depois desinchou, então, é isso, quando morde a gente toma urina pra provocar o vomito, esse era o remédio da gente.

[00:02:21]

G: E, a senhora, é, tem a cobra com algum significado para senhora, o que cobra representa para senhora?

EE11:Representa um animal mesmo, muito feroz, que Deus o livre se ele ferrar uma pessoa, né? E aqui dá muito. Ainda agora, outro dia

G: E na etnia Munduruku, tem alguma simbologia da cobra, representa alguma coisa na etnia, na história da etnia?

EE11: Só assim, que ela, é porque, mais na mata, também né, tem um tempo que elas entram na mata mesmo, tem um tempo que elas nem morde a gente, que toda assim, com aquelas peles mudadas.

[00:03:27]

G: E a senhora acha que as cobras servem para alguma coisa?

EE11:Como?

G:As cobras servem para alguma coisa no seu dia-dia?

EE11: Meu Deus do céu, para muitas coisas, para muitas pessoas, muita etnia serve sim

G: De que forma elas servem?

EE11: Serve a banha, serve ocolar

G: Colar é como uma proteção

[00:03:53]

G:E a senhora conhece algum tipo de cobra?

EE11: Um pouco.

G: Quais elas a senhora conhece?

EE11: A Surucucu é a mais, é a que mais gente conhece, porque é a que mais tem.

G: Surucuru, né? Além da surucuru, a senhora conhece alguma outra?

EE11: Sucuriju que mora dentro d’ agua, come.......que é uma beleza

[00:04:18]

G: Qual delas a senhora acha mais perigosa?

EE11: A surucucu e surucucurana, essa surucucurana quando ele não mata, ele aleija.

[00:04:33]

G: E se a senhora tivesse que falar o nome da cobra em Munduruku, a senhora ainda lembra como fala?

EE11: Poibú

G: Poibú, sua memória tá muito boa, né!

G: E Surucucu a senhora sabe como fala?

EE11:Não.

G: não, mas as cobras no geral, sim!

G: Poibú, ne!

[00:05:10 ]

G: Se por exemplo, alguém fosse picado por uma cobra hoje aqui, né, ou se a senhora já vivenciou, o que a senhora faria?

EE11: Agora a gente não se preocupa mais com essas coisas, porque agora nós tendo o polo, e é o polo que acolhe, tudo quanto é doença aqui, tem mais quem se preocupe com essas coisas nossa mesmo, nossas ervas. Até celular tem a lancha, e lá está ali a lancha, e lá vai pra la, quando não tem, desce o rabeta, tudo isso que tem, é rabeta, tem lancha, tem bem umas 04 lancha, tem uma da saúde

G: Então hoje é mais a equipe que faz o trabalho.

EE11: Se qualquer coisinha acontecer, é por lá, da maneira que for. Então esse cuidado já é deles, não é mais nosso.

G: Não tem mais aquela prática, assim, de ter o Pajé, o Benzedor, aqui, não?

EE11: Nós temos ainda, alguns lá

G: Aqui no Mucajá, não?

EE11: Não, tem uns pro lado de lá, mas pra ti encontrar ele é meio difícil.

G: Então é mais é polo mesmo.

EE11: Tudo é polo, remédio, tudo quanto é doença.

[00:06:48]

G: E a senhora tivesse que ter alguém mordido com a cobra, tivesse que usar uma parte da cobra, a senhora usaria?

EE11: Não, porque, a gente já tem o polo, chegou lá ,fulano ta assim, chama o agente de saúde, nós tem três agente de saúde, um que mora lá, outro que mora ali e outro que mora la bem, elas tão sendo fácil, então vamos dizer que tirou aquela preocupação, é mesmo que ser das crianças, crianças, mulher que tem nenê, ninguém mais se preocupa mais em casa e tudo no polo e quando não da jeito, leva pra nova Olinda, pra mandar tirar, nasce fora de tempo, manda pra Manaus, e acaba vencendo, nós tem uns menino ali bem que é assim, de 07 mês e acaba vencendo. Aqui nós não temos quase dificuldade, nós aqui, nós sermos umas pessoas meio assim, não depende de muitas coisas, não, porque é difícil, quando acontece.....aqui no mucajá as pessoas inteligentes, meio estudada, tem uma pratica meia boa.....então vamos dizer que, já não é aquelas pessoas que está, correndo pra casa do vizinho, a gente mesmo já caminha, caminha sozinho, eu acho assim que é muito importante professora, a gente saber caminhar só a gente, não se escorar no outro, aqui onde nós mora, nós somos de uma vigilância muito pesada, sobre tudo, tudo, saúde, quando ataca nessas doença contagiosa, ai a Federal não deixava nem encostar aqui, quando deu essa doença que matou esse povo, a Federal ficava era ai, era Nova Olinda e aqui, Nova Olinda e aqui, as vezes ficava por ali, olhando, nós tem dinheiro, vamos dizer que nós temos pessoas que gostam de nós ou se interesse é dele mesmo, não sei, mas que eles são umas pessoas muito assim, prestativas...pessoa muito educada, não persegue ninguém tem muitos que vai falar bom dia, que não está bom, tem muitos professor do Estado, muitos professor do município, tem uns encaminhamento da igreja católica, .....

**Entrevista 12**

**Caracterização do Entrevistado**

**Idade:** 65 anos

**Especialidade:** Parteira, pegadora de desmentidura, rezadora/benzedora e espirita

**Informações Relevantes:**

**a)** A entrevistada informou ter realizado reza em mordida de cobras, inclusive dentro do ambiente hospitalar.

**b)** Em seu relato, a entrevistada mencionou a banha da jiboia e da surucuriju de grande utilidade para tratamentos medicinais na aldeia. Outra utilidade mencionada foi o couro da cobra para defumação e cura de feitiço.

**e)** Em uma experiencia a especialista já curou mordidas de cobra com a folha da palheira,

banha do tijuaçú, banha da paca e da capivara.

**d)** Durante o tratamento em mordidas de cobras ficam restrito a ingestão de alimentos como: Paca, mutum, porco de casa, peixe liso, surubim e pirarara.

**Transcrição da entrevista**

[00.00.01]

G: Me conte se existem histórias, lendas, mitos, contos com cobras em seu povo?

EE12: Hum

G: A senhora lembra de alguma história aqui na sua aldeia, que seu pai ou sua mãe contava ou que a senhora conheça relacionada a cobra?

EE12: Hunrum. Eu lembro duma história que uma criança foi sumida né, sumiu aí de lá ele gerou assim um inseto, o inseto, ele transformou não sei, sei que virou inseto, cobra né. Até hoje essa cobra mora aí, mora abaixo daí do Fontenelle, dessa criança que sumiu, hoje é cobra grande. Eu lembro porque eu vi a criança, quando a gente viajo pro sucundurí essa criança tava atras da costa da mãe dela na prancha, de lá sumiu, a mãe nunca achou.

G: E, essa cobra no caso ela é uma cobra encantada?

EE12: Ela é cobra encantada, é, uma criança homem né, uma criança homem, sumiu.

G: E, quando ela é encantada assim, como que a gente consegue saber que ela foi encantada?

EE12: Porque os mestres né dele que existe ainda, esse negócio de mestre que é os curador ele descobre, descobre que a criança é, foi encantada. O bicho do fundo que leva né.

G: E quando ela é encantada, ela vai para fundo mas é lá tem, como é lá, a senhora sabe me dizer?

EE12: Ela tem a moradia na terra, no fundo. Mora na terra e quando a pessoa sabe desencantar, sabe o remédio para desencantar ele volta de novo a ser a pessoa que era né, já ele já vem crescido, grande.

G: A senhora já conheceu alguém que foi encantado e desencantado?

EE12: Já conheci já, sim.

G: E, como foi?

EE12: Desencantaram ele que ele foi encantado, bicho levou, bicho do fundo levou, que são espírito mau, maligno, levou aí alguém que sabia do remédio para descobrir, com é para desencantar, desencantou e voltou a ser de novo a merma pessoa que era, ele encanta, para encantaria não tem idade, vai, vai mermo.

G: Qualquer idade pode ser?

EE12: Qualquer idade pode ser encantada.

G: E tem algum perigo para ser encantado ou alguma coisa que eu não posso fazer pra não ser encantada?

EE12: Tem, quando a pessoa sabe fazer remédio né, as vezes a pessoa tem o corpo aberto, não sabe rezar não sabe fazer remédio nenhum é quando pego, levo, não sabe se defender.

[00.03.12]

G: E, a senhora já teve experiência com cobra aqui na sua aldeia?

EE12: Hum não, só com remé, só com a banha dela que faz remédio.

G: Como é que, o que que a senhora faz com a banha dela?

EE12: A banha dela a gente faz coquetel para passar em ferimento né, no corpo da pessoa quando tá com ferimento, a gente faz coquetel da banha dela né.

[00.03.42]

G: A senhora é, já cuidou de alguém mordido por cobra?

EE12: Não.

G: Mas já vivenciou?

EE12: Eu já vi só.

G: Como foi?

EE12: Já vi só um que veio para o hospital aí doente, mordido de cobra, aí vieram pegar a gente aqui, fui lá vê, mandaram eu rezar, eu rezei em cima que já estava roxeando a perna do homem, veio do sucundiri ele, aí eu rezei em cima tinha apanhado a vermelha que chama né, aí eu rezei e aí ele ficou aí no hospital não lembro se saiu bom, só sei que rezei pra vermelha.

G: A reza que a senhora fez foi uma reza específica, a senhora lembra?

EE12: Lembro.

G: A senhoria poderia me relatar?

EE12: De ensinar. (risos). Ensino sim, né que a gente sabe né é para ensinar.

G: Sim.

EE12: É, é assim, a senhora vai gravar aí?

G: Sim.

EE12: Caminhando pelo mundo, mundo, encontrei com Jesus Cristo, Jesus Cristo pergunto onde vai São Paulo, vou a procura de remédio pra essas, pra essa doença, ou mordia, sei como for, pra essa vermelha e volte para trás, reze em cima com os poderes de Deus e a virgem Maria, Pai, Filho e Espírito Santo. Entenderam?

G: Entendi. E a senhora faz algum sinal quando tá rezando?

EE12: Sim.

G: É, como é?

EE12: Se benze, Pai, o Filho e Espírito Santo.

G: E a senhora faz sozinha ou pode ter outras pessoas?

EE12: Pode ter outras pessoas, não tem problema não.

G: A senhora reza alto ou só para a senhora?

EE12: Eu rezo alto.

G: A pessoa precisa repetir?

EE12: Nem todos aprende assim rapidinho né.

G: Mas a pessoa repete com a senhora as suas falas ou não, ou só a senhora mesmo?

EE12: Não, só eu mermo.

G: Quantas vezes a senhora faz essa reza?

EE12: Eu faço só uma vez. Quando eu vou rezar é, assim, benzer né.

G: Precisa de vela, alguma coisa ou só?

EE12: Não, não, só para quando é praa batizar criança.

[00.06.03]

G: A senhora aprendeu esse cuidado da reza com alguém da sua família?

EE12: Com a minha mãe.

G: Com a sua mãe?

EE12: Humhum.

G: A sua mãe, ela ...

EE12: A minha mãe ela era pajé.

G: Humrum.

EE12: Pajé, pajé já de nascença né. Ela nasceu, ela já trouxe esse dom com ela.

G: Tem mais alguém na sua família também que tenha esse dom?

EE12: Não.

G: Não.

EE12: Não, só eu e essa minha filha aí, da família, e a (...).

[00.06.36]

G: E as cobras em particular elas possuem algum significado pra senhora? Elas tem alguma importância ou algum valor para a senhora as cobras?

EE12: As cobras têm valor só as banhas dela, da jiboia, surucucurana né, surucucu de fogo é venenosa, surucucu. São três tipos de surucucu.

G: A senhora conhece todas elas?

EE12: Conheço.

G: Conhece.

EE12: surucucu de fogo, é uma vermelha, a jiboia.

[00.07.13]

G: E, elas tem alguma utilidade no seu dia-a-dia, servem para alguma coisa?

EE12: Só pá morder a gente mermo essas outras cobra, para morder pra, quer dizer para dá prejuízo né, elas morde e aí.

G: No tratamento elas servem para alguma coisa?

EE12: Servi. A jiboia, banha da surucucu, a banha da sucuriju.

G: Além da banha a senhora usa mais alguma coisa das cobras?

EE12: Não.

G: Não. Ou usa a carne ou couro?

EE12: Não, não.

G: Mas a senhora já curou de alguém que tenha usado essas partes?

EE12: Já. Já curei já.

G: É.

EE12: Curei dum senhor por nome (...), ele veio mordido de cobra saindo sangue pelo toco do cabelo assim ó, sangue, ele estava mordido de cobra. E eu fiz o remédio graças a Deus, ele, até hoje, eu não sei para onde ele foi, ele tá velho.

G: Mas ele ficou melhorou com seu cuidado?

EE12: Ele ficou bom, ficou bom, graças a Deus, só com remédio, só com remédio caseiro mermo, o sumo da, do braço da palheira.

G: Da palheira?

EE12: Isso. A gente raspa né aquele negócio do braço da palheira, molha e espreme assim e dá pra pessoa beber, passa em cima, não precisa outro remédio, só com a ajuda de Deus mermo né.

G: Somente esse remédio?

EE12: Somente esse remédio.

[00.08.48]

G: E quais os tipos de cobra a senhora conhece?

EE12: Eu conheço várias delas né.

G: A senhora pode me falar o nome delas?

EE12: Posso. Surucurana é as perigosas, pois é surucurana, coral, pico de jaca, pico de jaca, a papagaia que pula na gente e é verde, a jiboia e a, como é o nome daquela cobra que subiu lá em casa sicuriju Zuca, Sucuriju.

[00.09.45]

G: Qual delas a senhora acha mais perigosa?

EF12: surucucurana.

G: A surucucurana?

EE12: É, a surucucurana.

[00.09.55]

G: E, a senhora sabe falar o nome é, das cobras em munduruku?

EE12: Não. Eu não aprendi a eu sei outros nome.

G: Mas a senhora fala munduruku?

EE12: Falo.

G: O que a senhora fala em munduruku?

EE12: A farinha o nome da farinha que eu sei em munduruku né na língua, é chitarom.

G: Chitarom.

EE12: Isso.

Acompanhante: Achimam.

EE12: Achimam é peixe, né, na língua. O, a tapioca é Siriquitá, a bom dia na nossa língua é cabiá.

G: Bom dia é cabia?

EE12: É cabia.

EE12: Boa tarde que a gente fala né é Aícate.

G: Aícate.

EE12: Aícate, boa tarde. Café é na língua da gente é caiperí.

G: A senhora falava fluentemente munduruku era?

EE12: Sim. Cachorro na língua é caretche, na língua né, da gente.

G: O seu nome como pegadora ou rezadora ou parteira tinha algum nome em munduruku, ou tem outro nome?

EE12: Não lembro.

G: Não lembra?

EE12: Não.

G: A senhora falava assim fluentemente é com as outras pessoas?

EE12: humrum.

[00.11.29]

G: A senhora realiza ou já realizou algum cuidado com alguém que foi mordido por cobra ?

EE12: Já.

G: Como foi?

EE12: Os cuidado, como é que a senhora estava falando?

G: A senhora é, já realizou algum cuidado com alguém que já foi mordido por cobra?

EE12: Humrum.

G: Como foi? Qual foi esse cuidado? A senhora usou alguma planta, alguma banha de algum animal?

EE12: Sim, pra cuidar do doente, da ferrada de cobra né que chamo, eu usei a banha do tijuaçú, com a banha do, da paca e a banha do coiso, como é meu Deus que eu esqueci agorinha, é a banha da paca, do tijuaçú e da capivara. Essas três banhas para fazer é.

G: Misturava era?

EE12: Misturado para emplastar em cima assim ó, com algodão no caso da ferrada do inseto, passando no algodão assim e botar em cima.

G: Quanto tempo deixava em cima?

EE12: Até secar né, ele seca e ele mermo caí o emplasto que a gente chama né, ele mesmo arria.

G: E quanto tempo a senhora preparava esses remédios, quanto tempo a senhora levava para preparar esse emplasto?

EE12: Quantas horas, como é que a senhora quer falar?

G: isso.

EE12: Não demorava muitas horas não. Acho que uns 15 minuto só porque a gente tem que fazer a mistura né, para fazer o emplasto.

[00.13.22]

G: Esse conhecimento, esse cuidado que a senhora faz com as pessoas a senhora herdou de alguém da família ou foi algum especialista da sua etnia que lhe ensinou ?

EE12: Não, ... com minha mãe, sempre com minha mãe.

G: A senhora acompanhava ela durante o cuidado?

EE12: Isso. Ela todo tempo durante a vida dela né, ela sentava comigo e ficava falando, minha filha esse tipo de banha, esse tipo de planta ele é bom para vários tipos de doença, e saia falando né, esse remédio aqui é bom para tal doença, esse outro aqui também, aí eu fui ficando com aquilo na minha cabeça.

G: Quando a sua mãe era viva a senhora a presenciou cuidar de alguém mordido de cobra?

EE12: Não, porque eu, no tempo que eu casei e vim me embora com meu esposo aqui do suncunduri para cá pra dentro né, e ela ficou morando em Borba aí, eu casei muito novinha.

[00.14.23]

G: E quando a senhora fez esse cuidado a senhora lembra em que momento foi, se foi logo após a picada, se foi é após o tratamento médico, ou se a senhora fez sozinha?

EE12: Eu fiz sozinha.

G: Fez sozinha, né.

EE12: Sim.

G: E, por quanto tempo a senhora fez esse cuidado? Quantas vezes assim a senhora fez?

EE12: Eu fiz duas veze.

G:Duas vezes?

EE12: Humrum.

G: Tinha intervalo de dia?

EE12: Sim.

G: Sim? Quantos dias de intervalo?

EE12: É, o intervalo que a senhora tá falando pra mim eu tô com a minha goela coçando, uma tosse péssima.

G: A senhora é, por exemplo, eram dias seguidos, ou era tipo assim, hoje daqui a três, quatro dias ou daqui a quinze dias?

EE12: Duas vezes no dia, de manhã e de tarde.

G: Duas vezes no dia, por dois dias a senhora fez?

EE12: Sim.

G: Tinha um horário específico?

EE12: É, sempre as 7 horas da noite né, que é o horário que a pessoa vai deitar, tá sossegado, o dia sempre a gente não tem as veze aquele tempo né de fazer o remédio, nem de tá em casa, as vez tá andando, tá fazendo alguma coisa.

[00:15:54]

G: E, se a senhora tivesse que é fazer esse cuidado assim hoje por exemplo ou antes, a senhora buscava o cuidado médico para fazer em conjunto ou era sempre sozinha?

EE12: Eu sempre sozinha.

G: Sempre sozinha.

EE12: É, porque eu nasci e me criei no interior, eu estudei no interior, eu terminei meu estudo na cidade de Borba.

[00.16.15]

G: Desses cuidados que a senhora fez, a senhora percebeu se teve alguma melhora?

EE12: Sim.

G: Sim. Quanto tempo depois?

EE12: Graças a Deus, melhorou, ficou bom. Cum um mês a pessoa já estava bem andando, bem trabalhando já.

[00:16:35]

G: E, esses recursos que a senhora usou, eles eram ou são encontrados com facilidade, a senhora acha rápido?

EE12: É, é com facilidade.

G: Tem assim ao redor aqui da sua casa, ou perto ou dentro da sua casa?

EE12: Tem não, aqui não, no interior tem.

G:É.

EE12: Aqui só tem capim ao redor. Lá em casa eu tenho muita planta, muita coisa mermo, tanto no meu quintal como no dela aí. Aqui não tem não, eu moro lá e venho aqui rapidinho só.

[00:17:09]

G: quanto tempo é, a pessoa ao todo teve que usar esse seu cuidado, quantos dias ao todo?

EE12: Acho que, que não teve muita demora não né.

G: Dias assim se a senhora for contar, quantos dias ou horas para cuidar dessa pessoa mordida?

EE12: Eu penso assim que uns três dias né, porque a pessoa adoece e para adoecer é rápido, agora para ficar bom, custa.

G: E, quando a senhora cuidava tinha alguma coisa que a pessoa não podia fazer quando estava mordida?

EE12: Sim.

G: O que por exemplo?

EE12: Comer comida remosa né, tá andando por aí no sol, num facilitar com a vida mesmo.

G:Quais os tipos de comida remosa que ela não podia comer?

EE12: Paca, mutum, porco de casa, é, vários tipos de comida, peixe liso, surubim, pirarara, isso aí é um veneno.

G: Quantos dias ela não podia comer?

EE12: Durante tá doente ela não podia comer, depois que sarasse aí ela podia comer, se fosse doença grave aí é uns 15 dias, aí já estava liberado, estava bom podia dizer né.

[00:18:52]

G: E entre os recursos que a senhora já presenciou ou já usou da própria cobra, é quais foram, assim quais partes da cobra a senhora já usou?

EE12: da parte da cobra só a banha e a pele.

G: A pele a senhora usou para quê?

EE12: Para defumar, para defumar macumba que é quando ajudio a gente né, a gente cisma que é aquela doença, aquela coisa né, aí a gente, se a gente tiver pode fazer a difamação. Se for.

G: Mas a senhora já chegou a fazer?

EE12: Eu cheguei a fazer sim senhora, esse meu pé aqui ó, tá aí de prova, ainda está pintando, foi judiado aí me ensinaram aí eu fiz. Graças a Deus fiquei boazinha.

G: Como a senhora fez?

EE12: A gente faz um foguinho numa vasilha de alumínio né, e coloca o carvão assim, faz um montinho, joga a pele em cima daquele fogo e bota o pé aqui ó, defumando, para o lado e para outro. Se for o que a gente pensa, vai ficar bom.

G: Quando defuma a senhora faz algum ritual, por exemplo, canta ou reza ou benze?

EE12: Não.

G: Não, só usa a fumaça.

EE12: Só uso mermo a fumaça.

G: Quanto tempo?

EE12: É, pelo uns três minuto por aí assim.

G: E quem a senhora já cuidou assim e que foi enfeitiçado a senhora também obteve melhora?

EE12: Eu fiquei boa. Só eu mermo que tive isso.

G: Que foi com a senhora mesmo né?

EE12: Isso, só comigo.

G: Com outro pessoa a senhora nunca fez?

EE12: Não, com outra pessoa não.

G: A senhora tem algum cuidado relacionado a cobra que a senhora não tenha me relatado, mas que a senhora lembrou posterior?

EE12: Humrum, não.

G: Então, dona (...).

Acompanhante: Do (...)...

G: Como é a história do (...)?

EE12: Do (...) que saiu em terra?

G: Como foi?

EE12: Tava numa festa dançando né o povo lá tu lembra o nome do ... encanta galo, encanta galo lá no madeira né, aí testava numa festona lá, estava dançando aí veio uma moça, puxou o rapaz bonito que estava sentado no banco, bonito mermo, branco, tudo vestido. Puxou ele, aí ele, falou não eu vou, vou dormir, amanhã ele tem que viajar, eu vou dormir. Aí ele deitou lá para o quarto, aí a outra mulher falou para a moça lá tu não vai chamar ele tá dormindo, vai dormir, com poucos minutos a danada foi lá chamar ele, chego lá que ela abriu a porta, olha o monstro rolo de cobra que estava do tamanho do quarto lá dentro. Ela falou tu vais chamar ele, a hora que ele levantar tu vais ver só o que vai acontecer, ela olhou o rolo da cobra lá e com poucos minutos aí, a velha, uma senhora saiu da urinar, quando ela saiu pra urinar que ela olhou estava com um palmo de terra já rachado, já, de fora a fora. Aí ela correu gritando, a mulher, fulano sai daí que tá tudo partido aqui atrás, que elas fizero alarme só foi uma, sentou tudinho onde estava. Sentou o salão contudo, sumiu. Aí era o (...0 que tinha se transformado em gente no mesmo tempo ela era o bicho né, cobra grande, e ele não queria que ninguém fosse chamar ele lá no quarto aí teirmaram, sentou tudo lá.

G: Mas, ele era uma pessoa ou ele era uma cobra?

EE12: Ele era uma pessoa.

G: Que se transformava em cobra?

EE12: Isso, ele era encantado, era não, é.

G: É encantado?

EE12: É encantado ainda ele, ele sentou lá, a sedia, só porque eles era teimoso né, que ele falou que não era pra chamar ele, e foi. Aí ele se apoquentou, humhum, a comunidade toda foi para fundo.

G: Essa comunidade não existe mais?

EE12: Não, não existe mais não, ela já está, é no fundo já.

G: E ela fica onde?

EE12: Lá no, eu esqueci o nome lá do, onde que sentou.

Acompanhante: Encanta galo mermo, na área de Borba.

EE12: É mais, ele tinha, tem outro nome, tinha outro nome não sei se mudou, ainda andei por lá ainda, ixi era uma comunidade bonita, bonita mermo, ele dançava na festa ninguém dizia que ele era um inseto um animal como é que chamo, uma serpente né.

Acompanhante: É por que ele era encantado, era não, é.

G: Ele era uma criança que foi encantada?

EE12: Ele era uma criança ele foi encantado.

Acompanhante: ... desapareceu.

EE12: Foi, sumiu, hoje ele é uma serpente grande. Mas foi real mermo.

G: Muito obrigada tá dona (...).

EE12: De nada, tá. Desculpa aí ...

**Entrevista 13**

**Caracterização do Entrevistado**

**Idade:** 42 anos

**Especialidade:** Pegadora de desmentidura e Curadora

**Informações Relevantes:**

**Informações Relevantes:**

**a)**  A entrevistada relatou que se considera pegadora de desmentidura e que possui certificação de curadora pela Fiocruz da Amazônia.

**b)** Em mordidas de cobras é aconselhável o resguardo semelhante a mulheres no período puerperal. Ficando restritas visitas ao doente, alimentos considerados remosos e relações sexuais.

**c)** Ela informou após o encerramento da entrevista que em ocorrências de mordidas de cobras ficam restritos alguns alimentos como peixe liso, matrixã, carne de paca e porco de casa.

**d)** Após o término da gravação a especialista também relatou que desde os 10 anos de idade ela é agoniada por espíritos e que desde então puxa desmentidura.

**Transcrição da entrevista**

[00:00:01]

G: Me conte se existem histórias, lendas, mitos ou contos com cobras em seu povo

EE13: Já? Aí a mamãe, a mamãe falava sempre para gente, quando ela começava contar história… ela disse que eles estavam numa festa né, lá no Canta galo, aí tinha aquele rapaz bonito, que se vestia bem mesmo, bem mesmo, bem bonito, bem branquinho, aí disque teve uma menina, que ela gostou dele né. Aí o pai dela dizia, cadê fulano que eu não me lembro o nome da moça… ai cadê o fulano, cadê o fulano… aí disque estavam lá no quarto né. Aí disque a mulher lá, a mãe dela sabia, só quele ela dizia não vai brechar, porque não pode brechar né. Aí passou, quando foi meia noite, disque a curiosa foi lá e brechou, quando ela brechou aí só virou aquele monte de cobra lá dentro do quarto. Ai que foi pra fundiar lá a comunidade né, da onde foi que a cobra estava, afundou todinho, e o que estava pra cá se salvou né, o que não estava foi tudo profundo.

G: No caso, a pessoa que estava com essa moça era uma cobra?

EE13: Era, uma cobra.

G: Só estava no corpo de uma pessoa?

EE13: No corpo de um rapaz.

G: E foi um lugar, que esse lugar também afundou foi? Como foi?

EE13: É, afundou lá, foi lá no Cantagalo, só ficou o remansão assim, eu ainda não fui lá, mas no sonho já fui (gargalhada).

G: No sonho já? A senhora foi no sonho porque a senhora tem alguma especialidade relacionado a esse local?

EE13: Não! Só fez passar por lá só.

G: É? Mas a senhora lembra como foi, quando a senhora em sonho?

EE13: Não... já faz tempo já, quando eu estava com vinte e dois anos, quando eu estava em Manaus ainda.

G: E o que a senhora foi fazer lá nesse lugar, quando a senhora passou por lá?

EE13: Foi só porque estavam me agoniando, estavam querendo me levar…

G: Lhe agoniando, a senhora fala assim…

EE13: Assim… porque eu ainda não tava, ainda assumindo ainda, assim o papel que eu tô fazendo agora né. Eu ainda estava ainda, eu estava afastada ainda. Aí por isso que eles foram lá, esses dito aí. De lá ele foi lá em Manaus, pra querer me levar. Ele mais outro rapaz, aí quem me livrou foi a mamãe e a tia (...).

G: Entendi… então, assim, antes de a senhora ser pajé, a senhora era perturba?

EE13: Sim, desde quando eu nasci.

G: Desde que a senhora nasceu?

EE13: Até com vinte e dois anos… não, é.. vinte e dois anos.

G: É? E em que momento a senhora passou a atuar?

EE13: Foi quando eu fui lá em Manaus, lá no batuque lá, tinha um homem, tem um homem chamado seu (...) né, aí o (...) me levou lá, aí ele falou pra mim, que eu tinha que assumir…. Ou puxar, ou benzer, ou fazer para lado do mau… ai ele falou que eu tinha que escolher um desses três né, para mim assumir. Aí quando interesse vinte e dois anos, era para mim assumir, porque se eu não assumisse eles vinham me buscar, porque… eles foram ao vivo mesmo para quererem me pegar, ao vivo mesmo.

G: E de lá para cá, a senhora atua quanto tempo?

EE13: Desde os vinte e dois anos. Eu tô com quarenta e dois.

G: A senhora então, tem assim, um guia ou um mestre?

EE13: O meu só é um. Linha vermelha.

G: É uma linha vermelha? Qual o nome do seu mestre?

EE13: (pausa, com risos…) eu não… eu só chama ela de loira.

G: De loira?

EE13: Hanhan… mas ela é lá da boca do… dali do Paranázinho. Lá eu não posso passar quando eu tô menstruada, tenho que passar lá por fora. Não posso passar por… ela mora nem na boca lá.

G: E se a senhora passasse assim menstruada, acontecia alguma coisa?

EE13: Eu passo uma vez né, quando eu vim de Borba, aí eu passei o tempo ruim. Fiquei ruim, aí ela veio no meu sonho, falou que não era mais pra mim passar lá mais, quando eu tivesse menstruada, porque senão eles ia me agoniar muito mesmo, até ficar doente né. Ai por isso que não gosto de passar por lá, passo la fora…

G: A senhora tem algum guia espiritual é… na forma de cobra?

EE13: Não!

G: Não? Nunca a senhora recebeu nenhum guia como cobra?

EE13: Não!

[00:04:41]]

G: Não? A senhora já teve experiência com cobra na sua aldeia?

EE13: Só pra fazer algum tratamento?

G: Isso!

EE13: Já!

G: Já? Como foi?

EE13: Para fazer tratamento, eu já cuidei já, é contra rasgadura, que eu uso também a banha da cobra né, e contra também o feitiço, que não é minha área isso daí, mas assim, com pena das pessoas já cuidei, mas não é minha praia. Já cuidei já com a coisa dela, como é? com a banha e com a casca... casca não, como é (pensando)… a pele dela. Já fez defumação também pra… contra o feitiço né, contra o mau olhado também que é bom.

G: Como é que a senhora fazia essa defumação, assim, o preparo? A senhora pode me falar?

EE13: O preparo a gente pega três pedra de carvão, aí pega três é… pedacinho de… pode ser aquela fatiazinha de qualquer pau, aí pega três fatia de, da ivarataia, aí coloca, coloca um pouco de farinha, aí um pouco de café também, aí coloca doze espinho de tucumã, aí pode fazer a defumação… de cima pra baixo, faz em cruz.

G: Mas tem alguma parte específica do corpo da pessoa?

EE13: Tem… pode fazer só que a pessoa teve na rede, aí tem que fazer só de baixo da rede, ou na porta também. Não pode passar ninguém perto também. Eu já fiz também isso aí.

G: E com a banha, quais foram os tratamentos que a senhora fez?

EE13: Com a banha a gente usa, para passar em cima, e para tomar também, é três pingos de banha….

G: Mas é em cima da mordida de cobra a senhora fala?

EE13: Não, da mordida de cobra, eu da não usei não. Mas eu que é o remédio que… pode pegar doze folha de pião roxo, aí bate, aí dá o sumo pra pessoa tomar, que é contra mordida de cobra, e também a água doce e eu já usei agora que me alembrei, já fazia… da idade do meu irmãos, desse do (...). Quantos anos o (...) tem mamãe? (perguntando da sua mãe, que estava presente durante a entrevista) 27 por aí assim né. Da vez a cobra mordeu o menino lá onde a gente morava, lá no interior. Aí ele chegou mordido de cobra né, e eu era nesse tempo… eu estava com uns dez anos, por aí assim eu estava…aí eu vi aquilo, o menino chegando agoniado assim, foi bem aqui na perna dele. Eu peguei, falei, vou já fazer um remédio para esse menino, eu fui lá, peguei fez água de açúcar, aí foi na caixa de fósforo né, tirei aquela cabecinha da caixa de fósforo, coloquei na água e dei para o menino, aí peguei o resto, raspei, aí coloquei em cima da cesura.

G: Onde foi mordido?

EE13: A senhora sabe que foi mesmo que jogar água em cima do fogo? Não careceu nem o menino vir pra Nova Olinda.

G: Não veio pra Nova Olinda?

EE13: Não. Esse daí foi o remédio que eu fiz pra ele. Quando eu era pequena ainda, que eu ainda não era nada (sorrisão)…

G: Mas a senhora já morava aqui no Macambira?

EE13: Não, morava lá no Suncuduri.

[00:08:30]

G: No Sucunduri né? E as cobras possuem algum significado particular pra senhora?

EE13: Para mim assim, eu já passei várias vezes por cima de cobra, e elas tem medo de mim (expressando sorriso) é que eu já passei por cima daquelas cobras azuis, uma vez eu passei por cima e ela foi embora, não me atacou. Outra vez passo por cima da cobra surucucu também, do mesmo jeito, ela tem medo de mim, não sei por quê.

G: Mas ele tem valor, importância pra senhora?

EE13: Tenho é medo dela (expressão de sorriso).

G: Ela tem medo da senhora, e senhora dela?

EE13: Ela é bom, só para fazer remédio, mas só a cobra que eu uso é só a sucuriju, a… essa surucucu né, e a… aquela jiboia, esses três aí que é bom para remédio.

G: E das três a senhora usa o que delas?

EE13: Das três… a jiboia pra atrair (expressão de gargalhada)

G: A jiboia serve para quê?

EE13: Da jiboia é, a gente usa só também a pele dela né, para fazer defumação, e a cabeça que assim, sempre o pessoal me procuraram… para atrair pessoa, como é o nome… a banha serve também, mas eu ainda não usei não, eu só…

G: A senhora só tem conhecimento?

EE13: Humhum… é. O (...) também usava é… aquela coisa da cobra na vista dele, disque era para atrair mulher (sorrindo).

G: E as outras cobras servem para que? Assim, o que a senhora tira delas?

EE13: Assim, da sucuriju como eu tava falando para senhora, da sucuriju eu uso a banha dela, para fazer o tratamento de pneumonia que eu uso. Para tratamento de, dessa rasgadura que chamam né, aí… para todo tratamento eu uso. Para passar em ferimento. Para cirurgia, que eu já usei em mim mesma né, para cirurgia e é bom para tudo, a banha do sucuriju.

G: Mas a senhora tem algum preparo que a senhora faz com ela?

EE13: É… eu coloco no xarope, ou é… para tomar né, pra pneumonia. Ou no meu de abelha, ou no… para usar pra batida… mão fica batida né, eu uso para, eu uso no, eu bato no… eu posso falar isso daí também? que eu coloco no pirarucu cá que eu bato né, aí eu bato a erva do passarinho, aí eu coloco três bago de pimenta do reino, e o amocrescido que eu bato. Aí eu faço emplastro, eu eu grudo em cima do ferimento né, com a banha do sucuriju. Que isso daí é muito bom. Que eu já fez né, no Hélio, eu já fez em várias pessoas também isso.

G: senhora faz como emplastro é?

EE13: É…

G: Mas tem um tempo de preparo?

EE13: Tem, mais não é muito demorado não.

[00:12:11]

G: E as cobras possuem alguma utilidade no seu dia a dia?

EE13: Só para mim tomar a banha dela?

G: Para tomar?

EE13: É…

G: É? Oral sem nada ou com alguma coisa?

EE13: Eu que já tomei para minha cirurgia, eu já tomei durante um mês, direto no café, para sarar rapidinho…

G: É… e é fácil encontrar por aqui?

EE13: Não. É difícil.

G: Mas a senhora sempre tem em casa, assim na aldeia?

EE13: Só eu que tenho (sorrindo)

G: A senhora tem no caso né? Ta…

EE13: Não sei… a (...) tem também.

[00:12:47]

G: Quais os tipos de cobra que a senhora conhece?

ER13: Conheço só a jararaca, surucucu, a sicuriju, a jiboia, e a papagaio, que é amarela né. Só essas daí, só as cinco. Ahh… e a coral mesmo, que é flamenguista. Esses seis tipos de cobra eu conheço.

[00:13:13]

G: E entre elas, qual a senhora considera mais perigosa?

EE13: Mas perigosa é a surucucu, a surucucu e a papagaia, essa daí são a mais…

G: A surucucu e a papagaio é?

EE13: Surucucu pico de jaca que chamam.

[00:13:39]

G: As cobras elas possuem é… algum nome específico na linguagem Munduruku? Ou como elas são chamadas na sua língua diária? A senhora sabe chamar cobra em Munduruku?

EE13: Já ouvi falar já… (sussurrando)

G: Como?

EE13: Poibu (sorrindo)

G: Poibu né?

EE13: Humhum…

G: Mas esse nome ele é só pra um tipo de cobra, ou pra todos os tipos de cobra? Ou Cada cobra tem um nome?

EE13: Todos os tipos de cobra, isso daí…

G: Então, pra ele se refere todos os tipos no caso?

EE13: Aham

[00:14:30]

G: A senhora realiza algum cuidado pra pessoas que foram envenenadas por serpentes, por cobras?

EE13: É… que quando a pessoa é picada de cobra né, ela não pode assim, pessoa olhar né, quando tá picada, porque, é, faz mal. Quando a pessoa tá picada de cobra, aí ele tem que ter o maior resguardo, igualmente quando a mulher está de resguarde de bebê, para não comer comida remosa, para as pessoas não olharem, que faz mal. É…para um monte de coisa. Nem o homem se triscar com a mulher, que isso é um grande perigo.

G: Mas se as pessoas olharem, o que acontece?

EE13: O que acontece… é porque inflama de novo, sai sangue pesa cisura.

G: Aí então, nesse período assim. Ela também fica distante do esposo é?

EE13: Uhum… é!

G: É… Ela no caso, ela mantém as atividades…

EE13: Durante é três meses, não pode bater… é… não pode manter relação não com o marido.

G: Entendi!

EE13: Quando a pessoa tá picada. É o mesmo resguardo da mulher quando tem a criança, é o da cobra.

G: Entendi. Então, se a senhora fosse falar assim, a pessoa não pode fazer isso, quando está picado de cobra, se tivesse que fazer uma lista assim, uma relação. O que a senhora me diria assim, o que eu não posso fazer? Se eu fosse mordido de cobra e senhora fosse me cuidar, o que eu não posso fazer, que a senhora falaria para mim?

EE13: O que eu ia falar pra senhora, a senhora não fazer movimento, não sair no público né, pra ninguém ver a cisura, e a senhora é… saiu até da minha mente agora… comer comida remosa, várias outras coisas né. Não podia ficar com a perna abaixada também, porque incha né.

G: E o que é considerado comida remosa?

EE13: Comida remosa é peixe liso, é matrinxã, é carne de paca, é de anta, porco de casa. É…

G: Porco de casa é?

EE13: É…

[00:00:17]

G: E você pode me relatar quais cuidados você realiza, como realiza, e quanto tempo de preparo os cuidados para curar as pessoas? A senhora pode me falar o que a senhora faz, assim, para tratar alguém?

EE13: Pode ser esse daqui ou do outro?

G: Pode. O que a senhora faz assim, como pajé, pra curar as pessoas?

EE13: No geral é? Eu assim, já cuidei da pessoa quando tão com pneumonia né, já fui muito procurada aqui em Nova Olinda mesmo, até lá no hospital já foi, e pra… contra tuberculose também né, já cuidei.

G: A senhora cura várias coisas né?

EE13: É!

G: Mas assim de cobra, a senhora…

EE13: Eu já, eu já… já cuidei também só um, agora que veio na minha também. Dum que tava aqui no hospital, já estava com uns três dias que a cobra tinha mordido ele lá no Sucunduri. Aí ele veio pra cá, ai a… ele é marido da minha prima né, aí o filho dela veio em casa, me procurou… tia o papai tá mordida de cobra, ele falou, lá no hospital. A senhora vai lá dar uma olhada lá… aí eu foi lá né, aí quando cheguei lá, a perna dele tava dessa grossurona assim oh. Coisa bem bem inchada. Aí eu, ah, vou já em casa já…. Ai eu voltei em casa, peguei a banha do jacuraru né, eu peguei a banha da paca que eu também não tinha, não tinha o féu, aí eu tinha a banha. Aí eu peguei lá, passei lá na perna dele, a banha do jacuraru e a banha da paca… a s embora sabe, quando foi de tarde, já tava tudo desinchado, ele já tava até andando, que ele não andava. Eu acredito que isso foi bom pra ele.

G: E quando ele foi pra casa, a senhora ainda cuidou dele? Continuou cuidando?

EE13: Não. Ele passou uns três aqui, aí depois que ele andou, ele foi embora né, que ele mora lá para cima do Sucunduri. Aí ele voltou para lá. Só o remédio que eu fiz, foi só isso para ele, mas ele falou que desde que passei essa banha, amodo assim, que acalmou tudo, parou a dor, parou tudo que ele estava sentindo, até os inchaços desceu.

G: E quando a senhora foi assim, la no hospital né, de Nova Olinda, eles é… aceitaram também a senhora tratar dele?

EE13: Eles aceitaram.

[00:19:44]

G: Aceitaram? E a senhora costuma relacionar o seu cuidado com o cuidado médico? Ou a senhora sempre faz o cuidado só? Como é que é essa questão?

EE13: Não porque, quando me chamam para ir lá no hospital né, aí a família que me leva lá, aí eu falo, olha, cuidado para eles não me ralharem (falas sorrindo). Aí não, eles não vão lhe ralhar não, que eu já falei com o pessoal aí, disseram a que senhora pode puxar.

G: Então, a senhora já foi lá algumas vezes?

EE13: Já… ixi… já fui várias vezes, até lá no parto já entrei pra ajudar menina.

G: Mas eles pedem autorização antes é?

EE13: Eles falam lá com eles primeiro né, aí eles liberam, aí que eu entro lá.

G: A senhora também é parteira é?

EE13: Não.

G: Não?

ES13: Eu só ajudei, mas eu não fez isso não. Só ajudei uns três partos só.

G: Só auxiliou?

EE13: Humhum

G: Mas a senhora é mesmo pajé né? A senhora fez algum preparo para ser pajé?

EE13: Só o certificado

G: O certificado?

EE13: Não porque assim, desde quando…, antes disso, eu já fazia trabalho né.

G: A senhora já fazia trabalho, na aldeia aqui?

EE13: Ai quando veio pra mim fazer esse curso, pra mim participar. Aí uns três mês antes, eu já foi já vendo, o que eu vou fazer, o que eu vou levar né. Aí já foi juntando minhas folhas, minhas coisas, pau, fez garrafada, fez xarope… ai tudo pra mim levar, ai fiquei pensando, será que… porque sempre que a gente vamos fazer alguma curso, a gente tem que levar alguma coisa para mostrar né. Aí falei, será que vai ser assim… quando foi na hora, chegou lá ninguém tinha levado casaca, fora a arrumar tudo só na hora lá, lá na comunidade mesmo. Aí eu falei pra (...), e aí minha cascas? Não… tem que trazer para cá. Foi levando… aí quando foi na hora eu teve que apresentar casca por casca, folha por folha, falar como era pra mim fazer os…

G: É… e esse curso que a senhora fez, que foi pra pajé né, ele foi feito aonde?

EE13: Lá no laranjal.

G: Mas foi um encontro é?

EE13: Uma assembleia…

G: É… e tinha pessoas de todas as aldeias?

EE13: Sim. Todas as aldeias e as pessoas lá de Manaus também. Dos dois rios né…

G: Mas vocês trocaram é… informações e conhecimentos foi?

EE13: Troca e saber. É troca é saber né (tirando uma dúvida com a pessoa que estava lhe acompanhando).

G: E faz quanto tempo?

EE13: Foi em setembro, dia dez de setembro.

G: Ah, foi recente então? Mas antes disso a senhora já atuava?

EE13: Já… Desde quando eu nasci, que eu já fez um remédio para a mamãe.

G: E as pessoas buscam a senhora é, para cuidar?

EE13: Desde quando eu nasci… eu me lembro benzinho, eu estava com oito anos, eu fez um remédio pra mamãe, que ela estava com um feitiço bem aqui na barriga dela.

[00:22:51]

G: E esse cuidado a senhora herdou de algum familiar ou de algum especialista da sua etnia?

EE13: Não… Foi da mamãe

G: Herdou da sua mãe? A sua mãe também ela tem essa especialidade?

EE13: Tem… a mamãe puxa a barriga, benze, reza para espinha… é… fez parto também, um monte de coisa a mamãe tem, ela e o papai.

G: Todos os dois?

EE13: É…

[00:23:21]

G: Você poderia me relatar é… em qual momento a senhora faz esse cuidado, se… quando a pessoa é mordida, se é logo depois da mordida, se é quando ela retorna do hospital ou se é em conjunto com o tratamento hospitalar?

EE13: Tem que ser na hora. Na hora que a pessoa é picado, a gente que dar logo um remédio para cortar o efeito. Amarrar no toco da perna, com um pano… tem que ser na hora, porque depois, é arriscado ir à óbito né.

G: Esse amarrar serve pra que?

EE13: Para dor não alterar aqui para cima.

[00:24:07]

G: A senhora realiza esse cuidado sozinha, ou associado ao cuidado médico?

EE13: Quando o médico não tá, é sozinha né.

[00:24:19]

G: Dos cuidados que a senhora já fez, teve melhora da picada de cobra?

EE13: Sim. Desse que estava dizendo, que eu fiz lá no Sucunduri. Que era só a gente mesmo e Deus que estava…

G: Não precisou levar para o hospital?

EE13: Não.

G: Foi só o tratamento em domicílio né?

EE13: Só…

G: Quanto tempo a senhora realizou esse cuidado, que foi lá no Sucunduri? Quantos dias?

EE13: Não. Careceu só mesmo, só no mesmo dia, ele… depois que foi passado o remédio, ele não sentiu mais nada. Não inchou, não fez nada. Foi só mesmo esse remédio que eu dei para ele. Não careceu… sempre eu ficava olhando… não inchou, não… ele não sentiu nada, nada.

G: Mas a senhora fez assim, algum… puxou, ou rezou, ou fez defumação com essa pessoa?

EE13: Fez só a defumação?

G: É? No mesmo dia?

EE13: Foi!

G: E como foi assim, que a senhora fez essa defumação? Queria que a senhora me falasse assim, como foi preparado, como ele estava, como a senhora…

EE13: Quando ele chegou né, aí a gente ficamos desesperado, como a gente tava só a gente lá no interior, só a mamãe… a (...) nesse tempo ainda era criança. Aí o menino trouxe, veio só o rapaz com ele, aí quando foi nesse hora a mamãe ficou agoniada, o que a gente vai fazer então… mamãe eu vou fazer um remédio doido, todo tempo era assim né, fazer um remédio doído. Aí cheguei, aí eu fez logo a água de açúcar, peguei esse coisa dei pra ele, a raspagem lá, aí peguei o resto, aí eu fez a defumação como o espinho de jauarí né, aí coloquei… coloquei o carvão, aí o café que é seu meu coisa de usar, o café e a farinha, e as três coisas daquela coisa de mandioca né, daquela que a gente torra a farinha…. Aquela que a gente torra a farinha, e fica só aqueles pauzinho… da crueira né. Eu coloquei lá, aí coloquei a perna dele assim, que nem a senhora tá no banco, fez a defumação embaixo, pra subir aquela…

G: Só embaixo de onde foi mordido?

EE13: Foi…Aí foi só isso que eu fez.

G: Quanto tempo durou essa defumação?

EE13: Acho que só uns dez minutos só.

G: Fazia alguma oração durante a defumação?

EE13: Não.

G: Somente a defumação?

EE13: Só… oração não é (sorrindo) não entendo não.

[00:00:26)

G: Os recursos que a senhora utilizou, ele eram encontrados com facilidade?

EE13: Era. Tudo a gente tem em casa.

G: Tudo tem em casa? Tudo guardado?

EE13: É… a gente tem em casa, farinha, café. Só a coisa da mandioca que é difícil, só quando torra né.

G: E aquele recurso que a senhora usou da cobra também tinha em casa?

EE13: (silenciosa)

[00:27:09]

G: Para senhora e encontrar esses recursos, a senhora levou quanto tempo mais ou menos? Assim para fazer todo esse preparo… em quanto tempo a senhora fez?

EE13: Uma hora.

G: Uma hora?

EE13: Para mim fazer, esse que eu tô falando né. Fazer xarope é mais.

[00:27:45]

G: Entre os recursos que a senhora… entre os recursos, a senhora já presenciou ou já utilizou algo da própria serpente para realização do cuidado? A senhora já usou, alguma parte da cobra? Vou dar um exemplo para senhora, a pele, a banha, a carne da cobra ou o osso, para tratar a própria mordida da cobra?

EE13: Hum… só a banha.

G: Só a banha? Como é que é a senhora utilizava?

EE13: A banha a gente toma a banha.

G: Só tomado?

EE13: Sim

G: Passava em cima?

EE13: A gente usa para passar em cima ou para tomar, para fazer compressa também. A pomada também…

G: Como é feito a compressa e a pomada?

EE13: A compressa a gente mistura com mel, com meu já… com andiroba. Aí com copaíba também, tem que ser só um pouquinho né, da copaíba, dois pingo só, não pode colocar muito. Aí coloca o… andiroba, copaíba, aí a banha também que usa. Ai amorna, aí a banha do jabuti também. Amorna e passa em cima. E eu já usei também para… quando os meninos estavam com os braços dele quebrado né, que eu passava assim, para emendar. Aí faz a compressa, bom… tem que fazer só a boca da noite isso. Amorna, aí faz assim. Aí vai puxando. Assim que é a compressa, que fala. Entendeu?

G: Entendi…

EE13: Só isso?

G: A senhora lembra assim de algum relato que a senhora não tenha falado, durante essas perguntas que a senhora queria falar assim, relacionado à como cuidar de alguém que mordido com cobra?

EE13: Não.

G: Não?

EE13: Não me lembro não.

G: Muito obrigado dona (...).

**Entrevista 14**

**Caracterização do Entrevistado**

**Idade:** 74 anos

**Especialidade:** Parteira, pegadora de desmentidura e benzedora

**Informações Relevantes:**

**Informações Relevantes:**

**a)** Para o tratamento em mordidas de cobra a especialista mencionou ter conhecimento do uso da carne da cobra para utilizar como emplasto.

**b)** A especialista utilizou para mordida de cobras, o chá do paricá (cipó), sumo do cupuaçu, misturado com a espetada de São Jorge para aplicar como emplasto. Ela também mencionou tratamento com o cipó taracuá, a banha do boto e a copaíba.

**c)** Como restrições alimentares foram mencionados carne, frango, galinha caipira e jaraqui da escama fina. E como medidas de resguardo estavam inclusos não pisar do carvão e não ter contato com outras pessoas.

**d)** Em uma experiência pessoal vivenciada de mordida de cobras, a entrevistada já realizou benzimento no doente com o intuito de retirar o veneno da cobra.

**e)** Em sua entrevista a especialista demonstrou conhecer vários tratamentos com o recurso da cobra, já tendo utilizado a banha, a carne e o couro para defumação.

**Transcrição da entrevista**

[00:00:01]

G: Me conte se existem histórias, lendas, mitos ou contos com cobras em seu povo.

EE14: Olha… a cobra, essa que mordeu o, que mordeu… eu já cuidei de muitas pessoas, eu e meu pai quando era vivo, porque quem trabalhava era o meu pai. Mas aí como eu disse né, eu assisti minha filha, o médico disse assim, a senhora não pode assistir porque pode acontecer alguma coisa. Eu disse não, porque vocês apreenderam aquilo de lápis, eu aprendi que Deus deu o meu dom né. Eu assisti minha filha direitinho. Ai porque assim, a mordida de cobra, ela tem dois opção né. Aliás três. Porque tem a surucucu, a arana e a mais perigosa é a surucucu pico de jaca, quando ela não mata, mas ela aleija né, e a pessoa fica sintomas. Meu filho foi picado, passei natal e ano aí no, passaram aí…?? Foi a surucurana,mas como ele não fui muito demorado, porque o remédio que a gente ensina pros filhos né, eles já ficam naquilo né. Aí a cobra morreu, ele matou a cobra. Pendurou, não inchou, né. Inchou, só ficou um pouco roxo, mas graças a Deus, ??? (Fala rápida, sem possibilidade de entendimento) mas a gente tem o remédio né. Que se chama o cipo taracuá,

G: Cipó taracuá?

EE14: O cipo taracuá… quando a cobra pica a gente, a gente pendura ela, e corta ela. Mata ela, do lado do pescoço, risca ela assim né, aí pendura ela e amarra onde ela pica a gente. Aí o veneno desce todinho.

G: E amarra com que?

EE14: Com o cipo taracuá. Para veneno e o sangue não subir, né… não dá hemorragia, essas coisas na gente né, porque sempre dá hemorragia né. Aí a gente deixa aquele sangue, deixa ela pendurada, aí quando aquele sangue secar, vai aliviando a dor, ele não sentiu muita dor, sentiu dor quando ele veio para cá, aí ele tomou injeção, ele passou o que, oito dias só aí. Tratei ele em casa,

G: Ele foi para o hospital e a senhora tratou ele em casa também?

EE14: É… tratei ele em Casa, com remédio caseiro né. Porque eu dei para ele a banha do boto, a banha do boto né, com a copaíba é muito bom.

G: Como a senhora fez esse preparo?

EE14: Eu? Botei na vasilha, o chá do paricá, o paricá é próprio para mordida de cobra mesmo. Não tem essas espadas de São Jorge, que tem muito na frente das casas? Aquilo é um santo remédio. Tira o sumo dele, do cupu né, desses cupus mesmo, mas nós chama para ele de cupuaçu também, cupui né, tira, bate bem e mistura com a espetada de São Jorge e o paricá e emplastra em cima, amarra, aí ele chupa o veneno todinho. Dar o chá para ele tomar, do paricá.

G: E quantas vezes a senhora faz isso?

EE14: A gente faz o emplastro né, é três vezes ao dia né. Bota a parte da manhã, quando começa secar, bota de novo até a tarde né, aí a noite a gente já não bota, a gente coloca outro tipo de remédio né, em cima né.

G: Que outro tipo de remédio?

EE14: Pode ser a copaíba né, com a banha do boto né, e a pinha da anta, tipo assim uma mistura ... que a gente faz tipo uma pomada, e a banha de jabuti e a tartaruga. A gente faz aquilo e passa, o cebo de olanda, só o redor onde está. Ai ele não incha, não pega frio, não pega vermelha, que chama inciva né… porque aquilo se a pessoa facilitar né, aquilo que apodrece o pé da gente, aí vira aquilo que chama aquele abscesso que é o tumor né, ele pode furar pra lá, comer comida remosa… agora a cobra tem esse mistério também, a gente da cidade, agora no interior tem um mistério, todos os olhos, porque lá a gente cumpre assim né. Quando chega uma pessoa, a gente não deixa a pessoa ver o mordido de cobra né, nem mulher grávida, porque nós tivemos muito exemplo de pessoas que foi picado de cobra, e a pessoa morrer, por aquelas pessoas tem o olho venenoso assim, porque se diz assim, nós do nos olhos, é bom, mas a gente não sabe, porque tem pessoas que olha a gente né, que chama olho mau, olhos grandes né, é o que acontece. Aquela criança se uma pessoa olhar, dar quebranto né? Dizem ah… o fulano deu quebranto. Quebranto é quando pega aquele mal olhado, que a criança provoca, dar diarreia, é assim… já tratai muito de criança, olha minha filha, o médico não cura não, o que trata é remédio caseiro, e ela tem a maior em remédio caseiro. E aí a gente conserva aquilo né, porque só dão o soro tetânico né, que um tipo de remédio.

G: E a senhora rezava, benzia?

EE14: A gente benze, para tirar força do veneno, e controlado né, comida também tem que ser suspensa, é mesmo que ser uma dieta, durante quarenta dias.

G: Como é essa dieta?

EE14: A dieta para não comer comida remosa né. Outras não olhar, não olhar a pessoa, manter a pessoa escondido, e não pisar em carvão, por causa da micose né, infecção, a senhora sabe como é né. É por isso, que eu digo, meu filho passou um mês e quinze dias assim.

G: Que tipo de alimento a senhora considera remorso?

EE14: Remoso né, por exemplo a carne, frango, a galinha do quintal faz mal. Ele só comia peixe. Jaraqui… nem jaraqui dava para ele, da escama grossa, da escama fina faz mal né. No interior tem muita comida boa, pescada, acará, cutia, essas comidas assim leves né, a gente comia. O pai dele comprava carne aqui, era comida, a dieta dele assim, e o remédio que ele tomava. Aí ele tomava, que o médico passou, aí acabou né. Eu disse, bom isso já é comigo… tratei dele, está aí, muitas pessoas antigamente nem tinha, nem precisava de médico não. Médico era gente no interior, com a ajuda de Deus. Teve gente que mordeu, oito horas da manhã, pra ser encontrado uma hora da tarde, botando sangue, botou sangue até pelos olhos, pelo canto do olho, mordido de cobra, mas nós escapemos, tá aí ele. Quando inteirou quinze dias, a cobra mordeu ele de novo, e tá vivo.

G: A senhora falou que a senhora fez reza nele né?

EE14: É reza nele. Eu que tratei do (...), que chamam sabá, eu que tratei dele.

G: A senhora poderia fazer essa reza para mim, para mim saber como é?

EE14: A oração eu não posso fazer.

G: Não pode né?

EE14: Não… não posso.

G: Tá bom… e a senhora fazia com é… fazia alguma defumação?

EE14: Fazia…

G: É? Como que fazia?

EE14: A defumação faz com pelo de… cuandu macaco prego

G: Pelo de cuandu?

EE14: É…é um porquinho assim, a senhora não conhece né… ele é cheio de espinho, a gente paga aquilo, se tiver com mal olhado na casa da gente, a defumação para dor de cabeça oh… aí bota para defumar…

G: Só na área afetada?

EE14: É só na área que tá… é o pé… pode defumar com essas espadas de São Jorge, essas espadas… não tem essas de enfeite que chama, pois é, a gente planta para remédio né. As pessoas plantam para enfeite lá, a gente planta para remédio. Para mordida de cobra, é muito bom.

[00:07:25]

G: A senhora já teve alguma experiência com cobra aqui na sua aldeia?

EE14: E já…

G: Como é que foi essa experiência?

EE14: A experiência de morder?

G: É…

EE14: Pois é… mordeu…

G: A senhora já foi mordida?

EE14: Já, eu já fui mordida. Meu filho com cinquenta anos, ele nunca foi picado de cobra, porque ele nunca… podia não acreditar… porque a gente usa remédio né… eu faço remédio pra eles usarem, o único que fez, teve que viajar… eu fazia remédio né, para bicho nunca picar eles, eles dormiram junto com surucucu pico de jaca, eles nunca mordeu né. Agora, ainda trouxeram a cobra, assim a cabeça… a senhora sabias que a cobra, ela não carrega o veneno nos tocos dos dentes dela assim. O veneno dela assim… quando ela vai beber água, ela provoca aquilo, ela tem uma bolsa, é uma bolsa que ela tem… se arrebentar aquela bolsa… quando ela… para de beber água, ela coloca, ela bota, ela torna botar para dentro. Aquela bolsa que ela vai espocar pra, quando ela pica as pessoas, estoura nos tocos dos dentes, quando cria o veneno, é… no corpo dela ela não tem veneno, aí o pessoal costuma matar a cobra surucucu e engolir o rabo né, aí nunca mais a cobra morde ninguém, atora o rabo, mas ele não teve coragem de engolir o rabo…

G: O seu filho?

EE14: É… olha já morreu várias pessoas lá, de antigamente já morreu. Morreu um rapaz lá, com mordida da cobra desse tamaninho surucucu, por causa de dez urisso de castanha, a cobra matou quatro, três cachorro e ele morreu. Foi chutar, a cobra pulou… porque o que mata as pessoas é o susto, que tem gente que tem muito medo né, ele morreu por causa do susto. Ele chutou a cobra, a cobra mordeu ele… ele morreu meia noite no meio do mato, com uma distância como daqui ao Fontinele, carregaram ele de noite, quando chegaram lá, ele já tinha morrido, porque não tinha como fazer remédio, e quando o rapaz veio avisar a gente, já era muito tarde. Quando chegaram e ele já estava mordo, já tinha morrido meia noite. Morreu ele e morreu os cachorros também. Os remédios que fizemos pra levar, não deu para ele e nem pros cachorros, porque morreram tudinho. O mesmo horário que ele morreu, os cachorros morreram também. A surucucu pico de jaca é mais perigosa que a surucucurana, dizem que quando ela não mata, ela aleija.

[00:09:53]

G: E as serpentes possuem algum significado para senhora?

EE14: De que? De serpentes?

G: É… elas têm algum valor, alguma importância pra senhora?

EE14: Pra mim eu acho que não. Aliás para mim, só a jiboia né. Porque a jiboia tanto serve para fazer remédio… e a banha da surucucu também, ela é muito bom para reumatismo, e a banha da jiboia, é muito bom pra… ela que é o remédio da surucucu quando morde, a banha da jiboia pode bater com o chá da paricá e tomar né, na hora que ela morder.

G: Então, ela tem utilidade para senhora?

EE14: Tem…

G: Tem? Serve para vários tipos de remédios?

EE14: Vários tipos de remédios… a surucucu e a jiboia né. Porque a jiboia sede é como a história do meu pai, pra meio longa né… e serve para história da surucucu, a banha dela né, porque ela mata a surucucu a jiboia, é… ela mata a surucucu. Agora não sei qual é o mistério, porque no mês de maio a cobra mais perigosa que tem né, é a jiboia.

[00:10:56]

G: E a senhora conhece quais tipos de cobras?

EE14: É… eu só conheço a surucucurana, essa que mordeu meu filho. A surucucu pico de jaca, que ela é igual uma jaca mesmo né. A corada né, e a cascavel, quela que entope… aquela que tufa o pescoço, ela pula longe né. E tem mais outros tipos de cobras venenosas, dizem né, que ela só vive no capim, só dar assim, embaixo do assoalha das casas, as vezes no capim, no roçado… elas é uma cobra que elas crescem grande, ela se trança, fica assim, um bolo… você tem que ter cuidado que só está no roçando né, tem que ter cuidado que ela ali, com os dentes dela, três passos a presa, uma pra cima e outra pra baixo. Ela é perigosa também. E tem aquele outro tipo de cobra, que chama de cobra cega também né, aquilo… eu já vi né, mas nunca vi morder né, mas dizem que é a cobra mais venenosa que existe né, que é a boiacica né, que é mãe das saúva, ela só vai para a banda das saúvas, ela é desse tamanho. Tem da branca e tem da amarela, dizem que ala é cobra cega, que ela anda sem rumo, mas ela é perigosa e venenosa né.

[00:12:07]

G: E a senhora sabe falar é… o nome de cobra na linguagem munduruku?

EE14: É… piabu…

G: Piabu?

EE14: É… piabu…

G: E a senhora é… o nome de cobra é para todas as cobras ou cada cobra tem um nome munduruku?

EE14: Não. A nossa munduruku aqui é essa, piabu. Agora dos outros sateré, outros tipos de indígenas, tem outros nomes de eles falarem né.

G: Uhum…

EE14: Agora a nossa é piabu, que é o nome dela, na língua munduruku.

[00:12:37]

G: A senhora realiza algum cuidado em pessoas envenenada por serpentes?

EE14: É?

G: A senhora já realizou algum cuidado com alguém?

EE14: Para que cuidado com eles?

G: Não, o cuidado… a senhora já realizou, igual que a senhora fez com seu filho?

EE14: Ah… tem.

G: Já cuidou de mais alguém?

EE14: Já cuidei, mais porque quando a gente avisa. Porque se a gente tá entrando em contato com uma pessoa, a gente tá na responsabilidade daquela pessoa né, se ele piorar, o problema é dele né… porque quando ele tá na convalescência assim, a gente dá o veneno pra limpar o veneno…

G: Mas a senhora já deu esse remédio para mais pessoas?

EE14: Já… eu já dei pro meu filho, o purgante da magnésia né, com azeite doce, no chá do mastruz com amocrescido e o jambú e o cominho. Porque o cominho ele é muito bom… cominho planta né, ele é bom para tudo. Aí a gente já dar, que é para purgante, é tipo um purgante, para alimpar tudinho, vai enfraquecendo o veneno todinho.

[00:13:35]

G: Quanto tempo mais ou menos de demora para senhora preparar?

EE14: Não, se eu der hoje, prepara hoje, toma… aí vai entrando em resguarde. Não pode comer peixe né… oito dias já está liberado.

G: Mas assim, horas…, quantas horas a senhora demora pra preparar?

EE14: Para preparar? O tempo do preparo é rápido né. Eu faço o chá, deixo dois dias no copo, aí eu ponho, uma colher de azeite, e dois ou três de magnésio, porque muitas vezes ataca o fígado humano, de muitos remédios né. Aí o azeite doce ele é para alimpar, eliminar o fígado né. O magnésio também. O magnésio que as vezes dar dor no estômago da gente, aí o magnésio vai fazendo uma limpeza, e desce todinho.

[00:14:18]

G: E a senhora herdou esse cuidado né…. A senhora herdou esse saber do remédio, de alguém da sua família ou quem lhe ensinou?

EE14: Não meu pai cuidava…, mas eu mesmo cuidei, pela responsabilidade minha mesmo.

G: Do seu próprio conhecimento?

EE14: Do meu próprio conhecimento… eu tratava as pessoas, fazia remédio para esses tipos de doenças né, assim… picada de bicho, tucandeira, arraia, essas coisas assim.

[00:14:50]

G: Em que momento a senhora faz esse cuidado, logo que é mordido, em que momento?

EE14: Logo quer mordido… quando chega, a gente que fazer logo também né, o tratamento logo, para ver né. A gente tem que fazer também se a pessoa vai… porque agora qualquer coisa a pessoa vai para o médico, porque antigamente não… quando era mordido a gente tinha que fazer logo os cuidados. Se tomasse o remédio, que ele precisasse, dá o remédio para não inflamar muito né, a gente dava… aplicava uma benzetacil né, ou outros tipos de antibiótico forte, pode dar a o… e aí toma o remédio contra o tétano que chamam né, que é a injeção, é o que venham tomar aqui. Depois que eles coiso aqui, vão embora né. Meu… foi mordido aqui… minto… só passa três dias aqui, toma a injeção e já vai.

G: Por quanto dias a senhora faz esse remédio?

EE14:Pra eles? Por exemplo, três dias quatro dias né… até sete dias. Porque assim, os médicos dizem assim, você vai tomar essa medicação durante sete dias né…. Aí você para, espera resultado né. A mesma coisa tem que fazer, espera sete dias o resultado do remédio né, aí passo para outro né… a mesma coisa a gente… eu pelo menos faço assim né. Por exemplo assim, vou fazer um remédio caseiro né, aí vou usar toda hora? Não…. O remédio caseiro você faz, mas não é pra dar toda hora não. Ele tem o mesmo horário do remédio da farmácia, se você for dar muita medicação, o quê que ele vai fazer? Ele vai atacar o fígado, vai dar problema na urina, vai dar problema no cocô e entre… vai aumentar a pressão, abaixar pressão né, então, a gente tem que ter o remédio controlado…

[00:16:34]

G: E a senhora faz esse tratamento por é… sozinha ou a senhora faz junto com o médico?

EE14: Não. Que médico não, médico é eu (fala com gargalhadas) médico é eu mesmo.

G: Só o seu cuidado?

ES14: Só o meu cuidado, e meu remédio. Sabe por quê? Quando eu assistia mulher que tinha dois, quatro parteiro eu não gostava…sabe por quê? Ou bem eu, ou bem a fulana…

[00:17:00]

G: E quando a senhora é… faz esses remédios, tem algum… qual resultado dele?

EE14: Ah… tem pessoas que tem logo, eu não sei se é a fé né, porque quem cura é a fé né…muitas pessoas recebem a benção na hora. As vezes vem gente lá da outra comunidade, tem gente que tem a gente de saúde. Já tem aquelas coisas certas… pegar barriga, ver como que o bebê tá… o quê que é o bebê né, já sabe… o quê que é o meu bebê (...)? Ah, eu já digo, é mulher ou homem né… já estão na ativa, e aí o meu porto? Vai ter um parto normal, tu vais fazer um cesáreo né…, aí eu digo também assim, eu digo pra elas, falo, converso assim aí… aí o remédio pra criança, eu acredito né… tios dias veio uma menina lá do Sorva, com um menino meia noite, gritando com dor estômago, provocando o que o menino tinha comido… fez um remédio pra ele, taí aí o menino, melhorou e foi embora né. Aí por isso, que eu estou dizendo, o mesmo… a cuida com o remédio em casa, remédio caseiro…. Eu tenho assim, olha meus filhos, eram pequenos, meus netos tão aí, eu não dava quase remédio da farmácia para verme, essa que chama ameba, que é a tuchina, eu trato isso com remédio caseiro, e não cria verme em criança não. Agora menino, como eu tava dizendo, porque ela acostumou o menino na doçura né… a gente… o hortelãzinho com amocrescido, o mastruz, o alho, a senhora pode pilar o mastruz com o hortelãzinho e o alho, bote no sereno, quando for de manhã, a senhor a bote num paninho, tira aquele sumo, dei no copo, bote uma colher ou duas colher… pra adulto é duas colher, de leite moça, pra criança uma colher. Aí bem aquele leite, em jejum, logo de manhã, pode dar.

[00:18:54]

G: Esse material todo que a senhora usa para fazer o remédio, é de fácil acesso, fácil encontrar?

EE14: É… fácil encontrar para mim, porque eu tenho plantado em casa né. A senhora pode tirar de manhã, bate e pode tomar, não toma café, põe no fogo, por exemplo se der sete horas, oito horas da manhã, não dar café. Aí quando for onze horas, alimentar… aí é outro pouco né, quando for na hora do jantar, lá antes de dormir, dá o restinho e pronto.

[00:19:54]

G: E quanto tempo a senhora…?

EE14: Aí passa três dias, a senhora espera o resultado.

G: Quanto tempo a senhora leva para encontrar o material que tem aqui na sua casa?

EE14: Ah, aqui na minha casa é rápido. Porque eu tenho meu hortelãzinho, eu tenho o mastruz, todos os ingredientes eu tenho né.

[00:19:37]

G: E da cobra, o que a senhora já usou?

EE14: Eu já usei vários tipos de remédio para cobra.

G: Que parte da cobra?

EE14: Da cobra, eu tenho é… até a carne dela serve pra curar a mordida dela. Quando ela morde assim, pode tirar a carne do pescoço, emplastra em cima, chupa o veneno todinho, chega fica preto. Aquilo a senhora vai jogar dentro d’água porque, porque foi assim que matou a cobra venenosa, ela colocou a bola do veneno, aí o foi, pegou, jogou n’água, quando ela voltou, ela estava bebendo água na beira, ela rodou, rodou até morrer, porque ela não encontrou a bolsa do veneno que ela deixou lá né. É por isso, que quando a gente bota o emplastro em cima, que aquilo está, você não pega, bota o emplastro em cima e vai jogar n’água. É mesmo com a timbó. Timbó botou, ela fica doidinha, rondando… que o timbó é um veneno forte, mata a arraia, você só fica e tchá… na arraia.

G: E qual outra parte da cobra a senhora já usou?

EE14: Da cobra? Por exemplo…

G: Usou a banha? (Exemplificando)

EE14: Usei a banha…

G: O couro? (Exemplificando)

EE14: O couro. O couro é bom para defumar né. Para réu não tem, disque que para mais difícil; pra macumba também. Pega o couro dela, queima e passa em cima da rasgadura.

G: E no caso de mordida?

EE14: De mordida…

G: A senhora usa também?

EE14: Pra emplastrar em cima?

G: Pra defumar ou pra… (exemplificando)

EE14: Não. Ela… o couro dela… a gente só uso outro tipo de remédio. Isso daí só serve pra negócio de rasgadura, desmentidura né, pra fazer esse trabalho… paara reumatismo. Queima o pó dela bem coisa e bota em cima. Quando a pessoa sente que tá com mal olhado, faz a defumação na gente, faz defumação na porta da casa. E a banha dela é muito boa. A minha cumadre tirou um litro de banha, de cobra ela vai me dar um pouco, eu tenho lá em casa também. Porque aquilo serve para tudo, para reumatismo para passar né. Essa tenho essa grande, eu estava até olhando, disse será se foi esse remédio que eu comprei, menina, um dor nos ossos, uma dor nos ossos, meu Deus do céu. Aí eu comecei tomar o remédio, o médico ortopedista passou a medicação né, mas não é dessa daqui. Eu comprei, setenta e dois reais…

G: E… a senhora usa mais alguma parte dela para tratar?

EE14: da cobra?

G: É…

EE14: Sim!

G: É?

EE14: É… porque tem que ter os cuidados né, por exemplo aonde ela… a gente ver, a gente presente, muitos não presente, mas eu presente onde a cobra está, e eu pressinto onde ela mora, e aonde ela passa. Porque você sente ô pitiú dela. A gente é… que chamam… pra pitiú ou a catinga dela, chama de pitiú. Onde vocês passam no mato, que vive ver, pressentir, pode ir. Aí o mais perigo é quando ela sai, que entra o som assim, ela sai. Aí a noite, é luar, ela sai para cantar, na porta do buraco ela fica cantando, ela canta igual aquelas corujinhas, você já sabe que ela. A gente já conhece, a gente tem conhecimento que é ela. A noite se você vê uma cobra, ela fica pendurada, aquela é cobra venenosa, pode ser uma surucucu. Pode ser uma pico de jaca. Porque a cobra pico de jaca, ela não tem, ela tem, ela não tem… repentina, ela não tem educação assim, de morder as pessoas. A surucucuna já é diferente, ela só morde se você tocar nela, se você tocar nela ela pula, a pico de jaca não, a pico de jaca uma pisadinha que ela presentir, ela pula né, ela pula. A… como é… à surucucurana não, aí só se você mexer com ela, mas se você não mexer, ela fica no canto dela. Mas o pico de jaca é diferente, ela core atrás da gente, é… é a cobra mais perigosa que tem.

G: Muito obrigado tá.

EE14: Tá…porque a coral nunca mordeu ninguém, também nunca ouvi falar que morreu gente não, morre… já ouvi falar assim, para outros lugares, já ouvi assim. A cascavel. Agora eu digo assim, porque que eles trouxeram tanta cobra as pessoas aí né, que cria cobra. Só que ela não tão com veneno né, porque o pessoal que estudam muito, eles tiram o dente delas, para ficar sem o dente (falas sorridentes). Ta bom mana, pois é…

**Entrevista 15**

**Caracterização do Entrevistado**

**Idade:** 30 anos

**Especialidade:** Pajé/Curadora há mais de 16 anos

**Informações Relevantes:**

**Informações Relevantes:**

**a)** A entrevistada embora tenha aceitado participar do estudo, demonstrou não estar a vontade para compartilhar seus saberes de cura.

**b)** Em seu relato ela mencionou a utilização de recursos da própria cobra para feitiços como a banha e a cabeça.

**e)** Ela também mencionou o uso da banha da cobra para tratamento e cura de ferradas de arraia e mordidas da própria cobra.

**d)** A entrevistada mencionou já ter utilizado o sumu da julica (Pau).

**Transcrição da entrevista**

[00.00.02]

G: É, me conte se existem histórias, lendas, mitos, contos com cobras em seu povo?

EE15: Não.

[00.00.10]

G: A senhora já teve alguma experiência com cobra aqui na aldeia?

EE15: Também não.

G: Também não?

EE15: Não.

G: Tá.

[00.00.16]

G: A cobra ela tem algum significado pra senhora?

EE15: Tem só pra quem faz negócio de mal pros outro né, aí tem. Tem gente que pega a pelha dela, .. . pega a banha dela, dente, té a cabeça também serve. Para quem gosta né, eu não gosto.

G: Então, a cobra ela, se a gente for pensar no significado dela para senhora, ele não tem um bom significado?

EE15: Não.

G: Não, ela tem um significado ruim né?

EE15: Hanram

G: Tá, entendi.

[00.00.50]

G: Ela tem alguma utilidade para senhora? Por exemplo, tem pessoas que fazem medicamento não para fazer o mal para pessoa, mas para fazer o bem usando algum material da cobra, a senhor acha assim, ou ela tem por exemplo alguma serventia? Para fazer algum, é, material tipo, uma bolsa ou um cinto, alguma coisa assim , ela tem algum utilidade no seu dia-a-dia?

EE15: Tem gente que faz né.

G: É?

EE15: Hunhum, a banha dela serve para mordida de bicho.

G: Quais bichos, por exemplo?

EE15: Aranha, arraia, até mordida mermo dela mermo de cobra, pode passar em cima.

G: Da própria cobra?

EE15: Da própria cobra.

G: E, qual a parte da cobra que é utilizada?

EE15: Para fazer o..

G: É o tratamento?

EE15: É, a banha dela.

G: A banha dela?

EE15: Só a banha dela. Agora o couro, o papai não disse como é.

G: Entendi. E é aaa, essa banha dela né que usa ela é feita também da cobra que a gente chama de venenosa?

EE15: Não, só a jiboia.

G: Só da Jiboia. Das outras não?

EE15: Não.

G: É, como a senhora falou de jiboia, aí tem aquela questão da história, tem alguma história relacionada a jiboia por exemplo, assim que a senhora conheça?

EE15: Não, tenho não.

G: Por que as vezes assim tem algumas pessoas que elas é, usam a cobra, por exemplo a jiboia branca, que chamam né, disque chama dinheiro né, mas tem algum é, que ela é atrativo, tem algum é, alguma história assim nesse sentindo assim que a senhora lembre?

EE15: da Jiboia branca só pra atrair gente mermo.

G: Pra atrair pessoas.

EE15: É.

G: Mas, o que é feito e como é feito, a senhora sabe ou já ouviu falar?

EE15: Só o couro dela.

G: O couro dela.

EE15: A pessoa toma como se fosse um banho?

G: Não.

EE15: Só botar na frente mermo.

G: Na frente da casa por exemplo?

EE15: É, do lado de dentro.

G: Aí ela chama as pessoas.

EE15: Isso.

G: Mas só da jiboia, das outras não:

EE15: Não.

G:Tá.

[00.02.56]

G: E, a senhora conhece que tipos de cobra?

EE15: Qualquer um.

G: É, a senhora sabe o nome delas? Fale para mim o nome delas?

EE15: Aquela cobra pico de jaca que falam, a jiboia, coral, aquela outra é cumtiboia.

G: Cutimboia?

EE15: Hunrum. Tem também.

G: Essas são as perigosas?

EE15: Não, cutimbóia não.

G: Não.

EE15: Só a pico de jaca e a surucucu.

[00.03.24]

G: E dentre essas qual a senhora acha que é a mais perigosa que as outras?

EE15: É a pico de jaca.

G: É a pico de jaca.

EE15: É.

[00.03.29]

G: A senhora nunca teve experiencia com ela assim de alguém ter sido mordido pela cobra?

EE15: Só o meu primo mais criança.

G: É, mas a senhora cuidou dele?

EE15: Não. Levarão ele pra Nova Olinda.

G: É.

EE15: Eu estava grávida nesse tempo aí.

G: Entendi.

[00.03.45]

G: Então não cuidou ele nem quando ele foi e nem quando ele voltou?

EE15: Quando ele mordeu logo ele eu dei remédio caseiro para ele só.

G: E qual remédio a senhora deu?

EE15: Eu dei julica para ele.

G: Julica, o que que é a julica?

EE15: Humhum, julica é um pau igual um coro de cobra mermo.

G: E a senhora lembra como que a senhora fez esse tratamento?

EE15: Só rapei, rapei o pau, tirei o sumu e dei para ele tomar. Puru mermo.

G: E, mas a senhora é, esquentou, ferveu?

EE15: Não, na água normal mesmo, só fiz bater, rapei,a bati com água, peguei o copo espremi lá na água e dei pra ele tomar.

G: E, e era acessível a julica?

EE15: Era.

G: Tinha perto de casa?

EE15: Tinha. Hunhum.

G: Quanto tempo mais ou menos que a senhora levou para preparar?

EE15: Acho que não levou nem meia hora.

G: E, deu imediato.

EE15: E, deu. Imediato.

G: A senhora é, esse cuidado com a julica a senhora adquiriu de alguém?

EE15: Não.

G: Não.

EE15: Eu mermo.

G: A senhora mesmo fez, e teve algum resultado?

EE15: Teve que não inchou muito. Curou mais o veneno dela.

G: E foi antes de ir pra Nova Olinda, no caso?

EE15: Foi antes de ir pra Nova Olinda.

G: E, depois que voltou não fez mais nenhum?

EE15: Não, só passei a banha mermo da joboia em cima.

G: Passou a banha da jiboia.

EE15: Que é pra sarar.

G: E a senhora mesmo que preparava essa banha?

EE15: É. Humhum.

G: É?

EE15: Por que eu tinha, a mamãe que me deu que ela tinha, eu disse pra ela que eu queria pra passar na perna dele que é muito bom, aí ela pegou ela me deu, aí eu passava toda boca da noite e vazia massagem.

G: Com a banha?

EE15: Com a banha.

G: E a senhora então percebeu que melhorou.

EE15: Hanram.

[00.05.30]

G: E, a senhora é, o algum nome de cobra em munduruku?

EE15: Não.

[00.05.40]

G: Se a senhora por exemplo quando fez esse cuidado, a senhora fez ele sozinha, né, assim, ficou só em casa cuidando ou a senhora fez ele associado por exemplo ao cuidado médico?

EE15: Não, só em casa mermo.

G: É, não chegou a ir pra Nova Olinda?

EE15: Ele? Foi.

G: Foi?

EE15: Mas ele voltou. Quando ele voltou eu cuidei dele mermo.

G: Entendi.

EE15: Durante oito dias eu continuei cuidando dele.

G: Então, assim oito dias ele estava aqui no seu cuidado, e ele passou oito dias pra ir pra lá foi?

EE15: Não, quando mordeu ele, de manhã quando foi de tarde levarão ele, os enfermeiros levarão ele, queria levar ele, e levaram ele.

G: Entendi. E e e e , da mordida até chegarem quanto tempo levou?

EE15: Pra cidade?

G: É.

EE15: Foram de lancha, foi rápido.

G: Mas chegaram aqui rápido também?

EE15: Chegaram.

G: A senhora lembra o tempo?

EE15: Mês de maio que mordeu ele.

G: Mês de maio é? Tá, e faz quando tempo mais ou menos que a senhora teve esse cuidado com ele?

EE15: Dilá pra cá?

G: É.

EE15: Tá, acho com dois ano já.

G: Ele teve alguma sequela?

EE15: Não, por causa disso daí e a banha. Ele não puxa o pé dele não ele.

G: Não ficou com nenhum problema?

EE15: Não.

G: Tá.

[00.07.07]

G: E a senhora já presenciou ou já viu alguém é, fazendo outro tipo de cuidado com recursos natural, com folha, com a própria banha do animal?

EE15: Nunca.

G: Nunca. Acho que a senhora respondeu tudo.

EE15: Então tá bom, muito obrigado.

G: Obrigada mesmo.

**Entrevista 16**

**Caracterização do Entrevistado**

**Idade:** 58 anos

**Especialidade:** Parteira

**Informações Relevantes:**

**Informações Relevantes:**

**a)** Em sua entrevista, a especialista mencionou ter conhecimento do uso da banha da cobra para cicatrização de feridas, reumatismo e para uso em feitiçaria.

**b)** Os cuidados a pessoa envenenada por cobra são comparados aos da mulher na fase do puerpério, durante 40 dias.

**c)** Entre os cuidados foram citados ficar dentro de casa, não sai pra andar fora, evitar vento, chuva, sereno, tomar banho dentro de casa e não receber visitas de outras pessoas durante tratamento.

**d)** Entre as restrições alimentares foram relatadas os alimentos remosos.

**Transcrição da entrevista**

[00:00:11]

G: Me conte se existe histórias, lendas, mitos, contos com cobras em seu povo?

EE16: É por exemplo assim, picada de cobra?

G: Assim, se a senhora já ouviu contarem uma história pra senhora?

EE16: É dizem que tem cobras que, é..., assim do mato e cobra da água. Que chamam cobra grande, sucuriju, jiboias, que dizem que, a sucuri, como é? O sucuriju ela engole negócio de cachorro, galinha, pato, tudo isso ela consome, ela mata para comer.

G: Mas a senhora já ouviu os antigos contarem, eles contam muito sobre encanto, essas coisas, que apareceu tal cobra, que encantou o fulano de tal em algum lugar, a senhora já ouviu?

EE16: E já...

G: Conte ai para a gente...

EE16: É a cobra, o nome da cobra é Norato né, aí ele encantou uma menina, que o nome dela era Branca, ai ela foi tomar banho, ela era média de nascença, ela foi tomar banho, ela tirou a roupa dela e caiu n’água e não voltou mais. Ai foram procurar os pajés e falaram que ela estava num encante com esse Norato. Ela se encantou e não voltou mais, até hoje.

[00:01:51]

G: Você já teve alguma experiencia com cobra aqui na sua aldeia?

EE16:…É com?

G: Já aconteceu algum fato assim por aqui de envolvendo cobra, assim, com alguém daqui, a senhora já ouviu falar?

EE16: Não, ouvi falar que, parece que alguém viu uma cobra de noite boiada no rio, ai botou essas pessoas para correr porque ficou com medo, é mais ele viu aquele torrão, parece um toro de pau, aí só quando eles viram aquilo eles voltaram da canoa e encostara na berada. Aquele grande banzeiro na canoa deles, ai quando olharam para lá não viram mais.

G: Sumiu?

EE16: Sumiu.

[00:02:39]

G: As cobras cobras possuem algum significado particular para você?

EE16: Acho que nesse caso é, é..., como assim?

G: Significado assim, que a senhora, as vezes as pessoas têm medo, como lhe falei, para gente nossa família tem um significado, já a cobra em particular, o quê que ela significa?

EE16: É uma cobra é uma coisa que a gente tem medo né da picada dela e ela é perigosa né, e leva até as vezes quando ela pica as pessoas, a gente, tem uns que até ela mata. E a gente tem medo de vê uma cobra assim, é mais quem quer matar assim, se ela mata a gente.

[00:03:31]

G: É, as cobras possuem alguma utilidade no seu dia a dia?

EE16:.., utilidade?

G: Utilidade, se a senhora já chegou a produzir algum remédio, a senhora tem na sua casa bens ou fins?

EE16: Sim, a banha da surucucu, serve para matar feridas brabas, que dizem que é, é coisas mandada e a banha do sucuriju serve pra reumatismo, para sarar umas ferida braba, pra alguma, remédio assim caseiro, para tudo isso serve.

G: Mas a senhora tem na sua casa, ou utiliza?

EE16: Eu tenho.

G: Mas utilidade dela?

EE16: E é muito procurada a banha do sucuriju.

[00:04:31]

G: Quais cobras você considera mais perigosa?

EE16: É a surucucu.

G: Surucucu, por que?

EE16: Por que ela é mais perigosa, por que ela as vezes quando ela não mata, ela aleija.

G: Ah considerada a mais perigosa.

EE16: Ela é a mais perigosa.

[00:04:50]

G: Quais as cobras você considera, as cobras possuem nome na linguagem Munduruku, ou é chamada de cobra mesmo?

EE16: Tem, tem é chamada na língua indígena, eu não sei como é que chamam, mas ela tem um nome.

[00:05:11]

G:Você realiza algum cuidado em pessoas envenenadas por serpentes, a senhora já cuidou de alguém que foi envenenado ou picado por cobra?

EE16: Eu ajudei a minha mãe a cuidar do meu irmão, ele foi picado por uma cobra, a gente cuidou dele em casa, a gente, é 40 dias que a gente cuida do picado pela cobra, a gente cuida dele que nem a gente cuida de uma pessoa que ta de resguarde. Ai ele não come muitas comidas remosas, tem que cuidar, só a gente tem que cuidar, não em que a receber visita de pessoa, só quem tá cuidando pode visitar.

[00:05:52]

G: Você poderia me relatar quais cuidados realizou, como realizou e o tempo de preparo?

A senhora disse que cuidou do seu irmão não é, como que a senhora fez, cuidou dele em casa, como foi realizado? Tempo de preparo, por que quando a gente faz um remédio, a gente sabe o tempo de preparo que a gente em para fazer?

EE16: Até porque antigamente não era como é hoje, qualquer pessoa picada de cobra vai para o hospital. Ai antigamente não, a gente levava para nossas casas, ele era guardado em casa, a gente cuidava dele, ai com remédio caseiro, com, banhos de, sumo de árvores, remédios caseiros, com o sumo de castanha, soro, banha de cobra mesmo, essa sucuriju, fora outros remédios caseiros, que até já esqueci o nome. Mas a gente cuidou dele ate 40 dias em casa.

G: Mas pra fazer esses preparos demora muito?

EE16: Não, demora muito não. A gente bota em cima, ferve, faz o chá pra tomar, tomando.

[00:07:31]

G: Esse cuidado você herdou de algum familiar ou de algum especialista de sua etnia?

EE16: Bom, era minha mãe, ela falava que era minha vó que ensinava ela, remédio caseiro, antigamente era, só era remédio caseiro, a gente não ia pra outro lugar. Era na nossa casa, ai ela ia me ensinando, um pouco que ela aprendeu, ela me ensinando fazer.

[00:07:56]

G: Esse cuidado é realizado em qual momento, por quanto tempo, no caso esse cuidado as pessoas fazem quando a pessoa é picada?

EE16:Sim, logo que a pessoa é picada de cobra, é a gente tem que lavar bem com sabão, o local lá, lavar bem com sabão. Ai depois que a gente vai botar o remédio caseiro em cima.

[00:08:28]

G: É o tempo de preparo, quanto tempo mais ou menos vocês observaram que teve sucesso o remédio que vocês fizeram?

EE16: A melhora né, com 8 dias a gente já, a pessoa picada, que os remédios que a gente faz, já ta ok. Já se sentava, já andava, já falava um pouco. E a gente continuava até completar 40 dias.

G: Com 8 dias já tava ok.

[00:08:57]

G: Esse cuidado é realizado em qual momento e por quanto, ( já perguntei).

G: Você realiza os cuidados de maneira isolada ou em associação com os cuidados médicos?

EE16: Isolado.

G: Isolado?

EE16: Dentro de casa, não sai pra andar fora, pra pegar vento, chuva, sereno, tomar banho dentro de casa e não tem ninguém que possa visitar enquanto ele tá naquele momento la.

[00:09:27]

G: E a alimentação?

EE16: A alimentação, não é todo tipo de alimento que ele possa comer né, peixe, carne se for uma carne bem leve, que não seja muito forte, remosa.

[00:09:47]

G: Os cuidados que você realizou houve alguma melhora no quadro da picada da cobra?

EE16:Sim, por exemplo, é a gente, logo que a pessoa foi picada, a gente fazia logo processo do remédio ai as dores ne, sentia muita dor, aí logo remédio fazia efeito logo, ai com 24 horas já tava sem a dor, já tava bem.

G: Já amenizava a dor né?

[00:10:17]

G:Os recursos utilizados nesta prática são usados com facilidade? Remédios que vocês usaram no caso, os remédios medicinais que vocês fizeram, foi encontrado com facilidade ou tiveram que sair de onde vocês moravam para procurar em outro lugar ou já tinha ali mesmo reservado?

EE16: Sim a gente saia, um tempo antes era, ali no polo, e hoje em dia falam polo. Antes o nome lá era, esqueci o nome, posto, posto médico. Ai a gente ia para lá né, pegava a pessoa que era responsável lá, para utilizar os remédios para a gente cuidar dele em casa.

[00:11:01]

G: Quanto tempo você ou a pessoa que teve os cuidados teve acesso aos recursos utilizados nesta prática?

G:Quanto tempo assim, vocês foram para cuidar dessa pessoa, vocês foram atras dos remédios, demorou quantas horas mais ou menos para se deslocar até la?

EE16: Sim, quanto a gente foi, ele não morava com a gente né, ai, ele morava ali para cima, quando a gente soube que ele foi picado pela cobra, a gente foi buscar ele lá,ao de canoa né, naquele tempo a gente não tinha motor, rabeta não tinha, a gente foi remando de canoa buscar ele, para trazer para lá, para onde a gente morava.

[00:11:43]

G: Quantas horas mais ou menos, foi dias?

EE16: Foi não, foi, um dia só.

G: Tá.

[00:11:52]

G: Entre esses recursos a senhora já presenciou ou utilizou algo da própria serpente para realização desses cuidados? No caso a senhora, foi utilizado o que da cobra para fazer esses processos de, do remédio?

EE16: Bom sei que uns, as pessoas antigas falavam que, quando a pessoa é mordida de cobra, eles tiram um pedacinho do rabinho da cobra, mata a cobra, aí tira um pedacinho do rabinho da cobra e dá para a pessoa engolir, ai pega a outra parte, tira aquele bucho né, bota em cima da mordida, onde ela morde. Ai, a gente amarra com uma corda, se for na perna a gente amarra aqui em cima com uma corda pra quela dor, porque, não posso passar né pro outro local.

....

G: Muito obrigada

**Entrevista 17**

**Caracterização do Entrevistado**

**Idade:** 42 anos

**Especialidade:** Pajé e pegador de desmentidora

**Informações Relevantes:**

**Informações Relevantes:**

**a)** Nos tratamentos para mordidas de cobra, o entrevistado mencionou ter usado a rapa do cupu e a banha do jacuraru.

**b)** Entre a dieta alimentar foram mencionados alimentos retritos como: Jatoarana, piranha, branquinha, negócio de peixe liso, porco. paca, cutia, branquinha.

**c)** O entrevistado relatou ter utilizado o rabo da cobra em cima do local afetado com a mordida.

**d)** Durante a entrevista, o pajé também mencionou a utilização do couro da surucucu para feitiços.

**Transcrição da entrevista**

[00:00:02]

G: Vamos lá, eu estou aqui com o Donizete, ele que tem conhecimento de remédios medicinais, ele pega desmentidura, costura rasgaduras também, né? Então vou aqui iniciar com as primeiras perguntas, tá?

[00:00:20]

G: Me conte se existe história, lendas, mitos, contos com cobras em seu povo? Não

EE17: Existe, né? Porque a cobra, ela é um tipo de animal que quando morde a pessoa, a primeira coisa que vem é o cansaço e logo que dá no corpo da gente, a perna da gente fica pesado e a gente tem que cuidar com o remédio. As vezes primeiro o caseiro, até chegar na, vamos dizer, na cidade assim, no hospital. O primeiro remédio caseiro que vem, quando não tem outro remédio é a rapa do cupu, você rapa bem aquela rapa do cupu ali, aquela tira aquela casca e rapa, ali amassa bem no caneco e pode tomar. E o resto pode passar em cima, fora desse daí, muitas vezes o cara tira o pedaço do, assim, da parte do rabo dela, parte, assim, né? E pode botar em cima da cisura dela, aí pode continuar passando ali o remédio ali do suco do cupu ali, da rapa do cupu, né? Em cima da cisura e tomando, aí o outro remédio, o leite condensado também, é a primeira remédio também pra combater o veneno. Pode tomar assim, misturar assim, botar assim em meio copo, puro mesmo, e pode tomar. Aí, tem o remédio também, a banha do jacuraru, a banha da paca, misturada com a banha do jacuraru, você pode misturar ali e tomar. E passar em cima também, né? Assim, onde dá a mordida da cisura dela, a cisura cobra, e pode botar em cima. E tem um remédio também, que é a folha do mato, que chamam pra ele de, esqueci o nome do (...) É um remédio, o mato assim que lhe dá uma folha assim mole, e é meia preta folha dele. Aí aqui também, o cara pode amassar ele ali, misturar aquele sumo ali, e toma um pouco ali e pode passar em cima. Que é muito bom, que é o veneno que o jacuraru come aquela folha para ele brigar com a cobra. E a cobra, ele brigando com ela, onde tá perto daquele mata ali, ele come, ele leva e volta de novo para brigar com ela. Quando ele tá enfraquecendo as forças dele, ele volta de novo, come aquele mato, até que ele vence ela. Ele mata ela.

G: O jacuraru?

EE17: O Jacuraru.

[00:03:13]

G: É, mas assim, o senhor tem conhecimento de histórias, lendas, mitos com o cobra aqui no seu povo? O senhor já ouviu contar alguma história que aconteceu?

EE17: Já.

G: Me conte aí, por favor.

EE17: Aqui, o amigo, que até se foi, ele contava aqui pra ele se garapé aqui, que vinha um temporal, né? Aí, ele contando que ele estava até esperando, aí ele voltou e embarcou da canoa e disse que ele vinha remando assim. Colega, quando ele deu, disse que foi perto assim. O colega, aquela monstra cobra assim, Sicuriju , disse que o bicho estava lá tirado no garapé. Ele deu com o canoa dele em cima e ele voltou e disse que ele só não pegou ele porque a cabeça dele estava boiada assim, distante. Acho que ele dobrou assim, pra ir atrás dele, saiu fora, disse que ele encostou na beira e correu.

G: Ela fugiu?

EE17: Fugiu. E aqui mesmo, eu já vi dizer ali no pacas, os caras já viram umas três horas. Ela também, mas boiada. Você rola de bota, diz que na larga dela, fica bicha espelhar assim no sol. Aí, onde chama o tal de torradinha.

G: Para lá, para dentro.

Ee17: Aqui, para dentro. Também lá no baixo do cacó, eles chamam a volta do bicho, ele também, o cara já viram lá. Era umas quatro horas. O bicho em cima assim, estava cheio assim, numa ponta, né? O bicho já estava boiada. Os caras, elas ficaram com medo. Esse bicho já estava aquele pedaço assim dela boiada lá no rio.

G: Acho que até hoje existe por lá, então.

Ee17: Existe mesmo lá. Ela que chamam a volta do bicho, ela...

G: Mas o senhor já foi lá?

Ee17:Já. Já foi lá, lá tem uns buracos, lá o já estava, com o já estava limpo lá.

[00:05:07]

G: Você já teve alguma experiência com cobras aqui na aldeia? O senhor já teve alguma experiência?

EE17:De...

G: Se o senhor já viu, já passou por um momento assim que o senhor ficou tipo assim com medo, viu ela, então já viu alguém ter sido picado por ela?

EE17: Eu já fui também picado de cobras.

G: Já?

EE17: Já. Dá muita sede na gente. O meu parceiro aí do (...) aí, que é o que trabalha aí na água ainda.

Ele já foi mordido também de cabra aqui pra dentro desse... Que do... Que era garapé do acre. Ele chegou... Chegou meio... Já lascado mesmo aqui. Bem...

G: Estava sozinho para lá ele.

EE17: Estava sozinho aí. Até onde a gente já tá chorando mais. Queriam até dar banho nele ainda. E para a picada de cobra, o banho não presta não.

G: Se tomar banho o que acontece?

EE17: Rum, se tomar banho Deus o livre, na hora a pessoa...

G: Morre.

EE17: Morre mesmo. Se não fosse o meu pai, já tinha dado banho nele. Ele tinha... uma hora dessa, ele não estava contando mais estrela não.

[00:06:23]

G: As cobras possuem algum significado particular para o senhor?

EE17: É, é possível, porque a gente conhece vários tipos de cobra. Tem umas que a cobra que mais venenosa que tem só é a surucucu, que é considerada aqui do Amazonas, a surucucu pico de jaca. E segundo vem a surucucu branca, que chama jararaca. Aí essas outras... A gente conhece a jiboia, tem as outras... A sacaí.

G: Qual que é essa sacaí?

EE17: A sacaí é uma cobrazinha assim, fininha, sei lá, bem fininha mesmo. Ela tem toda a cor. Tem umas azul, tem umas pretas, tem umas cinzentas.

G: Mas elas são venenosas?

EE17: São, mas não tem o veneno que essas outras tem não. A surucucu pico de jaca, essa outra jararaca que chama surucucu branca. Aí tem a cobra coral, tem aquela cutimboia.

G: Cutimboia.

EE17: Tem a...

G:Aquela pirarucu boia também, né?

EE17: Tem a... Todo tipo de cobra tem aquela que chama mãe de soba... O nome daquela cobra tem aquela pepeu aqui, aquela que chama lambadeira que ela lamba a gente.

G: Essa aqui, as pessoas se mijar onde ela passou...

EE17: É, essa daí mesmo é uma pepeu dessa cutimboia. Fazer xixi.

[00:08: 59]

G: As cobras possuem alguma utilidade no seu dia a dia? Assim o senhor já utilizou de alguma coisa dela para fazer tratamento em algum...

EE17: Já, eu tenho, eu tenho umas coisas dela, várias, várias coisas. O couro da surucucu pico de jaca, ela é muito bom para esse negócio de... Chama judiaria que faça, né? Já tratei de gente assim. Com a banha dela, o couro dela mesmo. Pode ir ali a... Queimar o couro dela bem queimado. Aí misture com a banha. Esquenta um pouco da banha dela. Pode misturar e pode passar.

G: A banha o óleo?

EE17: A banha com... Assim, com aquele couro dela queimado assim. Pode misturar ali. E pode passar em qualquer... Malefício que for assim de... Diarreia, que faça faço assim. Fica bom na hora.

G: O senhor já tratou de muitas pessoas?

EE17: Já, tratei.

G: E teve melhora?

EE17: Teve mermo. Inclusive, esse ano até o meu sobrinho. Ele veio assim que tinha um problema nas pernas dele assim. Nas pernas dele assim pra baixo. Já fazia muito isso. Tempo, né? Minha irmã trouxe ele. Eu fiz remédio para ele e ficou bom. Foi.

G: Mas e da cobra o senhor fez?

EE17: É, da cobra.

G: Com a banha e a perna.

EE17: Hum. Com a banha e a pele.

[00:09:21]

G: As cobras possuem nome em linguagem munduruku? Como o senhor? Ou o senhor conhece como é chamado mesmo cobra?

EE17: É cobra mesmo, mas ela tem o... Coisa mesmo.

G: Como é chamado, munduruku, né?

EE17: Munduruku, mas só que agora não sei no momento.

G: O senhor não tem conhecimento disso. Ah, tá.

EE17: É,

[00:09:41]

G: Você realiza algum cuidado em pessoas envenenadas por serpentes? O senhor já fez algum cuidado em alguém que foi picado por uma cobra?

EE17: Já, Pelo meu irmão mesmo que ele mora até no boa hora ali. Cobra o mordeu ele, a gente deu o primeiro tratamento com isso aí. Tô falando da rapa do cupu. Da árvore mesmo, né? Pode rapar ali, botar um pouco d'água ali. Tira aquele suco assim. Aquele sumo bem grosso ali, pode dar pra ele tomar ali. E o resto do sumo pode passar em cima, ficar passando em cima.

G: O resto de quê?

EE17: Do sumo da rapa.

G: Ah, então quer dizer que faz, aí a pessoa toma e ao mesmo tempo passa em cima.

EE17: Passa em cima, aquele sumo, né.

G: Que é de sumo, né?

EE17: O sumo, quer dizer.

[00:10:30]

G: Então o senhor já fez, já realizou os cuidados, né? Já realizou. E qual o tempo assim de preparo pra fazer esse remédio? É rápido assim?

EE17: É, tem que ser meio rápido. Porque assim, se passar 24 horas, quando ela tá muito venenosa, aí não tem mais jeito não. Porque passa o remédio, o veneno dela no corpo da pessoa. Tem que ser muito rápido para fazer esse remédio. Aqui tem umas que ela é venenosa, quando ela morde a pessoa, com poucos minutos aí, a pessoa não consegue mais enxergar nada não.

Vai perdendo a força da perna, perdendo a força do corpo, vai paradiando tudinho.

G: Qual é aquela, compadre, que ela faz a pessoa sangrar tanto no dente, pelo nariz, que quando ela seca também? Porque aconteceu o caso de um primo meu, né? Que ela foi mordida, secou a perna dele, teve que tirar.

EE17: Essa daí é essa surucucu pico de jaca.

G: Pico de jaca.

EE17: Essa daí que é a cobra mais venenosa que tem aqui no Amazonas, essa daí. Essa daí, quando ela morde a pessoa, tem um ditado. Quando ela não mata, ela aleija. Eu tenho um primo lá no Sorval que a cobra mordeu ele, e essa surucucu pico de jaca mesmo. Ela mordeu ele naqueles tempos, e ele só não morreu porque não era só a hora dele morrer mesmo. E eu fui pra Manaus. Essa perna dele ia apodrecendo tudinho. Aí o médico cortou daqui, assim, nas pontas dos dedos dele. Até no toco da coxa. Mas partiu esse pra tirar tudinho, aqui. Ele ficou meio sem com a perna dele de um lado.

G: Foi.

EE17: Até o agente de Saúde, lá do Sorral, agora ele é, o (...).

G:Não conheço.

EE17: E aí um dia que ele chama, já mostra aí como que ficou a perna dele. Os caras falaram que o médico era bom, porque se não fosse, tinha torado a perna dele.

[00:12:21]

G: Esse cuidado que o senhor, esses tratamentos que o senhor faz, o senhor herdou de algum familiar ou especialista daqui da aldeia, ou de outro lugar onde o senhor morou?

EE17: Eu já aprendi assim com as pessoas, né, que tem esse mesmo dom que eu tenho, eles me ensinaram. Aí falaram para mim, um pouco o cara sabe, a gente pode trabalhar com esse negócio de tratamento, assim, ou de cobra mesmo.

[00:12:51]

G: Ah, tá. É, você poderia me relatar quais cuidados realiza, não sei só esse de tratamento, no caso de picadas de cobra, ou se tem outros que o senhor realiza e o tempo de preparo, no caso, é preciso a pessoa ficar aqui na sua residência, o senhor vai visitar, e quanto tempo mais ou menos passa por esses tratamentos?

EE17: É, é mesmo o cuidado da mulher, né? de resguardo, é o que chama assim do coisa, né? Uhum. Quarenta dias. A pessoa, depois que é picada dessa cobra aí, que passa as coisas, e fica bom. A pessoa, não é qualquer pessoa que visita, não. Aí fica tipo assim, uma pessoa fica tipo isolada ali, só mesmo a família está lá. A gente, até as pessoas chegarem assim, o cara, eles metem qualquer algodão no vidro pra ele não estar escutando com o resto. E a pessoa, quando tem o olho, coisa para essas coisas assim, a voz, até a voz, se lhe prejudica. As senhoras, que é assim, uma coisa que tá picada de cobra assim, se lhe ouvir a sua voz, escutar o bicho dói lá onde ela mordeu. E aí, para assim, pra fazer mal é na hora. Eu sei do um colega meu, que é até marido da minha prima. Ele contava que ele mordeu ele assim mesmo. Contava que ele ia ir no, fora da barra mansa. E esse colega, ele tava lá, esse colega, ele escutou, uma tia dele mesmo, lá, esse colega, mas ele escutou naquela hora assim, mas que ela tinha um mordido ele. E esse aqui, a perna dele foi roxiando, tudinho. E esse colega foi bater em Nova Olinda também, disse que os caras sarjaram na hora. Também disse, colega o médico disse, se passasse mais um pouco, já era. Não tinha mais jeito. Não tinha mais jeito, não.

G: Pra ele ou ele ia perder a perna?

EE17: Ele ia perder a perna, pra ele mesmo, que ele ia pegar o negócio do teto, né? E ele falava, colegazinha, doeu demais, daí.

[00:15:07]

G: Esse cuidado é realizado em qualquer momento e por quanto tempo, compadre? O senhor já, praticamente já? É, você realiza esses cuidados de maneira isolada ou associação, cuidado médico? É, no caso, o senhor fica somente o senhor e a pessoa que tá picada ou vem alguém do polo para lhe auxiliar?

EE17: É, se tiver alguém do polo assim, né? Que ele pode coisar, né? Aí pode vir. Agora, a pessoa assim, né? Qualquer pessoa que pode vir também, né? Visitar, porque é um negócio sério mesmo. E outros tipos de comida. Tem que ser comida assim que a pessoa tem que ser comida escolhida. A jatoarana, piranha, branquinha, negócio de peixe liso, mantos, essas coisas assim, porco. Negócio que roia assim, paca, cutia, nem se mete. E vai fazer mal.

G: A branquinha é reimosa.

EE17: Rum, Deus o livre, você não dar nada pela branquinha, é um peixinho lindo todo. Tudo isso faz mal. Pela mordida de cobra, aí só uma cará, uma traíria, pássaro assim Nambú, e o macaco também aqui, a guariba, né? Esse tipo de macaco prego, esses outros tipos de macacos assim, caiarara que fala que for for comer, Deus o livre.

G: É, faz mal.

EE17: Faz mal mesmo.

[00:16:42]

G: É, dos cuidados que o senhor, é, que o senhor realizou, houve alguma melhora no quadro da picada de cobra?

EE17: Teve mesmo, muita, porque, eu cuidei do meu irmão, eles vieram de lá, chegaram aqui no Cipozinho, já fui para o Cipozinho, pra Nova Olinda, porque tinha que tomar o remédio mesmo aqui, assim, de lá, mas não era mais necessário ir para lá.

G: Qual foi o seu irmão, compadre?

EE17: O Valdeir.

G: Ah,

EE17: O Espanha.

G: Espanha. Ah, tá.

EE17: Ah, tem uma cana também, que chama aquela cana preta também, ela é muito bom para esse remédio aí, para picada de cobra. Pode bater bem ela, tirar o sumo, pode tomar. Tem muito remédio caseiro que a gente sabe, da medicina, assim, que faz esse tipo de remédio, que naqueles tempos, aí a pessoa nem precisava ir para cidade.

[00:17:35]

G: Esses recursos utilizados nesta prática, são encontrados com facilidade?

EE17: A gente encontra com facilidade, porque muitas vezes a gente caça por aí, o cara anda preparado, né?

G: Quando vocês vão caçar, vocês já andam preparados, porque eu vi uma história de uma senhora, compadre, que ela disse que tinha uma oração, que quando ela ia para o mato, ela orava e quando a cobra via ela, ela corria.

EE17: Mas isso é verdade mesmo, que muitas coisas, porque a gente anda no mato, eu nem simplesmente eu ando, mas eu não ando assim, de curva aberta no mato, porque a gente tem que preparar essas orações pra gente sair no mato, pra gente não encontrar, que é uma coisa mais difícil na vida, eu caçar por aí, encontrar esses tipos, graças a Deus, porque eu tenho a gente preparar, assim, pra defender dos bichos, né, se livrar, assim,

G: O senhor vai preparado pra isso?

EE17: É, mas se preparar para esses bichos.

[00:18:39]

G: Quanto tempo você, a pessoa que realizou os cuidados, teve acesso aos recursos usados nesta prática? Foi rápido o resultado dos remédios que o senhor fez?

EE17: Foi, do meu irmão, que ele estava onde a cobra mordeu ele, lá em cima, que eu tratei dele, né? Aí ele chegou lá na casa, já estava chorando, já era grande mesmo, eu tava com uns 14 anos, mais ou menos.

G: Ah, ele não era mais criança.

EE17: Não era, não. Aí já estava, e fomos tratando esse remédio aí. Aí, tempo que o cacique lá do farpado, aí usavam esse coisa aí, aí eu falei pra ele desse, do remédio desse, do leite moça, né? Aí nós tiremos lá um pouco, botei mais de meio copo pra ele, tomou aquele, foi aliviando com a rapa do cupu, né, que ele já tinha tomado.

G: A rapa do cupu é do cupu mesmo? Da árvore mesmo.

EE17: Da árvore. Da árvore, a gente pode raspar ali, bem raspado ali, bota na água aí, tira aquele sumo ali, que o bicho amarga demais. Aí pode tomar. Aí o resto daquele bagaço ali, o cara puxa em cima ali, da cisura da cobra.

G: A cisura é a picada, né?

EE17: É a picada que chama.

[00:19:54]

G: Entre esses recursos, você já presenciou ou utilizou algo da própria serpente para a realização desse cuidado? É, no caso da picada, se a pessoa for picada de cobra. Tem como fazer algum tratamento utilizando o próprio, a pele, ou a banha dela, ou dente, eu não sei como que...

EE17: Pra botar em cima?

G: Uhum.

EE17: É, como eu tô falando, aí o pedaço assim, tem assim pro lado da bunda dela, né? Aí o cara corta abaixo daquele coisa dela assim, aí parte ali, aí pode botar em cima.

G: É, no caso do rabo.

EE17: Do rabo. Pode botar em cima da mordida mesmo. Aí eu vejo o pedacinho do rabo assim, o cara corta e engole.

G: Engole? Sabia não

EE17: Olha, o remédio também é muito bom. É, as vezes utilizado por... As pessoas que sabem, muitas vezes não sabem. O rabo da jiboia, para quem não sabe, que pedaço o cara mata, pode engolir. Porque ele é muito próprio contra esse veneno. Porque a cobra diz que mais venenosa, o mês de Maio, isso é a jiboia também.

G: É, a Jiboia mesmo.

EE17: Mês de maio. E ela é brava, mês de maio.

G: Fica venenosa.

EE17: Fica venenosa e braba a Jiboia, todo tipo de Jiboia. Da branca, da vermelha.

G: Então, muito obrigada, compadre. Que Deus lhe abençoe. Gostei de vir entrevistar. O senhor é bem desinibido mesmo. Falou mesmo o que tinha que ter falado. Então, lhe agradeço muito, tá?

EE17: Tá bom

**ENTREVISTA 18**

**Caracterização do Entrevistado**

**Idade:** 55 anos

**Especialidade:** Pegadora de desmentidora e Parteira

**Informações Relevantes:**

**a)** Na medicina indígena Munduruku, a especialista relatou ter usado a castanha, o féu da paca, sumu do pião, sumo do cupuí e a banha do boto.

**b)** Em sua experiência a entrevistada relatou ter usado a banha da jiboia para tratamento de mordidas de cobras.

**c)** Em seu relato a entrevistado também mencionou o couro da cobra para associado a pimenta malagueta para defumar a casa e para tratar judiaria.

Transcrição da entrevista

[00.00.03]

G: É, então nós estamos aqui com a dona (...), é vou começar perguntando, fazendo as perguntas pra ela. É, me conte se existe histórias, lendas, mitos, contos com cobras em seu povo?

EE18: É...

G: É história, no caso assim os antigos sempre nos contavam né, a senhora lembra de alguma, de algum mito?

EE18: Eu sei que a pessoa se emprenhava de cobra (risos) porque tem muitas pessoas, a minha mãe mermo contavam, meu pai né, que a mulher que não se resguardavam assim nos nossos tempos né, aí ela apareceu, ela menstruou, aí ela jogava assim a roupa dela na, ao invés de guardar pela beira né, aí quando foi um tempo aí a mamãe conta que a colega dela teve duas cobrinha, duas cobrinha, disque quando ela estava pulando, brincado assim com as amigas dela né, assim como a gente é solteira né, foi solteira né, disque estava no meio da colega dela, diz que lá vem aquela cobrinha no rumo dela né, aí disque a colega dela dizia ai é uma cobra, é uma cobra. Aí disque ela sumia aquela cobra né, aí ela dizia é bem tua filha que fez filho com cobra. Aí era assim, aí disque quando ela ia dormir minha mãe contando que ela subia com a ... estava gemendo, gemendo, aí diz iam espiar, ela atravessava com a filha dela que era cobrinha, foi, até que ela levou ela.

G: A filha levou a mãe?

EE18: A filha levou a mãe. Por isso que ... tem muitos assim, quando eu menstruava, minhas filhas menstruava, tinha muito, até agora eu tenho cuidado com minhas filhas. Boto do mesmo jeito, o boto ele é sem vergonha né, essa mulher que eu paro lá, na dona Cila que ela conta, que ela morava ali aonde a Sabrina mora mais pra, o seu João conta que se engravidou a mulher dele de boto, aí ele, até hoje ele diz mermo que ele nunca foi traído por gente assim desse mundo né, foi traído de boto, e desse daí ele foi embora pra lá pra Nova Olinda, não quis mais morar aqui não.

G: Isso fez ele se mudar da aldeia?

EE18: Fez ele se mudar, diz que o boto traiu ele. E ela conta mermo que era um botinho desse tamaminho. Que ... mesmo que assistiu né.

G: Ela ainda chegou a parir o boto?

EE18: Chegou a parir o botinho.

G: É só isso que a senhora tem conhecimento? Que a senhora já ouvia falar?

EE18: Já ouvir falar, já ouvir falar também de ambuar emprenhar, papai que contava não era eu que contava, e a gente botava na cabeça naquele tempo a gente não era demais assim, os antigos contava a gente ficava escutando né, não ficava escutando assim perto porque eles não gostava mas ficava deitado aí escutando né, diz que a prima dele né que ela era bem nova em Parintins que ele morava, aí disque ela não tinha cuidado com os panos dela né, aí o marido dela ralhava com ela porque pra lá pra onde eles morava né era terra preta, e na terra preta da muito ambuar e lesma né, eu tenho muito medo de terra preta, eu não gosto de morar em terra preta não. Aí diz que quando ele ia espiar, ela ficar menstruada ela jogava o pano dela, não enterrava, ficava aquele quase dois litros de ambuar. Aí ele sempre falava para ela, mulher tem cuidado com teu pano, enterra esse pano, que o bicho, tu ainda apreces prenha de ambuar, aí passou diz o papai, aí ela foi ficando pálida já, não queria mais comer, já triste, aí levaram no curador. Levaram ela. Aí ela, o curador disse que ela estava gravida mermo, mas não era de gente, do marido dela, era de bicho, aí ele disse como ela ia ter, ela vai entrar no mês pra ela ter, aí quando ela entrou mês, o papai conta, isso daí ele contava até hoje quando ele estava vivo ele contava pra nós, contava pro meu marido, contava pra minhas filha. Aí doeu a barriga dela, ela ficou buchuda assim igualmente a gente mermo né, cresceu o bucho, aí quando entrou no mês ela se agoniou, estava doendo a barriga dela, aí o marido, que o curador falou né, quando doesse a barriga dela ela pra ele levasse ela. Aí disque quando doeu a barriga dela só que era longe, o tempo era de ... era como daqui lá pro laranjal, aí disque ele fez o giral da canoa, arrumou bem, agasalhou e botou ela no giral da canoa, ela ia gemendo, ia gemendo, aí disque quando ele olhou dona (...), o papai contava, ele olhou assim pra, ela estava deitada, ela parou de gemer né, aí que quando ele olhou diz ele, (...), ele contava pro meu marido e minha filha, esse daqui dela, por aqui pelo corpo dela, não tinha ... era preto, dona (...) diz ele, fecho, fecho disque aqueles bichos pretejo nela, tinha, ai meu Deus do céu, quando ele contava chega eu tinha era medo não tinha onde botar ... aí disque quando ele olhou né aí disque ele mexeu com ela, ... aí ele remou de novo até que ele chegou mas ela já estava morta já.

G: Aí ela morreu?

EE18: Morreu. Que ela não suportou ter aquele monte de bicho.

G: Mas de cobra a senhora só tem conhecimento daquele que a senhora falou primeiro?

EE18: Da que emprenhou a mãe? Emprenhou a menina?

G: Só né?

EE18: Só.

[00.06.19]

G: É, você já teve alguma experiencia com cobras em sua aldeia?

EE18: Experiencia, como experiencia, que morde? Já, porque esse meu cunhado que foi embora pra Manaus né ele foi mordido de cobra.

G: O Qual?

EE18: Esse, o (...), aí ele foi mordido de cobra isso daqui dele roxeou tudinho. Aí quem curou foi a mamãe, a mamãe pegou o sumo do pião, o sumo da do cupuí sem ser esse cupu que a gente toma né, a castanha que a gente come, a castanha mermo do Pará né que é, do Amazonas, aí né, e aquele outro como é já, o, a banha do da jiboia, a banha do, o féu da paca, ela fez ... aí ela deu uma colherada para ele beber. O feu da paca passou todinha a perna dele, amarrou, poucas horas ela deixou lá, aonde o bicho picou ele que ela amarrou, tirou, aquela castanha modo que chupo, estava roxinho, onde, é muito bom dona (...), esse remédio, quando não tem a coisa, pode dá a banha do boto passa a banha do boto, dá para a pessoa beber a banha do boto com a banha da jiboia junto corta logo o efeito do veneno.

[00.07.59]

G: É, as cobras elas possuem algum significado particular pra você?

EE18: ...

G: É por que a nossa família ela tem um, a gente, é pela nossa família a gente faz qualquer coisa né? A cobra assim para a senhora ela tem algum significado assim na sua vida, ou a senhora temer, alguma coisa assim?

EE18: Ah, medo dela? Não, quando eu vejo eu não tenho medo não, eu mato quando dá para eu matar, assim que tem uma vara assim que tá direita eu corto e passo o pau na cabeça dela (risos),, e quando tá muito perto aí e, assim eu chamo já os meu filho pra matar com espingarda, mas quando eu quando eu vejo elas eu não dispenso cobra não.

G: Não tem medo?

EE18: De matar não tenho medo não. Mato mermo.

[00.08.51]

G: As cobras possuem alguma utilidade no seu dia-a-dia?

EE18: Dentro de casa.

G: É a senhora já utilizou alguma coisa?

EE18: A se eu já utilizei assim minha casa, se já entrou algum bicho da, cobra dentro da minha casa?

G: Tipo assim se a senhora já utilizou é a pele dela para algum remédio?

EE18: Ah a pele dele também como ela é boa, como diz aquele lá, ela é venenosa, mas se a senhora tiver a assim, a sua casa mermo que a senhora possuir sua casa, pode ter na cidade, aí pega a aquela pele dela e a senhora pega pimenta malagueta, bote só uma não bote muito não, porque na cidade já viu né, a senhora defuma a sua casa, até mesmo na sua casa que a senhora vai morar, muito bom, a senhora saber que Deus o livre a senhora tá judiada pode defumar.

G: Mas como que faz dona (...), só faz queimar, dona (...) já?

EE18: A gente queima, faz o fogo com aquilo né, a tira aquele coro né, e bote em cima aonde tem diz aquele, judiaria que Deus o livre, ... aí se tiver a banha a senhora passe.

G: Mas a pele do qual cobra?

EE18: Surucucu.

G: Da surucucu?

EE18: Aí também mano, se ... vai embora.

G: O que acontece?

EE18: Morre.

G: Aí. A senhora já tentou fazer esse remédio?

EE18: Ainda não tentei, mas me ensino né.

G: Já lhe ensinaram, mas a senhora ainda não teve coragem pra fazer?

EE18: Não teve coragem porque nunca me judiaram assim né de coisa. Mas o dia que me judiarem. Eu tinha o coro que meu filho matou lá para onde ele morava lá para Soval o sogro dele matou um desse tamanho assim, quando eu cheguei de Nova Olinda estava secando, aí ... de pedirem coro, de pedirem couro para fazerem defumação na casa, cabo.

G: Ah. Serve também para defumar para carapanã?

EE18: Para olho gordo.

G: Para olho gordo?

EE18: Tem vez que a pessoa chega na casa da gente, como eu digo, tem muito que vai passear e tem muito que não vai passear, está conversando mas o olho está correndo no objeto da pessoa né.

G: eu sei como que é que é.

[00.11.09]

G: Quais os tipos de cobra que você conhece, que a senhora conhece?

EE18: Surucucu, jiboia, sucuriju, aquela cobra papagaia, aquela cobra petel que chamo né, aquele outro como é já, surucucu pico de jaca, surucu de fogo, tem aquela outra, jaracaca, tem a cobra cipó tudo tem, a coral, a coral é também uma mais perigosa que tem também.

G: É, assim a senhora já presenciou alguma dessa assim de perto da senhora?

EE18: A coral?

G: Alguma dessas que a senhora citou?

EE18: Já, a coral.

G: A coral.

EE18: No mato sempre quando eu com meu marido sempre a gente via ela, a gente matava com ele.

G: Matava.

EE18: Dessa outra também surucucu de fogo também nós já matemos.

G: Ela é perigosa?

EE18: É perigosa. Mordeu na hora na hora ela popoca logo.

[00.12.13]

G: Quais as cobras você considera mais perigosa dessas que a senhora falou para mim?

EE18: É a surucucurana, surucucu de fogo e a pico de jaca e essa coral que falo também, é perigosa.

G: Quais são as causas, os danos que ela causa na pessoa a senhora sabe me explica?

EE18: Quando ela morde? Essa coral quando ela morde disque ela aleija logo a pessoa, adormece logo. E aquela outra papagaia que a gente não dá nada por ela que ela amodo é besta né, mas não se fie mano que aquela também, lá no abacaxi quase matasse a (...), que ela mordeu aqui e inchou tudinho, inchou. E o remédio que é bom também dona (...) não tinha desse eu daí troxe uma vez la do Soval né, aí eu plantei trouxe um pouco de semente, eu botei no meu bolso quando cheguei eu samiei, aí eu plantei o meu dessa altura aí o bicho comeu né, aí eu pra cumpadre (...) , aí não tem aquele que chamo carrapateiro, um falo mamona outro carrapateiro né e ela tema aí a cumadre (...) . Aí quando bicho morde a surucucu, a gente num espera, nunca espera, e acontece, a gente ... aquele bracinho dela né, aí pendura no pescoço da gente, aí quando tem a folha a gente implasta, o veneno ele desincha tudinho.

G: Aqui por perto não tem?

EE18: Tem a comadre (...) tem.

G: Só ela que tem?

EE18: Só ela que tem. Eu nunca mais tinha visto esse remédio aqui ai eu peguei dei pra ela um pezinho, comadre esse daqui a gente num espera, a senhora que gosta de andar com seu cachorrinho por aí pela roça por aí pelo mato, esse aí é muito bom pra picada de bicho, uma vez o cachorro mordeu, a cobra mordeu o cachorro da vovó (...), aí veio carregada a cachorra, inchou inchou a cara da cachorra, aí minha avó tinha esse remédio, ela cortou todinho bracinho aí ela pegou enfio os colazinho, quando foi notro dia a cachorra, mas inchou mais num inchou muito, arriou todinho aquele inchaço, ela salvou a cachorra dela.

G: Ela salvou.

[00.14.40]

G: As cobras possuem nome em linguagem munduruku, ou como são chamadas na língua diária?

EE: Eu mermo, eu chamo só cobra mermo. Porque eu não sei o nome da cobra ainda vo perguntar como é que é, que o nome dos otros bicho a gente sabe na linguagem.

G: Outros a senhora sabe, mas da cobra não?

EE18: Da cobra não.

G: Não sabe né.

[00.15.03]

G: Você já realizou algum cuidado, você já realizou algum cuidado em pessoas envenenadas por, pela cobra?

EE18: Já.

G: Já. Como foi, me conte?

EE18: Ah, foi uma, foi até ela minha madrinha o nome dela é (...) né, ela estava boa já, ela não estava assim perigosa pra não estava mais... já tinha corrido passado o risco. Aí veio a sobrinha dela uma (...) o nome dela era, aí tem gente que é abelhuda né, aí disque que ela estava separada a minha ela, né, que é até mãe do, era vó, até vó dessa (...) aí do .. do coisa da, do (...), humhum, aí ela entrou lá né, mas num estava... já estava melhorzinha, estava paresque com 8 dia que tinha mordido de bicho né. Aí a mulher foi lá né, oh tia, aquela, oh tia, oh tia (...) que chamado pra ela, tia (...), oh (...) eu vim espiar a tia (...), disque ela estava sentada né, ela disse que quando ela falou disque a velha gritou, amodo que aquilo foi na hora, o sangue disque espirrou. Aí ela gritava, ai minha filha ela disse, tira essa mulher daí, manda ela sair daí ela disse, manda ela sair daí que ela vai me matar, mana diz a coisa, dona (...), a onde a mulher falou aquilo ficou negrinho quando foi 8 hora ela morreu, não chegou nem diz aqui, a hora dela, seis hora, sete hora ela foi lá falar com ela, quando foi 8 hora ela morreu.

G: E vocês não chegaram nem a fazer nenhum remédio desses que vocês tem ...?

EE18: Não, por causo que nós não tava, ela morava no mucajá e nós morava no laranjal. Aí fizeram o remédio mas não teve jeito porque ela era muito venenosa, matou na hora.

G: Meu Deus.

[00.17.15]

G: É, você poderia me relatar quais cuidados é, já realizou assim com alguém picado de cobra?

EE18: Por aqui mermo?

G: Por aqui ou em outro lugar que a senhora já morou.

EE18: Esse meu, mordido, também, ele foi mordido mas foi curado só com remédio caseiro também.

G: Foi.

EE18: Levaram ele pra Nova Olinda né só pra tomar injeção pra limpar lá, mas ele no mermo dia ele voltou, aqui mermo ele foi curado, tomando remédio caseiro também que nós ensinemos né.

G: Quais foram os remédios caseiros?

EE18: Foi o deram o sumo do pião pra ele, o su, o a banha da jiboia, e o sumo do cupuí, com esse outro, castanha né, passaram e ensinaram e ...

G: Como que vocês fizeram esse remédio? Como hoje é realizado?

EE18: A gente faz só, a gente né tira aquele sumo do algodão, aquela rapa tudinho né, aí pega o sumu do cupuí só aquele coisa né, aí pega a castanha quando tem pila ela um buncado né, aí fica só aquele leite, aí pega a banha do boto se tiver a banha da cobra, da jiboia né, banha do coisa como já, do féu da paca e amarra, aí não faz dano não.

G: Mas só é, esses remédios que vocês fazem dona Tereza é só para passar em cima, não tem nenhum chá que a pessoa possa tomar não?

EE18: E bebe também, e bebe, o caroço do abacate ó, ninguém dá nada por ele pode fazer.

G: Rala ele? Como é que faz ele?

EE18: Dá um poco para a pessoa, é ruim né, ... aí joga tudo veneno.

[00.19.12]

G: E passa muito, esse tempo de preparo é muito tempo assim que vocês passam preparando, tem algum ritual que vocês fazem ou não, é só faz?

EE18: Ele já tá pronto lá deixa lá toda hora tá passando, até ele matar a força do veneno memo e vê quando ele já tá movimentando, já não tá mais inchado aí continua passar.

G: É um tratamento:

EE18: É um tratamento. Aí pode, só se ele facilitar também.

G: Hunrum.

[00.19.40]

G: Esse cuidado a senhora herdou de algum familiar ou algum especialista da sua etnia?

EE18: Foi, da minha mãe que eu pegava experiência.

G: Me conte um pouco sobre essa história.

EE18: Não porque quando minha mãe estava assim fazendo os remédios dela, eu ficava espiando né, ela me chamava né que ela parteira também, e ela que me ensinou.

G: A senhora é parteira né?

EE18: Sou parteira ... Aí ela me ensinava, ela quando estava assim com dor de barriga, dor estomago, mãe do corpo que chama, pra puxar o estomago dela, aí eu puxava.

G: A senhora é muito boa nisso mesmo.

EE18: Fazia remédio para ela, não tem aquele vergalho da anta? Vergalho da anta? O prego da anta que chamo? Pode assar ele, la esta li um pedaço, té compraro de mim levarão para derrame. Meu cunhado ...

G: Sério! Como é que faz pra tirar derrame dona (...)?

EE18: A gente queima ele, aí procura a banha do jacaré, se a banha do jacaré, a banha do, a aguardente, aquele outro né, banha do jacaré, aguardente, a banha da guariba, se não tiver a banha mais pode queimar o pelo, queima esse daí esse daí ... queima ele, coa ele no paninho e o bagaço a gente passa nas pernas faz a fricção, aí pega a aguardente, aí o po...

G: O que que é po...?

EE18: É um que é igual uma ... ele dá por aqui, aí faz e dá para a pessoa... aí vieram atrás, e eu mandei um pedacinho.

G: Esse aí também serve para a mordida de cobra?

EE18: Não, só para a mãe do corpo e pa derrame.

G: A é? Muito bom.

EE18: Aí ela estava fazendo eu estava espiando né, ajudando ela né, eu ficava espiando, não é toda hora que minha mãe no meu poder, eu vou aprender remédio da mamãe. A por isso que quando vem minhas filhas com minhas netinhas tá doente, nós mermo daqui de casa, é difícil ir pro polo, só remédio caseiro, que eu cuido logo. Se eu ver o meu filho que tá com uma febre, uma dor, corro no caseiro.

G: A senhora mesmo prepara os remédios?

EE18: Eu mesmo preparo… e (...) diz mesmo, difícil eu ir no polo, é difícil.

G: Assim dona (...) (...), quero falar dona (...) (risos)…. (...), a senhora já se considera uma pessoa assim curadora, que no caso, é com esses remédios caseiros que a gente faz, a gente cuida das pessoas né. Então, a senhora já obteve resultado nesses remédios né. Então, a senhora já se considera alguém que cuida dessas pessoas também né?

EE18: É… aí venham aqui comigo, quando não tem eu ensino né, e vão fazer. Dois dia a menina estava com tosse de guariba, minha sobrinha. Ai peguei disse pra ela, ferve a erva do passarinho, ferve aquele hortelã peludo, mas não esfrega ele, ferve ele junto. Pega a folha da sabugueira, ferve aquele água dela, tu vai ver só, eu disse… que tua filha vai ficar bom. Ah, titia já fez tanto remédio, para minha filha, e nada… ela fez, tá aí a cuiatã, tá curada.

[00:23:03]

G: É… Você poderia me relatar quais cuidados já realiza (isso aqui, deixa eu perguntar) você poderia me relatar quais cuidados você realiza, como os realiza, e tempo de preparo? No caso esses remédios que senhora já fez para a cobra, como que a senhora realizou ele é tempo de preparo que a senhora prepara eles? Porque muitas vezes a gente vai fazer um remédio a gente conta um, dois, três minutos né, até meia hora pra preparar aquilo, a senhora quanto tema senhora…

EE18: Quando tem assim, o preparo né…

G: Uhum…

EE18: Mistura tudo junto logo, na mesma hora, pila tudo junto né.

G: Uhum…

EE18: Se tiver banha, se tiver quilo o… ou a folha. O sumo, pode fazer só uma cuiada mesmo assim…

G: Já faz tudo só de uma vez?

EE18: Só de uma vez, aí vai passando, passando… aí guarda ele, fica igual uma pomada né. Ver mesmo que já a melhorando, não deixa de não passar, vai passando. Assim que nós fazemos.

[00:24:09]

G: Esse cuidado é realizado em qual momento e por quanto tempo? No caso, como que… no caso a pessoa foi picado de cobra, aí vocês já entram com o remédio caseiro ou vocês…?

EE18: A gente dar primeiro aquele… manda primeiro eles… eles vão primeiro… o… da injeção la do polo né.

G: Uhum…

EE18: Aí quando chega lá ele, que nós vamos fazer logo nosso serviço… porque eles vão logo acudir com remédio de lá né, injeção. Aí deixa, aí já vem para casa da gente, fazer remédio caseiro.

G: Você… assim é… Os remédios… Assim, os remédios que a senhora faz, a senhora faz algum benze, algum… ou só faz remédio?

EE18: Não só faz assim, remédio.

[00:24:57]

G: É você é… você realiza esses cuidados de maneira isolada ou é associação ao cuidado médico?

EE18: É separado, isolado, assim como fica sozinho na casa. Só um cuidando né. Então, dois que vai espiar, que não é venenoso, ele vai lá espiar, aí também…

G: Mas como vocês sabem se a pessoa não é venenosa, em dona (...)?

EE18: Não porque, a gente conhece só na fala (risos) só na fala que é doído né, que a gente ver a pessoa que fala agoniado assim, amodo enjoado né, esse daí não vai espiar que é (sorrindo) eu acho que a senhora não é não (sorrindo)

G: É… você realiza é… esses cuidados, já perguntei né… isolada né?

EE18: HumHum

[00:25:43]

G: A senhora já respondeu. Dos cuidados que você realizou, houve alguma melhora do quadro da picada de cobra?

EE18: É. A gente tem a melhora.

G: Explica aí,

EE18: Porque quando a gente cuida do remédio direto mesmo né, para a picada de cobra, aí a gente ver logo o feito né. Se a gente não vir o feito, que ele tá gemendo, que Deus o livre, que dói né.

G: Uhum…

EE18: Que eu nunca peguei, mas meu marido diz que é vinte e quatro horas de dor. Aí que quando ele passou remédio caseiro né, a gente ver que vai moderando, diz ele, aí também vai… Amodo que vai arriando aquele inchaço com o remédio que vai fazendo né, aí vai arriando amodo aquele inchaço. Ai quando inteira vinte e quatro hora, passou do perigo, vai só se resguardando, não comer outras comidas remosa, até quarenta dia.

G: Até quarenta dias, é tendo uma alimentação só…

EE18: É… uma alimentação só mesmo que não comer comida remoso, comer outras coisas, só mesmo naquele… levando a dieta direto, assim, mesmo resguardado, ele não corre perigo, agora se ele facilitar, ele vai correr perigo.

G: Dos cuidados que você realizou, houve alguma melhora no quadro da picada… isso a senhora já me relatou.

EE18: E já…

[00:27:07]

G: É… os recursos utilizados nestas práticas, são encontrados com facilidade? É os remédio que senhora…

EE18: É os remédios né?

G: Aham…

EE18: É, por aqui que Deus o livre, a gente tem por aqui. Só que a gente, o que ainda não tirei, ainda não teenho é a banha da jiboia né, mas eu tinha, mas só que eu não tenho mais. Não é toda hora que a gente precisando não, mas um dia a gente precisa.

G: Qual é o tipo da jiboia que vocês utilizam?

EE18: Jiboia branca.

G: Da branca?

EE18: HumHum

G: Só a banha mesmo? A banha para fazer… juntar e fazer remédio?

EE18: É… para fazer o remédio.

G: Junta todinho para fazer o remédio?

EE18: Junta todinho…

[00:27:46]

G: Quanto tempo você ou a pessoa realiza estes cuidados teve acesso recurso utilizado, usados nessa prática? Exemplificando - Quanto tempo, se vocês foram atrás do remédio ou passaram um dia todo ou no mesmo dia encontraram?

EE18: No mesmo dia a gente encontra.

G: No mesmo dia né? Vocês já têm conhecimento né?

EE18: Já tem conhecimento…

G: Onde encontrar…

EE18: Onde encontra… Por aqui mesmo a gente planta, né. Aí já tem aquele remédio já.

[00:28:17]

G: Entre esses recursos, você já presenciou ou utilizou algo da própria serpente para realização deste cuidado?

EE18: ??

G: Qual assim, a senhora já utilizou da cobra?

EE18: Que cuidado?

G: Exemplificando… é a banha, ou pele…

EE18: É a pele… a banha, a gente já guarda já né, que assim quando os meus filhos matam, eu já tiro aquela banha. Banha de macaco, de guariba. A gente já sabe que é para remédio né, banha de macaco prego serve pra desmentidura. Banha do tatu já sabe, diz aquele… para puxar um estrepe, para puxar um… qualquer coisa.

G: Da cobra?

EE18: Da cobra também, do mesmo jeito.

G: A banha serve para quê a banha?

EE18: Serve para malefício, serve para a coisa, pra…mordida dela mesmo, passa, muito bom. Que Deus o livre, sua mãe tá judiada, mande comprar… porque pra lá pra… aí pra vargem, sempre eles tiram banha de surucucu né. Para lá dá mais do que pra cá né, sempre… meu irmão teve uma vez, tirou um cocão assim, cheio. Só que ele não falou comigo né, aí ele vendeu para uma mulher, aí de Nova Olinda mesmo, que ela queria, que era pra um malefício, a mãe dela estava judiada, aí ele vendeu para ela. A gente passa no corpo da gente, que Deus o livre, a gente tá… vai na curandeira, manda benzer, e… aí eles dizer que é judiaria né, pega banha de surucucu, e passa assim. Ela vai ensinar pra senhora, ai a senhora já tem, para fazer o remédio.

G: Muito bom, né dona (...).

EE18: Aí se for malefício, ele mesmo cai.

G: Então, muito obrigado (...). Aqui estive entrevistando dona (...), a parteira aqui da aldeia muito tempo. Como ela falou, ela é tanto parteira, como ela puxa desmentidura… desmentidura não…

EE18: Não…

G: Qual é?

EE18: Mãe do corpo.

G: Mãe do corpo e também produz alguns remédios caseiros, já salvou várias vidas aqui na aldeia, já salvou várias pessoas de está sentindo dor por aí.

EE18: Eu fiz onze parto, só aqui.

G: Aqui ela…

EE18: Minha filha,

G: Aqui na aldeia como ela acabou de falar, já fez nove partos então, é uma pessoa bem assim ativa, que também gosta de ajudar as pessoas aqui. Então, dona (...) muito obrigada, pela sua colaboração.

**Entrevista 19**

**Caracterização do Entrevistado**

**Idade:** 58 anos

**Especialidade:** Benzedora, rezadora e pegadora de desmentidura

**Informações Relevantes:**

**Informações Relevantes:**

**a)** A especialista mencionou que as práticas de cura da medicina não são realizadas como em tempos anteriores e que hoje há um predomínio o modelo biomédico.

**b)** Dos recursos naturais utilizados pela entrevista ela citou a utilização do caroço de abacate, ralado ingerir.

**c)** A entrevistada utilizou a banha a banha da cobra e o bucho para colocar no local afetado pela mordida, mencionando que o remédio serve tanto para casos com aranhas, tucandeiras ou ferradas de qualquer outro bicho.

**Transcrição**

[00:00:01]

G: Me conte se existem histórias, lendas, mitos ou contos com cobra em seu povo.

EE19: É… lá na sapucaiaroca existiu que era encantado, aí ele foi passar uma festa, aí ele arranjou uma namorada lá, aí quando estava para dar meia noite, ele disse que ia embora dormir, e a namorada não queria deixá-lo, sei que ele foi dormir numa casa lá. Aí ela não estava confirmada, ela foi expiar ele, ele tinha ido embora. Quando ela chegou lá, ela viu aquele monstro rolo de cobra, ela se assustou, ela correu, ela foi avisar o pessoal que tinha um bicho muito grande… aí que aconteceu, quando ela chegou para lá, aí que ela foi para lá, a terra foi partindo, e foi sumindo, a cidade com tudo para o fundo. Até hoje é um remanso muito feio lá, que não tem motor que passe não, tem não…esse daí só que eu sei contar, esse daí. Conhecido como Sapucaiaroca, aí pro lado de cima, mas é verdade mesmo do (...) isso daí.

[00:01:09]

G: É… você já teve alguma experiência com cobra na sua aldeia?

EE19: De cobra assim? Mordida assim? É por causa de remédio caseiro, ninguém sabe fazer mais. Só se for remédio de farmácia, injeção. E outra, por aqui, remédio caseiro só caroço de abacate, ralado pessoal tomar, e passar a banha dela em cima da cesura, e é só também que eu sei explicar.

[00:01:44]

G: As cobras possuem algum significado particular pra você?

EE19: Eu tenho muito medo de cobra, nós tudo temos medo de cobra. É porque quando ela morde a gente, muito difícil a pessoa escapar. É por isso, que a gente morre de medo de cobra. Eu tenho medo, só os outros também tem. É só isso, ela só vive escondida, ela traiçoeira, ninguém enxerga ela, é só isso mesmo.

[00:02:28]

G: As cobras possuem alguma utilidade no seu dia a dia?

Explicando (se a senhora sabe, é… se a senhora já usou a banha ou alguma coisa dela)

EE19: Por causa que assim, a banha dela, partilha e passa na Cisura dela, que é na mordida. No conhecimento da gente é Cisura. Na coisa ditei, é mordida. Aí tira a banha, passa em cima, puxa com catauari, que é pro inchaço não subir para cima. Aí puxa com catuari aí pronto, baixa o inchaço da bicha, da mordida.

[00:03:12]

G: Quais os tipos de cobra que você conhece?

EE19:Surucucu, sucuriju, jiboia, essas cobras verdes, é… é só essas daí mesmo.

[00:03:29]

G: Quais as cobras você considera mais perigosa?

EE19: É a surucucu.

G: Por quê?

EE19: Porque ela morde e é muito difícil a pessoa escapar. Só quando tem injeção mesmo, assim, na cidade que leva, se for longe a pessoa morre. Não tem remédio né, não tem injeção. Porque o remédio dela é injeção.

G: Mas, antigamente (...), existia algum tipo assim, de curador, no caso, a senhora tem conhecimento assim, fazia remédios para cura das picadas de cobras?

EE19: Acho que não tinha não, acho que não tem não. Se tinha, não era do meu tempo, antigamente eu acho que tinha, ainda não era do meu tempo, ainda não. Agora o pouco tempo, como diz a coisa… que eu me coisei no mundo, não vejo dizer não.

[00:04:24]

G: As cobras possuem nome em língua munduruku ou como são chamadas na língua diária]

EE19: É… na língua munduruku não sei chamar, mas na diariamente é cobra mesmo.

[00:04:38]

G: Você realiza algum cuidado em pessoas envenenadas por serpentes?

EE19: O rendido é esse daí, como a gente estava falando. Tem esse caroço de abacate ralado, tem o chama bem-te-vi-caá, a gente pila, puxa, o caroço do abacate, é ralado pra pessoa tomar, e esse bem-te-vi-caá pra picar o inchaço pra baixo com catauari, pode fazer que é bater e ver mesmo.

G: Só esse que a senhora tem conhecimento?

EE19: Só esses mesmo que eu sei.

[00:05:20]

G: Você poderia me relatar quais cuidados realiza, como realiza e o tempo de preparo? No caso esses remédios que a senhora citou, qual o tempo de preparo, como ele é feito… tem como a senhora explicar?

EE19: É porque é pilado com um pouquinho de água, é pilado bem pilado no pilão, mistura tudinho isso daí e pila, aí passa no inchaço, para não inchar. Se for no pé, o inchaço vai para cima, aí pra não ir pra cima a gente puxa pra baixo, amarra com uma corda, com cipó titica assim, acima do inchaço que ele não soube para cima não. É só isso mesmo.

[00:06:11]

G: Esse cuidado você herdou de algum familiar ou especialista da sua etnia? Esse remédio que a senhora sabe, foi alguém que lhe ensinou ou senhora ouviu alguém falar ou a senhora fez tratamento em alguém com ele?

EE19: É porque era minha mãe que falava pra mim, quando ela era viva, ela falava isso daí, aí eu finquei com aquilo. Aí quando foi um tempo, mordeu um rapaz aí, aí me mandaram me chamar. Mas eu não sou curandeira, não sou pajé, mas eu vou lá. Aí eu foi lá, ensinei remédio, pilei puxei, não é que ficou bom mesmo.

G: Ele voltou andar?

EE19: Voltou andar e pronto. Ficou bom mesmo.

G: Conta como a senhora cuidou dele?

EE19: O rapaz que mora aqui mesmo, o nome dele é (...). Aí cobra mordeu ele, estava para lá, perigo. Deixa-me espiar ainda, aí fui para lá espiar, a (....) disse, ah mana, cobra mordeu meu marido. Aí fui para lá, disse (...) bora fazer um remédio, enquanto a lancha chega. Ai mais que depressa, pega folha disso daí… bem-te-vi-caá…

G: Mas ele é encontrado aqui mesmo esse bem-te-vi-caá? É próximo?

EE19: É dificil dona (...), mas a gente encontra. Ele tipo o bem-te-vi mesmo. Tipo o bem-te-vi, tem uma listra assim, é uma planta, uma planta não, um mato.

G: É encontrado aqui na aldeia, ou vocês tiverem que ir em alguma Igarapé, alguma coisa?

EE19: Encontremos assim, na roça. Na roça que nós encontremos. Aí eu foi para lá, tirei, aí mandei ela pilar, ela pulou, nós puxemos, pronto…. Quando o homem chegou na lancha, ele já estava melhor, ele foi embora para lá tomar injeção, porque não confio mais no remédio caseiro, só já no remédio de farmácia. Aí ele foi embora tomar injeção para lá…

[00:08:11]

G: Você pode me relatar quais cuidados (esse eu já perguntei)… Esses cuidados em qual momento e por quanto tempo? No caso, esse cuidados que você fixou foi logo que ele foi mordido?

EE19: Foi, foi logo que ele foi mordido. Que ele chegou, fizeram aquele alarme, aí nós fomos para lá espiar, disque não presta a gente espiar, mas eu foi lá, fazer uma obra de caridade, aí foi na hora que a (....) disse, não sabe será de remédio (...), mana eu sei aí, tendo fé, o que cura é a fé. Aí nós fizemos, pronto o homem ficou bom, e foi para Laranjal só tomar injeção mesmo. Graças a Deus…

[00:08:49]

G: Você realiza o cuidado de maneira isolada ou associação ao cuidado médico? Esse cuidados que vocês fizeram, foi só entre a senhora, ela, e ele que estava picado da cobra?

EE19: Foi, foi só entre nós mesmo. Ele tava mordido de cobra, nós formos lá…

G: Foi no quarto essas coisas?

EE19: Não. Foi no amplo mesmo.

G: No amplo mesmo. Não foi isolado?

EE19: HumHum… não, não foi isolado não, foi no amplo mesmo. Aí porque ligaram para lancha vir buscar, foi rápido mesmo. Ai ninguém… foi só eu que fui lá, os outros não foram não, só a família dele que estava perto, e só ele mesmo e nós. E pronto! Foi esse remédio, graças a Deus com muita fé, passou mais a dor. O inchaço também não subiu mais, é só…

[00:09:36]

G: Dos cuidados que você realizou, houve alguma melhora no quadro da picada a de cobra?

EE19: HamHam…. Houve graças a Deus. Quando chegou lá no posto, no polo base, falaram o quê que tinham feito, aí ele falou… foi uma lá senhora que fez remédio para ele rápido, porque tava muito botando sangue, muito doendo, ela ensinou esse remédio, foi rapidinho que passou. E foi mesmo dona (...). HumHum… Graça a Deus, ele disse assim. Está aí, já vi uma mulher que sabe, é…. Dona (...).

G: Então, a senhora é curadora?

EE19: Rum… diz a coisa, se faz de besta porque ela quer (sorrindo)

[00:10:17]

G: Dos recursos utilizados nessa prática são encontrados com facilidades? Esse remédio que a senhora usou para fazer o tratamento dele, foi encontrado com facilidade? Vocês tiveram que caminhar, sair daqui de rabeta?

EE19: É por causa, que nós tivemos lá pela roça, andar atrás desse bem-te-vi-caá, esse catauari por aqui, não tem não, só tem no Mucajá. Aí nós ainda tínhamos um pedaço aí, que meu Velho tirou para reumatismo, que é bom também, aí foi disse pra ele, leva, aí eu levei pra lá, foi com remédio parou a dor mais do pé dele. Que era no pé, que ela tinha mordido, e aí parou, puxemos, o inchaço foi pra baixo, pronto, foi para o polo base tomar injeção, ficou bom, pronto… e foi rápido, rápido mesmo.

[00:11:02]

G: Quanto tempo você e pessoa realizou esse cuidado, teve acesso ao recurso utilizado nesta prática? Quanto tempo no caso o remédio que vocês usaram, quando tempo, quantas horas vocês viram que já estava melhorando?

EE19: Assim, parece que duma meia hora para a frente, foi rápido mesmo. Tivemos que correr, que ele estava gemendo, chorando, com tanta dor, aí nós puxemos mais rápido mesmo, mandado por Deus mesmo, na hora foi passando dor, graças a Deus, ainda falou assim, graças a Deus dona (...). Pois é, eu disse... Onde a gente não pensa, é lá que tá. Diz ele, é verdade mesmo, com esse remédio da senhora, eu já posso dizer, que o seu remédio é muito bom mesmo, remédio caseiro, que eu não conheço nem o que é… não conhece não, só quem sabe é eu mesmo, que sei, bem-te-vi-caá, é o remédio de picada de cobra também.

G: Qual foi a cobra que tinha picado ele?

EE19: Agora aí que eu não sei, dona (...), até me esqueci. Não sei mesmo.

G: A senhora não lembra?

EE19: Não lembro não.

[00:12:06]

G: Entre este recurso, você já presenciou, utilizou algo da própria serpente, para realização desse cuidado? No caso, no caso a banha (exemplo)

EE19: É, a banha dela também é remédio. A gente mata… a banha dela, tira, passa em cima. De qualquer bicho… se for uma tucandeira que ferra, pode matar ela, tirar o bucho e colocar em cima da Cisura, da mordida, da ferrada, que passa na hora. A aranha do mesmo jeito, se a aranha morder a pessoa, se estiver doendo, pode partir, tirar o bucho, botar em cima da mordida, dizem que hora que passa, passa mesmo, pois é, isso daí.

G: Então, dona (...) , muito obrigado, aqui… estivemos aqui com (...), é especialista na área da etnia Munduruku, como ela falou, há cinco anos ela também pega desmetidura, ela benze, entendeu. Ela não tem muito conhecimento na parte de curador, mas sempre ela faz alguns remédios caseiros.
