# Supplementary material for: Snakes and snakebites in the munduruku cosmology and medicine, central Brazilian Amazonia
Source: PLoS Negl Trop Dis. 2026 Jun 22;20(6):e0014462. doi: 10.1371/journal.pntd.0014462 (PMC13309027; doi:10.1371/journal.pntd.0014462)
Supplement: Attachment — Submitted filename: Response letter.pdf [file pntd.0014462.s004.pdf]

Dear Editor,

We appreciate the opportunity to respond to the reviewers' comments, which have helped improve the quality of our manuscript. We also thank you for the positive feedback on the work under review. In this revised version, we have addressed the suggestions and incorporated the corrections requested by all three reviewers, as detailed below.

Methods

Reviewer #1: See comments below.
Response: Done.

Reviewer #2: The study employs a qualitative, ethnographic methodology, which is appropriate given its focus on cosmology, healing practices, and culturally embedded interpretations of snakebites. This methodological orientation is well suited to the research questions, particularly in fields like Medical Anthropology and Ethnobiology, where understanding meaning, belief systems, and local epistemologies is central.
Response: Thanks for the positive comment.

Reviewer #3: - The authors interviewed traditional Munduruku healers. No hypothesis was tested. It is unclear how these individuals were identified and selected and to what extent this sample might be representative. A relatively high proportion of healers were midwives; it would be helpful to clarify whether this reflects the typical composition of healers in this context.
Response: We thank the reviewer for these important observations.

This study follows a qualitative exploratory design aimed at developing an explanatory model of snakebites within Munduruku cosmology and medicine. It is intended to generate in-depth, contextually grounded understanding of meanings, practices, and interpretations. We have clarified the study aim.

Regarding participant identification and selection, Munduruku caregivers were identified through collaboration with local health and education professionals working in the Indigenous territories, who are familiar with community-recognized healing specialists. This approach allowed us to access socially legitimized caregivers, in line with ethnographic and qualitative research practices focusing on the folk healthcare sector. We have expanded the description of this process in the Methods section to improve clarity. The sample size was achieved by saturation and composition were considered sufficient to achieve thematic saturation, as no substantially new themes emerged in the final interviews. This has now been clarified in the revised manuscript.

Finally, we appreciate the reviewer's observation regarding the relatively high proportion of midwives. In the studied context, midwives often play multiple roles within the Munduruku healthcare system, extending beyond childbirth to broader therapeutic practices, including the management of snakebites. Therefore, we believe that their prominence in our sample reflects their central and multifunctional role in the local

healing system, rather than a sampling imbalance. We have clarified this point in the Results and Discussion sections.

- Numbers provided for the village origins of the 19 participants do not add up.
Response: The numbers have been corrected accordingly.

- How was the interview script developed and why was this set of questions chosen?
Response: We thank the reviewer for this important question.

The script was developed by the research team, which has extensive experience in qualitative research and in the cultural and clinical aspects of snakebite envenoming in the Amazon. Care was taken to ensure that questions were open-ended, culturally sensitive, and flexible enough to allow participants to elaborate on their experiences and perspectives. The interview script was developed to align with the study's objective of constructing an explanatory model of snakebites within Munduruku cosmology and medicine. The questions were structured to explore key domains relevant to this framework, including participants' identities and roles, cultural meanings attributed to snakes, perceptions of snakebite causality, illness progression, and therapeutic practices.

We have clarified the development and rationale of the interview script in the Methods section of the revised manuscript.

- Some questions are not phrased neutral and open-ended but in a way that may introduce bias by implying desired responses. In particular, the question 'Did the care you provided result in any improvement in the snakebite?' presumes improvement and may create pressure on the respondent as it could be perceived as reflecting on their practice or expertise. This bias should be considered when reporting and discussing the responses.
Response: We thank the reviewer for this thoughtful and important observation.

We acknowledge that some questions in the interview script, including the one highlighted by the reviewer, may have been phrased in a way that could suggest an expectation of positive outcomes. However, we highlight that the interviews were conducted using a semi-structured and flexible approach, allowing participants to elaborate freely on their experiences, including situations of uncertainty, limitations, or lack of effectiveness of treatments.

The interviewer encouraged open-ended narratives and did not restrict responses to predefined assumptions, which helped mitigate potential bias introduced by specific question wording. In addition, participants often contextualized their responses within broader explanatory frameworks, rather than providing simple affirmative answers.

We agree that the possibility of response bias should be acknowledged. Therefore, we have included a statement in the Discussion section addressing this as a limitation, particularly considering the potential influence of social desirability and the positioning

of participants as recognized community experts. We appreciate this comment and believe it has strengthened the critical reflection in our manuscript.

We added this paragraph in the Discussion section:

'The semi-structured script format allowed flexibility for participants to expand their responses beyond the guiding questions, minimizing the potential influence of question framing. However, some questions in the interview script may have been phrased in a way that could suggest expected responses, particularly regarding the perceived effectiveness of treatments. Although interviews were conducted in a flexible and open-ended manner, allowing participants to elaborate freely, the possibility of response bias, especially social desirability bias, cannot be excluded, given the participants' roles as recognized healers within their communities.'

Results

Reviewer #1: See comments below.
Response: Done.

Reviewer #2: This study demonstrates that Munduruku responses to snakebites cannot be understood through a simple framework of "traditional versus biomedical" medicine. Instead, what emerges is a dynamic and adaptive medical system, in which practices from Indigenous healing, religious influences, and biomedicine are continuously incorporated, tested, and reinterpreted in pursuit of efficacy.
Response: This is correct. Thanks for the comment.

Ultimately, the findings highlight that improving snakebite care in the Amazon depends on moving beyond hierarchical models of healthcare. Instead, it requires symmetrical collaboration between biomedical practitioners and Indigenous healers, who act as essential mediators between human, animal, and spiritual domains. Recognizing and supporting this intercultural interface is key to developing healthcare systems that are both clinically effective and culturally meaningful.
Response: This is correct. Thanks for the comment.

Reviewer #3: A key results is that 'A snakebite is not seen merely as a natural phenomenon, but as an experience involving natural and supernatural forces, requiring care that combines material practices and rituals.' Building on this, the question arises as to how medically appropriate treatment can be implemented based on such a perception. The current manuscript touches on this only briefly.
Response: We thank the reviewer for this insightful comment. We agree that further elaboration on the practical implications of this finding is essential.

In this context, medically appropriate treatment, particularly the timely administration of antivenom, cannot be effectively implemented without engaging with these explanatory models. Rather than replacing Indigenous interpretations, successful

strategies should aim at building culturally sensitive and dialogical approaches that allow biomedical interventions to be incorporated into existing therapeutic itineraries. Our findings suggest that Munduruku healers already play a key role as mediators between different healthcare systems, recognizing situations that require referral to biomedical services while maintaining their role in the healing process. This creates an opportunity for developing collaborative models of care in which Indigenous healers and biomedical professionals work in partnership.

We have expanded the Discussion section to more explicitly address these implications, emphasizing the importance of intercultural health strategies, the inclusion of Indigenous healers in health planning, and the adaptation of healthcare delivery, such as decentralization of antivenom, to local sociocultural contexts.

We appreciate this comment and believe that it has strengthened the translational relevance of our study.

- Please double check numbers provided in subsection 'Participans' identities' as some seem not to add up, spelling checks and rounding may be reasonable in view of the small sample ("52.6b%").

Response: Corrections were made accordingly.

- Check gender-specific pronouns in table 3.

Response: We have carefully revised Table 3 to ensure consistency and accuracy in the use of gender-specific pronouns for all participants. All identified inconsistencies have been corrected in the revised manuscript.

In line 488, figures not figs.

Response: Correction was made accordingly.

- Some aspects of interest are not covered but maybe could be integrated. For example, how much experience the respondents have roughly with snakebites (one case every few years, weekly cases?), what their knowledge of species is (e.g., are bites from non-venomous species treated "successfully"?), is there is a basic understanding of snake venoms and their effects.

Response: We thank the reviewer for these comments. We agree that aspects such as the frequency of snakebite cases managed by the participants, their detailed knowledge of snake species, and their understanding of venom effects are highly relevant. However, the primary aim of this study was not to quantify experience or assess biomedical knowledge, but to explore explanatory models of snakebites within Munduruku cosmology and medicine. However, as all data presented in this study are based on participants' responses during in-depth interviews, it was not possible to quantify experience or assess biomedical knowledge from participants.

Nevertheless, elements related to these dimensions are indirectly reflected in the findings. Participants demonstrate experiential knowledge of snakebite severity, often classifying snakes retrospectively based on clinical progression, and report empirical validation of treatments, including observations from human and animal cases. We

present these points in the Results (please see sections 'Snakes and snakebites' and 'Course of sickness') section of the manuscript.

- While participant 16 suggests that hospital treatment might be the standard, would all participants agree that SBE patients should be transferred to a hospital if possible? Could this differ depending on the snake species involved?
Response: We thank the reviewer for this important point.
Our findings indicate that most participants recognized the importance of hospital-based treatment, particularly for severe cases, and were not opposed to referral to biomedical services. In this sense, there is a strong tendency among Munduruku healers to recommend hospital care when necessary, especially in situations perceived as severe or beyond their scope of action. At the same time, this does not imply a complete replacement of Indigenous care. Healers maintain an active and complementary role, providing treatment before referral, and after the patient's return to the village. We added this sentence in Results:
'Most participants recognized the importance of hospital-based treatment, particularly for severe cases, and did not express resistance to referral to biomedical services.'

- Figure 3 would benefit from clearer conceptual structuring and presentation. Currently, it is not clear how and in which order different aspects are connected.
Response: We have revised Figure 3 to improve its conceptual clarity and visual organization. The relationships between elements are now more clearly structured, with a more explicit flow indicating how different factors interact in the course of snakebite illness according to Munduruku medicine. In addition, we have refined the figure legend to better explain the sequence and connections between components. We believe that these changes enhance the readability and interpretability of the figure.

- The provided images were created how? Are these actual paintings by the authors or have they been created using AI tools?
Response: The images presented in the manuscript are original ethnographic illustrations created by one of the co-authors (UGO), an Indigenous artist from the study area. These illustrations were produced manually as paintings, based on narratives collected during the interviews, and aim to visually represent key elements of Munduruku cosmology and therapeutic practices. We have clarified this information in the Methods section of the revised manuscript.No artificial intelligence tools were used in the creation of these images.

Conclusions

Reviewer #1: See comments below.
Response: Done.

Reviewer #2: The absence of resistance to biomedical interventions,particularly antivenom, reveals that Munduruku medical practice is not oppositional but integrative. The coexistence of these systems underscores that effective treatment is not defined by

adherence to a single paradigm, but by the ability to navigate multiple knowledge systems simultaneously.
Response: Thanks for the comment.

Reviewer #3: - The aspect of SBE as a punishment for not following social norms or rules of coexistence is interesting and reads as a broader and more positive interpretation of 'compliance with dietary and sexual interdictions'. The results section suggests that this interpretation is particularly pronounced for women (e.g. in the context of pregnancy or menstruation). It would be helpful to clarify whether this reflects a gender difference or could also be reflective of the strong representation of midwives in the sample.
Response: We agree that the association between snakebites and the transgression of social norms, particularly in relation to pregnancy and menstruation, emerges prominently in the narratives. However, based on our data, we do not interpret this as solely a result of the higher representation of midwives in the sample. These themes were also consistently reported by male participants, suggesting that such interpretations are more broadly shared within the Munduruku cosmology rather than being restricted to female perspectives or to specific caregiver roles. In addition, previous ethnographic studies among Amazonian Indigenous groups have described similar associations between bodily states, social norms, and vulnerability to snakebites, reported by both men and women.

- The presentation of traditional treatment methods and their supposed effectiveness appears unbalanced. Suggesting any potential effectiveness of topical application of snake parts is highly speculative. On the other hand, unsuccessful treatments and possible complications from these applications are not addressed currently.
Response: We thank the reviewer for this important and thoughtful comment.

We agree that the presentation of traditional treatment practices requires careful balance to avoid unintended implications regarding clinical effectiveness. Our intention was not to validate the biomedical efficacy of these practices, but rather to document how they are perceived, used, and interpreted by Munduruku healers.

As observed in our data, topical preparations, particularly those involving animal and plant products, predominated among the therapeutic resources described by participants. This had already been observed among the Wari, as we discussed in this article (Conklin BA. O sistema médico Warí (Pakaanóva). In: Santos RV, Coimbra Jr. CA. Saúde e Povos Indígenas. Rio de Janeiro: Ed. FIOCRUZ, 1994, p. 161-186).

Regarding effectiveness, the predominantly positive accounts reported in the manuscript reflect the participants' own narratives and interpretations. Within Indigenous medical systems, the evaluation of efficacy often involves symbolic, experiential, and relational dimensions, which differ from biomedical criteria. This perspective has been previously explored by our group in the context of symbolic efficacy (please see the section intitled '(Symbolical) Efficacy of the SBE treatment prescribed by the indigenous caregivers' in the Discussion of de Farias AS, do Nascimento EF, Gomes Filho MR, Felix AC, da Costa Arévalo M, Adrião AAX, Wen FH, de Carvalho FG, Murta F, Machado VA, Sachett J, Monteiro WM. Building an explanatory

model for snakebite envenoming care in the Brazilian Amazon from the indigenous caregivers' perspective. PLoS Negl Trop Dis. 2023 Mar 10;17(3):e0011172).

At the same time, we acknowledge that the effectiveness of these practices cannot be established within the scope of this study, and that some passages may have conveyed an overly speculative interpretation.

- Traditional interventions can cause delays and serious harm. This contributes to morbidity and mortality and has been documented, for example, in several countries in Africa. Some of the traditional remedies described in the manuscript would appear to come with the risk of serious infection, which should be stated and could be discussed in view of literature on harmful treatments complicating SBE.

Response: The efficacy of the indigenous medicine is an interesting and complex question. In this work, as much as possible, we avoided classifying the procedures indicated by indigenous caregivers as effective, non-effective or deleterious or judging them in any other way. We believe that it is not up to researchers, at the risk of a colonialist attitude, to make judgments about ancient practices, especially those of a mythical-religious nature. Except for proven deleterious procedures, such as the use of tourniquets, the other practices still lack methodological resources to confirm or refute any benefit to the patient. In addition, we tried our best to respect the role that the caregivers play in their village, including the way they see themselves and the way they prefer to be addressed.

- No references provided in lines 671 and 677 despite placeholder "(ref)".

Response: The correction was made accordingly.

- A language edit and more concise writing would improve clarity of the section.

Response: Text was edited and revised accordingly.

- Please clarify what 'this translation' refers to in line 788.

Response: The correction was made accordingly. Sentence was re-written.

Summary and General Comments

Reviewer #1: This manuscript explores the cultural, cosmological, and therapeutic understanding of snakebites among the Munduruku people in the Brazilian Amazon through qualitative interviews with Indigenous healers. The study develops an explanatory model integrating spiritual, social, and biological dimensions of envenomation. It highlights the role of traditional medicine, beliefs, and healing practices in snakebite management. The work emphasizes the importance of integrating Indigenous and biomedical approaches to improve culturally appropriate care. The authors should be congratulated for addressing an underexplored and highly relevant aspect of snakebite envenoming, particularly in Indigenous populations that are disproportionately affected. Despite, this I have some suggestions I hope help to improve this nice study.

Response: Thanks for the comment and suggestions.

Major issues

1. The study includes 19 participants, which is acceptable for qualitative research, but the representativeness across the broader Munduruku population and potential regional variability is not sufficiently discussed.

Response: We thank the reviewer for this important comment. We agree that, as a qualitative study, our aim was not statistical representativeness, but rather to develop an in-depth, contextually grounded explanatory model based on the perspectives of socially recognized caregivers.

To address this concern, we have expanded the Discussion section to clarify the scope and limitations of our findings. We now explicitly acknowledge that our sample, although diverse in terms of therapeutic roles and including participants from different villages within the study area, does not capture the full heterogeneity of the Munduruku population across different territories. We also highlight that regional, historical, and intercultural dynamics may shape variations in cosmology and healthcare practices among different Munduruku groups.

Furthermore, we emphasize that our findings should be interpreted as analytically transferable rather than statistically generalizable, contributing to the understanding of Indigenous explanatory models of snakebite and informing intercultural health strategies, rather than representing all Munduruku communities.

The corresponding changes have been incorporated into the Discussion section (last paragraph):

'This study has limitations that should be acknowledged. Although the sample included diverse caregivers from different villages, it does not capture the full heterogeneity of the Munduruku population across territories. Regional and sociocultural differences may influence cosmological interpretations and therapeutic practices. Therefore, findings are not statistically generalizable but analytically transferable, offering a contextually grounded model to inform intercultural health strategies.'

2. Although reflexivity is mentioned, the influence of researcher background and interpretation on data analysis should be more critically examined, particularly given the cultural sensitivity of the topic.

Response: We thank the reviewer for this insightful comment. We agree that reflexivity is particularly important given the cultural sensitivity of the topic. The first author's Indigenous ancestry and academic background in Indigenous health and Amazonian health history positioned her in close proximity to the research context, which may have influenced data interpretation.

To address this, we have expanded the reflexivity section to more critically acknowledge this positionality. Throughout the study, a reflexive and methodologically rigorous approach was adopted to ensure that interpretations remained grounded in participants' narratives, experiences, and meanings. In addition, analyst triangulation

and regular team discussions were employed to minimize individual interpretative bias and enhance analytical consistency.
These clarifications have been incorporated into the Methods section (section named *Study team and reflexivity*).

'*Given the cultural sensitivity of the topic, reflexivity was considered throughout the research process. The first author's Indigenous ancestry and academic background positioned her close to the study context, which may have influenced interpretation. To address this, a rigorous reflexive approach ensured interpretations remained grounded in participants' narratives. Analyst triangulation and team discussions were used to minimize bias and strengthen consistency.*'

3. The study relies entirely on self-reported narratives. Integration with clinical data, reported snakebite cases, or health system outcomes would strengthen the translational relevance.
Response: We thank the reviewer for this important suggestion. We acknowledge that the study is based on self-reported narratives and does not include clinical or health system data, as access to such data at the study sites was not available.
However, the aim of this study was to develop an in-depth explanatory model of snakebites from the perspective of Munduruku caregivers, focusing on meanings, practices, and therapeutic itineraries. Within this qualitative framework, participants' narratives constitute the primary source of knowledge.
We agree that integrating qualitative findings with clinical and epidemiological data would enhance translational relevance, and we highlight this as an important direction for future research.

4. Some conclusions regarding healthcare integration and system design extend beyond the presented qualitative data.
Response: We thank the reviewer for this important observation. We agree that some statements in the Conclusion could be interpreted as extending beyond the empirical scope of the qualitative data. To address this, we have revised the Conclusion to more clearly anchor our statements in the study findings and to frame implications for healthcare integration as interpretative insights rather than prescriptive recommendations. The revised text emphasizes that our conclusions are grounded in participants' perspectives, particularly their openness to combining Indigenous and biomedical care, while avoiding normative generalizations about health system design.

These revisions have been incorporated into the Conclusion section.

5. The origin of the images created by the authors should be clarified. Some of the figures appear to be AI-generated or digitally illustrated, but no information is provided on how they were produced.
Response: The images presented in the manuscript are original ethnographic illustrations created by one of the co-authors (UGO), an Indigenous artist from the study area. These illustrations were produced manually as paintings, based on narratives collected during the interviews, and aim to visually represent key elements of Munduruku cosmology and therapeutic practices. We have clarified this information in

the Methods section of the revised manuscript.No artificial intelligence tools were used in the creation of these images.

Minor issues
1. Terms such as "snakebite," "envenomation," and "accident" are used interchangeably and should be standardized.
Response: We thank the reviewer for this observation. We have revised the manuscript to standardize terminology throughout the text. The term "snakebite envenoming" is now used when referring to cases involving venom injection, while "snakebite" is used more generally when appropriate. The term '*accident*' has been removed or replaced to ensure consistency with current scientific usage.

These changes have been applied throughout the manuscript.

2. Ethnographic figures are informative but would benefit from clearer legends and stronger linkage to the main text.
Response: We thank the reviewer for this helpful suggestion. We have revised the figure legends to provide clearer and more detailed descriptions of the ethnographic content. In addition, we strengthened the linkage between the figures and the main text by explicitly referring to them at relevant points in the Results section and clarifying their analytical contribution.

3. The description of qualitative analysis (codebook development, consensus process) could be slightly expanded for reproducibility.
Response: We thank the reviewer for this suggestion. We have expanded the description of the qualitative analysis to provide greater detail on the codebook development and consensus process. Specifically, we clarified how independent coding was conducted, how discrepancies were discussed and resolved, and how the final coding framework was established.
These additions have been incorporated into the Data Analysis section:
'Discrepancies between coders were systematically discussed and resolved through consensus meetings, with iterative refinement of the coding framework to ensure coherence, transparency, and analytical consistency across transcripts.'

4.
This is a valuable and original contribution that broadens the understanding of snakebite envenoming beyond biomedical paradigms. I was lucky to review this manuscript. I learned a lot.
Response: We sincerely thank the reviewer for this generous and encouraging feedback. We are pleased that the manuscript contributed to broadening perspectives on snakebite envenoming and appreciate the reviewer's thoughtful engagement with our work.

Reviewer #2: This manuscript presents an ethnographically grounded exploration of the cultural, cosmological, and medical meanings of snakes and snakebites among the Munduruku people. It addresses an important intersection between medical anthropology, ethnobiology, and public health, particularly in a region where snakebite

envenomation remains a major yet understudied issue. The paper is timely, relevant, and conceptually valuable, especially given increasing attention to Indigenous knowledge systems in healthcare.
Response: We sincerely thank the reviewer for this thoughtful and encouraging assessment of our work. We appreciate the recognition of the manuscript's relevance and its contribution to integrating perspectives from medical anthropology and public health.

Reviewer #3: Title: Snakes and Snakebites in Munduruku Cosmology and Traditional Medicine in the Central Brazilian Amazon

The authors Dias et al. address an important problem: how to close the access gap to medical treatment for snakebite envenoming (SBE) in remote locations without adequate healthcare infrastructure. To allow for a decentralized antivenom treatment in partnership with traditional medicine, the authors investigated how snakebite is understood and managed by an Indigenous group in the Brazilian Amazon, the Munduruku people, as a basis to inform effective and culturally appropriate care. For this, the authors interviewed traditional Munduruku healers. The study is relevant and involves complexity in implementation and interpretation. However, the current manuscript form raises questions and is not always easy to follow. It would benefit from a clearer and more concise presentation of the topic, the findings and implications.
Response: We thank the reviewer for this thoughtful and constructive assessment. We appreciate the recognition of the relevance and complexity of the study.
In response to this comment, we have revised the manuscript to improve clarity, coherence, and conciseness. Specifically, we refined the presentation of the study objectives, streamlined sections of the Results and Discussion, and clarified the articulation between findings and their implications. We also reduced redundancies and improved the overall structure to enhance readability.
These revisions have been implemented throughout the manuscript.

Introduction, also applicable to Results and Discussion
- Overall, the manuscript would benefit from linguistic revision, and in several places the text could be more concise and coherent. Avoid repetitions. Some sections are presented in considerable detail, but their connection to the overall argument is not always clear. Strengthening the coherence and explicitly linking these points to the main narrative would improve readability. For example, it is not obvious what common crops and trade has to do with the aim of the study and envenoming by Bothrops atrox is described over eleven lines in the introduction but this point is not addressed again later.
Response: We thank the reviewer for this detailed and constructive feedback. We agree that improvements in clarity, conciseness, and coherence were needed.
In response, we have carefully revised the manuscript to reduce redundancies, improve linguistic clarity, and strengthen the connection between different sections and the overall argument. We streamlined descriptive passages that were not directly linked to the study objectives, including the section on subsistence practices, and revised the introduction to ensure that epidemiological and clinical information, such as the

description of *Bothrops atrox* envenoming, are more clearly connected to the study rationale and discussed more consistently throughout the text.
These revisions were applied throughout the manuscript to enhance readability and narrative coherence.

- Line 125 and 176: 17,997 individuals and 4,171; are these numbers actually known so precisely? Otherwise, rounded figures would be more appropriate.
Response: We thank the reviewer for this observation. We agree that rounded figures improve readability and are more appropriate in this context. We have revised the manuscript to present these values as approximate population estimates.

Line 132: A more neutral phrasing could be considered such as 'detailed' rather than 'excellent' review.
Response: We thank the reviewer for this suggestion. We agree that a more neutral tone is appropriate, and we have revised the wording accordingly.
