# Supplementary material for: Snakes and snakebites in the munduruku cosmology and medicine, central Brazilian Amazonia
Source: PLoS Negl Trop Dis. 2026 Jun 22;20(6):e0014462. doi: 10.1371/journal.pntd.0014462 (PMC13309027; doi:10.1371/journal.pntd.0014462)
Supplement: Attachment — Submitted filename: Response letter_R2.docx [file pntd.0014462.s005.docx]

Dear Editor,

We appreciate the opportunity to respond to the reviewers’ comments, which have helped improve the quality of our manuscript. We also thank you for the positive feedback on the work under review. In this revised version, we have addressed the suggestions and incorporated the corrections requested by all three reviewers, as detailed below.

**Summary and General Comments**

Reviewer #1: The authors have replied all major comments and issues. I have no further suggestions. Congratulations! This is a nice piece of work.

Response: We thank the reviewer for this very positive comment.

Reviewer #3: Title: Snakes and Snakebites in Munduruku Cosmology and Traditional Medicine in the Central Brazilian Amazon
The authors have addressed most previous concerns and suggestions. They submitted a revised manuscript, more clearly and coherently written with additional clarifications in the methods and result sections, including figure 3. Limitations of the study, in particular the possibility of response bias / social desirability bias, are described. While some questions remain (e.g., how much experience with snakebites have healers), it as acknowledged that this is beyond the scope of this study.

Response: We thank the reviewer for these positive comments.

One aspect that, in my view, could still be addressed more appropriately is the presentation and discussion of traditional treatments. In its current form, it may be understood as suggesting some form of effectiveness and the citation of studies reporting in vitro inhibitory potential reinforces this impression: *>Although their efficacy cannot be confirmed, some biological evidence may partially support these practices: snakes possess endogenous toxin inhibitors, such as metalloproteinase and phospholipase inhibitors [67], and several plant metabolites, including those cited by Munduruku healers, have shown in vitro inhibitory potential [68,69].* <
However, such studies come with considerable limitations (e.g., long pre-incubation periods; non-physiological concentrations, specific vs. broad enzyme inhibitory effects) and do not appear to adequately support claims that traditional treatments might be effective in clinical practice. The authors argue that judgments about traditional practices, especially those of a mythical-religious nature, are highly sensitive and should be avoided. However, in its current form the manuscripts could be perceived as presenting such approaches in a rather positive light. Thus, rephrasing this part is recommended to avoid implying efficacy. In addition, the authors should consider to reference literature that documented harmful consequences of traditional SBE treatments in other parts of the world, to introduce the concept that such methods can be detrimental, without directly judging the methods described by Munduruku healers in this study.

Response: We thank the reviewer for this thoughtful comment.

We agree that evidence from in vitro studies should not be interpreted as proof of clinical efficacy and that discussing potential biological mechanisms may inadvertently suggest validation of traditional treatments for snakebite. Our intention was not to endorse or recommend these practices, but rather to acknowledge that some remedies reported by participants have been investigated experimentally. To avoid any implication of clinical effectiveness, we revised the text to emphasize the preliminary nature of these findings and the lack of evidence supporting their use in clinical management of snakebite envenoming. The revised wording now clearly distinguishes experimental observations from clinically validated interventions.

*“Although some medicines reported by participants have been investigated experimentally, their efficacy and safety in snakebite treatment remain unproven. Snakes possess endogenous toxin inhibitors, such as metalloproteinase and phospholipase inhibitors [67], and some plant metabolites have demonstrated inhibitory activity against venom components under laboratory conditions [68,69]. However, these findings are largely derived from experimental studies and should not be interpreted as evidence supporting the clinical use of traditional treatments for snakebite envenoming.”*
