# Supplementary material for: Snakes and snakebites in the munduruku cosmology and medicine, central Brazilian Amazonia
Source: PLoS Negl Trop Dis. 2026 Jun 22;20(6):e0014462. doi: 10.1371/journal.pntd.0014462 (PMC13309027; doi:10.1371/journal.pntd.0014462)
Supplement: Peer review history

# Peer review: Decision Letter 0

**DOI:** 10.1371/journal.pntd.0014462.r001

**Authors:** José Gutiérrez, Emeka Dingwoke

6 May 2026

PNTD-D-26-00565

Snakes and Snakebites in Munduruku Cosmology and Traditional Medicine in the Central Brazilian Amazon

PLOS Neglected Tropical Diseases

Dear Dr. Monteiro,

Thank you for submitting your manuscript to PLOS Neglected Tropical Diseases. After careful consideration, we feel that it has merit but does not fully meet PLOS Neglected Tropical Diseases's publication criteria as it currently stands. Therefore, we invite you to submit a revised version of the manuscript that addresses the points raised during the review process.

Please submit your revised manuscript within by 5th June, 2026. If you will need more time than this to complete your revisions, please reply to this message or contact the journal office at plosntds@plos.org. When you're ready to submit your revision, log on to https://www.editorialmanager.com/pntd/ and select the 'Submissions Needing Revision' folder to locate your manuscript file.

Please include the following items when submitting your revised manuscript:

* A letter that responds to each point raised by the editor and reviewer(s). You should upload this letter as a separate file labeled 'Response to Reviewers'. This file does not need to include responses to any formatting updates and technical items listed in the 'Journal Requirements' section below.

* A marked-up copy of your manuscript that highlights changes made to the original version. You should upload this as a separate file labeled 'Revised Manuscript with Track Changes'.

* An unmarked version of your revised paper without tracked changes. You should upload this as a separate file labeled 'Manuscript'.

If you would like to make changes to your financial disclosure, competing interests statement, or data availability statement, please make these updates within the submission form at the time of resubmission. Guidelines for resubmitting your figure files are available below the reviewer comments at the end of this letter.

As the corresponding author, your ORCID iD is verified in the submission system and will appear in the published article. PLOS supports the use of ORCID, and we encourage all coauthors to register for an ORCID iD and use it as well. Please encourage your coauthors to verify their ORCID iD within the submission system before final acceptance, as unverified ORCID iDs will not appear in the published article. Only the individual author can complete the verification step; PLOS staff cannot verify ORCID iDs on behalf of authors.

We look forward to receiving your revised manuscript.

Kind regards,

Emeka John Dingwoke, Ph.D

Academic Editor

PLOS Neglected Tropical Diseases

José María Gutiérrez

Section Editor

PLOS Neglected Tropical Diseases

Shaden Kamhawi

co-Editor-in-Chief

PLOS Neglected Tropical Diseases

orcid.org/0000-0003-4304-636XX

Paul Brindley

co-Editor-in-Chief

PLOS Neglected Tropical Diseases

orcid.org/0000-0003-1765-0002

**Additional Editor Comments:**

Dear Dr. Monteiro,

Thank you for submitting your manuscript entitled “Snakes and Snakebites in Munduruku Cosmology and Traditional Medicine in the Central Brazilian Amazon” to PLOS Neglected Tropical Diseases.

The manuscript addresses an important and underexplored dimension of snakebite envenoming, particularly within Indigenous populations. The integration of cultural, cosmological, and therapeutic perspectives represents a valuable contribution and aligns well with the journal’s scope.

After evaluation of the manuscript, you will see that the reviewers raised several important concerns that need to be addressed to ensure the manuscript meets the journal’s standards for scientific rigor, clarity, and appropriate interpretation of findings. These include the need to clarify participant selection and representativeness, expand the discussion of reflexivity and potential researcher influence, and provide better transparency in the qualitative analysis process. In addition, the conclusions should be more carefully aligned with the qualitative nature of the data, and the discussion should present a more balanced view of traditional treatment practices, including potential risks. Clarification is also required regarding the origin and presentation of figures and images, and all numerical inconsistencies and missing references should be corrected.

Several minor issues are also identified that should be addressed to improve clarity and presentation. These include standardizing terminology, improving language and concision, ensuring consistency in formatting, strengthening figure legends and their linkage to the text, and clarifying ambiguous statements where necessary.

The manuscript has clear potential, and a revised version addressing these points will be considered further.

**Journal Requirements:**

1) Please ensure that the CRediT author contributions listed for every co-author are completed accurately and in full.

At this stage, the following Authors/Authors require contributions: Gisele Reis Dias, Gilza Reis Dias Lavareda, Talyson Aparício Gomes, Gilce Reis Dias Silva, Geelle Sena Melo, Mateus Chagas Almeida, Maurício Lopes Silva, Uziel Guaynê Oliveira, Fan Hui Wen, Jacqueline Sachett, Altair Seabra Farias, Felipe Murta, Vinícius Azevedo Machado, and Wuelton Monteiro. Please ensure that the full contributions of each author are acknowledged in the "Add/Edit/Remove Authors" section of our submission form.

The list of CRediT author contributions may be found here: https://journals.plos.org/plosntds/s/authorship#loc-author-contributions

2) Figure 1: please (a) provide a direct link to the base layer of the map (i.e., the country or region border shape) and ensure this is also included in the figure legend; and (b) provide a link to the terms of use / license information for the base layer image or shapefile. We cannot publish proprietary or copyrighted maps (e.g. Google Maps, Mapquest) and the terms of use for your map base layer must be compatible with our CC BY 4.0 license.

Note: if you created the map in a software program like R or ArcGIS, please locate and indicate the source of the basemap shapefile onto which data has been plotted.

If your map was obtained from a copyrighted source please amend the figure so that the base map used is from an openly available source. Alternatively, please provide explicit written permission from the copyright holder granting you the right to publish the material under our CC BY 4.0 license.

If you are unsure whether you can use a map or not, please do reach out and we will be able to help you. The following websites are good examples of where you can source open access or public domain maps:

* U.S. Geological Survey (USGS) - All maps are in the public domain. (http://www.usgs.gov)

* PlaniGlobe - All maps are published under a Creative Commons license so please cite “PlaniGlobe, http://www.planiglobe.com, CC BY 2.0” in the image credit after the caption. (http://www.planiglobe.com/?lang=enl)

* Natural Earth - All maps are public domain. (http://www.naturalearthdata.com/about/terms-of-use/)

3) Figures 2, 3, and 5: Please confirm (a) that you are the photographer; or (b) provide written permission from the photographer to publish the photo(s) under our CC BY 4.0 license.

**Reviewers' Comments:**

Reviewer's Responses to Questions

**Key Review Criteria Required for Acceptance?**

As you describe the new analyses required for acceptance, please consider the following:

**Methods**

-Are the objectives of the study clearly articulated with a clear testable hypothesis stated?

-Is the study design appropriate to address the stated objectives?

-Is the population clearly described and appropriate for the hypothesis being tested?

-Is the sample size sufficient to ensure adequate power to address the hypothesis being tested?

-Were correct statistical analysis used to support conclusions?

-Are there concerns about ethical or regulatory requirements being met?

Reviewer #1: See comments below.

Reviewer #2: The study employs a qualitative, ethnographic methodology, which is appropriate given its focus on cosmology, healing practices, and culturally embedded interpretations of snakebites. This methodological orientation is well suited to the research questions, particularly in fields like Medical Anthropology and Ethnobiology, where understanding meaning, belief systems, and local epistemologies is central.

Reviewer #3: - The authors interviewed traditional Munduruku healers. No hypothesis was tested. It is unclear how these individuals were identified and selected and to what extent this sample might be representative. A relatively high proportion of healers were midwives; it would be helpful to clarify whether this reflects the typical composition of healers in this context.

- Numbers provided for the village origins of the 19 participants do not add up.

- How was the interview script developed and why was this set of questions chosen?

- Some questions are not phrased neutral and open-ended but in a way that may introduce bias by implying desired responses. In particular, the question ‘Did the care you provided result in any improvement in the snakebite?’ presumes improvement and may create pressure on the respondent as it could be perceived as reflecting on their practice or expertise. This bias should be considered when reporting and discussing the responses.

**********

**Results**

-Does the analysis presented match the analysis plan?

-Are the results clearly and completely presented?

-Are the figures (Tables, Images) of sufficient quality for clarity?

Reviewer #1: See comments below.

Reviewer #2: This study demonstrates that Munduruku responses to snakebites cannot be understood through a simple framework of “traditional versus biomedical” medicine. Instead, what emerges is a dynamic and adaptive medical system, in which practices from Indigenous healing, religious influences, and biomedicine are continuously incorporated, tested, and reinterpreted in pursuit of efficacy.

Ultimately, the findings highlight that improving snakebite care in the Amazon depends on moving beyond hierarchical models of healthcare. Instead, it requires symmetrical collaboration between biomedical practitioners and Indigenous healers, who act as essential mediators between human, animal, and spiritual domains. Recognizing and supporting this intercultural interface is key to developing healthcare systems that are both clinically effective and culturally meaningful.

Reviewer #3: A key results is that ‘A snakebite is not seen merely as a natural phenomenon, but as an experience involving natural and supernatural forces, requiring care that combines material practices and rituals.’ Building on this, the question arises as to how medically appropriate treatment can be implemented based on such a perception. The current manuscript touches on this only briefly.

- Please double check numbers provided in subsection ‘Participans’ identities’ as some seem not to add up, spelling checks and rounding may be reasonable in view of the small sample (“52.6b%”).

- Check gender-specific pronouns in table 3. In line 488, figures not figs.

- Some aspects of interest are not covered but maybe could be integrated. For example, how much experience the respondents have roughly with snakebites (one case every few years, weekly cases?), what their knowledge of species is (e.g., are bites from non-venomous species treated "successfully"?), is there is a basic understanding of snake venoms and their effects.

- While participant 16 suggests that hospital treatment might be the standard, would all participants agree that SBE patients should be transferred to a hospital if possible? Could this differ depending on the snake species involved?

- Figure 3 would benefit from clearer conceptual structuring and presentation. Currently, it is not clear how and in which order different aspects are connected.

- The provided images were created how? Are these actual paintings by the authors or have they been created using AI tools?

**********

**Conclusions**

-Are the conclusions supported by the data presented?

-Are the limitations of analysis clearly described?

-Do the authors discuss how these data can be helpful to advance our understanding of the topic under study?

-Is public health relevance addressed?

Reviewer #1: See comments below.

Reviewer #2: The absence of resistance to biomedical interventions,particularly antivenom, reveals that Munduruku medical practice is not oppositional but integrative. The coexistence of these systems underscores that effective treatment is not defined by adherence to a single paradigm, but by the ability to navigate multiple knowledge systems simultaneously.

Reviewer #3: - The aspect of SBE as a punishment for not following social norms or rules of coexistence is interesting and reads as a broader and more positive interpretation of ‘compliance with dietary and sexual interdictions’. The results section suggests that this interpretation is particularly pronounced for women (e.g. in the context of pregnancy or menstruation). It would be helpful to clarify whether this reflects a gender difference or could also be reflective of the strong representation of midwives in the sample.

- The presentation of traditional treatment methods and their supposed effectiveness appears unbalanced. Suggesting any potential effectiveness of topical application of snake parts is highly speculative. On the other hand, unsuccessful treatments and possible complications from these applications are not addressed currently.

- Traditional interventions can cause delays and serious harm. This contributes to morbidity and mortality and has been documented, for example, in several countries in Africa. Some of the traditional remedies described in the manuscript would appear to come with the risk of serious infection, which should be stated and could be discussed in view of literature on harmful treatments complicating SBE.

- No references provided in lines 671 and 677 despite placeholder “(ref)”.

- A language edit and more concise writing would improve clarity of the section.

- Please clarify what ‘this translation’ refers to in line 788.

**********

**Editorial and Data Presentation Modifications?**

Use this section for editorial suggestions as well as relatively minor modifications of existing data that would enhance clarity. If the only modifications needed are minor and/or editorial, you may wish to recommend “Minor Revision” or “Accept”.

Reviewer #1: See comments below.

Reviewer #2: Lines 671 and 677 appear to be missing references.

Reviewer #3: (No Response)

**********

**Summary and General Comments**

Use this section to provide overall comments, discuss strengths/weaknesses of the study, novelty, significance, general execution and scholarship. You may also include additional comments for the author, including concerns about dual publication, research ethics, or publication ethics. If requesting major revision, please articulate the new experiments that are needed.

Reviewer #1: This manuscript explores the cultural, cosmological, and therapeutic understanding of snakebites among the Munduruku people in the Brazilian Amazon through qualitative interviews with Indigenous healers. The study develops an explanatory model integrating spiritual, social, and biological dimensions of envenomation. It highlights the role of traditional medicine, beliefs, and healing practices in snakebite management. The work emphasizes the importance of integrating Indigenous and biomedical approaches to improve culturally appropriate care. The authors should be congratulated for addressing an underexplored and highly relevant aspect of snakebite envenoming, particularly in Indigenous populations that are disproportionately affected. Despite, this I have some suggestions I hope help to improve this nice study.

Major issues

1. The study includes 19 participants, which is acceptable for qualitative research, but the representativeness across the broader Munduruku population and potential regional variability is not sufficiently discussed.

2. Although reflexivity is mentioned, the influence of researcher background and interpretation on data analysis should be more critically examined, particularly given the cultural sensitivity of the topic.

3. The study relies entirely on self-reported narratives. Integration with clinical data, reported snakebite cases, or health system outcomes would strengthen the translational relevance.

4. Some conclusions regarding healthcare integration and system design extend beyond the presented qualitative data.

5. The origin of the images created by the authors should be clarified. Some of the figures appear to be AI-generated or digitally illustrated, but no information is provided on how they were produced.

Minor issues

1. Terms such as “snakebite,” “envenomation,” and “accident” are used interchangeably and should be standardized.

2. Ethnographic figures are informative but would benefit from clearer legends and stronger linkage to the main text.

3. The description of qualitative analysis (codebook development, consensus process) could be slightly expanded for reproducibility.

4.

This is a valuable and original contribution that broadens the understanding of snakebite envenoming beyond biomedical paradigms. I was lucky to review this manuscript. I learned a lot.

Reviewer #2: This manuscript presents an ethnographically grounded exploration of the cultural, cosmological, and medical meanings of snakes and snakebites among the Munduruku people. It addresses an important intersection between medical anthropology, ethnobiology, and public health, particularly in a region where snakebite envenomation remains a major yet understudied issue.The paper is timely, relevant, and conceptually valuable, especially given increasing attention to Indigenous knowledge systems in healthcare

Reviewer #3: Title: Snakes and Snakebites in Munduruku Cosmology and Traditional Medicine in the Central Brazilian Amazon

The authors Dias et al. address an important problem: how to close the access gap to medical treatment for snakebite envenoming (SBE) in remote locations without adequate healthcare infrastructure. To allow for a decentralized antivenom treatment in partnership with traditional medicine, the authors investigated how snakebite is understood and managed by an Indigenous group in the Brazilian Amazon, the Munduruku people, as a basis to inform effective and culturally appropriate care. For this, the authors interviewed traditional Munduruku healers. The study is relevant and involves complexity in implementation and interpretation. However, the current manuscript form raises questions and is not always easy to follow. It would benefit from a clearer and more concise presentation of the topic, the findings and implications.

Introduction, also applicable to Results and Discussion

- Overall, the manuscript would benefit from linguistic revision, and in several places the text could be more concise and coherent. Avoid repetitions. Some sections are presented in considerable detail, but their connection to the overall argument is not always clear. Strengthening the coherence and explicitly linking these points to the main narrative would improve readability. For example, it is not obvious what common crops and trade has to do with the aim of the study and envenoming by Bothrops atrox is described over eleven lines in the introduction but this point is not addressed again later.

- Line 125 and 176: 17,997 individuals and 4,171; are these numbers actually known so precisely? Otherwise, rounded figures would be more appropriate.

Line 132: A more neutral phrasing could be considered such as ‘detailed’ rather than ‘excellent’ review.

**********

PLOS authors have the option to publish the peer review history of their article (what does this mean?). If published, this will include your full peer review and any attached files.

If you choose “no”, your identity will remain anonymous but your review may still be made public.

**Do you want your identity to be public for this peer review?**  For information about this choice, including consent withdrawal, please see our Privacy Policy.

Reviewer #1: No

Reviewer #2: No

Reviewer #3: No

[NOTE: If reviewer comments were submitted as an attachment file, they will be attached to this email and accessible via the submission site. Please log into your account, locate the manuscript record, and check for the action link "View Attachments". If this link does not appear, there are no attachment files.]

**Figure resubmission:**

While revising your submission, we strongly recommend that you use PLOS’s NAAS tool (https://ngplosjournals.pagemajik.ai/artanalysis) to test your figure files. NAAS can convert your figure files to the TIFF file type and meet basic requirements (such as print size, resolution), or provide you with a report on issues that do not meet our requirements and that NAAS cannot fix.

After uploading your figures to PLOS’s NAAS tool - https://ngplosjournals.pagemajik.ai/artanalysis, NAAS will process the files provided and display the results in the "Uploaded Files" section of the page as the processing is complete. If the uploaded figures meet our requirements (or NAAS is able to fix the files to meet our requirements), the figure will be marked as "fixed" above. If NAAS is unable to fix the files, a red "failed" label will appear above. When NAAS has confirmed that the figure files meet our requirements, please download the file via the download option, and include these NAAS processed figure files when submitting your revised manuscript.

**Reproducibility:**

To enhance the reproducibility of your results, we recommend that authors of applicable studies deposit laboratory protocols in protocols.io, where a protocol can be assigned its own identifier (DOI) such that it can be cited independently in the future. Additionally, PLOS ONE offers an option to publish peer-reviewed clinical study protocols. Read more information on sharing protocols at https://plos.org/protocols?utm_medium=editorial-email&utm_source=authorletters&utm_campaign=protocols

# Author comment: Author response to Decision Letter 1

**DOI:** 10.1371/journal.pntd.0014462.r002

14 May 2026

Attachment Submitted filename: Response letter.pdf

# Peer review: Decision Letter 1

**DOI:** 10.1371/journal.pntd.0014462.r003

**Authors:** José Gutiérrez, Emeka Dingwoke, José Gutiérrez, Emeka Dingwoke

8 Jun 2026

PNTD-D-26-00565R1

Snakes and Snakebites in the Munduruku Cosmology and Medicine, Central Brazilian Amazonia

PLOS Neglected Tropical Diseases

Dear Dr. Monteiro,

Thank you for submitting your manuscript to PLOS Neglected Tropical Diseases. After careful consideration, we feel that it has merit but does not fully meet PLOS Neglected Tropical Diseases's publication criteria as it currently stands. Therefore, we invite you to submit a revised version of the manuscript that addresses the points raised during the review process.

Please submit your revised manuscript by June 22. If you will need more time than this to complete your revisions, please reply to this message or contact the journal office at plosntds@plos.org. When you're ready to submit your revision, log on to https://www.editorialmanager.com/pntd/ and select the 'Submissions Needing Revision' folder to locate your manuscript file.

Please include the following items when submitting your revised manuscript:

* A letter that responds to each point raised by the editor and reviewer(s). You should upload this letter as a separate file labeled 'Response to Reviewers'. This file does not need to include responses to any formatting updates and technical items listed in the 'Journal Requirements' section below.

* A marked-up copy of your manuscript that highlights changes made to the original version. You should upload this as a separate file labeled 'Revised Manuscript with Track Changes'.

* An unmarked version of your revised paper without tracked changes. You should upload this as a separate file labeled 'Manuscript'.

If you would like to make changes to your financial disclosure, competing interests statement, or data availability statement, please make these updates within the submission form at the time of resubmission. Guidelines for resubmitting your figure files are available below the reviewer comments at the end of this letter.

As the corresponding author, your ORCID iD is verified in the submission system and will appear in the published article. PLOS supports the use of ORCID, and we encourage all coauthors to register for an ORCID iD and use it as well. Please encourage your coauthors to verify their ORCID iD within the submission system before final acceptance, as unverified ORCID iDs will not appear in the published article. Only the individual author can complete the verification step; PLOS staff cannot verify ORCID iDs on behalf of authors.

We look forward to receiving your revised manuscript.

Kind regards,

Emeka John Dingwoke, Ph.D

Academic Editor

PLOS Neglected Tropical Diseases

José María Gutiérrez

Section Editor

PLOS Neglected Tropical Diseases

Shaden Kamhawi

co-Editor-in-Chief

PLOS Neglected Tropical Diseases

orcid.org/0000-0003-4304-636XX

Paul Brindley

co-Editor-in-Chief

PLOS Neglected Tropical Diseases

orcid.org/0000-0003-1765-0002

**Additional Editor Comments (if provided):**

Dear Dr. Monteiro,

Thank you for submitting the revised version of your manuscript, “Snakes and Snakebites in the Munduruku Cosmology and Medicine, Central Brazilian Amazonia.” The reviewer acknowledged that the manuscript has improved substantially, with clearer presentation, additional methodological clarification, improved results presentation, and inclusion of study limitations.

However, you will see that the reviewer raised a minor concern regarding the presentation and discussion of traditional snakebite treatments. Specifically, it is noted that the current wording may inadvertently suggest potential therapeutic effectiveness of some traditional practices, especially through the citation of in vitro studies reporting inhibitory activity of certain plant metabolites and endogenous toxin inhibitors. While the manuscript appropriately avoids direct judgment of culturally sensitive practices, the discussion should be revised to ensure a more balanced interpretation and to avoid implying clinical efficacy in the absence of robust evidence.

Please revise this section to more clearly acknowledge the important limitations associated with the cited experimental studies and avoid statements that may be interpreted as supporting effectiveness in clinical practice. In addition, please consider incorporating relevant literature documenting potential harmful consequences or delays in seeking effective medical care associated with some traditional snakebite treatments in other settings. This should be done carefully and respectfully, without directly discrediting or judging the practices described by Munduruku healers, but rather to provide broader clinical and public health context and a more balanced discussion of benefit-risk considerations.

**Journal Requirements:**

If the reviewer comments include a recommendation to cite specific previously published works, please review and evaluate these publications to determine whether they are relevant and should be cited. There is no requirement to cite these works unless the editor has indicated otherwise.

1) Please amend your detailed Financial Disclosure statement. This is published with the article. It must therefore be completed in full sentences and contain the exact wording you wish to be published.

i) State the initials, alongside each funding source, of each author to receive each grant. For example: "This work was supported by the National Institutes of Health (####### to AM; ###### to CJ) and the National Science Foundation (###### to AM).".

If you did not receive any funding for this study, please simply state: u201cThe authors received no specific funding for this work.u201d

**Reviewers' comments:**

Reviewer's Responses to Questions

**Key Review Criteria Required for Acceptance?**

As you describe the new analyses required for acceptance, please consider the following:

**Methods**

-Are the objectives of the study clearly articulated with a clear testable hypothesis stated?

-Is the study design appropriate to address the stated objectives?

-Is the population clearly described and appropriate for the hypothesis being tested?

-Is the sample size sufficient to ensure adequate power to address the hypothesis being tested?

-Were correct statistical analysis used to support conclusions?

-Are there concerns about ethical or regulatory requirements being met?

Reviewer #1: NA

Reviewer #3: n/a

**********

**Results**

-Does the analysis presented match the analysis plan?

-Are the results clearly and completely presented?

-Are the figures (Tables, Images) of sufficient quality for clarity?

Reviewer #1: NA

Reviewer #3: n/a

**********

**Conclusions**

-Are the conclusions supported by the data presented?

-Are the limitations of analysis clearly described?

-Do the authors discuss how these data can be helpful to advance our understanding of the topic under study?

-Is public health relevance addressed?

Reviewer #1: NA

Reviewer #3: n/a

**********

**Editorial and Data Presentation Modifications?**

Use this section for editorial suggestions as well as relatively minor modifications of existing data that would enhance clarity. If the only modifications needed are minor and/or editorial, you may wish to recommend “Minor Revision” or “Accept”.

Reviewer #1: NA

Reviewer #3: n/a

**********

**Summary and General Comments**

Use this section to provide overall comments, discuss strengths/weaknesses of the study, novelty, significance, general execution and scholarship. You may also include additional comments for the author, including concerns about dual publication, research ethics, or publication ethics. If requesting major revision, please articulate the new experiments that are needed.

Reviewer #1: The authors have replied all major comments and issues. I have no further suggestions. Congratulations! This is a nice piece of work.

Reviewer #3: Title: Snakes and Snakebites in Munduruku Cosmology and Traditional Medicine in the Central Brazilian Amazon

The authors have addressed most previous concerns and suggestions. They submitted a revised manuscript, more clearly and coherently written with additional clarifications in the methods and result sections, including figure 3. Limitations of the study, in particular the possibility of response bias / social desirability bias, are described. While some questions remain (e.g., how much experience with snakebites have healers), it as acknowledged that this is beyond the scope of this study.

One aspect that, in my view, could still be addressed more appropriately is the presentation and discussion of traditional treatments. In its current form, it may be understood as suggesting some form of effectiveness and the citation of studies reporting in vitro inhibitory potential reinforces this impression: >Although their efficacy cannot be confirmed, some biological evidence may partially support these practices: snakes possess endogenous toxin inhibitors, such as metalloproteinase and phospholipase inhibitors [67], and several plant metabolites, including those cited by Munduruku healers, have shown in vitro inhibitory potential [68,69]. <

However, such studies come with considerable limitations (e.g., long pre-incubation periods; non-physiological concentrations, specific vs. broad enzyme inhibitory effects) and do not appear to adequately support claims that traditional treatments might be effective in clinical practice. The authors argue that judgments about traditional practices, especially those of a mythical-religious nature, are highly sensitive and should be avoided. However, in its current form the manuscripts could be perceived as presenting such approaches in a rather positive light. Thus, rephrasing this part is recommended to avoid implying efficacy. In addition, the authors should consider to reference literature that documented harmful consequences of traditional SBE treatments in other parts of the world, to introduce the concept that such methods can be detrimental, without directly judging the methods described by Munduruku healers in this study.

**********

PLOS authors have the option to publish the peer review history of their article (what does this mean?). If published, this will include your full peer review and any attached files.

If you choose “no”, your identity will remain anonymous but your review may still be made public.

**Do you want your identity to be public for this peer review?**  For information about this choice, including consent withdrawal, please see our Privacy Policy.

Reviewer #1: No

Reviewer #3: No

[NOTE: If reviewer comments were submitted as an attachment file, they will be attached to this email and accessible via the submission site. Please log into your account, locate the manuscript record, and check for the action link "View Attachments". If this link does not appear, there are no attachment files.]

**Figure resubmission:**

While revising your submission, we strongly recommend that you use PLOS’s NAAS tool (https://ngplosjournals.pagemajik.ai/artanalysis) to test your figure files. NAAS can convert your figure files to the TIFF file type and meet basic requirements (such as print size, resolution), or provide you with a report on issues that do not meet our requirements and that NAAS cannot fix.

After uploading your figures to PLOS’s NAAS tool - https://ngplosjournals.pagemajik.ai/artanalysis, NAAS will process the files provided and display the results in the "Uploaded Files" section of the page as the processing is complete. If the uploaded figures meet our requirements (or NAAS is able to fix the files to meet our requirements), the figure will be marked as "fixed" above. If NAAS is unable to fix the files, a red "failed" label will appear above. When NAAS has confirmed that the figure files meet our requirements, please download the file via the download option, and include these NAAS processed figure files when submitting your revised manuscript.

**Reproducibility:**

To enhance the reproducibility of your results, we recommend that authors of applicable studies deposit laboratory protocols in protocols.io, where a protocol can be assigned its own identifier (DOI) such that it can be cited independently in the future. Additionally, PLOS ONE offers an option to publish peer-reviewed clinical study protocols. Read more information on sharing protocols at https://plos.org/protocols?utm_medium=editorial-email&utm_source=authorletters&utm_campaign=protocols

# Author comment: Author response to Decision Letter 2

**DOI:** 10.1371/journal.pntd.0014462.r004

9 Jun 2026

Attachment Submitted filename: Response letter_R2.docx

# Peer review: Decision Letter 2

**DOI:** 10.1371/journal.pntd.0014462.r005

**Authors:** José Gutiérrez, Emeka Dingwoke, José Gutiérrez, Emeka Dingwoke, José Gutiérrez, Emeka Dingwoke

11 Jun 2026

Dear Dr. Monteiro,

We are pleased to inform you that your manuscript 'Snakes and Snakebites in the Munduruku Cosmology and Medicine, Central Brazilian Amazonia' has been provisionally accepted for publication in PLOS Neglected Tropical Diseases.

Before your manuscript can be formally accepted you will need to complete some formatting changes, which you will receive in a follow up email. A member of our team will be in touch with a set of requests.

Please note that your manuscript will not be scheduled for publication until you have made the required changes, so a swift response is appreciated.

IMPORTANT: The editorial review process is now complete. PLOS will only permit corrections to spelling, formatting or significant scientific errors from this point onwards. Requests for major changes, or any which affect the scientific understanding of your work, will cause delays to the publication date of your manuscript.

Should you, your institution's press office or the journal office choose to press release your paper, you will automatically be opted out of early publication. We ask that you notify us now if you or your institution is planning to press release the article. All press must be co-ordinated with PLOS.

Thank you again for supporting Open Access publishing; we are looking forward to publishing your work in PLOS Neglected Tropical Diseases.

Best regards,

Emeka John Dingwoke, Ph.D

Academic Editor

PLOS Neglected Tropical Diseases

José María Gutiérrez

Section Editor

PLOS Neglected Tropical Diseases

Shaden Kamhawi

co-Editor-in-Chief

PLOS Neglected Tropical Diseases

orcid.org/0000-0003-4304-636XX

Paul Brindley

co-Editor-in-Chief

PLOS Neglected Tropical Diseases

orcid.org/0000-0003-1765-0002

***********************************************************

p.p1 {margin: 0.0px 0.0px 0.0px 0.0px; line-height: 16.0px; font: 14.0px Arial; color: #323333; -webkit-text-stroke: #323333}span.s1 {font-kerning: none

Reviewer's Responses to Questions

**Key Review Criteria Required for Acceptance?**

As you describe the new analyses required for acceptance, please consider the following:

**Methods**

-Are the objectives of the study clearly articulated with a clear testable hypothesis stated?

-Is the study design appropriate to address the stated objectives?

-Is the population clearly described and appropriate for the hypothesis being tested?

-Is the sample size sufficient to ensure adequate power to address the hypothesis being tested?

-Were correct statistical analysis used to support conclusions?

-Are there concerns about ethical or regulatory requirements being met?

Reviewer #3: n/a

**********

**Results**

-Does the analysis presented match the analysis plan?

-Are the results clearly and completely presented?

-Are the figures (Tables, Images) of sufficient quality for clarity?

Reviewer #3: n/a

**********

**Conclusions**

-Are the conclusions supported by the data presented?

-Are the limitations of analysis clearly described?

-Do the authors discuss how these data can be helpful to advance our understanding of the topic under study?

-Is public health relevance addressed?

Reviewer #3: n/a

**********

**Editorial and Data Presentation Modifications?**

Use this section for editorial suggestions as well as relatively minor modifications of existing data that would enhance clarity. If the only modifications needed are minor and/or editorial, you may wish to recommend “Minor Revision” or “Accept”.

Reviewer #3: n/a

**********

**Summary and General Comments**

Use this section to provide overall comments, discuss strengths/weaknesses of the study, novelty, significance, general execution and scholarship. You may also include additional comments for the author, including concerns about dual publication, research ethics, or publication ethics. If requesting major revision, please articulate the new experiments that are needed.

Reviewer #3: The authors have addressed most of the concerns and suggestions raised. While not all issues have been fully resolved, the manuscript has significantly improved and the findings are valuable. Overall, I recommend publication.

**********

PLOS authors have the option to publish the peer review history of their article (what does this mean?). If published, this will include your full peer review and any attached files.

If you choose “no”, your identity will remain anonymous but your review may still be made public.

**Do you want your identity to be public for this peer review?**  For information about this choice, including consent withdrawal, please see our Privacy Policy.

Reviewer #3: No

# Editor report: Acceptance letter

**DOI:** 10.1371/journal.pntd.0014462.r006

**Authors:** José Gutiérrez, Emeka Dingwoke, José Gutiérrez, Emeka Dingwoke, José Gutiérrez, Emeka Dingwoke

Dear Dr. Monteiro,

We are delighted to inform you that your manuscript, "Snakes and Snakebites in the Munduruku Cosmology and Medicine, Central Brazilian Amazonia," has been formally accepted for publication in PLOS Neglected Tropical Diseases.

We have now passed your article onto the PLOS Production Department who will complete the rest of the publication process. All authors will receive a confirmation email upon publication.

The corresponding author will soon be receiving a typeset proof for review, to ensure errors have not been introduced during production. Please review the PDF proof of your manuscript carefully, as this is the last chance to correct any scientific or type-setting errors. Please note that major changes, or those which affect the scientific understanding of the work, will likely cause delays to the publication date of your manuscript. Note: Proofs for Front Matter articles (Editorial, Viewpoint, Symposium, Review, etc...) are generated on a different schedule and may not be made available as quickly.

Soon after your final files are uploaded, the early version of your manuscript will be published online unless you opted out of this process. The date of the early version will be your article's publication date. The final article will be published to the same URL, and all versions of the paper will be accessible to readers.

For Research Articles, you will receive an invoice from PLOS for your publication fee after your manuscript has reached the completed accept phase. If you receive an email requesting payment before acceptance or for any other service, this may be a phishing scheme. Learn how to identify phishing emails and protect your accounts at https://explore.plos.org/phishing.

Thank you again for supporting open-access publishing; we are looking forward to publishing your work in PLOS Neglected Tropical Diseases.

Best regards,

Shaden Kamhawi

co-Editor-in-Chief

PLOS Neglected Tropical Diseases

Paul Brindley

co-Editor-in-Chief

PLOS Neglected Tropical Diseases
